# Patterns of genomic divergence in sympatric and allopatric speciation of three Mihoutao (*Actinidia*) species

**DOI:** 10.1093/hr/uhac054

**Published:** 2022-03-03

**Authors:** Yongbo Liu, Wenhao Yu, Baofeng Wu, Junsheng Li

**Affiliations:** State Key Laboratory of Environmental Criteria and Risk Assessment, Chinese Research Academy of Environmental Sciences, 8 Dayangfang, Beijing 100012, China

## Abstract

Isolation by geographic distance is involved in the formation of potential genomic islands and the divergence of genomes, which often result in speciation. The mechanisms of sympatric and allopatric speciation associated with geographic distance remain a topic of interest to evolutionary biologists. Here, we examined genomic divergence in three *Actinidia* species from large-scale sympatric and allopatric regions. Genome sequence data revealed that hexaploid *Actinidia deliciosa* originated from *Actinidia chinensis* and supported the speciation-with-gene-flow model in sympatric regions. The common ancestor of *Actinidia setosa* and *A. deliciosa* migrated from the mainland to the Taiwan Island ~2.91 Mya and formed *A. setosa* ~0.92 Mya, and the speciation of *A. setosa* is consistent with the divergence-after-speciation model with selective sweeps. Geographic isolation resulted in population contraction and accelerated the process of lineage sorting and speciation due to natural selection. Genomic islands contained genes associated with organ development, local adaptation, and stress resistance, indicating selective sweeps on a specific set of traits. Our results highlight the patterns of genomic divergence in sympatric and allopatric speciation, with the mediation of geographic isolation in the formation of genomic islands during *Actinidia* speciation.

## Introduction

Speciation based on geographical distance can be categorized into sympatric and allopatric types [1]. Sympatric speciation occurs in the same geographic location without isolation, and natural selection is the most common driver [2]. Allopatric speciation is one of the main mechanisms of population divergence and species formation [3] because geographic isolation decreases gene flow and thus increases genetic distance between allopatric populations [4]. For example, mountains, rivers, oceans, and isolated island-like habitats alter the genetic structure of populations and drive population differentiation [5].

Understanding the process of speciation and post-speciation ecological adaptation has been advanced by studies of genomic polymorphism and divergence among the genomes of closely related species [6, 7], and genomic islands of divergence containing functional variants undergoing speciation have been widely observed [8]. Such genomic islands are generated by variation in gene flow between loci, ancestral diverged haplotypes, recurrent background selection with genomic recombination, and ecological adaptation [8–10]. The identification of genomic islands of population divergence or speciation requires information on ancient polymorphisms, gene flow, and natural selection on traits [8, 11], as genomic divergence varies with evolutionary history, length of isolation, and adaptation to local post-speciation selection [12].

Genomic data provide evidence for the role of tectonic evolution in speciation and bioregion formation [10, 13, 14]. This study aimed to understand the genomic basis for speciation of three closely related wild *Actinidia* species (*Actinidia chinensis*, *Actinidia deliciosa* and *Actinidia setosa*) and to elucidate their adaptation to local selection in nature, employing population genomic resequencing. The *Actinidia* genus (named Mihoutao in Chinese) originated in China and is now mainly distributed from the Qinling Mountains to the Hengduan Mountains. The geographic distribution of *A. chinensis* and *A. deliciosa* has sympatric regions, with the former predominating in the eastern part of China and the latter in the west, whereas *A. setosa*, an endemic species, is restricted to the island of Taiwan and is allopatric to the other two species. Island and mountain topography separate biological populations and accelerate population differentiation and speciation because of limited gene flow between populations [14–17]. Thus, the three wild kiwifruit species provide ideal subjects for investigating the role of geographic isolation in sympatric and allopatric speciation.

The phylogenetic relationships of the three species are still controversial. *A. chinensis* has diploid and tetraploid wild individuals, whereas *A. deliciosa* exhibits complex ploidy in nature, ranging from tetraploid to octoploid [18]. *A. setosa* has only diploid individuals. *A. chinensis* and *A. deliciosa* have been described as two distinct species based on morphological features such as hair types, over-wintering buds, and mature fruit flesh color [19]. *A. setosa* was once considered to be a variety of *A. chinensis* [19, 20], but it was subsequently reclassified as a distinct species [21]. Huang et al. (2014) reclassified them as three varieties of a single species [22], although morphological intermediates and hybrid forms between *A. deliciosa* and *A. chinensis* have been found in nature [23]. Chloroplastic and mitochondrial DNA sequence data suggest that *A. deliciosa* and *A. chinensis* represent a species complex from which *A. setosa* diverged [24].

## Materials and methods

### Plants and sampling

The three species *A. chinensis*, *A. deliciosa*, and *A. setosa* are closely related but show distinct morphological differences; the main difference is the trichomes in fruits, leaves, and stems. *A. chinensis* fruits are subglobose and tomentose or glabrous, *A. deliciosa* have subglobose to cylindrical fruits that are densely hispid, and *A. setosa* fruits are subglobose or ellipsoidal and densely hispid [25].

We sampled the three *Actinidia* species in nature from six *A. chinensis* populations, three *A. deliciosa* populations, and one *A. setosa* population sampled from three mountains on the Taiwan Island (Table S1). Leaves from more than 500 individual plants were sampled randomly. The ploidy of individual plants was measured by flow cytometry 1–2 days after collecting fresh leaves (Supplementary Fig. S1) [26]. In addition, *Actinidia arguta* was sampled from Shaanxi province as an outgroup.

### Library preparation

We extracted genomic DNA from 187 samples of three species from nine natural populations (1.5 μg per sample). Following the manufacturer’s recommendations, DNA sequencing libraries were generated using a TruSeq Nano DNA HT sample preparation kit (Illumina, USA). DNA samples were fragmented by sonication to a size of 350 bp, and further PCR amplification was performed to end-polish, A-tail, and ligate the DNA fragments with the full-length adapter for Illumina sequencing. The PCR products were purified (AMPure XP system), and the size distributions of the libraries were analyzed and quantified using an Agilent 2100 Bioanalyzer and real-time PCR, respectively.

### Genome sequencing and quality control

Whole genomes of 187 samples were sequenced on the Illumina HiSeq 2500 platform, generating 1346 Gb of raw data. We removed low-quality paired-end reads (>10 nt aligned to the adaptor, allowing for ≤10% mismatches; ≥10% unidentified nucleotides [N]; >50% bases with phred quality <5; putative PCR duplicates) and retained 935.5 Gb of high-quality paired-end reads (Q20 ≥ 94.2% and Q30 ≥ 86.7%) (Table S1). After removing low-quality samples, a total of 139 samples (including one outgroup) with high-quality data were used for subsequent analyses, representing 12–15 plants per population (Table S1). Genome alignment of the *Actinidia* sequences indicated an average depth of 7.7-fold and coverage of 93.3% relative to the reference genome of *A. chinensis* (the heterozygous kiwifruit “Hongyang”) (Table S2) [27].

### Read mapping and SNP calling

The remaining high-quality paired-end reads were mapped to the diploid *A. chinensis* “Hongyang” reference genome [27] using BWA (Burrows-Wheeler Aligner) version 0.7.8 [28] with the parameters “mem -t 4 -k 32 -M” [28]. SAMtools was used to remove duplicate reads [29]. After alignment, single nucleotide polymorphisms (SNPs) were called on a population scale using a Bayesian approach implemented in SAMtools (version 1.3). We then calculated genotype likelihoods and allele frequencies from reads for each individual at each genomic location using a Bayesian approach. SNPs were identified using the “mpileup” command with the parameters “-q 1 -C 50 -t SP -t DP -m 2 -F 0.002”. To exclude SNP-calling errors caused by incorrect mapping or InDels, SNPs were filtered (coverage depth ≥2 and ≤50, RMS mapping quality ≥20, maf ≥0.05, miss ≤0.1). Consequently, 3 556 911 high quality SNPs remained after filtering from the initial collection of 44 549 362 raw SNPs.

To solve the difference in SNPs from diploid and polyploid populations, we have called SNPs with the GATK software setting HaplotypeCaller “—ploidy 4” for tetraploid populations and “—ploidy 6” for hexaploid populations. However, most software packages, including ADMIXTURE, PLINK, and VCFtools, cannot deal with multiallelic SNPs but only with biallelic SNPs, which limits the next analysis. We only obtained an NJ tree based on multiallelic SNPs for polyploid populations after low-quality SNP filtering, and the tree was the same as that constructed from biallelic SNPs. This may indicate that the analysis of biallelic SNPs is reliable for polyploid populations, as previous studies have suggested [30]. In addition, we analyzed genetic structure using only homozygous or heterogenous biallelic SNPs of tetraploid and hexaploid individuals (data not shown), and the results were similar to those obtained using all SNPs. Thus, the next analysis was performed using all biallelic SNPs. SNPs were annotated based on comparisons to the reference genome using ANNOVAR [31].

### Phylogenetic tree and population structure

Individual-based maximum-likelihood (ML) and neighbor-joining (NJ) trees were constructed based on the p-distance using TreeBeST v1.9.2 software [32]. The population genetic structure was analyzed using an expectation maximization algorithm implemented in frappe [33]. The number of assumed genetic clusters K was set from 2 to 8, with 10 000 iterations for each run. Principal component analysis (PCA) was performed to evaluate the genetic structure of populations using GCTA software [34].

### Genetic diversity and LD

Nucleotide diversity (θπ) of each population was calculated using a sliding-window approach (40-kb windows with 20-kb increments). We calculated the correlation coefficient (r^2^) of alleles to estimate linkage disequilibrium (LD) using Haploview [35]. The average r^2^ value was calculated for pairwise markers in 500-kb windows and averaged across the whole genome, and LD decay figures were drawn using an R script.

### Demographic history and gene flow

To investigate the demographic history of the three species, the effective population size (Ne) of the three *Actinidia* species was estimated over the last 10 million years using a pairwise sequentially Markovian coalescence (PSMC) model [36]. Parameters were set to “- N30 -t15 -r5 -p’4 þ 25^*^2 þ 4 þ 6’”. The mutation rate (μ) was set to 5.4 × 10^−9^ per base per generation [37], and the generation time (g) was set to six years because kiwifruit begin to bear fruit at about three years of age.

We inferred the demographic history of the three species using the diffusion approximation for demographic inference (∂a∂i) approach [38]. We used the model θ = 4 × μ × N_ref_ × L, where μ was the mutation rate (5.4 × 10^−9^) and L was the number of SNPs. To ensure selective neutrality, only the SNPs within intergenic regions of autosomal chromosomes were used. We evaluated model fitting and optimized model selection using a composite-likelihood ratio test. The optimal model was selected with the highest log-likelihood value from five constructed divergence models.

The divergence time of these natural *Actinidia* populations in China was estimated under a Bayesian approach using BEAST v2.3.4 [39] according to a neutral mutation rate (μ = 5.4 × 10^−9^). MCMC runs were performed for 10 000 000 generations, with sampling every 1000 generations following a burn-in of the initial 50% of cycles. We examined the sampling adequacy and convergence of the chains to a stationary distribution using Tracer v1.5. TreeAnnotator summarized a post burn-in tree and produced a maximum clade credibility chronogram showing mean estimated divergence time with 95% HPD intervals [40].

A population-level admixture analysis was conducted to detect historical gene flow between wild *A. chinensis* populations using TreeMix v.1.12 [41] with the command “-i input -bootstrap -k 10000 -m migration events -o output”. The populations were regarded as candidates around which potential migration edges were added, generating new arrangements of the ML tree accounting for migration events [41]. From one to ten migration events were gradually added to the ML tree until 98% of the variance between the populations could be explained.

### Identification of genomic windows and selected regions

To identify genome-wide selective sweeps associated with natural adaptation, we measured genome-wide variation for the pairwise comparisons of the three species (*A. chinensis*, *A. deliciosa*, and *A. setosa*) and effects of geographic isolation on speciation. Geographic isolation included three scenarios, i.e. oceanic island (TWS + ZSC as one oceanic population *vs.* TPC + YAC + SYC + JZD + ZLD as one mainland population), lake island (JSC island *vs.* YMC land), and mountain isolation (four diploid populations, TPC, YAC, SYC, and YMC). A sliding window approach (40-kb window and 20-kb step size) was used to identify selected regions associated with specific adaptations. The genome-wide distribution of *F*_ST_ and Tajima’s D was calculated using VCFtools [42]. Absolute divergence (*D_XY_*) was calculated from sample allele frequency posterior probabilities at each site and was then averaged over each 20-kb window using an in-house python script.

Population-scaled recombination rates (ρ = 4N_e_c) were estimated for diploid species between each pair of SNPs and then weight-averaged over each 20-kb window using the program LDhelmet v.1.10 [43] with default parameters (100 000 burn-in iterations, 1 000 000 Markov chain iterations, and a block penalty of 50). We only used retained windows with at least 20 SNPs after filtering SNPs with MAF > 5% to minimize the effects of rare variants [9].

Genomic regions with significant high *F*_ST_ values (Z-*F*_ST_ > 2, corresponding to a top 5% level) were identified as highly divergent regions and designated genomic islands. Z-F_ST_ was defined by the formula Z-F_ST_ = (F_ST_ × F_ST_′)/std-F_ST_ (Han et al. 2017), where F_ST_ was a per-window estimate, and F_ST_′ and std-F_ST_ were the mean and standard deviation of F_ST_ across windows. To identify regions in the genome linked to potential lineage-specific adaptations, genetic diversity, the number of SNPs, Tajima’s D, and *D_XY_* in genomic islands were compared with genomic background for any pairwise comparisons. We compared characteristics of genomic islands in the pairwise comparisons.

We considered the windows with the top 5% *F*_ST_ and log2 (θπ ratio) values simultaneously as candidate outliers under strong selective sweeps for pairwise comparisons of the three species and geographic isolation scenarios. All outlier windows were assigned to corresponding SNPs and genes.

ANOVA and randomization analysis were used to detect differences in *D_XY_* between genomic islands and the rest of the genome for each pairwise comparison using R software.

### Annotation analysis of selection regions

We annotated genes in outlier genomic regions using the *A. chinensis* “Hongyang” reference genome. Enrichment analyses of these genes were conducted using the Gene Ontology (GO) [44] and KEGG (http://www.genome.jp/kegg/) databases. Significant enriched gene function was tested using a false discovery rate (FDR) with a corrected binomial distribution probability approach at the level of P < 0.0545. The functions of the candidate genes were inferred from the NCBI (http://www.ncbi.nlm.nih.gov) and PIR (http://www.uniprot.org) databases.

### GWAS analysis

A total of 3 556 911 SNPs (MAF ≥0.05; missing rate ≤0.1, depth ≥2) were used in GWAS for the trichome trait. The trichome trait was defined here as a presence or absence trait; *A. chinensis* had a value of “0” (absence) and *A. deliciosa* a value of “1” (presence). To reduce the risk of false positives from population structure, we conducted the analysis with the genome-wide efficient mixed-model association (GEMMA) software package [45]. For mixed linear model analysis, we used the following equation:

\begin{equation*} y = X\alpha + S\beta + K\mu + e. \end{equation*}

where *α* and *β* are fixed effects representing marker effects and non-marker effects, respectively; *y* represents phenotype; and *μ* represents unknown random effects. *X*, *S*, and *K* are the incidence matrices for *α*, *β*, and *μ*, respectively, and *e* is a vector of random residual effects. The *S* matrix for population-structure correction was built up using the top three PCs. We built up the *K* matrix using the matrix of simple matching coefficients.

## Results

### Genome resequencing and population structure

After stringent quality filtering, we identified a total of 3 556 911 high-quality SNPs from the initial collection of 44 549 362 raw SNPs. The number of SNPs varied among the populations and ploidy races, and the percentage of heterogeneous SNPs in hexaploid and tetraploid populations was higher than that in diploid populations, with the lowest percentage in the TWS population (Table S3).

**Figure 1:**
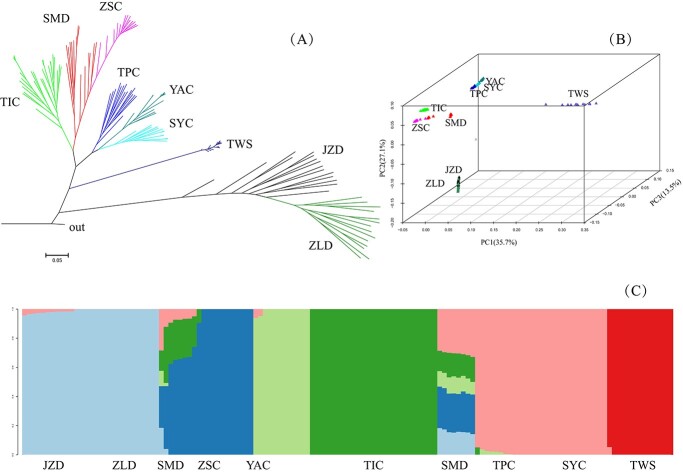
Phylogenetic and population genetic analyses of *A. chinensis*, *A. deliciosa*, and *A. setosa*. (A) Phylogenetic network inferred using the ML method based on genome-wide SNPs. (B) PCA plots of SNP data for the nine natural populations. (C) Population structure bar plots show the clustering of samples into six clusters (best K = 6). Each vertical bar indicates a single individual, and the height of colored bars represents the proportion of assignments to that cluster.

Based on phylogenetic analysis, individuals from the same population clustered together, and each of the three species clustered into a different subclade (Fig. 1A). *A. setosa* (TWS) had short clade branch lengths. The ZSC population (tetraploid *A. chinensis*) and the SMD population (tetraploid *A*. *deliciosa*) clustered together and were located in the middle of diploid TIC and TPC + YAC + SYC.

Principal component analysis (PCA) of SNP genotypes among all individuals clearly revealed a history of population divergence (Fig 1B). The first principal component (PC1; variance explained = 35.7%) separated *A. setosa* (TWS) from the other two species, and the second principal component (PC2; variance explained = 27.1%) separated hexaploid *A. deliciosa* (JZD and ZLD) from the other lineages. The geographically isolated populations (eastern TIC vs. central SYC + YAC + TPC) within *A. chinensis* were separated by the third principal component (variance explained = 13.5%). The PCA results were consistent with the clustering analyses that showed geographic populations (best K = 6) (Fig. 1C). Three clusters corresponded to the three species (K = 3), and when K = 2, two clusters were *A. chinensis* and *A. setosa,* and the hexaploid *A. deliciosa* included the other two species information (Fig. 1C). The tetraploid *A. deliciosa* (SMD) had a genetic background from other populations in cases of all K values, indicating that it is likely to be an intermediate phenotype produced in the heterozygote, which may be attributed to shared ancestral polymorphism or introgression events (Fig. 1C).

The genetic diversity (π) and population-scaled recombination rate (ρ = 4N_e_c) of *A. setosa* were the lowest of the three species (Fig. S2, Table S4). *A. deliciosa* exhibited the highest genetic diversity and showed much more extensive genome-wide LD, which probably resulted from asymmetric genetic introgression from *A. chinensis* into *A. deliciosa*. The Tajima’s D values of the three species were significantly greater than zero, and *A. deliciosa* showed the highest value (Table S4).

### Demographic history

**Figure 2:**
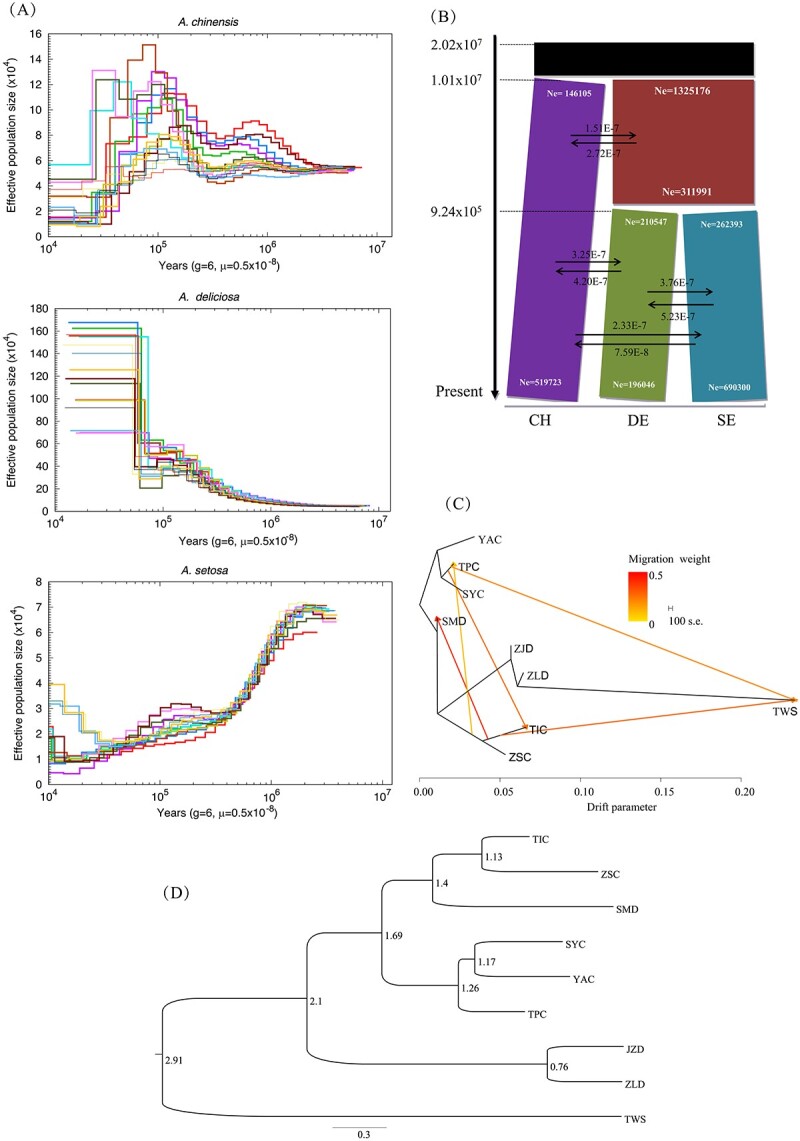
Speciation and demographic history of the three *Actinidia* species. (A) PSMC analysis results for *N*e over the last 10^7^ years. (B) ∂*a*∂*i* analysis showing the demographic history of the three *Actinidia* species (CH, *A. chinensis*; DE, *A. deliciosa*; SE, *A. setosa*) from ~2.02 × 10^7^ years ago to the present. Species divergences occurred at 1.01 × 10^7^ and 9.24 × 10^5^ years ago. The average number of migrants per year between species is shown between the black arrows. (C) Phylogenetic network of the inferred relationships among the nine natural populations. Arrows indicate migration events, and a spectrum of heat colors indicates the migration weights of the migration events. The scale bar shows ten times the average standard error of the entries in the sample covariance matrix. (D) Divergence times between natural populations estimated using BEAST analysis.

The effective population size (*Ne*) of *A. chinensis* and *A. setosa* began to decrease at ~1 Mya, experienced an increase at ~0.3–0.5 Mya, and then experienced a bottleneck event at ~100 kya (Fig. 2A) around the Marine Isotope Stage 5 (MIS, 80–130 kya) during the last major interglacial period. *A. deliciosa* increased rapidly after ~100 kya and exhibited the largest historical *Ne* values (Fig. 2A). The smallest historical *Ne* in *A. setosa* was consistent with its restricted island distribution in Taiwan.

A ∂a∂i analysis revealed that the last common ancestor of the three species was present ~20.2 Mya (Fig. 2B), consistent with one leaf fossil record of *Actinidia* (20–26 Mya, during the Miocene Epoch) [46]. The divergence time between *A. chinensis* and the other two species was ~10.1 Mya (Fig. 2B), and *A. deliciosa* and *A. setosa* diverged ~0.92 Mya. The degree of gene flow between *A. deliciosa* and *A. setosa* was the highest, followed by that between *A. deliciosa* and *A. chinensis*, and the lowest was between *A. chinensis* and *A. setosa* (Fig. 2B).

At the population scale, BEAST analysis showed that the TWS population diverged from the inland populations ~2.91 Mya (95% confidence interval 2.87–2.95) (Fig. 2C). The two hexaploid populations ZLD and JZD diverged 0.76 Mya and together diverged from the other populations 2.10 Mya. The divergence times between these diploid populations were ~1.13–1.69 Mya (Fig. 2D). TreeMix analysis showed gene flow in local regions, from TIC (diploid) to SMD (tetraploid) in eastern China, and from north to south, including from TPC to TIC and then to TWS (Fig. 2C).

### Genomic islands of divergence

**Figure 3:**
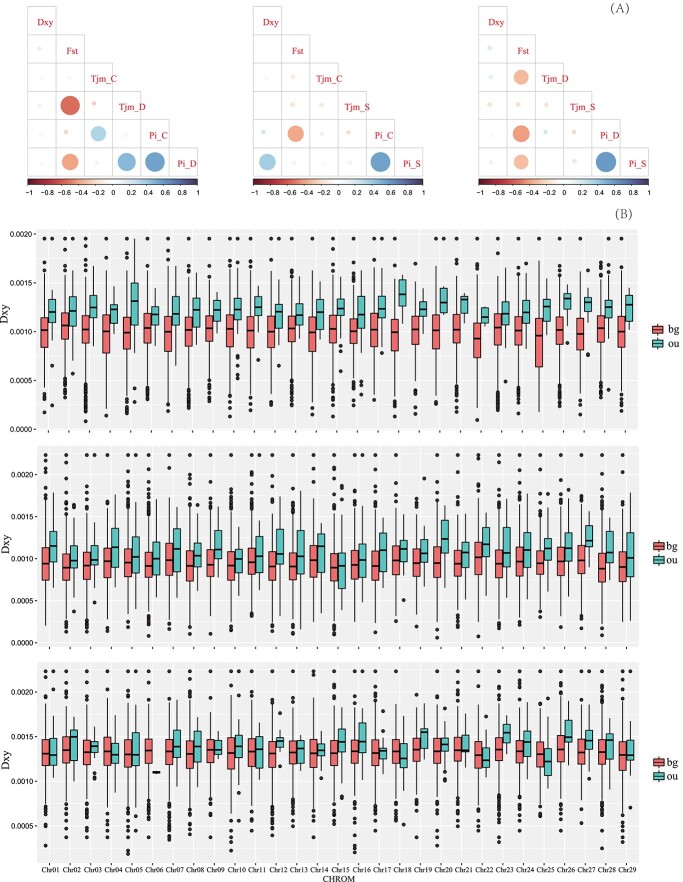
The heterogeneity of genomic divergence in the three species. (A) Correlations of absolute divergence (Dxy), genetic divergence (FST), Tajima’s D (Tjm), and nucleotide diversity (Pi) for the three pairwise comparisons of *A. chinensis* (C), *A. deliciosa* (D), and *A. setosa* (S). Blue and red circles indicate a positive or negative correlation, respectively. The color intensity and circle size are proportional to the Spearman’s correlation coefficient. (B) Values of Dxy in genomic islands compared with the background genome in the three pairwise comparisons of *A. chinensis*–*A. deliciosa* (top), *A. chinensis*–*A. setosa* (middle), and *A. deliciosa*–*A. setosa* (bottom). The “ou” represents genomic islands, and “bg” represents the background genome.

The landscape of the weighted *F_ST_* distribution varied in the three pairwise species comparisons (Kolmogorov–Smirnov test, P < 2.2e−16) (Fig. S3, Table S4). Genetic divergence (*F_ST_*) was highly heterogeneous along genomes (Fig. S4A) and negatively related to nucleotide diversity (π) and Tajima’s D (Fig. S5; Fig. 3A). Genomic regions with Z-transformed *F_ST_* values (Z-*F_ST_*) ≥2, corresponding to 4–5% of windows, were considered to be genomic islands for pairwise comparisons. We identified 56 common genomic islands among the three pairwise comparisons. These genomic islands showed reduced π and Tajima’s D values, indicating that linked selection had acted on these islands (Table S5).

We observed significantly elevated *D_XY_* in genomic islands compared with the rest of the genomes in pairwise comparisons of the three species (P < 0.001; randomization test) (Fig. 3B; Table S5), irrespective of whether the comparisons were between sympatric (*A. chinensis* and *A. deliciosa*) or allopatric species (*A. setosa* vs. *A. chinensis* or *A. deliciosa*). Genomic islands showed a significantly reduced recombination rate (ρ) compared with the rest of the genome for the three pairwise comparisons (P < 2.2e−16, Mann–Whitney U test; Table S5).

To characterize the genetic basis of this speciation, we conducted selective sweeps to identify genes with the top 5% of *F*_ST_ (genomic islands) and log2 (θπ ratio) simultaneously (Fig. S4B). Among a total of 719 genes in these genomic islands under positive selection, 543 genes were identified (Table S6). Of these, 55 genes were shared among the three comparisons, which indicates the presence of diverged haplotypes in their ancestral population possibly as a result of strong divergent selection. No highly enriched GO terms or KEGG pathways were found for the three pairwise comparisons. Some genes were associated with local adaptation (e.g. WRKY 40), organ development (e.g. ZFP, bHLH, MYB), and reproductive isolation (e.g. FPA, KAN2, MS1) (Table S6).

### Divergence of isolation

The genetic diversity (θπ) of oceanic and lake islands was not significantly different from those of land populations (Table S7). *F_ST_* values of oceanic isolation (0.2–0.4) were significantly higher than the value for lake isolation (<0.2) (Fig. S6). The four mountain populations of diploid *A. chinensis* did not show an isolation-by-distance effect of *F_ST_* (Table S7).

**Figure 4:**
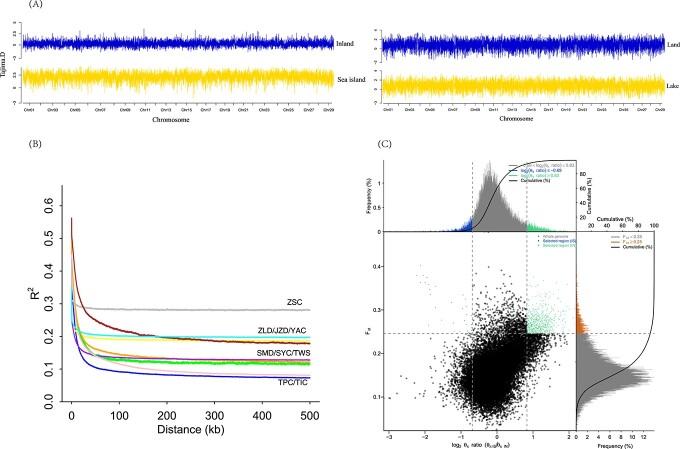
Genomic regions with strong selective signals in natural populations of the three species. (A) Tajima’s D values in oceanic island (lower left), mainland (upper left), lake island (lower right), and neighbor land (upper right) populations. (B) Decay of linkage disequilibrium (LD) in the natural populations, with one line per population. (C) Distribution of log2(*θ*π ratios) and *F_ST_* values calculated in 40-kb sliding windows with 20-kb increments between the oceanic group (TWS and ZSC) and the mainland group (TPC + YAC + SYC + JZD + ZLD). The data points in blue and green (corresponding to the top 5% of the empirical log_2_(*θ*π ratios) ratio distribution and the top 5% of the empirical *F_ST_* distribution) are genomic regions under selection.

The Tajima’s D values of oceanic island and lake island populations significantly differed from 0 (>0), indicating positive selection or a bottleneck effect in oceanic islands (Fig. 4A). Moreover, ZSC showed a higher LD among loci than other populations (P < 10^−16^, Mann–Whitney U test) (Fig. 4B), reflecting relatively higher inbreeding and genetic drift in the island population and skewed allele frequency spectra. The three mountain populations, ZLD, JZD, and YAC, showed higher LD than the other populations (except ZSC) (Fig. 4B).

Oceanic isolation showed 1006 genomic windows and lake isolation showed 910 windows, with 76 common windows. These genomic islands showed increased levels of Tajima’s D and nucleotide diversity (π) in oceanic island populations, whereas genomic islands had reduced Tajima’s D values and nucleotide diversity for lake isolation (Table S5). The values of absolute divergence *D_XY_* were positively correlated with *F_ST_* for oceanic isolation but not for lake isolation (Fig. S7). These results indicate the effect of geographic isolation on genomic divergence is related to the time length of isolation.

*D_XY_* was elevated in genomic islands compared with the rest of the genomes in the lake island comparison (P < 0.001), whereas *D_XY_* decreased in genomic islands in the oceanic island comparison (P < 0.001) (Fig. S8; Table S5). The decreased *D_XY_* values indicate that such genomic islands resulted from recurrent background selection or selective sweeps because long-term selection reduced genetic variation in the most recent common ancestor (MRCA).

To examine the genetic basis of geographic isolation associated with speciation, we identified genes undergoing selective sweeps using the top 5% of *F_ST_* values and θπ ratio cutoffs (Fig. 4C). Among a total of 780 genes under positive selection, 623 genes were identified (Table S8). A total of 11 identified genes were shared among the oceanic and lake isolation comparisons. One highly enriched GO term, “single-organism developmental process” (GO:0044767, biological process), was identified (7 genes, corrected P = 0.026) for oceanic isolation (Fig. S9), and the significantly enriched KEGG pathway “selenocompound metabolism” (2 genes, corrected P = 0.08) was associated with oceanic isolation (Fig. S10). No significantly enriched GO terms or KEGG pathways were found for lake isolation.

### GWAS analysis for the trichome trait

**Figure 5:**
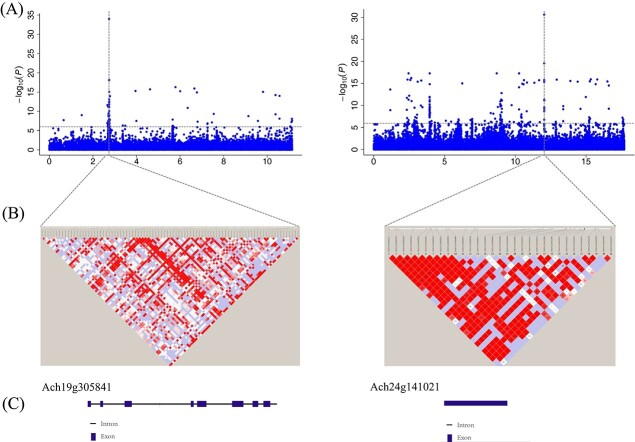
Genome wide association study (GWAS) of the trichome trait in the *Actinidia* species. (A) Manhattan plots showing significant SNPs on chromosomes 19 (left) and 24 (right). The y-axis is the negative log_10_ transformed *p*-values of SNPs from a GWAS analysis of trichomes against the genetic distance in Mb. (B) A magnified view of chromosomes 19 (left) and 24 (right), with the bottom showing the candidate range for the *Ach19g305841* and *Ach24g141021* genes. The extent of LD in this region is based on pairwise r^2^ between the estimated SNPs. (C) The structure of the *Ach19g305841* and *Ach24g141021* genes.

Trichome morphology differs among the three species. A genome-wide association study (GWAS) for the trichome trait was performed to test the local adaptation of the three species, and it revealed 2268 SNPs and 2033 genes for the trichome trait (Table S9). The selective sweep analysis for oceanic isolation and the trichome trait GWAS revealed 20 common genes (Table S10). The trichome trait was significantly associated with an interval on chromosome (Chr) 19, as well as Chr 24 regions of the reference genome (Fig. S11, Table S11). The highest associated SNPs on Chr 19 and Chr 24 corresponded to two genes, Ach19g305841 (phosphoglycerate kinase, PGK) and Ach24g141021 (DNA/RNA-binding protein KIN17), respectively (Fig. 5).

GWAS analysis for the trichome trait revealed genes involved in trichome initiation, formation, and development, such as zinc finger proteins (ZFPs), TCP proteins, bHLH and WD40 proteins, MYB transcription factors, DUF/TBL1(trichome birefringence-like) proteins, and cellulose synthase, and in ecological adaptation via biotic and abiotic stress resistance, such as members of the WRKY gene family, PERK1, auxin response factors and induced proteins, and genes in the ethylene (EIN3) and gibberellin (GA) phytohormone pathways (Table S9).

## Discussion

Our population genomic analysis suggests heterogeneous patterns of genomic divergence in the sympatric and allopatric speciation of three *Actinidia* species. The genomic islands of divergence that distinguish the three *Actinidia* species most likely derived from gene flow in the sympatric regions, lineage sorting of ancient polymorphisms, and ecological adaptation under selective sweeps. Geographic isolation plays a key role in the formation of genomic islands during speciation, which varies with the time length of divergence.

Our results indicate the common ancestor of *A. deliciosa* and *A. setosa* diverged from *A. chinensis* at ~10.1 Mya. *A. chinensis* and *A. deliciosa* interbreed in sympatric regions but maintain distinct morphologies. A previous study found that *A. deliciosa* has a family of repeat sequences not present in diploid *A. chinensis*, which suggests that it may be an allopolyploid species and that other species were involved in the origin of *A. deliciosa* [47]. In the current study, we found that *A. deliciosa* diverged from *A. chinensis*, but there was insufficient information to support its allopolyploid or autopolyploid origin. Diploid *A. chinensis* is probably itself a cryptic polyploid or rediploidized paleopolyploid because its basic chromosome number (n = 29) is high [18, 48]. Most chromosomes of *A. chinensis* have Robertsonian-like centromeric translocations [49]. Polyploidy is one of the important mechanisms of speciation, and approximately 70% of flowering plants have undergone polyploidy events during their evolutionary history [50], which increases their adaptive capability. Indeed, we found that the effective population size of *A. deliciosa* increased rapidly, which presumably was advantageous for ecological adaptation. Morphological intermediates and hybrid forms between *A. deliciosa* and *A. chinensis* have been found in nature [23], and the two species have had extensive gene flow and shared ancestral variation, even across ploidy levels [51].

Our results suggest that *A. setosa* is derived from the last common ancestor shared with *A. deliciosa* in mainland China ~2.91 Mya and formed a separate species in the Taiwan Island at ~0.92 Mya. The divergence time (~2.91 Mya) of *A. setosa* was less than the formation time (~5 Mya) of the Taiwan Island [52]. A low effective population size (Ne) and recombination rate (ρ) probably contributed to the low degree of *A. setosa* genetic diversity [53]. Liang and Ferguson (1985) proposed that *A. setosa* is a separate species [21], and Chat et al. (2004) found that *A. setosa* diverged from *A. chinensis* and *A. deliciosa* based on chloroplastic and mitochondrial DNA sequences [24]. We conclude that the formation of *A. setosa* resulted from its low effective population size under island isolation, with minimal gene flow, reduced recombination, and increased genetic drift due to a high hitchhiking effect or selective sweeps [54].

The level of divergence (*F_ST_*) between the three species was highly heterogeneous along the genome, probably owing to demographic processes and genetic drift [11]. Population contraction due to geographic isolation (*A. setosa*) or population expansion due to gene flow and introgression (*A. deliciosa*) resulted in rapid stochastic loss or fixation of alleles (haplotypes) [55]. However, absolute divergence (*D_XY_*) was elevated in genomic islands for both sympatric speciation (*A. chinensis*–*A. deliciosa*) and allopatric speciation (*A. deliciosa*–*A. setosa* and *A. chinensis*–*A. setosa*). Elevated *D_XY_* in genomic islands resulted from differential gene flow between genomic regions or from the presence of anciently diverged haplotypes [8]. In sympatric speciation under the speciation-with-gene-flow model, genetic exchange via hybridization and introgression can result in a genetic admixture, which elevates absolute and relative divergence in genomic islands [56]. In allopatric divergence without gene flow, genomic islands are generally formed by lineage sorting [8]. Divergence resulting from geographic isolation leads to lineage sorting and the retention of ancestral polymorphisms, with the accumulation of species-specific genetic changes and the sorting of ancestral variation among lineages [57]. Moreover, natural selection (selective sweeps) in the isolation of the Taiwan Island accelerated lineage sorting in accordance with the divergence-after-speciation model [10, 12], as there was no recurrent interspecific hybridization and introgression during allopatric speciation. *A. chinensis* occurs mostly at altitudes between 200 and 1200 m, whereas *A. deliciosa* is usually found at 800–1400 m and *A. setosa* at 1300–2600 m [48]. We conclude that selective sweeps decreased the genetic diversity (π) of *A. setosa* in the Taiwan Island but fixed the variation of neutral loci through a hitchhiking effect and increased genetic diversity in genomic islands, which exhibited a low recombination rate compared with the background genome. Low recombination regions are generally associated with a high hitchhiking effect or selective sweeps [54]. This suggests a key role for genomic islands in plant speciation, associated with geographic isolation that presents a barrier to gene flow and recombination and thus accelerates divergence under selective sweeps [58].

Interestingly, we found reduced *D_XY_* values in genomic islands of oceanic island populations (TWS + ZSC) compared with mainland populations, indicating ongoing background selection and recurrent selective sweeps due to linked selection in the genomic islands of isolated populations [8]. This suggests that habitat isolation resulted in strong natural selection stresses with low genetic diversity and positive high Tajima’s D in oceanic island populations. Inbreeding caused by geographic isolation leads to the fixation and deletion of alleles and subsequent loss of genetic variance [59, 60]. Island populations are often genetically isolated from the mainland, and most island populations are small, thus facilitating random genetic drift [57].

Our results showed that the genetic effects of geographic isolation varied with the time length of isolation, over 60 years for the thousand-island lake isolation, 7000–9000 years for the Zhoushan Archipelago, and ~5 Mya for the Taiwan Island [52]. Long-term oceanic isolation led to decreased genetic diversity (π) of populations in the oceanic islands and diverged (*F_ST_*) from mainland populations, whereas short-term lake isolation did not. A new species (*A. setosa*) was formed on the Taiwan Island, and a tetraploid population of *A. chinensis* was generated in the Zhoushan Archipelago. Geographic isolation is one of the main reasons for population differentiation and eventual speciation, with limited gene flow among populations [15, 61–63]. As an example, Mes et al. [64] found that ca. 85% of the species in genus *Aeonium* resulted from geographic isolation. We suppose that the Zhoushan Archipelago population derived from the SMD population because the Zhoushan Archipelago and the Siming mountains belonged to the Tiantai Mountain Chain before formation of the Zhoushan Archipelago as a result of rising sea levels. The high LD of the ZSC population indicates that individuals experienced positive selection or selective sweeps in the isolated Zhoushan Archipelago, which may explain the maintenance of high divergence between species despite extensive gene flow [65]. In addition, genetic differentiation was not significantly related to geographic distance in four diploid *A. chinensis* populations. The dioecy characteristics of *A. chinensis* can decrease its inbreeding rate and counteract the negative effects of geographic isolation [66]. Gene flow can weaken the differentiation effects of geographic isolation [67–70]. The differential effects of geographic isolation on genetic diversity resulted from the time length of isolation and local ecological adaptation, which probably cause a time-delayed extinction debt [71].

Genes in genomic islands have been associated with local adaptation, organ development, and reproductive isolation, and they are likely to experience morphological and ecological divergence between lineages due to geographic isolation [65]. For example, MS1 (PHD finger protein MALE STERILITY 1), a nuclear transcriptional activator, participates in tapetal development and pollen wall biosynthesis. FPA regulates flowering time via an independent day-length pathway, and the KANADI gene family regulates the development of the carpel and the outer integument of the ovule [72]. In addition, a large proportion of genes were transcription factors, e.g. WRKY transcription factors involved in the response to pathogens [73]. In the current study, some genes associated with isolation divergence were related to trichome initiation, such as members of the TBL (Trichome Birefringence-like) gene family that participate in the synthesis and deposition of secondary cell wall cellulose, and C2H2-like zinc finger protein (ZFP) genes that regulate trichome initiation, branching, and shoot maturation [74]. MYB, bHLH69, and WD3 genes are associated with trichome differentiation, and DUF/TBL genes contribute to secondary wall cellulose synthesis [75]. Similarly, a WD40-bHLH-MYB regulatory mechanism plays a key role in controlling trichome formation and development [76–78]. In addition, proteins in the plant-specific transcription factor families TCP, YUC, and NAC modulate cell proliferation and differentiation, regulate development, and may be involved in trichome cell differentiation and leaf development and morphology [11, 79]. Our GWAS analysis also identified PGK1 and KIN17 genes, which are significantly associated with the trichome trait. KIN17 participates in the control of plant growth and development in response to oxidative stress and UV radiation [80]. PGK is important for glycolysis (EMP pathway) and the tricarboxylic acid cycle, and PGK1 is associated with cold responses and cell wall metabolism [81, 82]. These results suggest that the trichome trait is involved in stress responses, e.g. enhancing cold tolerance by promoting low levels of anaerobic respiration and high levels of aerobic respiration to reduce sugar consumption, which may explain the high elevation distribution of *A. deliciosa* plants with trichomes.

In conclusion, our study provides insights into the phylogenetic relationships, speciation histories, and corresponding genomic divergence of three *Actinidia* species and sheds light on the mechanisms by which geographic isolation influences speciation through its effects on population size and gene flow among populations. Regardless of the large-scale or small-scale biogeographic patterns, the three species showed post-speciation adaptation to the local environment, with the trichome trait phenotype and related genes. The results distinguished the mechanisms of genomic divergence for sympatric and allopatric speciation processes of the three species, involving gene flow, ancestral polymorphisms, and selective sweeps. Our study suggests that geographic isolation represents an important factor in speciation with selective sweeps or background selection, and it highlights the significance of the time length of divergence.

## Acknowledgements

We thank Liu F., Han W., and local farmers for their assistance in field sampling and Prof. Cheng F. and Dr. Yang Y. for their assistance in the analysis of recombination rate. This work was financially supported by the Biodiversity Survey, Observation and Assessment of the Ministry of Ecology and Environment, China (2019HJ2096001006) and the General Research Project of CRAES, China (No. 2016YSKY-08).

## Author contributions

Y.L. designed the study. Y.L., W.Y., B.W., and J.L. performed the experiment. Y.L. wrote the manuscript. All authors read and approved the manuscript.

## Data availability

Data will be uploaded after acceptance.

## Conflict of interest

The authors declare that they have no conflict of interest.

## Supplementary data

Supplementary data is available at *Horticulture Research Journal* online.

## Supplementary Material

Web_Material_uhac054
