# Supplementary material for: Patterns of genomic divergence in sympatric and allopatric speciation of three Mihoutao (Actinidia) species
Source: Hortic Res. 2022 Mar 3;9:uhac054. doi: 10.1093/hr/uhac054 (PMC9113235; doi:10.1093/hr/uhac054)
Supplement: Web_Material_uhac054 [file web_material_uhac054.zip › Sfigs.pdf]

A

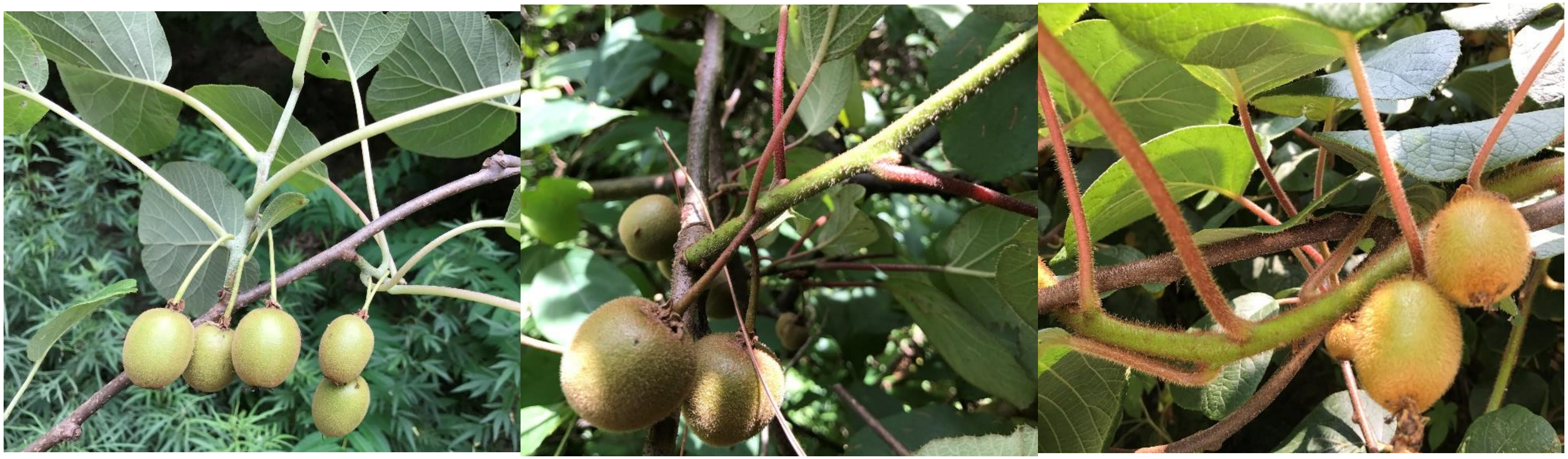

B

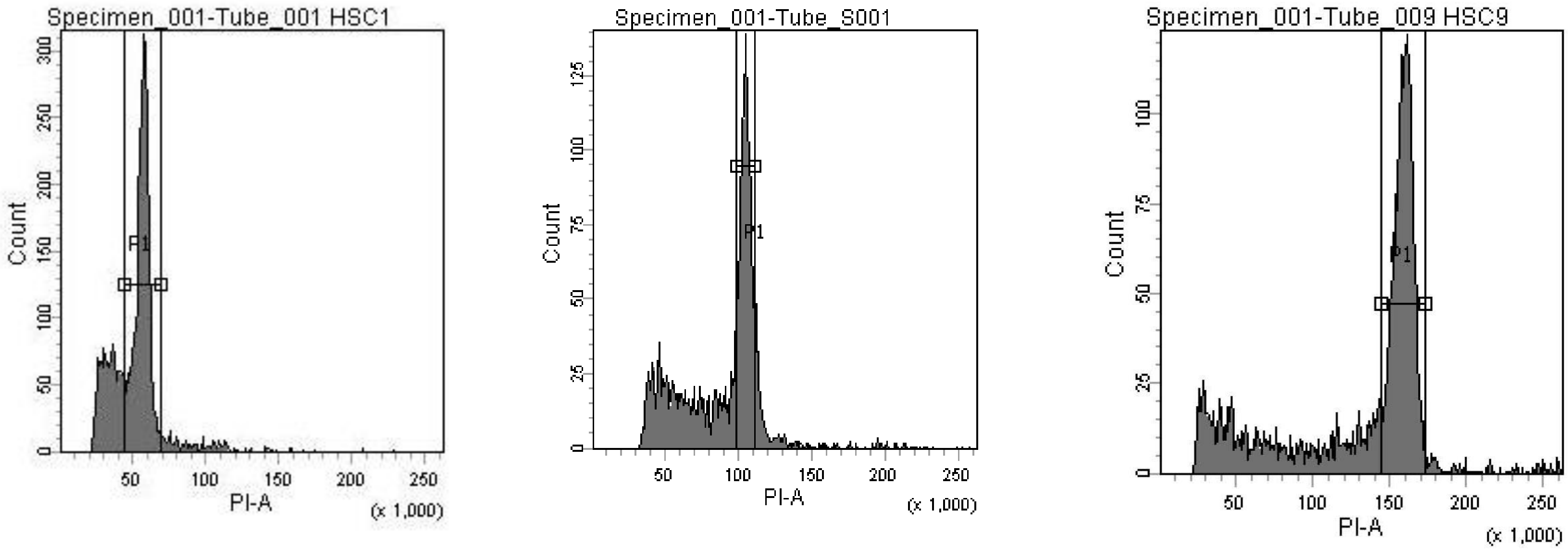

Supplementary Fig. S1. Photos of the morphology for *Actinidia chinensis*, and *A. deliciosa*. A, the fruit and leaf of *A. chinensis* (left), tetraploid *A. deliciosa* (middle) and hexaploidy *A. deliciosa* (right); **B** The flow cytometry result of diploid, tetraploid and hexaploid.

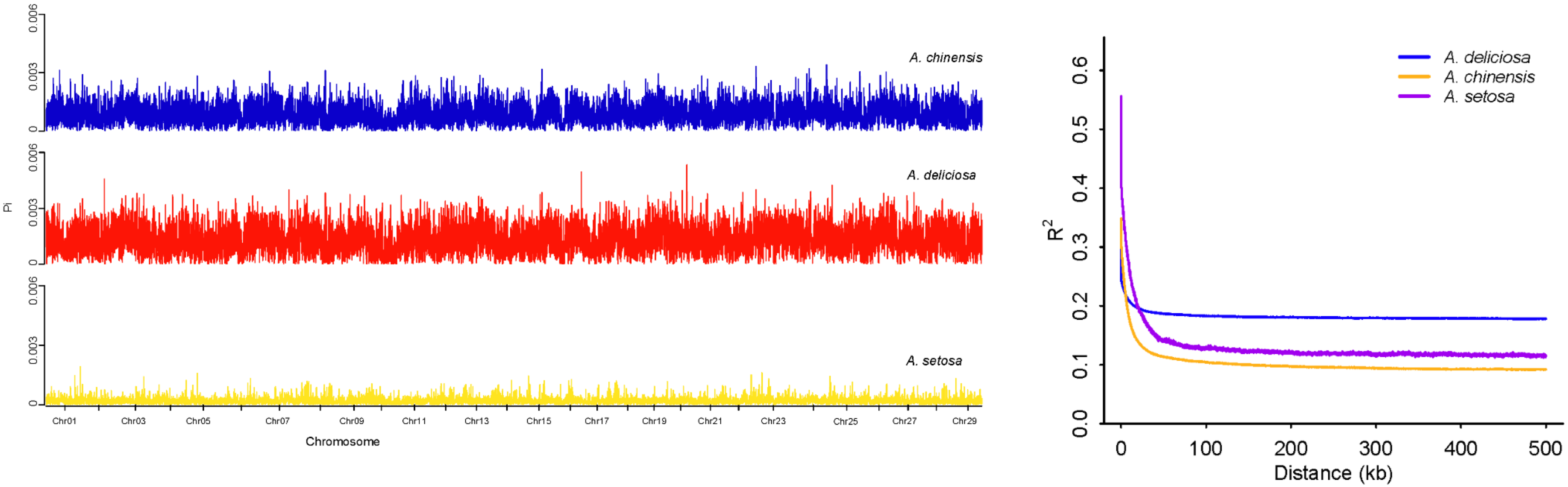

Supplementary Fig. S2. Genetic diversity (Pi) and LD of the three species, *A. chinensis*, *A. deliciosa* and *A. setosa*.

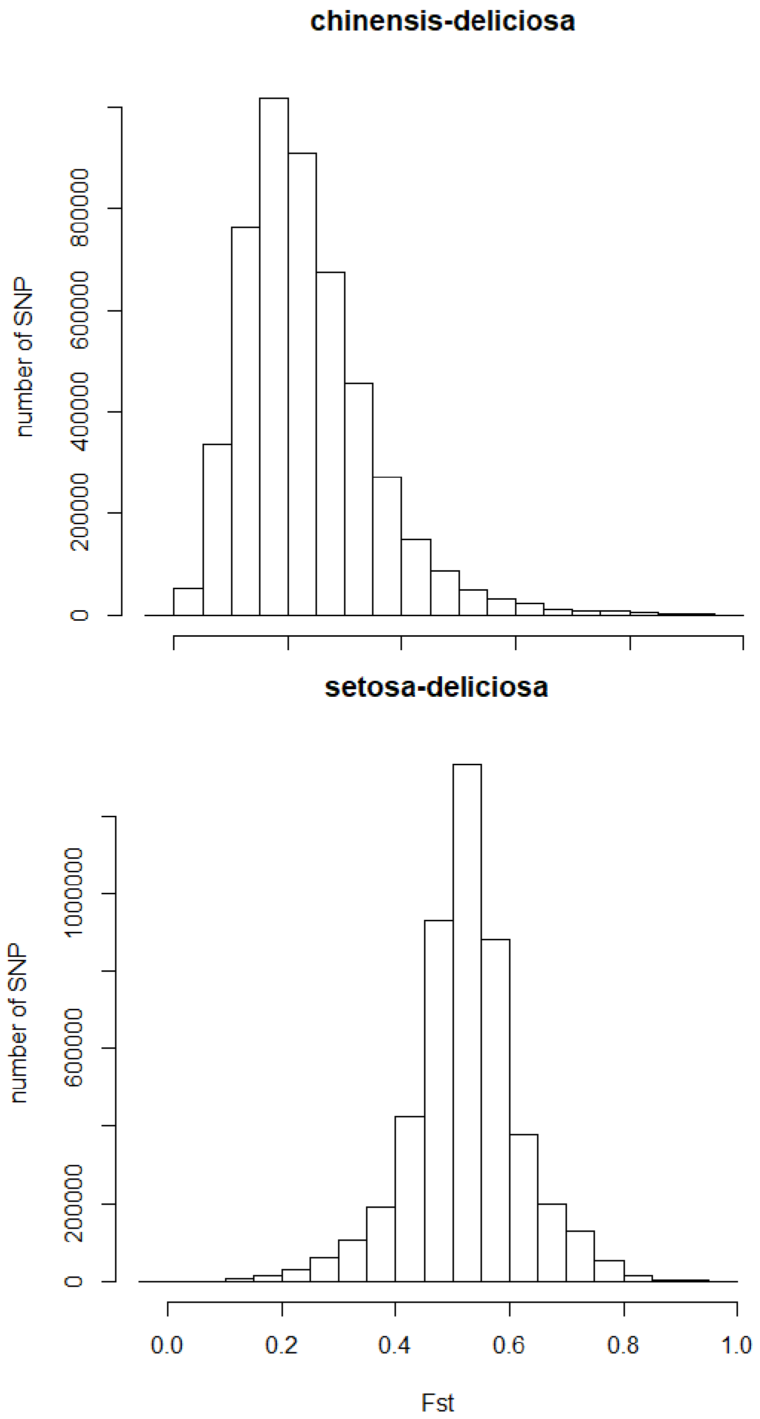

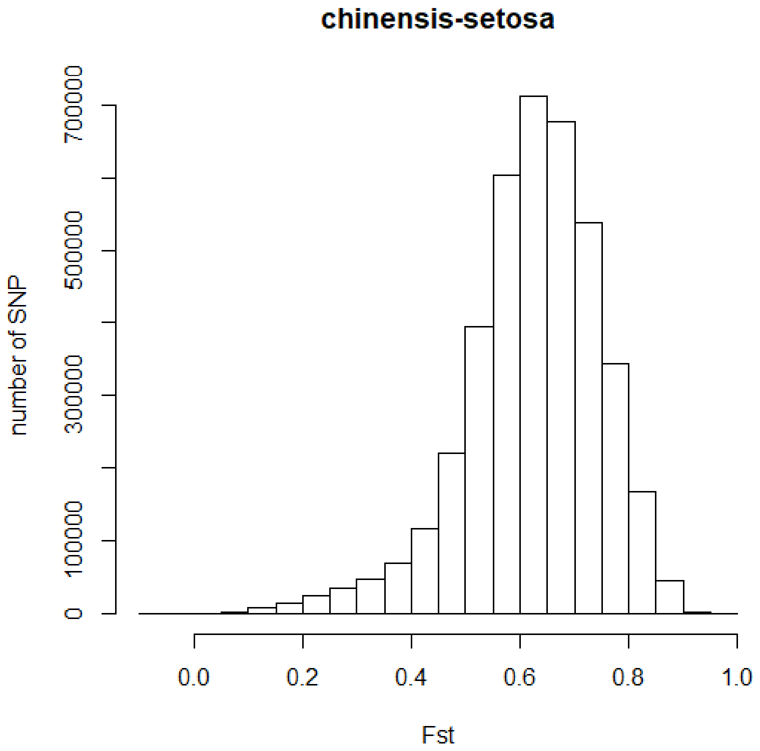

Supplementary Fig. S3. Fst of three species comparisons.

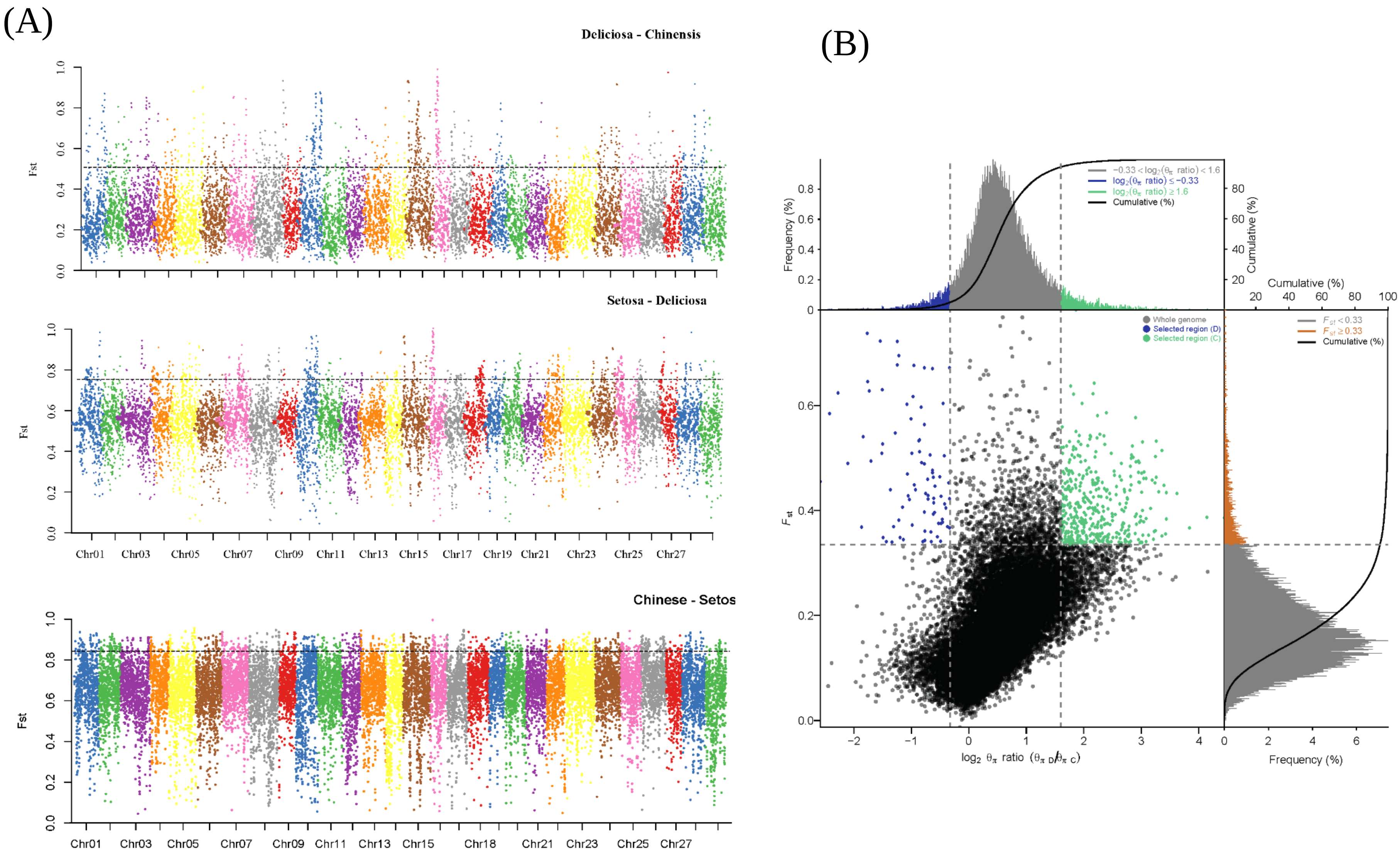

Supplementary Fig. S4. (A) Fst of the three species, *A. chinensis*, *A. deliciosa* and *A. setosa*. Points above lines indicate genomic islands (Z-*FST*>2). (B) Distribution of log2($\theta\pi$ ratios) and *F*ST values calculated in 40-kb sliding windows with 20-kb increments between *A. chinensis* and *A. deliciosa*.

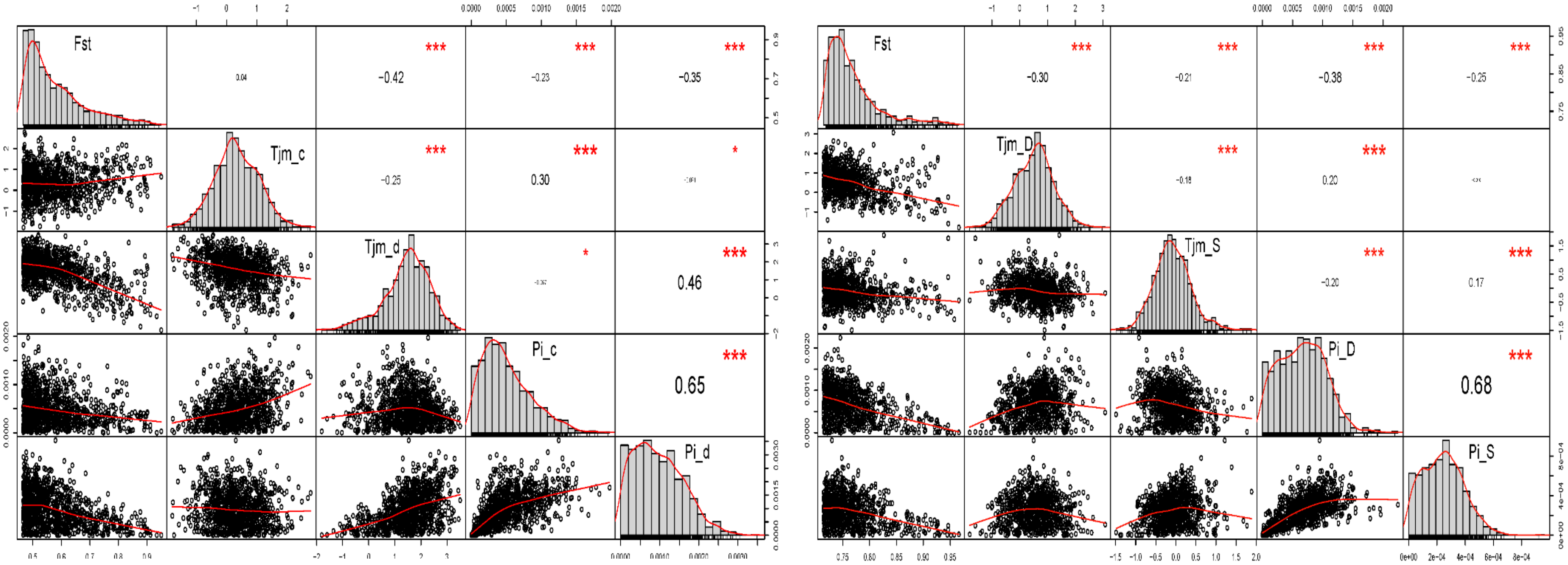

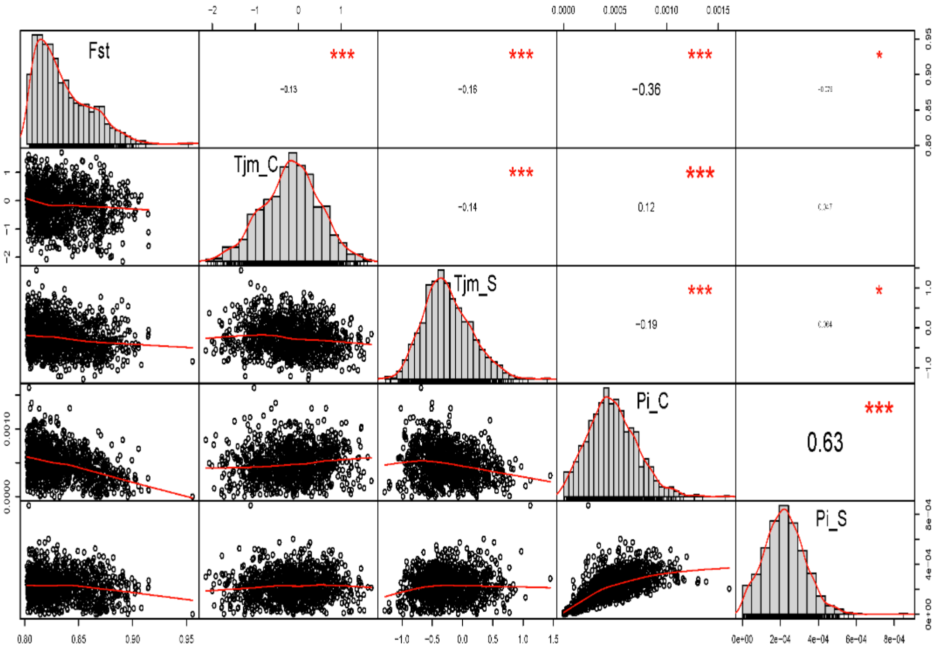

Supplementary Fig. S5. Correlation between Fst, Tajima’s D, and nuclear diversity of three species comparisons.

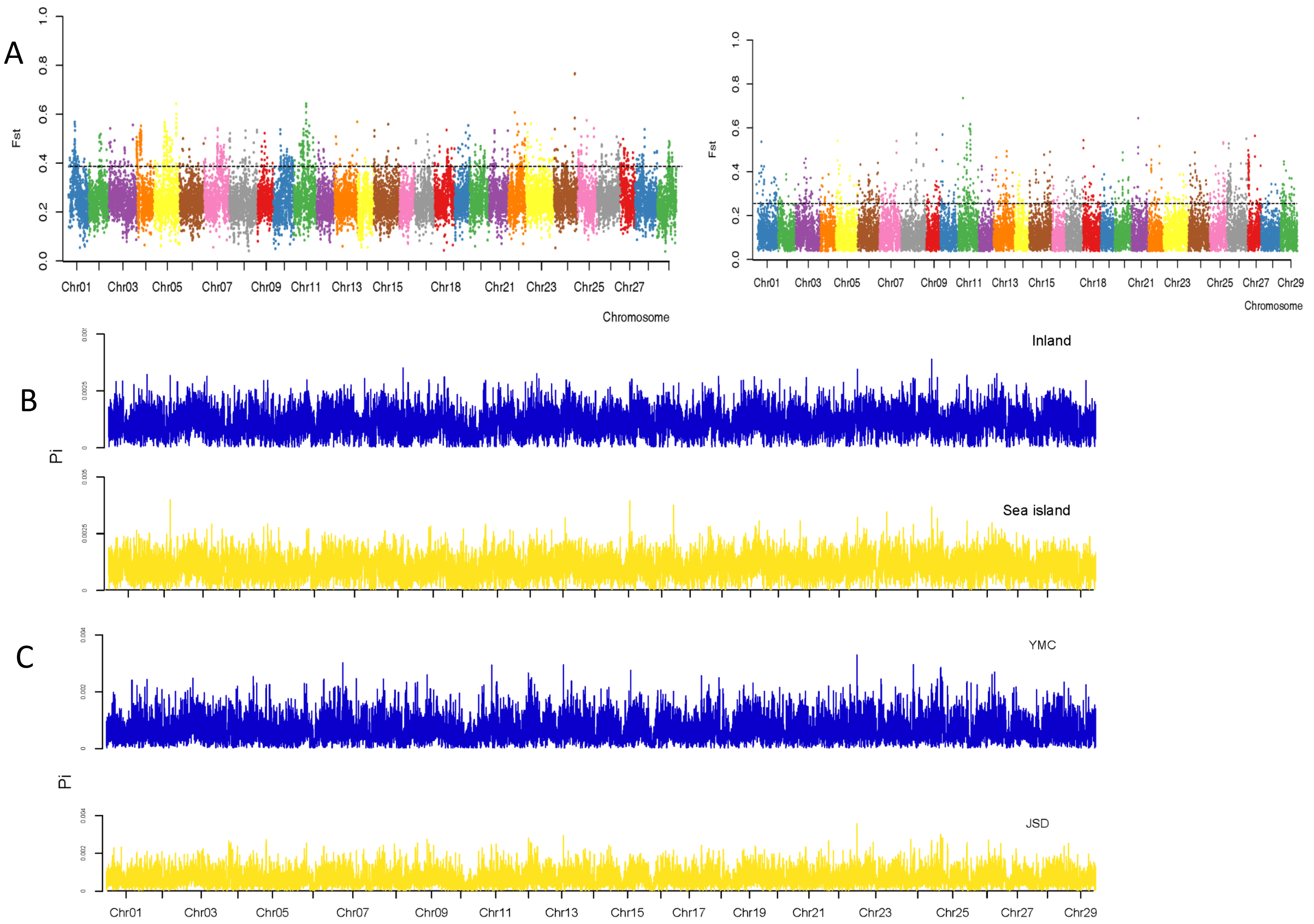

Supplementary Fig S6. Relative divergence (Fst) and nuclear diversity (pi) of oceanic and lake isolation.

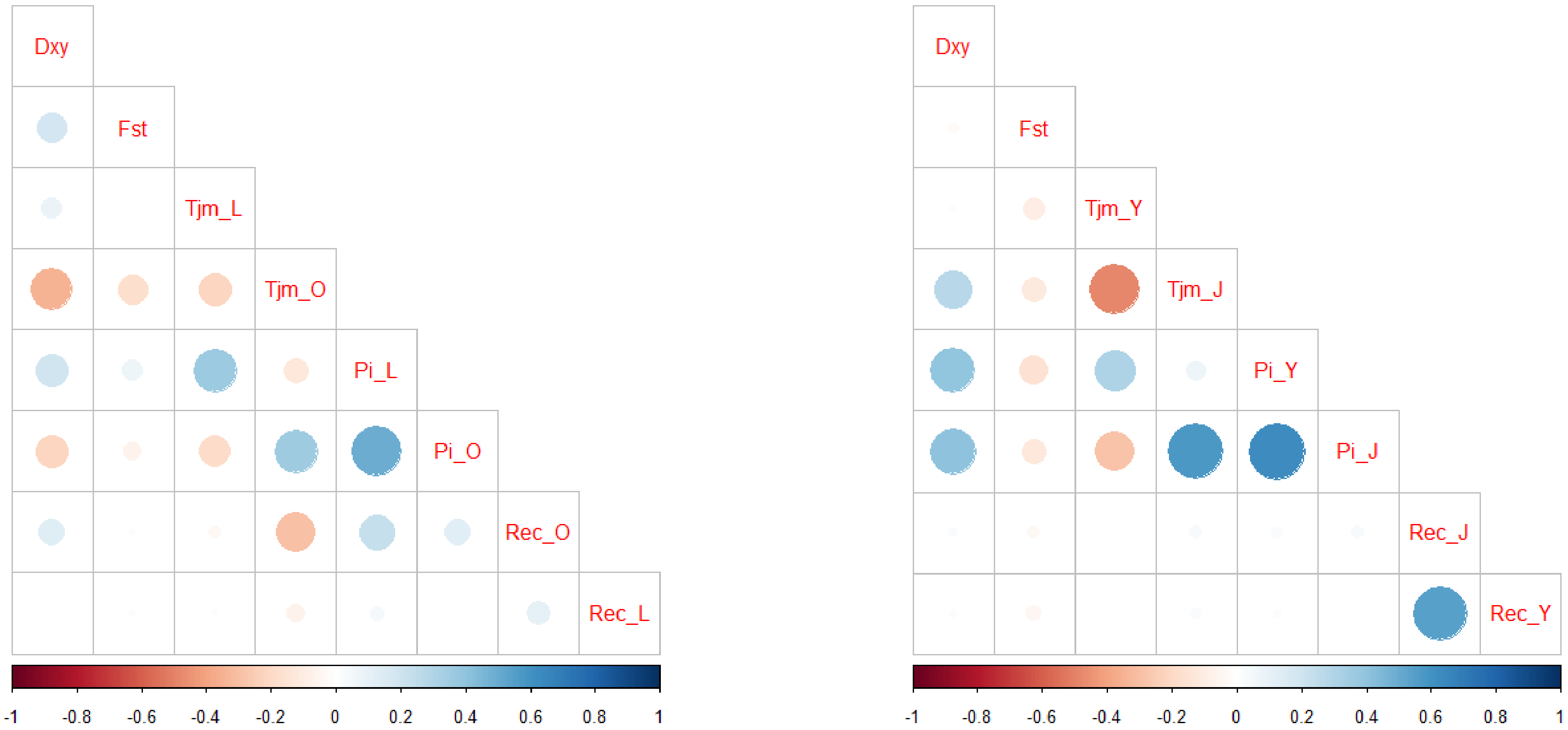

Supplementary Fig. S7. Correlation of genetic parameters for oceanic isolation (left) and lake isolation (right).

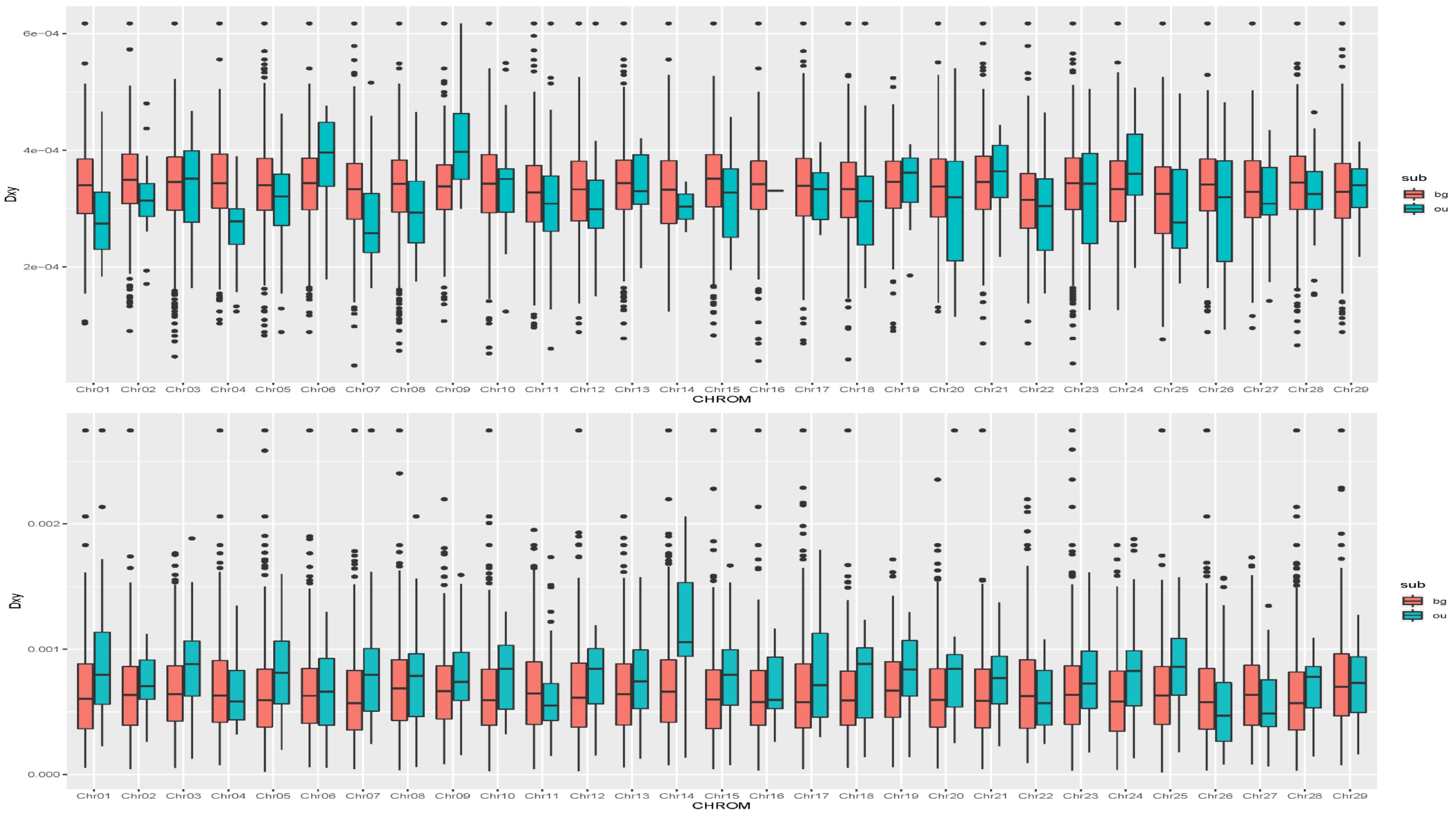

Supplementary Fig. S8. Decreased Dxy in genomic island compared to background genome for oceanic isolation (A, above) and increased Dxy for lake isolation (B, bottom). Symbol “ou” represents genomic islands and “bg” represents background genome.

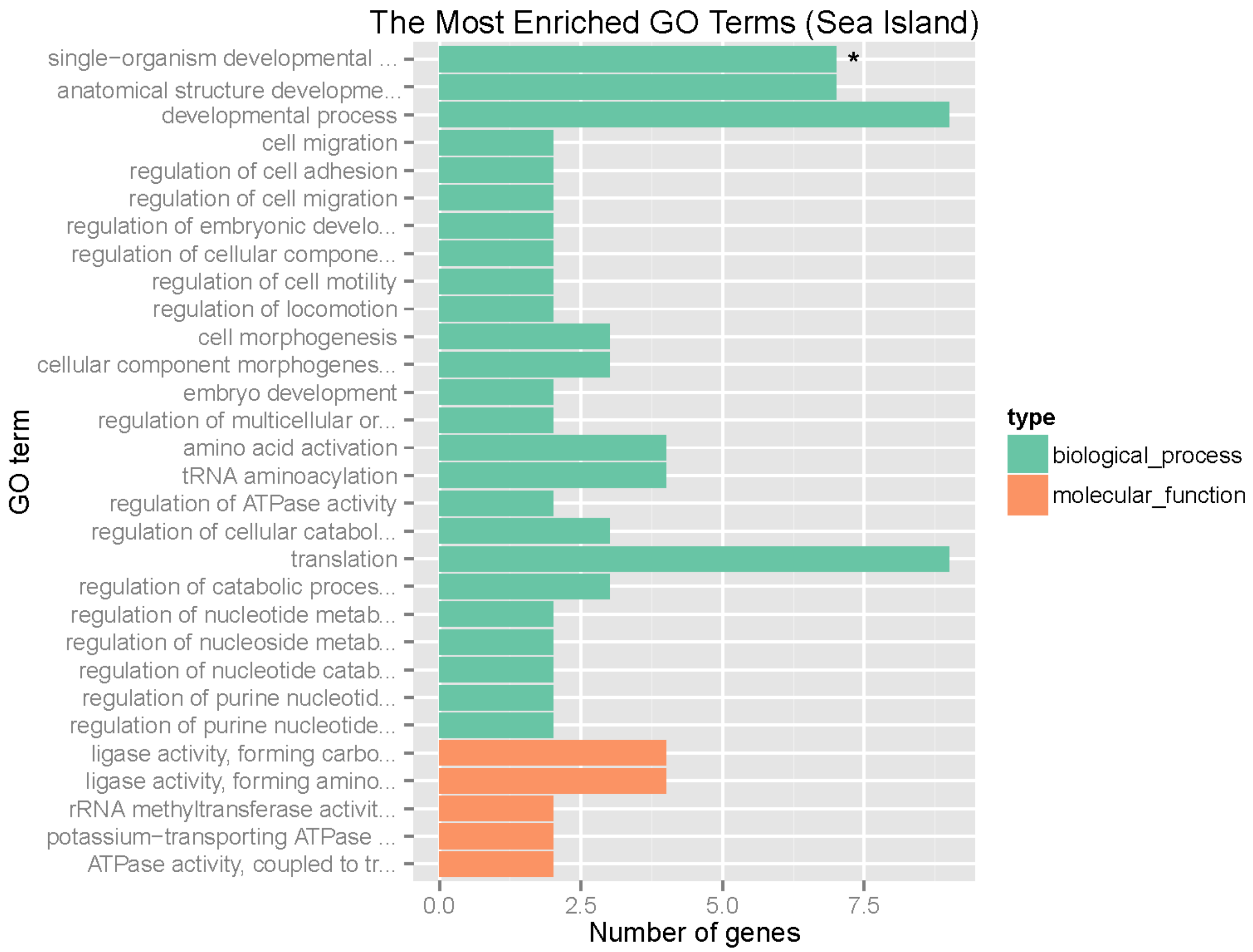

Supplementary Fig. S9. GO analysis of the expansion genes in oceanic island populations.

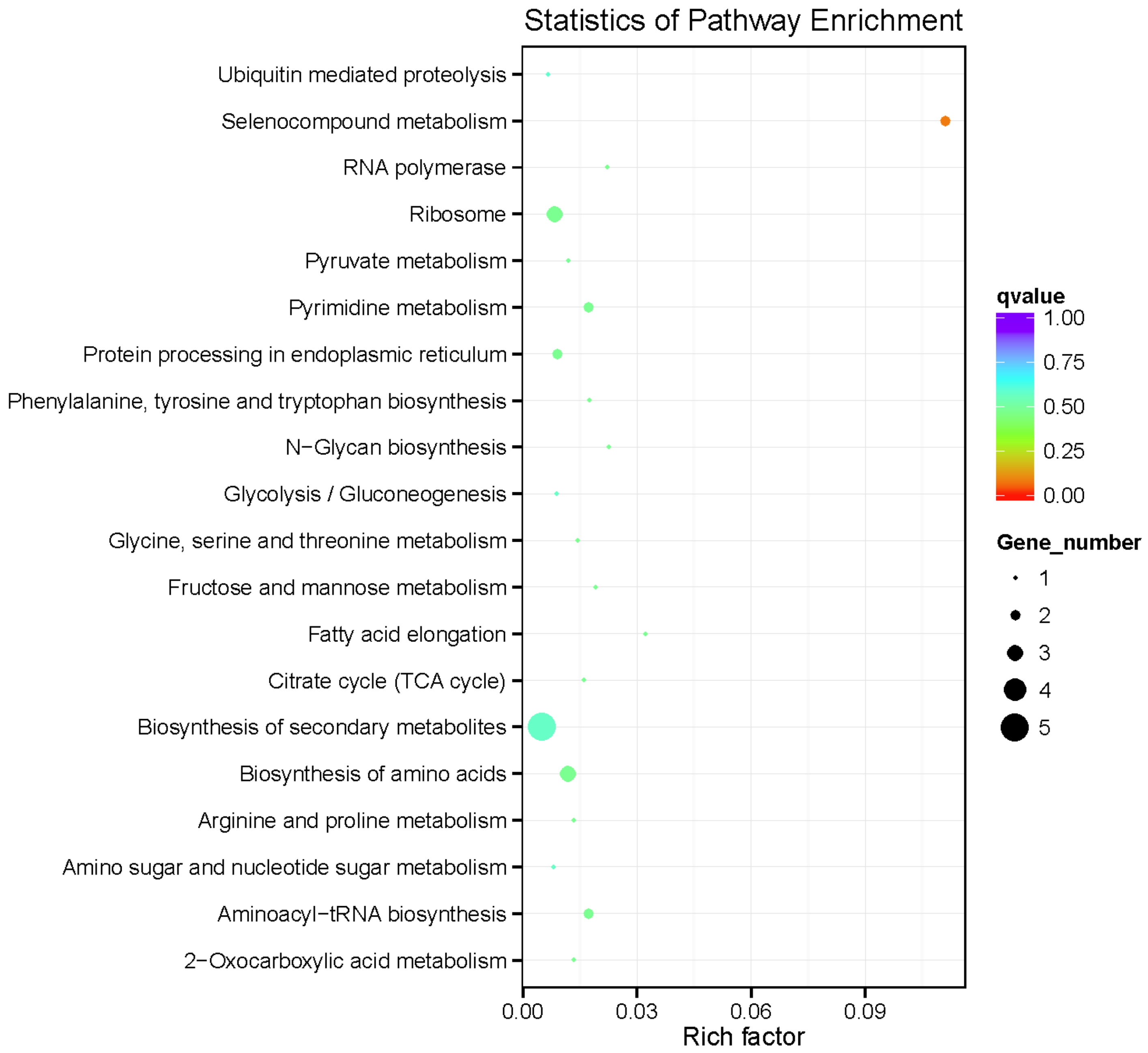

Supplementary Fig. S10. KEGG analysis of the expansion genes in oceanic island populations.

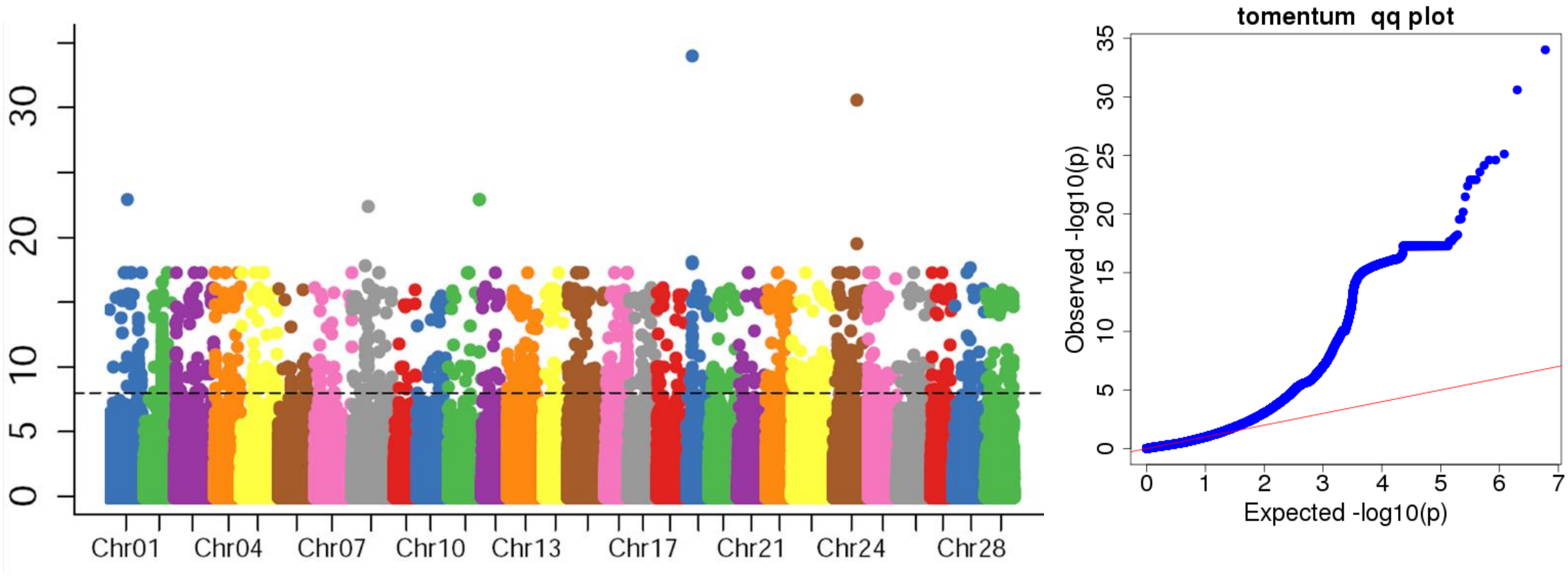

Supplementary Fig. S11. Manhattan and quantile-quantile (QQ) plots of genome-wide association studies (GWAS) of trichome traits in the three Actinidia species. Each dot represents an SNP. The horizontal dashed red lines indicate the Bonferroni-corrected significance thresholds.
