# Supplementary material for: Patterns of genomic divergence in sympatric and allopatric speciation of three Mihoutao (Actinidia) species
Source: Hortic Res. 2022 Mar 3;9:uhac054. doi: 10.1093/hr/uhac054 (PMC9113235; doi:10.1093/hr/uhac054)
Supplement: Web_Material_uhac054 [file web_material_uhac054.zip › Supplementary tables.pdf]

Supplementary files

Tables

Table S1. Sample information for Actinidia chinensis in nine populations from seven provinces, China

Provinces	Populations	Longitude	Latitude	Altitude (m)	No. of samples	Species	Ploidy
Zhejiang (TIC)	Lake island (JSC)	118.6379	29.1961	102	13	*A. chinensis*	2
	Neighbor land (YMC)	118.6379	29.1961	116	14	*A. chinensis*	2
Henan	Taipingzhen (TPC)	111.7094	33.6336	1143	15	*A. chinensis*	2
Hunan	Shangyancun (SYC)	110.9263	30.0398	402	13	*A. chinensis*	2
Guangxi	Yongancun (YAC)	110.3239	25.8001	480	12	*A. chinensis*	2
Zhejiang	Zhoushan (ZSC)	122.1157	30.0859	108	13	*A. chinensis*	4
	Simingshan (SMD)	121.0956	29.7491	950	15	*A. deliciosa*	4
Hubei	Junzipao (JZD)	110.7265	31.4652	1460	15	*A. deliciosa*	6
Sichuan	Zhonglingcun (ZLD)	103.0049	30.374	1380	14	*A. deliciosa*	6
Taiwan (TWS)	Taipingshan	121.5618	24.5003	1700	4	*A. setosa*	2
	Lishan	121.2571	24.2201	1650	7		
	Qilanshan	121.6515	24.8877	1650	3		

Table S2. Overview of sequencing statistics.

Populations	Raw Base(bp)	Clean Base(bp)	Effective Rate(%)	Error Rate(%)	Q20(%)	Q30(%)	GC Content(%)	Clean reads	Mapped reads	Mapping rate	Average depth
JZD	4,790,442,600	4,781,706,600	99.82	0.03	96.53	90.85	35.85	31878044	29195857	91.59%	5.34
JZD	6,543,868,200	6,534,839,400	99.86	0.03	97.25	92.31	36.95	43565596	38205338	87.70%	7.37
JZD	6,471,848,700	6,456,803,100	99.77	0.03	96.4	90.81	39.25	43045354	30668969	71.25%	5.8
JZD	6,133,847,100	6,121,611,600	99.8	0.03	95.81	89.7	36.36	40810744	36301528	88.95%	7.09
JZD	7,644,423,300	7,633,984,500	99.86	0.03	97.1	91.92	36.65	50893230	45892788	90.17%	8.98
JZD	8,066,871,900	8,054,031,600	99.84	0.03	96.89	91.46	36.5	53693544	48607278	90.53%	9.45
JZD	7,587,195,900	7,577,079,600	99.87	0.03	96.81	91.31	36.25	50513864	45950925	90.97%	9
JZD	6,703,682,400	6,692,182,800	99.83	0.03	97.25	92.29	38.03	44614552	38278690	85.80%	7.46
JZD	6,209,548,500	6,199,224,900	99.83	0.03	95.75	89.68	36.42	41328166	36421996	88.13%	7.06
JZD	6,705,044,700	6,684,937,800	99.7	0.03	96.39	90.87	36.23	44566252	40200513	90.20%	8.04
JZD	7,132,693,200	7,123,852,200	99.88	0.03	97.11	91.95	36.9	47492348	42369462	89.21%	8.18
JZD	6,945,306,600	6,932,311,200	99.81	0.03	97.2	92.19	36.77	46215408	41575063	89.96%	8.05
JZD	6,872,135,100	6,858,975,300	99.81	0.03	97.43	92.66	36.91	45726502	40529685	88.63%	7.98
JZD	6,718,248,300	6,709,904,100	99.88	0.03	96.97	91.63	37.97	44732694	38016772	84.99%	7.47
JZD	6,577,629,600	6,558,300,900	99.71	0.03	96.5	90.8	40.71	43722006	29816693	68.20%	5.59
SMD	6,604,028,700	6,579,792,900	99.63	0.03	96.43	90.94	38.55	43865286	37839329	86.26%	7.58
SMD	5,293,642,200	5,278,853,700	99.72	0.03	96.47	90.8	36.76	35192358	31787687	90.33%	5.99
SMD	5,557,212,600	5,536,721,100	99.63	0.03	96.18	90.19	37.42	36911474	31073259	84.18%	5.93
SMD	5,273,412,300	5,244,187,500	99.45	0.03	96.53	90.9	37.75	34961250	28772502	82.30%	5.22
SMD	6,496,739,700	6,464,330,700	99.5	0.03	96.71	91.7	39.85	43095538	35027884	81.28%	6.81
SMD	8,654,837,700	8,604,248,100	99.42	0.03	97.09	91.97	37.64	52634238	47496820	90.24%	10.17
SMD	9,578,879,700	9,557,520,600	99.78	0.03	96.76	91.25	40.83	63716804	49177575	77.18%	9.79
SMD	7,736,422,200	7,704,576,600	99.59	0.03	96.84	91.41	36.57	47128184	44151630	93.68%	9.29

SMD	6,218,897,100	6,205,245,900	99.78	0.03	96.33	90.89	36.62	41368306	36594462	88.46%	7.41
SMD	6,666,584,700	6,652,598,400	99.79	0.03	95.37	88.28	41.94	44350656	31789296	71.68%	6.2
SMD	6,156,611,400	6,148,109,700	99.86	0.03	97.11	91.91	36.67	40987398	38742610	94.52%	7.78
SMD	6,304,656,600	6,289,927,800	99.77	0.03	96.4	90.59	36.75	41932852	38810628	92.55%	7.9
SMD	10,290,714,000	10,257,447,600	99.68	0.03	97.13	92.06	40.76	68382984	52970764	77.46%	10.42
SMD	7,815,100,200	7,788,953,400	99.67	0.03	97.39	92.6	40.87	51926356	38938178	74.99%	7.78
SMD	5,996,893,800	5,971,424,400	99.58	0.03	96.72	91.11	37.23	36525554	32425543	88.77%	6.69
SYC	6,012,630,000	5,992,204,500	99.66	0.04	95.38	88.46	37.17	39948030	35682925	89.32%	7.38
SYC	6,814,795,500	6,797,535,300	99.75	0.03	96.57	90.94	41.94	45316902	35134443	77.53%	7.08
SYC	7,216,891,200	7,195,079,700	99.7	0.03	96.58	91.22	39.55	47967198	40161867	83.73%	8.27
SYC	7,728,358,500	7,711,658,700	99.78	0.03	97.17	92.06	42.02	51411058	39433044	76.70%	8.03
SYC	7,085,809,200	7,067,929,800	99.75	0.03	96.14	90.24	42.53	47119532	34337382	72.87%	7.39
SYC	6,613,449,600	6,602,870,100	99.84	0.03	95.9	89.5	38.45	44019134	36337153	82.55%	7.76
SYC	7,267,899,600	7,206,209,400	99.15	0.03	97.02	91.94	37.06	48041396	44032356	91.66%	8.43
SYC	5,678,613,300	5,654,091,000	99.57	0.03	97.61	93	41.33	37693940	29581028	78.48%	6.11
SYC	6,552,285,600	6,534,471,000	99.73	0.03	95.89	89.99	44.81	43563140	27280158	62.62%	5.24
SYC	6,990,675,300	6,974,070,900	99.76	0.03	96.63	91.33	39.34	46493806	40086506	86.22%	8.45
SYC	6,834,701,400	6,815,499,000	99.72	0.03	96.12	90.36	42.42	45436660	32512048	71.55%	6.63
SYC	7,287,752,100	7,268,664,900	99.74	0.03	96.72	91.52	38.68	48457766	41487608	85.62%	8.59
SYC	6,505,697,100	6,487,956,300	99.73	0.03	96.72	91.19	39.16	43253042	36610152	84.64%	7.54
JSC	4,143,660,300	4,113,213,000	99.27	0.03	96.62	91.03	35.92	27421420	26717690	97.43%	5.05
JSC	6,755,895,000	6,734,875,200	99.69	0.03	96.51	90.59	36.87	44899168	42093615	93.75%	8.48
JSC	8,983,985,700	8,970,255,300	99.85	0.03	95.89	89.41	37.05	59801702	56147544	93.89%	12.17
JSC	7,572,213,000	7,559,944,500	99.84	0.03	97.35	92.44	42.41	50399630	37918901	75.24%	7.6
JSC	12,234,578,100	12,191,866,200	99.65	0.03	97.43	92.86	42.13	81279108	61572958	75.75%	13.03
JSC	7,062,989,400	7,033,326,900	99.58	0.03	96.64	91	41.9	46888846	34984336	74.61%	6.7

JSC	7,022,808,900	7,005,228,300	99.75	0.03	96.53	90.83	41.47	46701522	37095107	79.43%	7.74
JSC	7,095,638,400	7,084,353,900	99.84	0.03	95.5	88.66	37.18	47229026	42712791	90.44%	9.2
JSC	6,894,449,400	6,873,984,300	99.7	0.03	96.46	90.97	40.09	45826562	38729705	84.51%	7.99
JSC	6,633,304,500	6,618,195,000	99.77	0.03	96.42	90.56	37.89	44121300	39193546	88.83%	8.26
JSC	5,585,944,200	5,545,010,100	99.27	0.03	96.1	90.07	38.71	36966734	31295927	84.66%	6.03
JSC	6,561,481,500	6,543,844,200	99.73	0.03	98.17	94.47	38.2	43625628	39307377	90.10%	7.64
JSC	6,773,388,600	6,758,766,300	99.78	0.03	96.06	89.78	36.58	45058442	42747002	94.87%	8.2
YMC	7,071,997,200	7,033,602,900	99.46	0.03	96.41	90.88	38.06	46890686	39669219	84.60%	8.13
YMC	7,079,192,400	7,057,828,500	99.7	0.03	96.2	90.55	42.52	47052190	34258610	72.81%	6.92
YMC	6,555,527,100	6,483,734,700	98.9	0.03	96.82	91.35	37.61	43224898	40328918	93.30%	8.44
YMC	6,648,902,400	6,582,805,800	99.01	0.03	96.75	91.38	36.46	43885372	41559365	94.70%	8
YMC	7,048,458,900	7,029,131,100	99.73	0.03	96.83	91.44	39.82	46860874	35470043	75.69%	7.3
YMC	8,536,623,300	8,520,488,100	99.81	0.03	97.31	92.34	38.89	56803254	47988186	84.48%	9.97
YMC	5,103,353,700	4,764,552,300	93.36	0.03	97.78	93.26	38.11	31763682	27487006	86.54%	5.5
YMC	7,582,244,100	7,507,916,700	99.02	0.03	96.83	91.5	35.98	50052778	48084515	96.07%	9.23
YMC	8,764,657,200	8,754,919,800	99.89	0.03	97.03	91.77	37.26	58366132	54379874	93.17%	11.22
YMC	5,877,300,300	5,843,862,600	99.43	0.03	96.55	90.87	35.81	38959084	37288537	95.71%	7.4
YMC	6,360,125,100	6,315,024,900	99.29	0.03	95.91	89.78	36.29	42100166	40501999	96.20%	8.79
YMC	6,159,458,100	6,122,599,500	99.4	0.03	96.62	91.1	38.3	40817330	35112833	86.02%	7.12
YMC	7,352,139,000	7,303,715,700	99.34	0.03	96.37	90.78	37.03	48691438	45097118	92.62%	9.36
YMC	7,175,798,700	7,111,041,000	99.1	0.03	96.8	91.36	38.84	47406940	40901456	86.28%	8.39
TPC	7,134,606,300	7,116,200,100	99.74	0.03	97.23	92.12	36.9	47441334	44730740	94.29%	9.07
TPC	8,552,205,900	8,512,804,500	99.54	0.03	95.45	88.55	36.31	56752030	52262672	92.09%	10.98
TPC	10,330,057,200	10,316,501,400	99.87	0.03	97.23	92.12	40.58	68776676	57056015	82.96%	11.81
TPC	6,473,594,700	6,463,125,900	99.84	0.03	96.46	90.7	36.61	43087506	41042694	95.25%	7.84
TPC	6,772,146,300	6,670,367,100	98.5	0.03	96.94	91.74	38.54	44469114	37432068	84.18%	7.43

TPC	6,688,413,600	6,645,183,900	99.35	0.03	96.83	91.5	36.42	44301226	42145140	95.13%	8
TPC	6,730,748,100	6,700,871,400	99.56	0.03	95.97	89.53	37.3	44672476	40904058	91.56%	8.65
TPC	7,061,782,500	7,048,533,300	99.81	0.03	96.34	90.76	42.16	46990222	35400719	75.34%	7.26
TPC	5,558,409,300	5,548,607,700	99.82	0.03	95.96	89.72	36.05	36990718	35831639	96.87%	6.83
TPC	6,583,368,000	6,574,413,300	99.86	0.03	96.54	91.09	37.69	43829422	39351826	89.78%	8.14
TPC	5,754,098,100	5,742,287,400	99.79	0.03	96.8	91.41	35.87	38281916	37638084	98.32%	7.31
TPC	6,601,927,500	6,593,688,600	99.88	0.03	96.7	91.21	35.92	43957924	43074312	97.99%	8.39
TPC	8,688,579,600	8,665,606,800	99.74	0.03	95.86	89.31	36.67	57770712	54004392	93.48%	11.44
TPC	7,190,939,400	7,179,682,500	99.84	0.03	96.07	90.22	36.33	47864550	45896473	95.89%	9.46
TPC	5,821,227,900	5,776,332,000	99.23	0.03	96.52	90.86	37.07	38508880	34150386	88.68%	6.66
TWS	6,619,925,100	6,580,715,400	99.41	0.03	96.53	90.94	44.96	43871436	26630324	60.70%	5.41
TWS	4,910,543,400	4,888,995,000	99.56	0.03	96.43	90.69	39.77	32593300	25635506	78.65%	4.98
TWS	7,490,093,400	7,451,079,000	99.48	0.03	96.65	91.23	47.3	34116290	24938739	73.10%	4.86
TWS	6,285,843,000	6,267,278,100	99.7	0.03	96.36	90.95	38.02	41781854	37110348	88.82%	7.98
TWS	6,401,690,400	6,389,490,900	99.81	0.04	94.22	86.74	37.42	42596606	37594836	88.26%	8.02
TWS	6,138,307,800	6,120,881,700	99.72	0.03	96.66	91.5	40.3	40805878	31674867	77.62%	6.86
TWS	4,397,876,100	4,386,369,600	99.74	0.03	96.69	91.28	39.66	29242464	22814515	78.02%	4.28
TWS	6,708,194,100	6,688,923,000	99.71	0.03	96.72	91.25	37.73	44592820	42642565	95.63%	9.38
TWS	6,458,029,500	6,445,693,200	99.81	0.03	96.46	91.17	40.7	42971288	32644854	75.97%	7.04
TWS	6,722,536,500	6,706,690,800	99.76	0.03	96.13	90.35	37.09	44711272	42223460	94.44%	9
TWS	4,788,531,300	4,767,370,500	99.56	0.03	96.6	91.06	39.06	31782470	27594934	86.82%	5.41
TWS	5,594,433,600	5,581,690,500	99.77	0.03	96.45	90.72	36.88	37211270	34582442	92.94%	7.02
TWS	7,462,497,600	7,447,359,300	99.8	0.03	96.53	90.86	36.72	49649062	46415402	93.49%	9.79
TWS	4,642,395,300	4,634,158,500	99.82	0.03	96.14	90.09	36.3	30894390	29380812	95.10%	5.93
YAC	5,973,038,100	5,956,736,100	99.73	0.03	96.25	90.59	36.13	39711574	38733799	97.54%	7.8
YAC	8,723,767,500	8,715,477,600	99.9	0.03	97.4	92.43	36.54	58103184	56568614	97.36%	11.71

YAC	7,090,115,400	7,081,736,700	99.88	0.03	96.41	90.86	36.03	47211578	45796185	97.00%	9.64
YAC	6,499,142,100	6,489,939,300	99.86	0.03	97.33	92.4	37.84	43266262	39330688	90.90%	7.94
YAC	6,208,502,100	6,183,353,400	99.59	0.03	96.95	91.76	39.45	41222356	35605364	86.37%	6.84
YAC	7,487,375,400	7,474,419,600	99.83	0.03	97.14	92.07	42.46	49829464	38529303	77.32%	7.86
YAC	8,632,664,100	8,605,046,700	99.68	0.03	95.82	89.23	36.68	57366978	54177046	94.44%	11.69
YAC	9,371,334,900	9,347,218,500	99.74	0.03	95.44	88.57	38	62314790	55970946	89.82%	12.05
YAC	6,842,607,000	6,305,564,700	92.15	0.03	97.35	92.48	37.71	42037098	39208959	93.27%	8.18
YAC	6,306,074,100	6,253,064,700	99.16	0.03	97.12	91.97	37.24	38255838	36574019	95.60%	7.76
YAC	6,586,729,500	6,568,228,800	99.72	0.03	96.62	91.13	42.4	43788192	32695979	74.67%	6.55
YAC	6,843,378,600	6,832,506,600	99.84	0.03	96.62	90.97	39.84	45550044	38267997	84.01%	8.12
ZLD	6,875,681,400	6,860,598,900	99.78	0.03	96.4	90.87	38.67	45737326	37076292	81.06%	7.41
ZLD	6,358,422,300	6,346,702,500	99.82	0.03	97.45	92.7	38.44	42311350	35125299	83.02%	6.88
ZLD	6,956,871,600	6,931,991,700	99.64	0.03	97.17	92.21	40.83	46213278	30402919	65.79%	5.84
ZLD	6,744,188,100	6,733,115,700	99.84	0.03	95.8	89.75	39.94	44887438	33856533	75.43%	6.56
ZLD	6,785,904,900	6,775,801,500	99.85	0.03	97.12	92.03	37.77	45172010	38059500	84.25%	7.44
ZLD	6,838,296,000	6,825,965,700	99.82	0.03	97.14	92.04	40.39	45506438	31195133	68.55%	5.95
ZLD	6,061,060,800	6,049,448,400	99.81	0.03	97.09	91.97	43.5	40329656	23947650	59.38%	4.31
ZLD	7,731,224,700	7,720,693,500	99.86	0.03	97.09	91.97	39.52	51471290	40896294	79.45%	8.03
ZLD	6,246,576,300	6,236,551,800	99.84	0.03	97.19	92.14	37.16	41577012	35899020	86.34%	6.96
ZLD	6,432,830,400	6,422,178,300	99.83	0.03	95.9	89.91	39.05	42814522	33748463	78.82%	6.58
ZLD	6,984,672,900	6,969,746,100	99.79	0.03	97.25	92.33	44.3	46464974	28949365	62.30%	5.51
ZLD	5,788,356,000	5,761,077,900	99.53	0.03	96.19	90.23	36.73	38407186	33212166	86.47%	6.23
ZLD	4,971,240,000	4,956,366,900	99.7	0.03	96.72	91.33	38.89	33042446	24745910	74.89%	4.29
ZLD	4,808,106,000	4,794,428,100	99.72	0.03	96.05	89.96	38.27	31,962,854	25967890	81.24%	4.7
ZSC	6,376,942,500	6,364,710,000	99.81	0.03	97.38	92.5	36.32	42431400	39991584	94.25%	8.08
ZSC	5,951,259,600	5,930,901,600	99.66	0.03	96.72	91.25	37.33	40663912	36633529	90.09%	7.53

ZSC	6,187,240,500	6,168,469,200	99.7	0.03	96.71	91.22	36.63	41123128	38557007	93.76%	7.69
ZSC	6,675,441,900	6,652,263,000	99.65	0.03	97.12	92.14	39.22	44348420	35992560	81.16%	7.61
ZSC	7,741,995,600	7,731,251,100	99.86	0.03	95.5	88.69	35.89	51541674	48352062	93.81%	10.16
ZSC	6,487,035,000	6,470,668,800	99.75	0.03	96.45	90.67	36.58	43137792	40610430	94.14%	8.33
ZSC	6,192,423,000	6,178,078,500	99.77	0.03	96.56	90.91	37.2	41187190	38026498	92.33%	7.72
ZSC	5,843,211,000	5,834,028,000	99.84	0.03	96.03	89.94	36.03	38893520	35422883	91.08%	6.39
ZSC	5,482,441,200	5,470,470,600	99.78	0.03	96.45	90.77	40.2	36469804	27736797	76.05%	5.16
ZSC	6,553,498,200	6,518,366,100	99.46	0.03	97.44	93.03	37.75	58520166	51796155	88.51%	10.19
ZSC	6,947,280,600	6,923,946,300	99.66	0.03	96.38	90.58	37.3	46159642	42314179	91.67%	8.73
ZSC	6,680,467,800	6,665,137,800	99.77	0.03	96.16	90.08	36.84	44434252	40813089	91.85%	8.24
ZSC	6,742,899,600	6,704,463,300	99.43	0.03	96.59	90.95	39.03	44696422	36025306	80.60%	6.94

Table S3. The number of heterogenous and homogenous SNPs in samples.

sample	miss	gene	het	hom	snp	ratio
JSC	91285	2654392	431249	379985	811234	0.5315963
JSC	72053	2655910	440643	388305	828948	0.5315689
JSC	30496	2684041	417746	424628	842374	0.4959151
JSC	123022	2654687	407090	372112	779202	0.5224448
JSC	22031	2683479	446277	405124	851401	0.5241678
JSC	206469	2617501	369885	363056	732941	0.5046586
JSC	139996	2639126	406445	371344	777789	0.5225646
JSC	86325	2681217	386203	403166	789369	0.4892553
JSC	105617	2648659	423786	378849	802635	0.5279934
JSC	105261	2653633	418769	379248	798017	0.524762
JSC	116556	2644300	416404	379651	796055	0.5230845
JSC	115817	2637881	425195	378018	803213	0.5293677
JSC	124362	2628695	428200	375654	803854	0.5326838
JZD	191720	1895032	1223603	246556	1470159	0.832293
JZD	235264	1884333	1191259	246055	1437314	0.8288092
JZD	380731	1859051	1082353	234776	1317129	0.8217517
JZD	272621	1885300	1158285	240705	1398990	0.8279437
JZD	175058	1872016	1260725	249112	1509837	0.8350074
JZD	166414	1904367	1227368	258762	1486130	0.825882
JZD	168815	1891495	1253317	243284	1496601	0.8374423
JZD	237921	1880970	1197691	240329	1438020	0.8328751
JZD	254563	1889400	1168863	244085	1412948	0.8272513
JZD	218911	1868787	1222669	246544	1469213	0.8321932
JZD	208983	1871269	1227551	249108	1476659	0.831303
JZD	219322	1888290	1202189	247110	1449299	0.8294969
JZD	207095	1888023	1218381	243412	1461793	0.8334839
JZD	219745	1893964	1196245	246957	1443202	0.8288826
JZD	423807	1837457	1067969	227678	1295647	0.8242747
TWS	833647	2008496	253817	460951	714768	0.355104
TWS	386512	2258624	175352	736423	911775	0.1923194
TWS	535589	2184717	212723	623882	836605	0.2542693
TWS	380273	2261616	160131	754891	915022	0.1750024
TWS	380146	2259479	175754	741532	917286	0.1916022
TWS	471943	2214877	200442	669649	870091	0.230369
TWS	466095	2223141	193253	674422	867675	0.2227251
TWS	283256	2288794	154524	830337	984861	0.1568993
TWS	446959	2225490	190606	693856	884462	0.215505
TWS	298151	2293877	158892	805991	964883	0.1646749
SYC	152973	2796272	371538	236128	607666	0.6114181
SYC	141644	2805101	372579	237587	610166	0.6106191
SYC	88126	2835949	385424	247412	632836	0.6090425
SYC	81968	2842869	386706	245368	632074	0.6118049

SYC	116688	2823632	370634	245957	616591	0.6011019
SYC	101283	2832190	374717	248721	623438	0.6010493
SYC	80421	2839157	384395	252938	637333	0.6031305
SYC	86860	2846530	373584	249937	623521	0.5991522
SYC	328419	2677878	336363	214251	550614	0.6108871
SYC	83378	2848902	376741	247890	624631	0.6031417
SYC	158836	2810638	351217	236220	587437	0.5978803
SYC	81909	2843739	377513	253750	631263	0.5980281
SYC	115020	2827143	367474	247274	614748	0.5977636
SMD	134372	2457427	689882	275230	965112	0.7148207
SMD	103482	2477166	701762	274501	976263	0.7188247
SMD	108105	2468286	780629	199891	980520	0.7961378
SMD	159773	2450962	737819	208357	946176	0.7797904
SMD	174530	2441996	669627	270758	940385	0.7120775
SMD	65077	2478144	804231	209459	1013690	0.7933698
SMD	64196	2478124	809386	205205	1014591	0.7977461
SMD	75956	2444783	825822	210350	1036172	0.7969932
SMD	125977	2443080	776284	211570	987854	0.7858287
SMD	214476	2417186	719734	205515	925249	0.7778814
SMD	103160	2469173	706476	278102	984578	0.7175419
SMD	105210	2468311	709239	274151	983390	0.7212184
SMD	63979	2444781	839404	208747	1048151	0.8008426
SMD	100327	2469055	735965	251564	987529	0.7452591
SMD	160972	2442199	748923	204817	953740	0.7852486
TPC	65049	2843228	449317	199317	648634	0.6927127
TPC	39298	2862066	447331	208216	655547	0.6823782
TPC	24999	2868626	459042	204244	663286	0.6920725
TPC	79630	2843888	431831	201562	633393	0.6817742
TPC	130226	2810882	419272	196531	615803	0.6808541
TPC	87801	2800049	473557	195504	669061	0.7077935
TPC	76708	2849977	436414	193812	630226	0.6924722
TPC	134900	2813121	402086	206804	608890	0.660359
TPC	54530	2854856	433803	213722	647525	0.6699402
TPC	85162	2853462	431116	187171	618287	0.6972749
TPC	51943	2849492	437026	218450	655476	0.6667307
TPC	63338	2850800	438457	204316	642773	0.6821335
TPC	36466	2864313	447731	208401	656132	0.6823795
TPC	62136	2850107	441637	203031	644668	0.6850611
TPC	65241	2840126	443116	208428	651544	0.6801014
ZSC	106566	2480759	678188	291398	969586	0.6994614
ZSC	140449	2397471	738921	280070	1018991	0.7251497
ZSC	116607	2403351	755479	281474	1036953	0.7285566
ZSC	122524	2401698	750359	282330	1032689	0.7266069
ZSC	55231	2403065	816573	282042	1098615	0.7432749

ZSC	102471	2407839	762966	283635	1046601	0.7289941
ZSC	116173	2438962	714298	287478	1001776	0.7130317
ZSC	142886	2393219	740757	280049	1020806	0.7256589
ZSC	136113	2400127	740657	280014	1020671	0.7256569
ZSC	125608	2401014	749421	280868	1030289	0.7273891
ZSC	81838	2407176	785309	282588	1067897	0.735379
ZSC	92932	2408889	772199	282891	1055090	0.7318797
ZSC	182335	2379494	717435	277647	995082	0.7209808
TWS	328798	2276098	167178	784837	952015	0.1756044
TWS	274254	2296590	154847	831220	986067	0.157035
TWS	223171	2313184	142021	878535	1020556	0.1391604
TWS	275951	2296850	148762	835348	984110	0.151164
YAC	141311	2789149	352843	273608	626451	0.5632412
YAC	29871	2832609	389473	304958	694431	0.560852
YAC	49293	2811079	411418	285121	696539	0.5906604
YAC	108200	2781171	397402	270138	667540	0.5953231
YAC	193101	2728530	380535	254745	635280	0.5990036
YAC	84695	2793186	404405	274625	679030	0.5955628
YAC	34540	2837050	378893	306428	685321	0.5528694
YAC	30093	2844876	364637	317305	681942	0.5347038
YAC	103832	2804627	374500	273952	648452	0.5775293
YAC	121182	2805017	350387	280325	630712	0.555542
YAC	185332	2735116	380871	255592	636463	0.5984181
YAC	93644	2805491	373027	284749	657776	0.5671034
YMC	101253	2650606	420324	384728	805052	0.5221079
YMC	141709	2635937	407228	372037	779265	0.5225796
YMC	128899	2624939	430458	372615	803073	0.5360135
YMC	107072	2655447	413728	380664	794392	0.5208109
YMC	167626	2615157	403686	370442	774128	0.5214719
YMC	54896	2675339	424931	401745	826676	0.5140236
YMC	297888	2542307	377806	338910	716716	0.5271349
YMC	68131	2670588	424732	393460	818192	0.5191104
YMC	31289	2665166	457605	402851	860456	0.5318169
YMC	51344	2673972	434774	396821	831595	0.5228194
YMC	79783	2667603	422030	387495	809525	0.5213304
YMC	177336	2628358	376583	374634	751217	0.5012972
YMC	76818	2670193	418135	391765	809900	0.5162798
YMC	111246	2646515	417050	382100	799150	0.521867
ZLD	215101	1874200	1184477	283133	1467610	0.8070789
ZLD	312747	1855810	1119955	268399	1388354	0.8066783
ZLD	234497	1868923	1179487	274004	1453491	0.8114856
ZLD	256370	1872791	1149173	278577	1427750	0.8048839
ZLD	291023	1864032	1127622	274234	1401856	0.8043779
ZLD	431116	1818517	1052199	255079	1307278	0.8048778

ZLD	326350	1855416	1109974	265171	1375145	0.8071687
ZLD	247311	1876789	1160224	272587	1432811	0.8097537
ZLD	432091	1830769	1035695	258356	1294051	0.800351
ZLD	651283	1750639	923154	231835	1154989	0.7992751
ZLD	219853	1875607	1180074	281377	1461451	0.8074674
ZLD	286853	1868917	1127405	273736	1401141	0.8046335
ZLD	327407	1847271	1111980	270253	1382233	0.8044809
ZLD	421129	1834404	1045194	256184	1301378	0.8031441

Table S4. Population genetic summary statistics. Mean (±standard deviation) values of nucleotide diversity π, Tajima’s D statistic, pairwise relative measure of differentiation (FST, below the diagonal) and absolute divergence (Dxy, above the diagonal) between all comparisons.

						Fst/Dxy($\times 10^{-3}$)		
Species	Populations	No. of individuals	π($\times 10^{-3}$)	Tajima's D	Recombination rate (ρ)	*A. chinensis*	*A. deliciosa*	*A. setosa*
A. chinensis	YMC+TPC+YAC+SYC	4+4+4+4	0.882±0.469	0.46±0.65	0.0079±0.059	——	1.01±0.26	0.96±0.27
A. deliciosa	JZD+ZLD	8+8	1.331±0.677	1.61±0.62	0.0702±0.092	0.23±0.12	——	1.33±0.24
A. setosa	TWS	14	0.22±0.152	0.38±0.72	0.0016±0.035	0.61±0.14	0.51±0.11	——

Table S5 Comparison of population genomic parameters (the mean ± standard deviation values) of genomic islands with the rest of the genomic regions for all pairwise comparisons by Mann–Whitney U test.

Parameter	Species	Genomic background	Genomic islands	*P*-value
Chi-Del				
F_{ST}		0.21±0.09	0.57±0.10	2.20E-16
Dxy		0.00099±0.00025	0.0012±0.00021	2.20E-16
π	Chi	0.00090±0.00047	0.00051±0.00035	2.20E-16
	Del	0.00135±0.00067	0.00098±0.00064	2.20E-16
Tajima's D	Chi	0.47±0.65	0.34±0.73	1.76E-09
	Del	1.62±0.60	1.53±0.87	2.70E-02
ρ	Chi	0.0081±0.059	0.0036±0.036	2.20E-16
	Del	0.071±0.093	0.053±0.064	2.20E-16
Chi-Set				
F_{ST}		0.59±0.13	0.83±0.03	2.20E-16
Dxy		0.00095±0.00027	0.0011±0.00029	2.20E-16
π	Chi	0.00090±0.00047	0.00048±0.00024	2.20E-16
	Set	0.00022±0.00015	0.00023±0.00011	8.94E-15
Tajima's D	Chi	0.49±0.63	-0.15±0.68	2.20E-16
	Set	0.42±0.72	-0.24±0.39	2.20E-16
ρ	Chi	0.0082±0.060	0.0025±0.019	2.20E-16
	Set	0.0017±0.036	0.00045±0.010	2.20E-16
Del-Set				
F_{ST}		0.50±0.099	0.76±0.05	2.20E-16
Dxy		0.0013±0.00024	0.0014±0.00024	5.23E-08
π	Del	0.0014±0.00067	0.00068±0.00039	2.20E-16
	Set	0.00022±0.00015	0.00026±0.00015	2.20E-16
Tajima's D	Del	1.65±0.57	0.57±0.71	2.20E-16
	Set	0.40±0.72	-0.08±0.45	2.20E-16
ρ	Del	0.072±0.093	0.030±0.057	2.20E-16
	Set	0.0017±0.036	0.00008±0.0002	2.20E-16
Oceanic isolation				
F_{ST}		0.21±0.056	0.40±0.05	2.20E-16
Dxy		0.00034±0.00007	0.0031±0.00008	2.20E-16
π	island	0.0010±0.00049	0.0012±0.00049	2.20E-16
	mainland	0.0011±0.00053	0.00090±0.00045	2.20E-16
Tajima's D	island	2.10±0.70	2.51±0.78	2.20E-16
	mainland	0.28±0.53	-0.06±0.57	2.20E-16
ρ	island	0.00015±0.00029	0.000072±0.00015	2.20E-16
	mainland	0.0055±0.053	0.0026±0.016	2.20E-16
Lake isolation				
F_{ST}		0.073±0.050	0.28±0.07	2.20E-16

Dxy		0.00065±0.00034	0.00074±0.00039	2.71E-11
π	island	0.00066±0.00041	0.00058±0.00045	4.39E-12
	neighbor land	0.00066±0.00041	0.00056±0.00040	2.20E-16
Tajima's D	island	0.87±0.79	0.62±1.14	2.05E-11
	neighbor land	0.91±0.82	0.80±1.03	1.60E-03
ρ	island	0.011±0.10	0.0056±0.048	2.20E-16
	neighbor land	0.010±0.10	0.0056±0.051	2.20E-16

Table S6. Genes in genomic islands of three comparisons of the three species *A. chinensis* (Chi), *A. deliciosa* (Del) and *A. setosa* (Set) under strong selective sweeps.

comparison	CHROM	BIN_START	END	GeneID	anno
Chi-Del	Chr13	9778509	9780888	Ach13g352911	AGL6b [Actinidia chinensis]
Chi-Del	Chr18	12942028	12949105	Ach18g072001	beta-amylase 7 [Camellia sinensis]
Chi-Del	Chr07	6312396	6312689	Ach07g437001	Calcium-dependent ARF-type GTPase activating protein family isoform 1 [Theobroma cacao]
Chi-Set	Chr15	11573251	11582310	Ach15g455311	Calcium-dependent protein kinase 20 [Morus notabilis]
Chi-Del	Chr23	12938789	12983041	Ach23g347661	Callose synthase [Medicago truncatula]
Chi-Set	Chr26	1033647	1034708	Ach26g297111	calmodulin 1 [Lilium longiflorum]
Chi-Del	Chr16	774777	783408	Ach16g213671	calmodulin-binding protein [Nicotiana tabacum]
Chi-Del	Chr13	5838326	5841359	Ach13g146501	Caspase, putative [Theobroma cacao]
Chi-Del	Chr08	16549321	16554137	Ach08g152481	chlorophyll synthase [Camellia sinensis]
Chi-Del	Chr15	7450197	7461029	Ach15g001951	chlorophyll synthase [Nicotiana tabacum]
Chi-Del	Chr03	19898542	19915320	Ach03g185531	conserved hypothetical protein [Ricinus communis]
Chi-Del	Chr15	6656953	6658065	Ach15g001671	Cucumisin precursor, putative [Ricinus communis]
Chi-Set	Chr26	15088250	15091224	Ach26g179151	cyclin B [Camellia sinensis]
Chi-Del	Chr23	12756524	12760665	Ach23g043101	cyclin family protein [Populus trichocarpa]
Chi-Del	Chr15	2476493	2480052	Ach15g380531	Cytosolic enolase 3 [Morus notabilis]
Chi-Del	Chr15	6969575	6974638	Ach15g002441	DEA(D/H)-box RNA helicase family protein isoform 1 [Theobroma cacao]
Chi-Del	Chr12	5591974	5598311	Ach12g428481	Desacetoxyvindoline 4-hydroxylase, putative [Ricinus communis]
Chi-Set	Chr03	19377316	19377611	Ach03g447291	DNA repair metallo-beta-lactamase family protein isoform 1 [Theobroma cacao]
Chi-Del	Chr24	13526954	13546436	Ach24g406991	embryonic flower 2 [Camellia sinensis]
Chi-Set	Chr04	1420952	1424072	Ach04g123311	enhancer OF AG-4-like protein, putative [Medicago truncatula]
Chi-Del	Chr15	7439157	7442648	Ach15g002291	FAR1-related protein [Medicago truncatula]
Chi-Del	Chr17	12600942	12609921	Ach17g173191	F-box family protein [Populus trichocarpa]
Chi-Del	Chr17	12609824	12616473	Ach17g173041	Galacturonosyltransferase 10 [Theobroma cacao]
Chi-Del	Chr15	6854068	6855936	Ach15g002491	GRAS family transcription factor [Theobroma cacao]
Chi-Set	Chr07	16424447	16425010	Ach07g073911	H0502G05.11 protein [Theobroma cacao]
Chi-Del	Chr16	2970009	2979431	Ach16g035801	hypothetical protein AMTR_s00049p00143510 [Amborella trichopoda]

Chi-Del	Chr05	17714741	17718720	Ach05g269951	hypothetical protein B456_003G012100 [Gossypium raimondii]
Chi-Set	Chr13	3188	26572	Ach13g229511	hypothetical protein B456_007G118500 [Gossypium raimondii]
Chi-Del	Chr25	47461	48321	Ach25g452061	hypothetical protein B456_012G136700 [Gossypium raimondii]
Chi-Del	Chr19	6336219	6351692	Ach19g382841	hypothetical protein CARUB_v10008779mg [Capsella rubella]
Chi-Del	Chr04	9732044	9735287	Ach04g280441	hypothetical protein CICLE_v10000281mg [Citrus clementina]
Chi-Del	Chr29	9637568	9641228	Ach29g386491	hypothetical protein CICLE_v10006030mg [Citrus clementina]
Chi-Del	Chr15	6040338	6042052	Ach15g002861	hypothetical protein CICLE_v10023737mg [Citrus clementina]
Chi-Set	Chr25	1263332	1294839	Ach25g098761	hypothetical protein CICLE_v10030510mg [Citrus clementina]
Chi-Del	Chr26	8330401	8330634	Ach26g456951	hypothetical protein CICLE_v10033613mg [Citrus clementina]
Chi-Set	Chr26	3259496	3270987	Ach26g253461	hypothetical protein CISIN_1g002661mg [Citrus sinensis]
Chi-Del	Chr05	709545	710903	Ach05g205151	hypothetical protein CISIN_1g008539mg [Citrus sinensis]
Chi-Set	Chr14	4488129	4510607	Ach14g206201	hypothetical protein CISIN_1g010372mg [Citrus sinensis]
Chi-Set	Chr07	16428190	16433838	Ach07g074101	hypothetical protein CISIN_1g011701mg [Citrus sinensis]
Chi-Del	Chr11	11531596	11541146	Ach11g225731	hypothetical protein CISIN_1g011811mg [Citrus sinensis]
Chi-Del	Chr28	700964	706529	Ach28g378141	hypothetical protein CISIN_1g018568mg [Citrus sinensis]
Chi-Del	Chr05	17619543	17624247	Ach05g206791	hypothetical protein CISIN_1g020187mg [Citrus sinensis]
Chi-Del	Chr15	6833457	6838011	Ach15g392421	hypothetical protein CISIN_1g022953mg [Citrus sinensis]
Chi-Del	Chr29	9622158	9622520	Ach29g386751	hypothetical protein CISIN_1g0373262mg, partial [Citrus sinensis]
Chi-Del	Chr12	119856	120173	Ach12g473371	hypothetical protein CISIN_1g038383mg, partial [Citrus sinensis]
Chi-Del	Chr12	5620223	5622483	Ach12g119981	hypothetical protein EUGRSUZ_D02508 [Eucalyptus grandis]
Chi-Del	Chr13	9771233	9776477	Ach13g352191	hypothetical protein glysoja_042070 [Glycine soja]
Chi-Set	Chr07	11737508	11737993	Ach07g110781	hypothetical protein JCGZ_17729 [Jatropha curcas]

Chi-Del	Chr08	5953108	5953410	Ach08g112381	hypothetical protein L484_001231 [Morus notabilis]
Chi-Del	Chr03	1192888	1209459	Ach03g358811	hypothetical protein L484_011867 [Morus notabilis]
Chi-Del	Chr23	11336752	11339550	Ach23g397211	hypothetical protein L484_017501 [Morus notabilis]
Chi-Set	Chr15	5386478	5399462	Ach15g227961	hypothetical protein L484_023337 [Morus notabilis]
Chi-Del	Chr06	13806687	13833896	Ach06g247081	hypothetical protein L484_024148 [Morus notabilis]
Chi-Set	Chr05	1002001	1008620	Ach05g205081	hypothetical protein MIMGU_mgv1a019111mg, partial [Erythranthe guttata]
Chi-Set	Chr05	986752	987078	Ach05g205371	hypothetical protein MIMGU_mgv1a020512mg, partial [Erythranthe guttata]
Chi-Set	Chr18	5451709	5455124	Ach18g413151	hypothetical protein PHAVU_002G038400g [Phaseolus vulgaris]
Chi-Set	Chr21	6912518	6913192	Ach21g290371	hypothetical protein PHAVU_004G096500g [Phaseolus vulgaris]
Chi-Del	Chr26	8339621	8346731	Ach26g298081	hypothetical protein PHAVU_010G036200g [Phaseolus vulgaris]
Chi-Set	Chr15	15737554	15738328	Ach15g219441	hypothetical protein POPTR_0001s27010g [Populus trichocarpa]
Chi-Del	Chr15	5404040	5405543	Ach15g227981	hypothetical protein POPTR_0003s08530g [Populus trichocarpa]
Chi-Del	Chr26	5268978	5280294	Ach26g084151	hypothetical protein POPTR_0003s09480g [Populus trichocarpa]
Chi-Set	Chr26	1126950	1131119	Ach26g406621	hypothetical protein POPTR_0012s03950g [Populus trichocarpa]
Chi-Set	Chr26	1118402	1120882	Ach26g406611	hypothetical protein POPTR_0015s05370g [Populus trichocarpa]
Chi-Set	Chr04	477802	484443	Ach04g205031	hypothetical protein POPTR_0229s00250g [Populus trichocarpa]
Chi-Set	Chr21	6917846	6929065	Ach21g290811	hypothetical protein PRUPE_ppa000257mg [Prunus persica]
Chi-Del	Chr15	6656450	6656776	Ach15g392451	hypothetical protein PRUPE_ppa002068mg [Prunus persica]
Chi-Set	Chr11	12489143	12529958	Ach11g100661	hypothetical protein PRUPE_ppa002593mg [Prunus persica]
Chi-Set	Chr09	10658612	10661512	Ach09g019841	hypothetical protein PRUPE_ppa003787mg [Prunus persica]
Chi-Set	Chr24	167188	172357	Ach24g102521	hypothetical protein PRUPE_ppa005480mg [Prunus persica]

Chi-Del	Chr15	6915031	6919102	Ach15g001791	hypothetical protein PRUPE_ppa005786mg [Prunus persica]
Chi-Set	Chr14	158437	159126	Ach14g375271	hypothetical protein PRUPE_ppa005821mg [Prunus persica]
Chi-Del	Chr08	16523864	16525770	Ach08g342231	hypothetical protein PRUPE_ppa006591mg [Prunus persica]
Chi-Del	Chr23	19688458	19689720	Ach23g320621	hypothetical protein PRUPE_ppa009851mg [Prunus persica]
Chi-Del	Chr23	11332460	11335637	Ach23g069221	hypothetical protein PRUPE_ppa010017mg [Prunus persica]
Chi-Del	Chr05	17734165	17736583	Ach05g452471	hypothetical protein PRUPE_ppa010911mg [Prunus persica]
Chi-Set	Chr13	12537232	12541293	Ach13g190461	hypothetical protein PRUPE_ppa011119mg [Prunus persica]
Chi-Del	Chr14	4810927	4811544	Ach14g237351	hypothetical protein PRUPE_ppa011414mg [Prunus persica]
Chi-Del	Chr08	2935968	2936291	Ach08g226561	hypothetical protein PRUPE_ppa015232mg [Prunus persica]
Chi-Set	Chr19	1857569	1863533	Ach19g276951	hypothetical protein PRUPE_ppa023494mg, partial [Prunus persica]
Chi-Del	Chr15	7679957	7680325	Ach15g392291	hypothetical protein PRUPE_ppa024857mg [Prunus persica]
Chi-Set	Chr07	165370	173093	Ach07g341051	hypothetical protein PRUPE_ppa025789mg, partial [Prunus persica]
Chi-Del	Chr15	6953008	6960611	Ach15g002451	hypothetical protein PRUPE_ppa027142mg, partial [Prunus persica]
Chi-Del	Chr15	7670201	7672862	Ach15g392301	hypothetical protein PRUPE_ppa1027125mg [Prunus persica]
Chi-Del	Chr23	17627893	17630774	Ach23g127461	hypothetical protein SOVF_075080 [Spinacia oleracea]
Chi-Del	Chr05	17741876	17756868	Ach05g452481	hypothetical protein SOVF_172130 [Spinacia oleracea]
Chi-Set	Chr21	10671976	10673084	Ach21g417641	hypothetical protein SOVF_205780 [Spinacia oleracea]
Chi-Del	Chr15	8895477	8910045	Ach15g154221	hypothetical protein VITISV_000578 [Vitis vinifera]
Chi-Del	Chr03	20166345	20167313	Ach03g185681	hypothetical protein VITISV_003244 [Vitis vinifera]
Chi-Set	Chr12	12944697	12950281	Ach12g435821	hypothetical protein VITISV_006775 [Vitis vinifera]
Chi-Del	Chr23	19682841	19687147	Ach23g320431	hypothetical protein VITISV_018909 [Vitis vinifera]
Chi-Del	Chr07	7930853	7931197	Ach07g393031	hypothetical protein VITISV_027661 [Vitis vinifera]

Chi-Set	Chr04	508581	508868	Ach04g204911	hypothetical protein VITISV_028502 [Vitis vinifera]
Chi-Del	Chr05	715752	733598	Ach05g205141	hypothetical protein VITISV_036910 [Vitis vinifera]
Chi-Del	Chr28	13780070	13781458	Ach28g085201	hypothetical protein VITISV_037734 [Vitis vinifera]
Chi-Set	Chr19	10426313	10430209	Ach19g198721	hypothetical protein VITISV_038451 [Vitis vinifera]
Chi-Del	Chr15	7447314	7447589	Ach15g002271	hypothetical protein ZEAMMB73_478467 [Zea mays]
Chi-Set	Chr02	8800345	8802810	Ach02g086621	Isoprenylcysteine carboxyl methyltransferase family [Theobroma cacao]
Chi-Del	Chr15	7194358	7198780	Ach15g002381	kinase family protein [Populus trichocarpa]
Chi-Del	Chr15	6682262	6684049	Ach15g002561	leucoanthocyanidin dioxygenase [Actinidia chinensis]
Chi-Del	Chr08	16535094	16540642	Ach08g152491	Metallopeptidase M24 family protein isoform 1 [Theobroma cacao]
Chi-Set	Chr02	10449993	10462347	Ach02g286581	MYB transcription factor [Camellia sinensis]
Chi-Del	Chr15	6633112	6634173	Ach15g002591	Nucleotide-sugar transporter family protein [Theobroma cacao]
Chi-Del	Chr03	1180624	1183125	Ach03g465751	O-Glycosyl hydrolases family 17 protein [Theobroma cacao]
Chi-Del	Chr05	6748590	6748937	Ach05g440351	pentatricopeptide repeat-containing protein 123, partial [Lippia rhodocnemis]
Chi-Set	Chr01	7216532	7231028	Ach01g285221	Peroxisomal fatty acid beta-oxidation multifunctional protein [Medicago truncatula]
Chi-Del	Chr09	9859912	9866251	Ach09g294431	PHD finger family protein / bromo-adjacent domain-containing protein isoform 1 [Theobroma cacao]
Chi-Set	Chr26	4433273	4436244	Ach26g251981	Plant intracellular ras group-related LRR 4 isoform 1 [Theobroma cacao]
Chi-Del	Chr15	8920878	8922938	Ach15g153731	polyubiquitin 3 [Medicago truncatula]
Chi-Del	Chr12	5588569	5590658	Ach12g120301	PREDICTED: 1-aminocyclopropane-1-carboxylate oxidase homolog 4-like isoform X2 [Vitis vinifera]
Chi-Del	Chr08	13334826	13349473	Ach08g320691	PREDICTED: 2-isopropylmalate synthase A-like [Vitis vinifera]
Chi-Set	Chr24	179074	187318	Ach24g102531	PREDICTED: 50S ribosomal protein L3-2, chloroplastic [Vitis vinifera]
Chi-Del	Chr18	12956387	12957173	Ach18g072011	PREDICTED: 60S ribosomal protein L36-2-like [Nicotiana tomentosiformis]

Chi-Set	Chr23	16475638	16497406	Ach23g191181	PREDICTED: ABC transporter B family member 25, mitochondrial-like isoform X2 [Elaeis guineensis]
Chi-Del	Chr02	11016044	11050611	Ach02g293961	PREDICTED: ABC transporter C family member 12-like [Fragaria vesca subsp. vesca]
Chi-Del	Chr26	8326051	8327895	Ach26g298001	PREDICTED: acetolactate synthase 3, chloroplastic-like [Prunus mume]
Chi-Del	Chr03	1184620	1188822	Ach03g358791	PREDICTED: acyl-CoA-binding domain-containing protein 1-like [Vitis vinifera]
Chi-Set	Chr15	15699915	15721579	Ach15g220031	PREDICTED: acyl-CoA-binding domain-containing protein 4-like isoform X1 [Vitis vinifera]
Chi-Set	Chr25	4029336	4071604	Ach25g409681	PREDICTED: ADP-ribosylation factor GTPase-activating protein AGD4-like [Citrus sinensis]
Chi-Del	Chr13	5910714	5916643	Ach13g146771	PREDICTED: ALA-interacting subunit 3-like [Malus domestica]
Chi-Del	Chr07	11089476	11101770	Ach07g392141	PREDICTED: aldo-keto reductase family 4 member C10-like [Pyrus x bretschneideri]
Chi-Del	Chr23	14486720	14489547	Ach23g081931	PREDICTED: alpha carbonic anhydrase 7-like [Jatropha curcas]
Chi-Del	Chr12	5624723	5632916	Ach12g119971	PREDICTED: alpha/beta hydrolase domain-containing protein 11 [Vitis vinifera]
Chi-Set	Chr14	5700663	5703350	Ach14g179781	PREDICTED: ankyrin repeat and protein kinase domain-containing protein 1-like [Erythranthe guttatus]
Chi-Set	Chr07	3806	18706	Ach07g410361	PREDICTED: ankyrin repeat domain-containing protein 2 [Jatropha curcas]
Chi-Del	Chr08	7415393	7418508	Ach08g291981	PREDICTED: annexin D4 [Prunus mume]
Chi-Set	Chr21	10660567	10668421	Ach21g041401	PREDICTED: AP-4 complex subunit mu-like isoform X1 [Solanum tuberosum]
Chi-Del	Chr15	6590459	6601623	Ach15g392461	PREDICTED: armadillo repeat-containing kinesin-like protein 2 isoform X2 [Nelumbo nucifera]
Chi-Set	Chr24	16951524	16964681	Ach24g312921	PREDICTED: aspartate--tRNA ligase, cytoplasmic-like [Solanum tuberosum]
Chi-Del	Chr05	2335529	2349310	Ach05g364021	PREDICTED: aspartic proteinase-like protein 2-like [Citrus sinensis]
Chi-Set	Chr07	9106978	9124123	Ach07g157401	PREDICTED: ATP-dependent 6-phosphofructokinase 5, chloroplastic [Nicotiana sylvestris]

Chi-Del	Chr15	8150700	8170353	Ach15g109391	PREDICTED: ATP-dependent zinc metalloprotease FTSH 10, mitochondrial-like [Gossypium raimondii]
Chi-Set	Chr04	12820548	12832966	Ach04g343601	PREDICTED: AT-rich interactive domain-containing protein 5-like [Solanum tuberosum]
Chi-Set	Chr09	10677363	10694586	Ach09g019851	PREDICTED: AT-rich interactive domain-containing protein 6-like isoform X2 [Nicotiana tomentosiformis]
Chi-Del	Chr27	8442471	8473125	Ach27g338481	PREDICTED: auxin transport protein BIG [Vitis vinifera]
Chi-Del	Chr13	9057761	9064846	Ach13g352541	PREDICTED: auxin-responsive protein IAA11 isoform X2 [Jatropha curcas]
Chi-Del	Chr09	9870415	9879182	Ach09g294421	PREDICTED: beta-galactosidase 3 [Vitis vinifera]
Chi-Del	Chr03	12049519	12072297	Ach03g153611	PREDICTED: beta-taxilin-like isoform X1 [Citrus sinensis]
Chi-Set	Chr22	11624782	11653554	Ach22g059741	PREDICTED: bromodomain and WD repeat-containing protein 3 isoform X1 [Vitis vinifera]
Chi-Set	Chr14	123735	131859	Ach14g375311	PREDICTED: calcium-dependent protein kinase 4 isoform X3 [Vitis vinifera]
Chi-Set	Chr05	10477799	10487350	Ach05g048301	PREDICTED: calcium-transporting ATPase 1-like [Vitis vinifera]
Chi-Set	Chr01	11004	11222	Ach01g461571	PREDICTED: cation/H(+) antiporter 15 [Eucalyptus grandis]
Chi-Set	Chr09	10696009	10700005	Ach09g019761	PREDICTED: cellulose synthase-like protein D4 [Eucalyptus grandis]
Chi-Set	Chr26	521556	521864	Ach26g296941	PREDICTED: CENP-B homolog protein 2-like [Camelina sativa]
Chi-Set	Chr21	5220383	5260530	Ach21g029241	PREDICTED: centromere-associated protein E isoform X1 [Vitis vinifera]
Chi-Set	Chr08	5707933	5719446	Ach08g452971	PREDICTED: chaperone protein dnaJ 1, mitochondrial [Vitis vinifera]
Chi-Set	Chr08	5698394	5707555	Ach08g452961	PREDICTED: chaperone protein dnaJ 1, mitochondrial-like [Citrus sinensis]
Chi-Set	Chr03	18903345	18904397	Ach03g218261	PREDICTED: chaperone protein dnaJ 49 [Solanum lycopersicum]
Chi-Del	Chr28	14905201	14933862	Ach28g429261	PREDICTED: chromatin structure-remodeling complex protein SYD [Erythranthe guttatus]
Chi-Del	Chr06	3469748	3471756	Ach06g183351	PREDICTED: CLAVATA3/ESR (CLE)-related protein 25 [Vitis vinifera]

Chi-Del	Chr05	796940	811996	Ach05g205111	PREDICTED: cleft lip and palate transmembrane protein 1 homolog [Populus euphratica]
Chi-Set	Chr08	5721103	5740149	Ach08g273401	PREDICTED: clustered mitochondria protein [Prunus mume]
Chi-Set	Chr08	20972142	20974956	Ach08g104201	PREDICTED: conserved oligomeric Golgi complex subunit 4 [Nelumbo nucifera]
Chi-Del	Chr15	7050589	7054765	Ach15g002401	PREDICTED: cyclin-T1-5-like [Vitis vinifera]
Chi-Del	Chr15	6639337	6640892	Ach15g002581	PREDICTED: cytochrome P450 76C4-like [Camelina sativa]
Chi-Del	Chr05	17678155	17699594	Ach05g269931	PREDICTED: D-2-hydroxyglutarate dehydrogenase, mitochondrial isoform X2 [Vitis vinifera]
Chi-Set	Chr10	5347069	5355651	Ach10g444561	PREDICTED: DEAD-box ATP-dependent RNA helicase 50 isoform X2 [Nelumbo nucifera]
Chi-Set	Chr25	10724145	10737341	Ach25g430441	PREDICTED: DNA-directed RNA polymerase IV subunit 1 isoform X2 [Vitis vinifera]
Chi-Del	Chr15	6756584	6768318	Ach15g392441	PREDICTED: DNA-directed RNA polymerase IV subunit 1 isoform X3 [Vitis vinifera]
Chi-Del	Chr07	6321855	6353508	Ach07g175321	PREDICTED: DNA-directed RNA polymerase V subunit 1 [Sesamum indicum]
Chi-Del	Chr29	9629049	9631996	Ach29g413991	PREDICTED: DNA-directed RNA polymerases II, IV and V subunit 9A [Sesamum indicum]
Chi-Set	Chr06	11916157	11945001	Ach06g276381	PREDICTED: endoribonuclease Dicer homolog 3a isoform X1 [Vitis vinifera]
Chi-Set	Chr19	10434928	10453354	Ach19g198711	PREDICTED: eukaryotic initiation factor 4A-11 [Vitis vinifera]
Chi-Del	Chr15	14310809	14331520	Ach15g274411	PREDICTED: eukaryotic translation initiation factor 4G [Jatropha curcas]
Chi-Del	Chr12	7778619	7779779	Ach12g058681	PREDICTED: F-box protein At3g07870-like [Jatropha curcas]
Chi-Set	Chr26	15094693	15095973	Ach26g179291	PREDICTED: F-box/kelch-repeat protein At5g26960 [Nelumbo nucifera]
Chi-Del	Chr08	5953756	5954175	Ach08g112771	PREDICTED: F-box/LRR-repeat protein 25-like [Vitis vinifera]
Chi-Set	Chr08	11493032	11494322	Ach08g237911	PREDICTED: flowering time control protein FPA isoform X2 [Jatropha curcas]
Chi-Set	Chr08	11489271	11492974	Ach08g237901	PREDICTED: flowering time control protein FPA-like [Pyrus x bretschneideri]

Chi-Del	Chr23	12731330	12736308	Ach23g042371	PREDICTED: formin-like protein 3 [Nicotiana sylvestris]
Chi-Del	Chr05	2332809	2334857	Ach05g364011	PREDICTED: FRIGIDA-like protein 3 [Vitis vinifera]
Chi-Del	Chr23	14617890	14619551	Ach23g422311	PREDICTED: GATA transcription factor 8-like [Vitis vinifera]
Chi-Del	Chr15	6966431	6968497	Ach15g001831	PREDICTED: general transcription factor IIE subunit 2-like [Nelumbo nucifera]
Chi-Set	Chr12	12942506	12943886	Ach12g167841	PREDICTED: glucan endo-1,3-beta-glucosidase 5 [Vitis vinifera]
Chi-Del	Chr08	2937453	2943672	Ach08g226951	PREDICTED: glucose-induced degradation protein 8 homolog isoform X1 [Citrus sinensis]
Chi-Del	Chr26	8337761	8338711	Ach26g298011	PREDICTED: glucuronoxylan 4-O-methyltransferase 1 [Vitis vinifera]
Chi-Set	Chr14	146281	157338	Ach14g375281	PREDICTED: glutamate receptor 3.2 [Populus euphratica]
Chi-Del	Chr24	2885664	2889339	Ach24g082421	PREDICTED: glutamine synthetase leaf isozyme, chloroplastic [Cucumis melo]
Chi-Del	Chr24	2892710	2896569	Ach24g422271	PREDICTED: glutamine synthetase leaf isozyme, chloroplastic [Populus euphratica]
Chi-Set	Chr10	11629030	11640673	Ach10g257071	PREDICTED: glutamine-dependent NAD(+) synthetase [Populus euphratica]
Chi-Del	Chr09	9400871	9409262	Ach09g405741	PREDICTED: glutathione S-transferase zeta class-like isoform X2 [Prunus mume]
Chi-Set	Chr01	11992485	12044553	Ach01g178631	PREDICTED: glycine--tRNA ligase 2, chloroplastic/mitochondrial isoform X1 [Vitis vinifera]
Chi-Del	Chr15	6623106	6627967	Ach15g002601	PREDICTED: guard cell S-type anion channel SLAC1 [Vitis vinifera]
Chi-Del	Chr23	14498594	14512523	Ach23g422291	PREDICTED: helicase protein MOM1 isoform X1 [Vitis vinifera]
Chi-Set	Chr16	11290714	11303747	Ach16g443221	PREDICTED: histidine--tRNA ligase [Vitis vinifera]
Chi-Set	Chr11	15458917	15477958	Ach11g216891	PREDICTED: histone acetyltransferase HAC1-like [Vitis vinifera]
Chi-Del	Chr12	7796025	7798169	Ach12g058691	PREDICTED: histone-lysine N-methyltransferase, H3 lysine-9 specific SUVH1 [Vitis vinifera]
Chi-Set	Chr06	14077151	14089478	Ach06g247261	PREDICTED: importin-4 [Vitis vinifera]
Chi-Del	Chr29	9608396	9610053	Ach29g386741	PREDICTED: inactive protein RESTRICTED TEV MOVEMENT 1-like [Sesamum indicum]
Chi-Set	Chr03	18907523	18927935	Ach03g218121	PREDICTED: kinesin-13A [Vitis vinifera]

Chi-Del	Chr07	1126033	1139642	Ach07g005811	PREDICTED: kinesin-related protein 11 isoform X1 [Vitis vinifera]
Chi-Del	Chr06	13796824	13800770	Ach06g247091	PREDICTED: kinetochore protein ndc80-like [Vitis vinifera]
Chi-Set	Chr02	8371251	8376963	Ach02g459471	PREDICTED: laccase-17-like [Nicotiana tomentosiformis]
Chi-Del	Chr08	9515782	9519100	Ach08g302041	PREDICTED: LDLR chaperone MESD [Vitis vinifera]
Chi-Del	Chr14	4782414	4783334	Ach14g237361	PREDICTED: leucine-rich repeat extensin-like protein 5 [Nicotiana tomentosiformis]
Chi-Del	Chr08	9493482	9508226	Ach08g302731	PREDICTED: LOW QUALITY PROTEIN: ATP-dependent helicase BRM [Prunus mume]
Chi-Set	Chr04	1419655	1419993	Ach04g428991	PREDICTED: LOW QUALITY PROTEIN: ENHANCER OF AG-4 protein 2 [Prunus mume]
Chi-Del	Chr15	6823210	6828758	Ach15g002501	PREDICTED: lysophospholipid acyltransferase 1-like [Pyrus x bretschneideri]
Chi-Del	Chr19	6327556	6332025	Ach19g382871	PREDICTED: MATE efflux family protein 6 isoform X1 [Vitis vinifera]
Chi-Del	Chr15	6574080	6574501	Ach15g392471	PREDICTED: MATE efflux family protein DTX1 [Jatropha curcas]
Chi-Set	Chr12	4491284	4507423	Ach12g075391	PREDICTED: MATH domain-containing protein At5g43560-like isoform X1 [Prunus mume]
Chi-Del	Chr02	12634966	12673926	Ach02g393321	PREDICTED: mediator of RNA polymerase II transcription subunit 13 [Vitis vinifera]
Chi-Del	Chr24	2973738	2997032	Ach24g082881	PREDICTED: mediator of RNA polymerase II transcription subunit 33A isoform X1 [Vitis vinifera]
Chi-Set	Chr07	11716739	11734125	Ach07g110771	PREDICTED: methyl-CpG-binding domain-containing protein 9-like [Sesamum indicum]
Chi-Del	Chr11	16587022	16594416	Ach11g404801	PREDICTED: mitochondrial adenine nucleotide transporter ADNT1-like [Nelumbo nucifera]
Chi-Set	Chr13	7057100	7062363	Ach13g214031	PREDICTED: mitoferrin [Nicotiana sylvestris]
Chi-Del	Chr06	13785314	13794879	Ach06g247141	PREDICTED: mitogen-activated protein kinase kinase kinase 1-like [Vitis vinifera]
Chi-Del	Chr11	16580726	16583080	Ach11g275911	PREDICTED: mitogen-activated protein kinase kinase kinase 7-like [Nicotiana sylvestris]

Chi-Del	Chr15	6325050	6345834	Ach15g001541	PREDICTED: monocopper oxidase-like protein SKU5 [Vitis vinifera]
Chi-Del	Chr10	7651585	7659967	Ach10g120401	PREDICTED: monoglyceride lipase-like [Jatropha curcas]
Chi-Set	Chr05	16236472	16289681	Ach05g207281	PREDICTED: MORC family CW-type zinc finger protein 3 [Vitis vinifera]
Chi-Set	Chr19	1843391	1855166	Ach19g453521	PREDICTED: mRNA-decapping enzyme-like protein [Jatropha curcas]
Chi-Del	Chr15	7010532	7016575	Ach15g001851	PREDICTED: myosin-9 isoform X1 [Vitis vinifera]
Chi-Set	Chr10	15203350	15204927	Ach10g168501	PREDICTED: non-specific lipid-transfer protein D, cotyledon-specific isoform-like [Citrus sinensis]
Chi-Del	Chr05	17641172	17653879	Ach05g269941	PREDICTED: nuclear-pore anchor isoform X1 [Nelumbo nucifera]
Chi-Del	Chr04	9737190	9746690	Ach04g280421	PREDICTED: nucleobase-ascorbate transporter 3 [Nicotiana sylvestris]
Chi-Del	Chr28	710252	738662	Ach28g378151	PREDICTED: paladin-like isoform X2 [Solanum tuberosum]
Chi-Del	Chr04	9721741	9722052	Ach04g280431	PREDICTED: pentatricopeptide repeat-containing protein At3g12770-like [Nelumbo nucifera]
Chi-Del	Chr23	12724313	12726439	Ach23g043081	PREDICTED: pentatricopeptide repeat-containing protein At3g29230 [Vitis vinifera]
Chi-Set	Chr26	148135	150036	Ach26g296991	PREDICTED: pentatricopeptide repeat-containing protein At4g18750, chloroplastic-like [Citrus sinensis]
Chi-Del	Chr15	8690772	8692293	Ach15g109061	PREDICTED: pentatricopeptide repeat-containing protein At5g02830, chloroplastic isoform X2 [Vitis vinifera]
Chi-Del	Chr15	14338779	14343740	Ach15g274401	PREDICTED: pentatricopeptide repeat-containing protein At5g39710-like [Vitis vinifera]
Chi-Del	Chr05	6747981	6748549	Ach05g440341	PREDICTED: pentatricopeptide repeat-containing protein At5g39980, chloroplastic [Cucumis sativus]
Chi-Del	Chr23	19690533	19697930	Ach23g320421	PREDICTED: peptidyl-prolyl cis-trans isomerase [Eucalyptus grandis]
Chi-Set	Chr26	525537	528588	Ach26g297051	PREDICTED: peroxidase 31-like [Eucalyptus grandis]
Chi-Set	Chr02	8257189	8266424	Ach02g312061	PREDICTED: phosphatidate phosphatase PAH1 isoform X2 [Vitis vinifera]

Chi-Del	Chr15	7693040	7700458	Ach15g002021	PREDICTED: phosphatidylinositol N-acetylglucosaminyltransferase GPI3 subunit-like [Nicotiana sylvestris]
Chi-Set	Chr12	12814502	12833463	Ach12g167891	PREDICTED: phosphoinositide phosphatase SAC3 [Vitis vinifera]
Chi-Del	Chr06	3482247	3502554	Ach06g183341	PREDICTED: phospholipid-transporting ATPase 3-like [Musa acuminata subsp. malaccensis]
Chi-Del	Chr07	11070440	11073502	Ach07g110601	PREDICTED: phytosulfokine receptor 1 [Vitis vinifera]
Chi-Del	Chr15	7038042	7047383	Ach15g002411	PREDICTED: plant intracellular Ras-group-related LRR protein 6-like [Malus domestica]
Chi-Del	Chr08	5936809	5949390	Ach08g112371	PREDICTED: polyamine oxidase 1 isoform X1 [Jatropha curcas]
Chi-Del	Chr16	2415214	2444300	Ach16g035851	PREDICTED: potassium channel SKOR [Vitis vinifera]
Chi-Del	Chr13	9075539	9090549	Ach13g464621	PREDICTED: pre-mRNA-processing factor 39-like isoform X1 [Populus euphratica]
Chi-Del	Chr15	6852791	6853414	Ach15g001761	PREDICTED: probable (S)-N-methylcoclaurine 3'-hydroxylase isozyme 2 [Vitis vinifera]
Chi-Set	Chr14	159208	159546	Ach14g467861	PREDICTED: probable anion transporter 5 [Fragaria vesca subsp. vesca]
Chi-Del	Chr05	17731592	17732883	Ach05g269921	PREDICTED: probable dimethyladenosine transferase [Nicotiana sylvestris]
Chi-Set	Chr04	11273958	11275196	Ach04g197621	PREDICTED: probable DNA-3-methyladenine glycosylase 2 [Vitis vinifera]
Chi-Set	Chr07	162189	164146	Ach07g340271	PREDICTED: probable flavin-containing monooxygenase 1 [Vitis vinifera]
Chi-Del	Chr25	6176841	6186514	Ach25g374401	PREDICTED: probable fructose-bisphosphate aldolase 3, chloroplastic [Sesamum indicum]
Chi-Set	Chr24	174903	177536	Ach24g103641	PREDICTED: probable GTP diphosphokinase CRSH, chloroplastic [Nelumbo nucifera]
Chi-Set	Chr10	5357751	5372312	Ach10g218811	PREDICTED: probable methionine--tRNA ligase [Sesamum indicum]
Chi-Set	Chr12	8657298	8671745	Ach12g192231	PREDICTED: probable methyltransferase PMT7-like [Solanum tuberosum]
Chi-Del	Chr26	5241500	5247018	Ach26g084161	PREDICTED: probable transcription factor KAN2 isoform X2 [Vitis vinifera]
Chi-Set	Chr02	11675628	11687335	Ach02g292921	PREDICTED: probable tRNA (guanine(26)-N(2))-dimethyltransferase 2 [Vitis vinifera]

Chi-Del	Chr11	3440075	3465530	Ach11g265331	PREDICTED: probable ubiquitin-like-specific protease 2B isoform X2 [Vitis vinifera]
Chi-Set	Chr02	8236998	8246039	Ach02g459491	PREDICTED: protein ALUMINUM SENSITIVE 3-like [Populus euphratica]
Chi-Del	Chr16	3126966	3135114	Ach16g035781	PREDICTED: protein argonaute 1-like isoform X2 [Nicotiana sylvestris]
Chi-Set	Chr07	9099080	9102739	Ach07g157411	PREDICTED: protein CURVATURE THYLAKOID 1D, chloroplastic isoform X2 [Vitis vinifera]
Chi-Del	Chr17	10868750	10871226	Ach17g221541	PREDICTED: protein DJ-1 homolog B [Sesamum indicum]
Chi-Del	Chr17	10874925	10880241	Ach17g444601	PREDICTED: protein DJ-1 homolog B-like [Jatropha curcas]
Chi-Del	Chr15	6724884	6733313	Ach15g001711	PREDICTED: protein FAM63A-like [Nelumbo nucifera]
Chi-Set	Chr09	10665865	10671596	Ach09g019771	PREDICTED: protein FAR1-RELATED SEQUENCE 5 [Jatropha curcas]
Chi-Del	Chr15	7442946	7443446	Ach15g002281	PREDICTED: protein FAR1-RELATED SEQUENCE 5-like [Malus domestica]
Chi-Set	Chr04	12836527	12846155	Ach04g343591	PREDICTED: protein FAR1-RELATED SEQUENCE 5-like [Vitis vinifera]
Chi-Set	Chr07	32245	37303	Ach07g340231	PREDICTED: protein FAR1-RELATED SEQUENCE 9 isoform X2 [Vitis vinifera]
Chi-Del	Chr15	6702555	6708289	Ach15g001701	PREDICTED: protein FIZZY-RELATED 2 [Vitis vinifera]
Chi-Set	Chr13	7043679	7051429	Ach13g214271	PREDICTED: protein FLX-like 1 [Nelumbo nucifera]
Chi-Del	Chr15	6902234	6911685	Ach15g002481	PREDICTED: protein HAPLESS 2 isoform X3 [Nicotiana sylvestris]
Chi-Del	Chr15	7672966	7673319	Ach15g002011	PREDICTED: protein NRT1/ PTR FAMILY 5.5 [Prunus mume]
Chi-Set	Chr12	11890546	11893617	Ach12g167311	PREDICTED: protein phosphatase 2C 77 isoform X2 [Vitis vinifera]
Chi-Del	Chr28	13794779	13802673	Ach28g085121	PREDICTED: protein ROOT PRIMORDIUM DEFECTIVE 1 [Vitis vinifera]
Chi-Set	Chr26	3271668	3277757	Ach26g404961	PREDICTED: protein RST1 [Vitis vinifera]
Chi-Del	Chr05	790234	793573	Ach05g205311	PREDICTED: protein SUPPRESSOR OF GENE SILENCING 3-like [Prunus mume]
Chi-Set	Chr01	7191879	7209407	Ach01g285301	PREDICTED: protein tesmin/TSO1-like CXC 5 [Vitis vinifera]
Chi-Set	Chr28	6057837	6061040	Ach28g093461	PREDICTED: protein TIC 62, chloroplastic [Populus euphratica]

Chi-Set	Chr13	81286	86948	Ach13g229481	PREDICTED: protein transport protein SEC23 [Vitis vinifera]
Chi-Set	Chr01	11922084	11945094	Ach01g178601	PREDICTED: protein VASCULAR ASSOCIATED DEATH 1, chloroplastic isoform X3 [Jatropha curcas]
Chi-Set	Chr18	11225757	11226260	Ach18g032111	PREDICTED: protein YIPF6 homolog [Jatropha curcas]
Chi-Del	Chr23	14589469	14607231	Ach23g422301	PREDICTED: protein-tyrosine sulfotransferase [Vitis vinifera]
Chi-Del	Chr17	323390	340674	Ach17g251361	PREDICTED: protoporphyrinogen oxidase 1, chloroplastic [Vitis vinifera]
Chi-Del	Chr08	13355918	13364630	Ach08g460261	PREDICTED: PTI1-like tyrosine-protein kinase 1 isoform X1 [Malus domestica]
Chi-Set	Chr05	10455966	10461885	Ach05g048251	PREDICTED: putative clathrin assembly protein At4g40080 [Sesamum indicum]
Chi-Del	Chr13	5891802	5903665	Ach13g146481	PREDICTED: putative cyclin-B3-1 isoform X2 [Vitis vinifera]
Chi-Del	Chr15	6585705	6586580	Ach15g001641	PREDICTED: putative DNA-binding protein ESCAROLA [Sesamum indicum]
Chi-Del	Chr13	7349599	7354183	Ach13g035191	PREDICTED: putative ER lumen protein-retaining receptor C28H8.4 [Vitis vinifera]
Chi-Del	Chr13	7340426	7346232	Ach13g417291	PREDICTED: putative G3BP-like protein [Sesamum indicum]
Chi-Set	Chr06	14051314	14067006	Ach06g247251	PREDICTED: putative leucine-rich repeat-containing protein DDB_G0290503-like [Solanum tuberosum]
Chi-Del	Chr24	6911246	6923316	Ach24g077191	PREDICTED: putative lysine-specific demethylase JMJ16 [Vitis vinifera]
Chi-Set	Chr06	14069441	14071366	Ach06g448381	PREDICTED: putative pentatricopeptide repeat-containing protein At3g49142 [Fragaria vesca subsp. vesca]
Chi-Del	Chr15	5401514	5403645	Ach15g227971	PREDICTED: putative pentatricopeptide repeat-containing protein At5g37570 [Vitis vinifera]
Chi-Del	Chr03	13020903	13032591	Ach03g328101	PREDICTED: R3H domain-containing protein 2 [Vitis vinifera]
Chi-Set	Chr04	510345	516636	Ach04g204901	PREDICTED: ran-binding protein 10-like [Jatropha curcas]
Chi-Set	Chr28	5870946	5872844	Ach28g093691	PREDICTED: ras-related protein Rab11C-like [Glycine max]
Chi-Set	Chr12	7614607	7628872	Ach12g058771	PREDICTED: regulation of nuclear pre-mRNA domain-containing protein 1B-like [Vitis vinifera]

Chi-Set	Chr14	5247923	5280939	Ach14g308531	PREDICTED: regulator of nonsense transcripts 1 homolog isoform X1 [Vitis vinifera]
Chi-Del	Chr15	5425198	5447565	Ach15g392661	PREDICTED: regulator of nonsense transcripts 1 homolog isoform X2 [Vitis vinifera]
Chi-Set	Chr11	150106	176713	Ach11g252691	PREDICTED: regulatory-associated protein of TOR 1-like isoform X1 [Nicotiana tomentosiformis]
Chi-Del	Chr15	7170972	7190860	Ach15g002391	PREDICTED: RNA polymerase II C-terminal domain phosphatase-like 2 isoform X2 [Vitis vinifera]
Chi-Set	Chr15	5365526	5386384	Ach15g445511	PREDICTED: RNA-binding protein 5-B isoform X1 [Vitis vinifera]
Chi-Set	Chr01	17971	28391	Ach01g331181	PREDICTED: SAL1 phosphatase-like [Nelumbo nucifera]
Chi-Del	Chr03	11924938	11943270	Ach03g153661	PREDICTED: serine carboxypeptidase-like 27 [Vitis vinifera]
Chi-Set	Chr28	6073912	6086098	Ach28g093451	PREDICTED: serine/arginine repetitive matrix protein 1 isoform X4 [Populus euphratica]
Chi-Del	Chr24	2861921	2871013	Ach24g082831	PREDICTED: serine/arginine-rich SC35-like splicing factor SCL28 isoform X3 [Nicotiana sylvestris]
Chi-Del	Chr12	11843545	11864288	Ach12g167291	PREDICTED: serine/threonine-protein kinase CBK1-like isoform X3 [Citrus sinensis]
Chi-Del	Chr12	134213	157432	Ach12g028831	PREDICTED: serine/threonine-protein kinase cst-1 isoform X2 [Vitis vinifera]
Chi-Set	Chr26	13423496	13430001	Ach26g108781	PREDICTED: serine/threonine-protein kinase GRIK2 [Vitis vinifera]
Chi-Del	Chr28	14903439	14903918	Ach28g123841	PREDICTED: serine/threonine-protein kinase Nek6 [Vitis vinifera]
Chi-Set	Chr12	8672155	8674033	Ach12g192401	PREDICTED: signal recognition particle subunit SRP72 [Vitis vinifera]
Chi-Del	Chr08	12657020	12659114	Ach08g096161	PREDICTED: sn1-specific diacylglycerol lipase beta isoform X4 [Populus euphratica]
Chi-Del	Chr15	6735146	6746814	Ach15g002541	PREDICTED: SPX domain-containing membrane protein At4g22990 [Vitis vinifera]
Chi-Set	Chr08	8665636	8686261	Ach08g379431	PREDICTED: SPX domain-containing membrane protein At4g22990-like [Nicotiana tomentosiformis]

Chi-Del	Chr28	666212	673174	Ach28g195011	PREDICTED: squamosa promoter-binding-like protein 1 [Vitis vinifera]
Chi-Del	Chr24	2842040	2855094	Ach24g082431	PREDICTED: squamosa promoter-binding-like protein 7 [Vitis vinifera]
Chi-Set	Chr04	11260510	11266031	Ach04g197301	PREDICTED: sugar transporter ERD6-like 6 [Prunus mume]
Chi-Set	Chr02	14102704	14131083	Ach02g039091	PREDICTED: sulfite oxidase [Nicotiana sylvestris]
Chi-Set	Chr12	12838265	12846682	Ach12g167781	PREDICTED: suppressor of mec-8 and unc-52 protein homolog 2 [Nelumbo nucifera]
Chi-Del	Chr17	12618958	12619881	Ach17g173051	PREDICTED: syntaxin-124 [Cucumis melo]
Chi-Set	Chr03	19365265	19372292	Ach03g240051	PREDICTED: syntaxin-22-like isoform X2 [Jatropha curcas]
Chi-Set	Chr26	15608558	15614949	Ach26g239601	PREDICTED: TATA-box-binding protein [Phoenix dactylifera]
Chi-Del	Chr16	5406459	5414172	Ach16g415911	PREDICTED: T-complex protein 1 subunit gamma-like [Camelina sativa]
Chi-Del	Chr16	5414243	5421130	Ach16g415901	PREDICTED: T-complex protein 1 subunit gamma-like [Musa acuminata subsp. malaccensis]
Chi-Del	Chr02	11073627	11087861	Ach02g292721	PREDICTED: T-complex protein 1 subunit zeta-like isoform X1 [Nicotiana sylvestris]
Chi-Del	Chr15	8696526	8703245	Ach15g466591	PREDICTED: thioredoxin-like protein AAED1, chloroplastic [Pyrus x bretschneideri]
Chi-Set	Chr28	5858702	5863915	Ach28g424631	PREDICTED: transcription elongation factor B polypeptide 1 isoform X2 [Sesamum indicum]
Chi-Set	Chr08	21053919	21062048	Ach08g104741	PREDICTED: transcription factor 25 [Vitis vinifera]
Chi-Del	Chr23	12701289	12710247	Ach23g043071	PREDICTED: transcription factor bHLH117 [Vitis vinifera]
Chi-Set	Chr13	7032153	7039207	Ach13g214021	PREDICTED: transcription initiation factor TFIID subunit 15b-like [Nicotiana tomentosiformis]
Chi-Del	Chr15	6842587	6848134	Ach15g001751	PREDICTED: transcriptional corepressor SEUSS isoform X1 [Malus domestica]
Chi-Del	Chr07	7917981	7926771	Ach07g224641	PREDICTED: transmembrane emp24 domain-containing protein p24delta3-like [Vitis vinifera]
Chi-Set	Chr08	21064458	21082244	Ach08g104161	PREDICTED: tricin synthase 1-like [Pyrus x bretschneideri]
Chi-Del	Chr24	6931941	6939092	Ach24g076871	PREDICTED: tubulin-folding cofactor B isoform X2 [Sesamum indicum]

Chi-Set	Chr01	6146014	6153421	Ach01g406681	PREDICTED: two-component response regulator ARR12 [Vitis vinifera]
Chi-Del	Chr08	18945711	19001248	Ach08g149291	PREDICTED: ubiquitin carboxyl-terminal hydrolase 12 isoform X1 [Jatropha curcas]
Chi-Set	Chr15	11587424	11646868	Ach15g455321	PREDICTED: ubiquitin carboxyl-terminal hydrolase 12 isoform X1 [Vitis vinifera]
Chi-Del	Chr02	11054828	11065311	Ach02g292701	PREDICTED: ubiquitin receptor RAD23c-like isoform X2 [Nicotiana tomentosiformis]
Chi-Del	Chr08	9476176	9491960	Ach08g302031	PREDICTED: ubiquitin-conjugating enzyme E2-23 kDa [Vitis vinifera]
Chi-Set	Chr07	11738152	11754109	Ach07g110791	PREDICTED: ubiquitin-like-specific protease 1D isoform X2 [Vitis vinifera]
Chi-Del	Chr01	3897981	3904657	Ach01g022861	PREDICTED: U-box domain-containing protein 35-like [Nicotiana sylvestris]
Chi-Set	Chr16	11267187	11271537	Ach16g443211	PREDICTED: U-box domain-containing protein 52-like [Sesamum indicum]
Chi-Set	Chr21	3664003	3665931	Ach21g372201	PREDICTED: UDP-glycosyltransferase 85A5-like [Prunus mume]
Chi-Del	Chr15	7021418	7032816	Ach15g392411	PREDICTED: ultraviolet-B receptor UVR8 isoform X1 [Vitis vinifera]
Chi-Del	Chr25	6202633	6210093	Ach25g373781	PREDICTED: uncharacterized PKHD-type hydroxylase At1g22950-like [Nelumbo nucifera]
Chi-Del	Chr07	7938836	7943173	Ach07g223951	PREDICTED: uncharacterized protein At1g10890-like [Musa acuminata subsp. malaccensis]
Chi-Del	Chr15	8172678	8176138	Ach15g109401	PREDICTED: uncharacterized protein At5g02240 [Cucumis sativus]
Chi-Del	Chr17	399660	401890	Ach17g251331	PREDICTED: uncharacterized protein C9orf78 homolog [Nelumbo nucifera]
Chi-Del	Chr17	391684	395284	Ach17g448871	PREDICTED: uncharacterized protein C9orf78 isoform X2 [Nicotiana sylvestris]
Chi-Del	Chr03	11945801	11957490	Ach03g433471	PREDICTED: uncharacterized protein LOC100240985 isoform X1 [Vitis vinifera]
Chi-Del	Chr06	13764508	13771866	Ach06g247101	PREDICTED: uncharacterized protein LOC100241871 [Vitis vinifera]
Chi-Del	Chr28	13651395	13652203	Ach28g290201	PREDICTED: uncharacterized protein LOC100243225 [Vitis vinifera]
Chi-Del	Chr10	8933155	8951091	Ach10g400501	PREDICTED: uncharacterized protein LOC100243690 [Vitis vinifera]
Chi-Set	Chr26	155111	174302	Ach26g296971	PREDICTED: uncharacterized protein LOC100244234 isoform X2 [Vitis vinifera]

Chi-Set	Chr05	973592	978534	Ach05g441301	PREDICTED: uncharacterized protein LOC100244237 isoform X1 [Vitis vinifera]
Chi-Set	Chr02	8805315	8818275	Ach02g086381	PREDICTED: uncharacterized protein LOC100244691 [Vitis vinifera]
Chi-Set	Chr24	137292	153431	Ach24g102511	PREDICTED: uncharacterized protein LOC100247698 [Vitis vinifera]
Chi-Del	Chr08	12639076	12653195	Ach08g096151	PREDICTED: uncharacterized protein LOC100248021 isoform X2 [Vitis vinifera]
Chi-Del	Chr11	3236415	3242433	Ach11g125331	PREDICTED: uncharacterized protein LOC100249059 [Vitis vinifera]
Chi-Del	Chr23	15381218	15385393	Ach23g172441	PREDICTED: uncharacterized protein LOC100250176 [Vitis vinifera]
Chi-Del	Chr29	13722144	13722506	Ach29g130091	PREDICTED: uncharacterized protein LOC100251046 [Vitis vinifera]
Chi-Set	Chr19	7827701	7841368	Ach19g399221	PREDICTED: uncharacterized protein LOC100251871 [Vitis vinifera]
Chi-Set	Chr23	16457027	16471022	Ach23g191171	PREDICTED: uncharacterized protein LOC100253529 isoform X2 [Vitis vinifera]
Chi-Del	Chr17	3827402	3837764	Ach17g460951	PREDICTED: uncharacterized protein LOC100253761 [Vitis vinifera]
Chi-Del	Chr03	7469812	7473078	Ach03g046101	PREDICTED: uncharacterized protein LOC100255280 [Vitis vinifera]
Chi-Set	Chr19	7819879	7824321	Ach19g399211	PREDICTED: uncharacterized protein LOC100257111 isoform X1 [Vitis vinifera]
Chi-Del	Chr12	11869785	11881900	Ach12g436041	PREDICTED: uncharacterized protein LOC100257683 isoform X1 [Vitis vinifera]
Chi-Set	Chr06	14093665	14093949	Ach06g448391	PREDICTED: uncharacterized protein LOC100258265 [Vitis vinifera]
Chi-Set	Chr01	16156359	16184475	Ach01g190881	PREDICTED: uncharacterized protein LOC100258878 [Vitis vinifera]
Chi-Set	Chr02	8234633	8236003	Ach02g312071	PREDICTED: uncharacterized protein LOC100259190 [Vitis vinifera]
Chi-Del	Chr05	682873	697050	Ach05g205161	PREDICTED: uncharacterized protein LOC100259729 isoform X1 [Vitis vinifera]
Chi-Del	Chr01	15680594	15693903	Ach01g135951	PREDICTED: uncharacterized protein LOC100260062 isoform X2 [Vitis vinifera]
Chi-Del	Chr05	678504	680577	Ach05g205281	PREDICTED: uncharacterized protein LOC100264919 isoform X2 [Vitis vinifera]
Chi-Set	Chr26	15596315	15607714	Ach26g239591	PREDICTED: uncharacterized protein LOC100265613 [Vitis vinifera]
Chi-Del	Chr05	17659800	17667885	Ach05g452441	PREDICTED: uncharacterized protein LOC100266005 [Vitis vinifera]
Chi-Set	Chr24	16969680	16981895	Ach24g312911	PREDICTED: uncharacterized protein LOC100854295 isoform X1 [Vitis vinifera]

Chi-Set	Chr15	11561687	11567302	Ach15g286031	PREDICTED: uncharacterized protein LOC100854533 [Vitis vinifera]
Chi-Del	Chr13	9069273	9072347	Ach13g352531	PREDICTED: uncharacterized protein LOC102586584 [Solanum tuberosum]
Chi-Set	Chr13	12551567	12552601	Ach13g190441	PREDICTED: uncharacterized protein LOC102595892 [Solanum tuberosum]
Chi-Set	Chr26	15072517	15086654	Ach26g179281	PREDICTED: uncharacterized protein LOC102622518 [Citrus sinensis]
Chi-Del	Chr10	8927900	8930525	Ach10g400511	PREDICTED: uncharacterized protein LOC102624759 isoform X3 [Citrus sinensis]
Chi-Del	Chr19	6319688	6323463	Ach19g382851	PREDICTED: uncharacterized protein LOC102625973 isoform X1 [Citrus sinensis]
Chi-Set	Chr20	3865977	3866828	Ach20g120751	PREDICTED: uncharacterized protein LOC103320297 [Prunus mume]
Chi-Set	Chr03	18930402	18951640	Ach03g218241	PREDICTED: uncharacterized protein LOC103323014 [Prunus mume]
Chi-Set	Chr09	10653612	10654604	Ach09g019831	PREDICTED: uncharacterized protein LOC103334643 [Prunus mume]
Chi-Set	Chr06	14095100	14098055	Ach06g448401	PREDICTED: uncharacterized protein LOC103411071 [Malus domestica]
Chi-Del	Chr23	17637624	17638805	Ach23g127431	PREDICTED: uncharacterized protein LOC103928682 [Pyrus x bretschneideri]
Chi-Del	Chr03	1174195	1174434	Ach03g358831	PREDICTED: uncharacterized protein LOC103983076 [Musa acuminata subsp. malaccensis]
Chi-Del	Chr24	2914350	2917182	Ach24g082861	PREDICTED: uncharacterized protein LOC104114273 [Nicotiana tomentosiformis]
Chi-Del	Chr10	13093784	13098408	Ach10g255181	PREDICTED: uncharacterized protein LOC104114922 [Nicotiana tomentosiformis]
Chi-Del	Chr15	2467662	2469972	Ach15g412551	PREDICTED: uncharacterized protein LOC104239101 [Nicotiana sylvestris]
Chi-Del	Chr05	17658537	17658878	Ach05g452431	PREDICTED: uncharacterized protein LOC104417910 [Eucalyptus grandis]
Chi-Del	Chr02	11078195	11078536	Ach02g293941	PREDICTED: uncharacterized protein LOC104421839 [Eucalyptus grandis]
Chi-Set	Chr13	12543247	12549047	Ach13g190451	PREDICTED: uncharacterized protein LOC104597551 [Nelumbo nucifera]
Chi-Set	Chr16	11257989	11265995	Ach16g443201	PREDICTED: uncharacterized protein LOC104599198 isoform X2 [Nelumbo nucifera]
Chi-Set	Chr13	12528944	12536686	Ach13g190231	PREDICTED: uncharacterized protein LOC104603526 isoform X1 [Nelumbo nucifera]

Chi-Del	Chr25	6191492	6200808	Ach25g373771	PREDICTED: uncharacterized protein LOC104882088 isoform X1 [Vitis vinifera]
Chi-Set	Chr12	8675898	8677822	Ach12g192211	PREDICTED: uncharacterized protein LOC105127630 [Populus euphratica]
Chi-Set	Chr07	17525738	17534113	Ach07g179571	PREDICTED: uncharacterized protein LOC105133752 isoform X2 [Populus euphratica]
Chi-Set	Chr14	161451	162560	Ach14g375261	PREDICTED: uncharacterized protein LOC105157495 [Sesamum indicum]
Chi-Del	Chr05	6750824	6757762	Ach05g198111	PREDICTED: uncharacterized protein LOC105168755 isoform X3 [Sesamum indicum]
Chi-Set	Chr01	11970941	11980000	Ach01g437451	PREDICTED: uncharacterized protein LOC105643225 [Jatropha curcas]
Chi-Del	Chr13	5855994	5860753	Ach13g146761	PREDICTED: uncharacterized protein LOC105962165 [Erythranthe guttatus]
Chi-Del	Chr07	6300100	6307158	Ach07g437011	PREDICTED: uridine 5'-monophosphate synthase [Sesamum indicum]
Chi-Set	Chr06	11958185	11973156	Ach06g453491	PREDICTED: vacuolar protein sorting-associated protein 52 A [Jatropha curcas]
Chi-Set	Chr08	20953226	20967897	Ach08g425581	PREDICTED: vacuolar protein sorting-associated protein 8 homolog isoform X1 [Vitis vinifera]
Chi-Set	Chr08	20950354	20953167	Ach08g104721	PREDICTED: vacuolar protein sorting-associated protein 8 homolog isoform X2 [Vitis vinifera]
Chi-Del	Chr25	13710151	13716887	Ach25g187131	PREDICTED: vesicle-associated membrane protein 711 [Vitis vinifera]
Chi-Del	Chr08	5509053	5550318	Ach08g273451	PREDICTED: vicianin hydrolase-like isoform X2 [Prunus mume]
Chi-Del	Chr03	1160777	1164644	Ach03g358761	PREDICTED: WD repeat-containing protein YMR102C [Vitis vinifera]
Chi-Del	Chr06	13802174	13805775	Ach06g247151	PREDICTED: WD-40 repeat-containing protein MSI1 [Sesamum indicum]
Chi-Del	Chr29	405410	408034	Ach29g164641	PREDICTED: zinc finger BED domain-containing protein DAYSLEEPER-like [Nicotiana sylvestris]
Chi-Del	Chr13	5861771	5878510	Ach13g146491	PREDICTED: zinc finger CCCH domain-containing protein 37 isoform X1 [Nelumbo nucifera]
Chi-Del	Chr23	12988975	12996280	Ach23g347651	PREDICTED: zinc finger CCCH domain-containing protein 44-like [Sesamum indicum]

Chi-Del	Chr03	20169406	20181679	Ach03g185751	PREDICTED: zinc finger CCCH domain-containing protein 58-like [Solanum tuberosum]
Chi-Del	Chr17	362729	373837	Ach17g251351	Protein CHMP7, putative [Ricinus communis]
Chi-Set	Chr15	2845656	2849354	Ach15g314441	protein kinase, putative [Ricinus communis]
Chi-Del	Chr01	15696317	15697685	Ach01g135931	Pti1-like kinase [Medicago truncatula]
Chi-Del	Chr28	688586	691531	Ach28g194991	Pto-interacting 1 [Gossypium arboreum]
Chi-Del	Chr26	8392556	8402730	Ach26g456941	putative inactive purple acid phosphatase 27 [Morus notabilis]
Chi-Del	Chr15	2471040	2474520	Ach15g380541	putative phosphoenolpyruvate enolase [Arabidopsis thaliana]
Chi-Del	Chr17	319836	322667	Ach17g251301	Putative polygalacturonase [Glycine soja]
Chi-Del	Chr01	3917287	3941752	Ach01g023451	RecName: Full=Cycloartenol Synthase [Panax ginseng]
Chi-Del	Chr15	7016799	7017182	Ach15g002421	RecName: Full=Tubulin beta-2 chain; AltName: Full=Beta-2-tubulin [Eleusine indica]
Chi-Del	Chr15	8924928	8934527	Ach15g154211	RING/U-box domain-containing protein, putative isoform 1 [Theobroma cacao]
Chi-Del	Chr07	11454866	11455408	Ach07g110641	Serine/threonine-protein kinase WNK-related [Theobroma cacao]
Chi-Set	Chr23	13945078	13968517	Ach23g347031	Serine/threonine-protein phosphatase BSL3 -like protein [Gossypium arboreum]
Chi-Del	Chr15	7110457	7114988	Ach15g001881	Silencing defective 5, putative isoform 2, partial [Theobroma cacao]
Chi-Del	Chr15	6713107	6719952	Ach15g002551	SOC1a [Actinidia chinensis]
Chi-Set	Chr04	495493	502999	Ach04g204921	SPla/RYanodine receptor domain-containing protein isoform 2 [Theobroma cacao]
Chi-Del	Chr04	9722800	9725333	Ach04g454251	Tetratricopeptide repeat-like superfamily protein, putative [Theobroma cacao]
Chi-Del	Chr15	6947713	6948432	Ach15g001821	thaumatin-like protein [Actinidia deliciosa]
Chi-Del	Chr06	14378417	14386009	Ach06g288611	Thiamin biosynthesis protein, putative isoform 3 [Theobroma cacao]
Chi-Del	Chr07	11456609	11473305	Ach07g110201	Threonine--tRNA ligase [Morus notabilis]
Chi-Del	Chr03	1176753	1177539	Ach03g358781	thylakoid lumenal 17.4 kDa protein [Medicago truncatula]
Chi-Set	Chr14	135157	143138	Ach14g375381	Tonoplast monosaccharide transporter2 isoform 1 [Theobroma cacao]
Chi-Del	Chr15	6617002	6621640	Ach15g001651	TVP38/TMEM64 family membrane protein [Glycine soja]
Chi-Del	Chr15	8683771	8689815	Ach15g109621	type II peroxiredoxin 2 [Vitis vinifera]
Chi-Set	Chr07	17872970	17875072	Ach07g261391	ubiquitin [Riftia pachyptila]

Chi-Del	Chr12	5615245	5618490	Ach12g119991	Ubiquitin supergroup,Ribosomal protein L40e [Theobroma cacao]
Chi-Del	Chr29	9623062	9627078	Ach29g386501	ubiquitin-conjugating enzyme family protein [Populus trichocarpa]
Chi-Set	Chr06	11975678	11994057	Ach06g276421	Vps52 / Sac2 family isoform 2 [Theobroma cacao]
Chi-Set	Chr26	504242	511351	Ach26g297041	Zinc finger (Ran-binding) family protein, putative isoform 2 [Theobroma cacao]
Chi-Del	Chr11	4273465	4282936	Ach11g277271	zinc finger protein, putative [Ricinus communis]
Del-Set	Chr15	364261	371226	Ach15g184361	26S proteasome non-ATPase regulatory subunit RPN12A [Morus notabilis]
Del-Set	Chr07	13643129	13644531	Ach07g391441	actin family protein [Populus trichocarpa]
Del-Set	Chr22	9859254	9866591	Ach22g135791	Ankyrin repeat family protein isoform 1 [Theobroma cacao]
Del-Set	Chr14	7486561	7487865	Ach14g313661	Basic helix-loop-helix DNA-binding family protein [Theobroma cacao]
Chi-Del	Chr27	8852818	8855608	Ach27g297271	chloroplast carbonic anhydrase [Pachysandra terminalis]
Del-Set	Chr22	8761048	8767375	Ach22g016021	cyclin-dependent kinase A1 [Scutellaria baicalensis]
Del-Set	Chr25	3946381	3946821	Ach25g316741	DNA binding protein, putative [Ricinus communis]
Chi-Del	Chr15	421952	425821	Ach15g184401	Heat shock protein 70 (Hsp 70) family protein [Theobroma cacao]
Del-Set	Chr08	11437270	11444044	Ach08g237881	hydroxysteroid dehydrogenase, putative [Ricinus communis]
Chi-Del	Chr27	7147202	7149091	Ach27g338951	hypothetical protein AMTR_s00184p00023820 [Amborella trichopoda]
Del-Set	Chr27	388790	391582	Ach27g451361	hypothetical protein CICLE_v10031922mg [Citrus clementina]
Del-Set	Chr26	3293966	3309857	Ach26g253451	hypothetical protein CISIN_1g003233mg [Citrus sinensis]
Chi-Del	Chr15	426934	427485	Ach15g184701	hypothetical protein CISIN_1g0095371mg, partial [Citrus sinensis]
Chi-Del	Chr15	1494056	1499149	Ach15g005671	hypothetical protein CISIN_1g019125mg [Citrus sinensis]
Chi-Del	Chr27	7140498	7145822	Ach27g339151	hypothetical protein CISIN_1g026871mg [Citrus sinensis]
Chi-Del	Chr17	646021	655781	Ach17g064881	hypothetical protein Csa_3G536140 [Cucumis sativus]
Del-Set	Chr27	742691	746699	Ach27g451391	hypothetical protein Csa_4G614180 [Cucumis sativus]

Del-Set	Chr22	9917603	9922059	Ach22g135841	hypothetical protein L484_024485 [Morus notabilis]
Chi-Del	Chr01	10853333	10855759	Ach01g088221	hypothetical protein L484_025595 [Morus notabilis]
Del-Set	Chr18	12340637	12341064	Ach18g256931	hypothetical protein OsI_37074 [Oryza sativa Indica Group]
Chi-Del	Chr24	9743852	9773643	Ach24g202501	hypothetical protein POPTR_0001s44980g [Populus trichocarpa]
Chi-Del	Chr15	1478103	1481968	Ach15g005681	hypothetical protein POPTR_0003s13270g [Populus trichocarpa]
Del-Set	Chr25	1225090	1238850	Ach25g098771	hypothetical protein POPTR_0005s09700g [Populus trichocarpa]
Del-Set	Chr08	11160674	11161276	Ach08g237801	hypothetical protein PRUPE_ppa001251mg [Prunus persica]
Del-Set	Chr27	57492	64325	Ach27g271391	hypothetical protein PRUPE_ppa003330mg [Prunus persica]
Del-Set	Chr23	20495	27831	Ach23g070191	hypothetical protein SOVF_009100, partial [Spinacia oleracea]
Chi-Del	Chr13	13130442	13134135	Ach13g349981	hypothetical protein SOVF_077850 [Spinacia oleracea]
Del-Set	Chr20	11099887	11107567	Ach20g467971	hypothetical protein VITISV_015444 [Vitis vinifera]
Del-Set	Chr22	10033597	10049242	Ach22g135761	isopentenyl diphosphate isomerase [Actinidia arguta]
Chi-Del	Chr02	6967396	6983439	Ach02g168621	Nuclear transport factor 2 family protein with RNA binding domain isoform 1 [Theobroma cacao]
Del-Set	Chr10	15345833	15349481	Ach10g168421	PDR-type ACB transporter [Nicotiana benthamiana]
Chi-Del	Chr27	7149117	7149774	Ach27g463171	phosphoenolpyruvate carboxykinase, partial [Mitella kiusiana]
Del-Set	Chr14	7062002	7081665	Ach14g278281	phragmoplastin [Camellia sinensis]
Del-Set	Chr22	8846412	8851047	Ach22g015991	PREDICTED: 22.0 kDa class IV heat shock protein [Jatropha curcas]
Chi-Del	Chr24	13443743	13445952	Ach24g319321	PREDICTED: ankyrin repeat-containing protein At2g01680-like [Citrus sinensis]
Chi-Del	Chr15	649642	650573	Ach15g438091	PREDICTED: arabinogalactan peptide 20-like [Nelumbo nucifera]
Chi-Del	Chr16	447896	453410	Ach16g213731	PREDICTED: beta-glucosidase 44-like [Citrus sinensis]
Del-Set	Chr01	8637579	8642715	Ach01g166991	PREDICTED: CBS domain-containing protein CBSCBSPB3 isoform X1 [Jatropha curcas]

Del-Set	Chr22	2624956	2639512	Ach22g286451	PREDICTED: cell division cycle and apoptosis regulator protein 1 isoform X2 [Sesamum indicum]
Chi-Del	Chr25	6609209	6628982	Ach25g374251	PREDICTED: cysteine--tRNA ligase, cytoplasmic-like [Erythranthe guttatus]
Del-Set	Chr20	10307419	10307856	Ach20g447641	PREDICTED: DNA ligase 4 isoform X2 [Vitis vinifera]
Del-Set	Chr26	1117701	1118369	Ach26g297121	PREDICTED: extra-large guanine nucleotide-binding protein 3-like [Jatropha curcas]
Chi-Del	Chr27	8878734	8883236	Ach27g297291	PREDICTED: GDSL esterase/lipase At3g27950 [Vitis vinifera]
Del-Set	Chr10	5834676	5857464	Ach10g187671	PREDICTED: glycerol-3-phosphate acyltransferase, chloroplastic-like [Malus domestica]
Del-Set	Chr25	2327872	2331261	Ach25g137301	PREDICTED: histidine--tRNA ligase, cytoplasmic [Jatropha curcas]
Chi-Del	Chr10	13928857	13939420	Ach10g163301	PREDICTED: histone-lysine N-methyltransferase ASHR3 isoform X2 [Nelumbo nucifera]
Chi-Del	Chr15	555862	556609	Ach15g184681	PREDICTED: hydroquinone glucosyltransferase-like [Nelumbo nucifera]
Chi-Del	Chr27	8870928	8877214	Ach27g297281	PREDICTED: L-aspartate oxidase, chloroplastic [Vitis vinifera]
Chi-Del	Chr24	3493811	3503080	Ach24g460301	PREDICTED: long chain acyl-CoA synthetase 6, peroxisomal [Vitis vinifera]
Chi-Del	Chr28	8618192	8622394	Ach28g409531	PREDICTED: LOW QUALITY PROTEIN: uncharacterized protein LOC100242929 [Vitis vinifera]
Chi-Del	Chr24	9818693	9824436	Ach24g204021	PREDICTED: methyltransferase-like protein 1 [Vitis vinifera]
Chi-Del	Chr02	6959260	6964837	Ach02g168851	PREDICTED: mitochondrial import receptor subunit TOM9-2-like [Sesamum indicum]
Del-Set	Chr13	6594048	6601402	Ach13g195331	PREDICTED: mitogen-activated protein kinase homolog NTF6 [Vitis vinifera]
Chi-Del	Chr01	10847846	10849713	Ach01g088131	PREDICTED: pectinesterase 2 [Vitis vinifera]
Del-Set	Chr04	522613	525108	Ach04g204891	PREDICTED: pentatricopeptide repeat-containing protein At4g01570 [Vitis vinifera]
Chi-Del	Chr28	8430896	8437102	Ach28g304091	PREDICTED: peroxisomal membrane protein 13 [Glycine max]
Del-Set	Chr25	3946957	3949605	Ach25g316731	PREDICTED: PHD finger protein MALE STERILITY 1 [Vitis vinifera]

Chi-Del	Chr27	8844190	8849619	Ach27g297261	PREDICTED: phosphoglucan phosphatase LSF1, chloroplastic isoform X1 [Vitis vinifera]
Chi-Del	Chr22	5128168	5142180	Ach22g285641	PREDICTED: phosphoglucomutase, cytoplasmic-like [Citrus sinensis]
Chi-Del	Chr28	8427417	8427935	Ach28g303021	PREDICTED: probable ATP-dependent RNA helicase DDX11 [Gossypium raimondii]
Del-Set	Chr18	10432534	10440403	Ach18g229791	PREDICTED: probable E3 ubiquitin-protein ligase XBOS32 [Nicotiana tomentosiformis]
Del-Set	Chr08	12172152	12220780	Ach08g238121	PREDICTED: probable isoleucine--tRNA ligase, cytoplasmic [Nicotiana sylvestris]
Chi-Del	Chr24	9803769	9815992	Ach24g204031	PREDICTED: probable lysine-specific demethylase ELF6 isoform X1 [Vitis vinifera]
Chi-Del	Chr15	519107	523530	Ach15g184441	PREDICTED: probable polygalacturonase [Nelumbo nucifera]
Del-Set	Chr22	8796557	8801236	Ach22g016031	PREDICTED: probable protein phosphatase 2C 33 [Jatropha curcas]
Del-Set	Chr27	126413	133638	Ach27g271451	PREDICTED: probable protein S-acyltransferase 22 [Vitis vinifera]
Chi-Del	Chr15	536730	538857	Ach15g184451	PREDICTED: probable WRKY transcription factor 41 [Populus euphratica]
Del-Set	Chr14	7185113	7186723	Ach14g278241	PREDICTED: protein CURVATURE THYLAKOID 1B, chloroplastic [Jatropha curcas]
Del-Set	Chr18	9302351	9302851	Ach18g481341	PREDICTED: protein GLUTAMINE DUMPER 3 [Pyrus x bretschneideri]
Del-Set	Chr10	7276857	7277336	Ach10g447821	PREDICTED: protein GLUTAMINE DUMPER 3 [Vitis vinifera]
Chi-Del	Chr15	8800703	8817788	Ach15g364771	PREDICTED: protein IQ-DOMAIN 1-like [Nicotiana sylvestris]
Chi-Del	Chr15	402017	407022	Ach15g184391	PREDICTED: protein NLP7 isoform X1 [Sesamum indicum]
Chi-Del	Chr15	1484068	1492201	Ach15g005641	PREDICTED: protein SGT1 homolog A-like [Jatropha curcas]
Chi-Del	Chr15	558315	563130	Ach15g184671	PREDICTED: protein trichome birefringence-like 23 [Solanum lycopersicum]
Del-Set	Chr25	2289860	2301883	Ach25g431531	PREDICTED: putative E3 ubiquitin-protein ligase RING1a [Vitis vinifera]
Del-Set	Chr04	545802	566110	Ach04g204871	PREDICTED: putative methyltransferase At1g22800 isoform X2 [Gossypium raimondii]

Chi-Del	Chr02	11275286	11285404	Ach02g393561	PREDICTED: putative nuclear matrix constituent protein 1-like protein isoform X2 [Vitis vinifera]
Del-Set	Chr22	9178484	9196264	Ach22g015931	PREDICTED: putative RNA-binding protein Luc7-like 2 isoform X2 [Nelumbo nucifera]
Chi-Del	Chr28	8626267	8630731	Ach28g409521	PREDICTED: rhodanese-like domain-containing protein 4A, chloroplastic [Vitis vinifera]
Chi-Del	Chr03	11746519	11746899	Ach03g139091	PREDICTED: serine/threonine protein phosphatase 2A 57 kDa regulatory subunit B' beta isoform-like [Populus euphratica]
Del-Set	Chr08	11182922	11183386	Ach08g237811	PREDICTED: sphinganine C(4)-monooxygenase 1-like isoform X2 [Camelina sativa]
Chi-Del	Chr01	10356308	10359674	Ach01g088391	PREDICTED: transcription factor bHLH18-like [Sesamum indicum]
Del-Set	Chr27	552610	555921	Ach27g258171	PREDICTED: transcription termination factor 2 isoform X4 [Jatropha curcas]
Chi-Del	Chr24	13446124	13459528	Ach24g406981	PREDICTED: U6 snRNA phosphodiesterase [Jatropha curcas]
Chi-Del	Chr28	8386679	8410225	Ach28g303041	PREDICTED: ubiquitin carboxyl-terminal hydrolase 5 [Vitis vinifera]
Chi-Del	Chr13	13124755	13126702	Ach13g350371	PREDICTED: uncharacterized protein LOC100255634 [Vitis vinifera]
Chi-Del	Chr17	637413	640128	Ach17g420501	PREDICTED: uncharacterized protein LOC100258835 isoform X2 [Vitis vinifera]
Chi-Del	Chr10	9570196	9586322	Ach10g145851	PREDICTED: uncharacterized protein LOC100262718 isoform X2 [Vitis vinifera]
Chi-Del	Chr28	8507389	8510548	Ach28g409581	PREDICTED: uncharacterized protein LOC100852824 [Vitis vinifera]
Chi-Del	Chr15	539794	545217	Ach15g438111	PREDICTED: uncharacterized protein LOC101210365 isoform X2 [Cucumis sativus]
Chi-Del	Chr28	8411856	8425616	Ach28g303031	PREDICTED: uncharacterized protein LOC101247846 [Solanum lycopersicum]
Del-Set	Chr01	8935709	8936602	Ach01g167061	PREDICTED: uncharacterized protein LOC103339118 [Prunus mume]
Chi-Del	Chr03	11985638	11987439	Ach03g153651	PREDICTED: uncharacterized protein LOC103432448 [Malus domestica]
Del-Set	Chr08	11843222	11851122	Ach08g238001	PREDICTED: uncharacterized protein LOC103432724 isoform X1 [Malus domestica]
Del-Set	Chr28	5438448	5439535	Ach28g321311	PREDICTED: uncharacterized protein LOC103503220 [Cucumis melo]

Chi-Del	Chr03	11705797	11706174	Ach03g138811	PREDICTED: uncharacterized protein LOC103936350 [Pyrus x bretschneideri]
Del-Set	Chr20	10459269	10459634	Ach20g368961	PREDICTED: uncharacterized protein LOC104718701 isoform X1 [Camelina sativa]
Del-Set	Chr08	11404292	11411492	Ach08g237871	PREDICTED: uncharacterized protein LOC104879471 [Vitis vinifera]
Chi-Del	Chr03	11715966	11717302	Ach03g431321	PREDICTED: uncharacterized protein LOC104881124 [Vitis vinifera]
Chi-Del	Chr15	646822	647106	Ach15g184521	PREDICTED: uncharacterized protein LOC104881234 [Vitis vinifera]
Chi-Del	Chr15	376018	384751	Ach15g184721	PREDICTED: uncharacterized protein LOC104881368 isoform X4 [Vitis vinifera]
Del-Set	Chr02	8314075	8320020	Ach02g459481	PREDICTED: uncharacterized protein LOC105131470 [Populus euphratica]
Chi-Del	Chr27	8856741	8863358	Ach27g456831	PREDICTED: uncharacterized protein LOC105167711 isoform X2 [Sesamum indicum]
Chi-Del	Chr17	633939	634298	Ach17g064741	PREDICTED: uncharacterized protein LOC105956476 [Erythranthe guttatus]
Del-Set	Chr01	8933702	8934694	Ach01g167021	PREDICTED: uncharacterized protein LOC105972900 [Erythranthe guttatus]
Del-Set	Chr08	11859092	11875951	Ach08g402711	PREDICTED: zinc finger BED domain-containing protein RICESLEEPER 1-like [Populus euphratica]
Del-Set	Chr01	8922787	8927534	Ach01g167011	PREDICTED: zinc finger CCCH domain-containing protein 16 isoform X2 [Vitis vinifera]
Chi-Del	Chr15	437486	440246	Ach15g184411	putative phosphatidylinositol 4-kinase type 2-beta [Morus notabilis]
Del-Set	Chr22	2408331	2410278	Ach22g286481	RING/FYVE/PHD zinc finger superfamily protein isoform 1 [Theobroma cacao]
Del-Set	Chr18	12058931	12062123	Ach18g449791	trehalose-6-phosphate synthase [Camellia sinensis]
Chi-Del	Chr27	7155021	7170524	Ach27g339161	tRNA-dihydrouridine synthase, putative [Ricinus communis]
Chi-Del	Chr15	657446	672233	Ach15g184611	ubiquitin-conjugating enzyme E2 [Camellia sinensis]

Table S7. Population genetic summary statistics. Mean (±standard deviation) values of nucleotide diversity π, Tajima's D statistic, pairwise relative measure of differentiation (FST) and absolute divergence (Dxy) between all comparisons.

Comparisons	Groups	Populations	No. of individuals	$\pi(\times10^{-3})$	Tajima's D	F_{ST}	$Dxy(10^{-3})$
sea isolation	oceanic island	ZSC+TWS	13+14	1.02±0.459	2.122±0.710		
	mainland	TPC+YAC+SYC +JZD+ZLD	6+6+6+6+6	1.082±0.529	0.267±0.536	0.214±0.069	0.335±0.075
lake isolation	island	JSC	13	0.652±0.408	0.859±0.815		
	neighbor land	YMC	14	0.653±0.409	0.906±0.838	0.081±0.066	0.656±0.342

Comparisons	Groups	Populations	No. of individuals	$\pi(\times10^{-3})$	Tajima's D	Fst/Dxy($\times10^{-3}$)			
						TPC	SYC	YAC	YMC
geographic isolation	north	TPC	15	0.755±0.432	0.899±0.758	——	0.640±0.338	0.714±0.372	0.725±0.310
	west	SYC	13	0.597±0.381	0.942±0.806	0.144±0.091	——	0.821±0.425	0.865±0.361
	south	YAC	12	0.573±0.375	1.088±0.934	0.211±0.114	0.254±0.132	——	0.933±0.390
	east	YMC	14	0.653±0.409	0.906±0.838	0.292±0.149	0.357±0.170	0.389±0.163	——

Table S8. Genes in genomic islands of oceanic and lake isolation under strong selective sweeps

isolation	region	BIN_START	END	GeneID	anno
ocean	inland	327742	337835	Ach04g204991	invertase 3 [Camellia sinensis]
ocean	inland	12188884	12189399	Ach07g432251	PREDICTED: mediator of RNA polymerase II transcription subunit 27 [Jatropha curcas]
ocean	inland	388790	391582	Ach27g451361	hypothetical protein CICLE_v10031922mg [Citrus clementina]
ocean	inland	725438	736796	Ach27g258201	PREDICTED: transcription factor DIVARICATA [Sesamum indicum]
ocean	inland	2509660	2511275	Ach22g286511	hypothetical protein VITISV_002957 [Vitis vinifera]
ocean	inland	2624956	2639512	Ach22g286451	PREDICTED: cell division cycle and apoptosis regulator protein 1 isoform X2 [Sesamum indicum]
ocean	inland	2530095	2534800	Ach22g455431	PREDICTED: uncharacterized protein LOC100796928 isoform X1 [Glycine max]
ocean	inland	522613	525108	Ach04g204891	PREDICTED: pentatricopeptide repeat-containing protein At4g01570 [Vitis vinifera]
ocean	inland	12215453	12215812	Ach07g139491	PREDICTED: uncharacterized protein LOC103503220 [Cucumis melo]
ocean	inland	2477753	2478205	Ach04g242941	conserved hypothetical protein [Ricinus communis]
ocean	inland	3288636	3290991	Ach04g057781	sucrose transporter 4 [Camellia sinensis]
ocean	inland	7344897	7350938	Ach01g285151	PERK1-like protein kinase [Nicotiana tabacum]
ocean	inland	6924436	6925681	Ach01g308441	PREDICTED: F-box protein At5g07610-like [Nicotiana sylvestris]
ocean	inland	9714200	9715611	Ach05g107181	ACT1 [Actinidia deliciosa]
ocean	inland	2868813	2871015	Ach04g057641	AP-1 complex subunit sigma-1 [Glycine soja]
ocean	inland	7214368	7221235	Ach06g103931	CAS1 domain-containing 1 [Gossypium arboreum]
ocean	inland	12548105	12571638	Ach07g391211	E3 ubiquitin-protein ligase RKP [Morus notabilis]
ocean	inland	16421834	16424660	Ach05g207361	ferulate 5-hydroxylase [Camptotheca acuminata]
ocean	inland	7423432	7426348	Ach01g285361	fibronectin type III domain-containing family protein [Populus trichocarpa]
ocean	inland	3009757	3013554	Ach04g057621	forkhead-associated domain-containing family protein [Populus trichocarpa]
ocean	inland	11388003	11389300	Ach08g237861	GRAS family transcription factor SHR2 [Quercus suber]
ocean	inland	11926784	11928210	Ach07g432221	hypothetical protein B456_007G243100 [Gossypium raimondii]
ocean	inland	2370106	2392469	Ach04g242971	hypothetical protein CICLE_v10000028mg [Citrus clementina]
ocean	inland	6719980	6739310	Ach11g262871	hypothetical protein CICLE_v10001133mg [Citrus clementina]
ocean	inland	8768001	8781344	Ach01g167091	hypothetical protein CICLE_v10025298mg [Citrus clementina]

ocean	inland	7279200	7281118	Ach01g285311	hypothetical protein EUGRSUZ_C01032 [Eucalyptus grandis]
ocean	inland	9699217	9707919	Ach05g107341	hypothetical protein L484_012192 [Morus notabilis]
ocean	inland	9376943	9380453	Ach05g107271	hypothetical protein L484_020727 [Morus notabilis]
ocean	inland	414757	422596	Ach04g441011	hypothetical protein L484_025447 [Morus notabilis]
ocean	inland	5649186	5658629	Ach10g306521	hypothetical protein MIMGU_mgv1a0017942mg, partial [Erythranthe guttata]
ocean	inland	2594885	2601796	Ach04g242931	hypothetical protein MIMGU_mgv1a004528mg [Erythranthe guttata]
ocean	inland	9442126	9447426	Ach05g107231	hypothetical protein MIMGU_mgv1a025126mg [Erythranthe guttata]
ocean	inland	10445408	10461730	Ach04g048921	hypothetical protein PHAVU_009G025800g [Phaseolus vulgaris]
ocean	inland	9602078	9605396	Ach18g052001	hypothetical protein POPTR_0001s10240g [Populus trichocarpa]
ocean	inland	6691511	6692137	Ach11g262891	hypothetical protein POPTR_0005s15990g [Populus trichocarpa]
ocean	inland	8931725	8947301	Ach24g442521	hypothetical protein POPTR_0008s02980g [Populus trichocarpa]
ocean	inland	11132178	11133157	Ach20g369111	hypothetical protein POPTR_0009s16850g, partial [Populus trichocarpa]
ocean	inland	129169	129471	Ach04g456781	hypothetical protein POPTR_0013s12040g [Populus trichocarpa]
ocean	inland	14299780	14305033	Ach11g475841	hypothetical protein POPTR_0017s06650g [Populus trichocarpa]
ocean	inland	477802	484443	Ach04g205031	hypothetical protein POPTR_0229s00250g [Populus trichocarpa]
ocean	inland	14305307	14308052	Ach11g050431	hypothetical protein PRUPE_ppa001233mg [Prunus persica]
ocean	inland	6686012	6686902	Ach11g262731	hypothetical protein PRUPE_ppa005521mg [Prunus persica]
ocean	inland	83552	88022	Ach23g226171	hypothetical protein PRUPE_ppa016079mg [Prunus persica]
ocean	inland	13351466	13354454	Ach05g247611	hypothetical protein RCOM_1278130 [Ricinus communis]
ocean	inland	8877082	8877396	Ach22g016051	hypothetical protein SORBIDRAFT_01g050160 [Sorghum bicolor]
ocean	inland	20495	27831	Ach23g070191	hypothetical protein SOVF_009100, partial [Spinacia oleracea]
ocean	inland	6124266	6124718	Ach24g443891	hypothetical protein VITISV_008130 [Vitis vinifera]
ocean	inland	9816481	9817593	Ach01g446051	hypothetical protein VITISV_015002 [Vitis vinifera]
ocean	inland	2602770	2605105	Ach04g242981	hypothetical protein VITISV_020704 [Vitis vinifera]
ocean	inland	10540727	10544762	Ach17g253141	hypothetical protein VITISV_024322 [Vitis vinifera]
ocean	inland	9817936	9818346	Ach01g446061	hypothetical protein VITISV_026939 [Vitis vinifera]

ocean	inland	508581	508868	Ach04g204911	hypothetical protein VITISV_028502 [Vitis vinifera]
ocean	inland	6124849	6125842	Ach24g216171	hypothetical protein VITISV_029152 [Vitis vinifera]
ocean	inland	122358	122687	Ach04g282821	hypothetical protein VITISV_035137 [Vitis vinifera]
ocean	inland	12549243	12553062	Ach05g047721	hypothetical protein VITISV_039453 [Vitis vinifera]
ocean	inland	6126121	6126756	Ach24g443881	hypothetical protein VITISV_043921 [Vitis vinifera]
ocean	inland	12145455	12150880	Ach08g238101	inositol transporter 1 [Camellia sinensis]
ocean	inland	410170	411403	Ach04g204961	MYB1 [Actinidia chinensis]
ocean	inland	10041038	10045944	Ach27g234721	oxidoreductase, putative [Ricinus communis]
ocean	inland	374941	376374	Ach04g205011	Patatin T5 precursor, putative [Ricinus communis]
ocean	inland	7437194	7444506	Ach01g285371	PREDICTED: 3-hydroxyisobutyryl-CoA hydrolase-like protein 3, mitochondrial isoform X1 [Vitis vinifera]
ocean	inland	13599000	13599962	Ach07g000451	PREDICTED: 54S ribosomal protein L10, mitochondrial isoform X1 [Vitis vinifera]
ocean	inland	9606370	9607854	Ach18g052011	PREDICTED: 6-phosphogluconate dehydrogenase, decarboxylating 1, chloroplastic [Vitis vinifera]
ocean	inland	127127	143083	Ach23g226121	PREDICTED: 7-dehydrocholesterol reductase-like [Malus domestica]
ocean	inland	9599095	9600927	Ach18g051991	PREDICTED: ABC transporter C family member 10-like [Vitis vinifera]
ocean	inland	7020972	7027286	Ach05g193501	PREDICTED: acyltransferase-like protein At1g54570, chloroplastic isoform X2 [Vitis vinifera]
ocean	inland	7006215	7017444	Ach05g193491	PREDICTED: acyltransferase-like protein At3g26840, chloroplastic [Vitis vinifera]
ocean	inland	2620410	2628976	Ach04g242911	PREDICTED: alpha-(1,4)-fucosyltransferase [Vitis vinifera]
ocean	inland	10560195	10561496	Ach17g253131	PREDICTED: alpha-1,6-mannosyl-glycoprotein 2-beta-N-acetylglucosaminyltransferase [Vitis vinifera]
ocean	inland	10569153	10570628	Ach10g270151	PREDICTED: aspartic proteinase nepenthesin-1 [Vitis vinifera]
ocean	inland	8896565	8902925	Ach22g015971	PREDICTED: aspartic proteinase-like protein 2 [Solanum lycopersicum]
ocean	inland	8880909	8887395	Ach22g015981	PREDICTED: aspartic proteinase-like protein 2, partial [Malus domestica]
ocean	inland	9608567	9617386	Ach05g107191	PREDICTED: autophagy-related protein 13 isoform X1 [Sesamum indicum]
ocean	inland	6696767	6702518	Ach11g262881	PREDICTED: auxin response factor 17 [Jatropha curcas]
ocean	inland	14093067	14095675	Ach10g418461	PREDICTED: bifunctional purine biosynthesis protein purH-like [Glycine max]
ocean	inland	38620	67094	Ach07g341101	PREDICTED: brefeldin A-inhibited guanine nucleotide-exchange protein 1 [Nicotiana sylvestris]
ocean	inland	11624782	11653554	Ach22g059741	PREDICTED: bromodomain and WD repeat-containing protein 3 isoform X1 [Vitis vinifera]

ocean	inland	9613789	9620407	Ach18g052381	PREDICTED: BTB/POZ domain-containing protein POB1-like [Vitis vinifera]
ocean	inland	12106093	12108379	Ach08g238091	PREDICTED: calcium-dependent protein kinase 24 isoform X1 [Nicotiana tomentosiformis]
ocean	inland	7093598	7103005	Ach01g285281	PREDICTED: casein kinase I-like isoform X2 [Gossypium raimondii]
ocean	inland	13562682	13568770	Ach21g094961	PREDICTED: catalase isozyme 3 [Nicotiana tomentosiformis]
ocean	inland	8637579	8642715	Ach01g166991	PREDICTED: CBS domain-containing protein CBSCBSPB3 isoform X1 [Jatropha curcas]
ocean	inland	9237297	9241508	Ach29g413931	PREDICTED: CHD3-type chromatin-remodeling factor PICKLE [Nicotiana tomentosiformis]
ocean	inland	14086263	14088028	Ach10g418471	PREDICTED: cytochrome P450 76A2-like [Eucalyptus grandis]
ocean	inland	6660226	6671778	Ach11g393751	PREDICTED: DIS3-like exonuclease 2-like isoform X1 [Citrus sinensis]
ocean	inland	11533579	11561991	Ach05g047971	PREDICTED: DNA topoisomerase 3-alpha [Sesamum indicum]
ocean	inland	5622126	5622425	Ach10g458301	PREDICTED: dynamin-like protein ARC5 [Jatropha curcas]
ocean	inland	149955	160297	Ach23g226191	PREDICTED: E3 ubiquitin-protein ligase MARCH1 [Vitis vinifera]
ocean	inland	12520477	12544106	Ach07g000311	PREDICTED: E3 ubiquitin-protein ligase RKP [Vitis vinifera]
ocean	inland	6942643	6946567	Ach01g308461	PREDICTED: equilibrative nucleotide transporter 8 isoform X1 [Jatropha curcas]
ocean	inland	7394775	7395767	Ach01g285351	PREDICTED: glycine-rich cell wall structural protein-like [Eucalyptus grandis]
ocean	inland	44453	57596	Ach23g226151	PREDICTED: histone acetyltransferase HAC1-like [Vitis vinifera]
ocean	inland	10050811	10062531	Ach27g234731	PREDICTED: inositol hexakisphosphate and diphosphoinositol-pentakisphosphate kinase 2-like isoform X1 [Citrus sinensis]
ocean	inland	3444831	3448470	Ach04g057591	PREDICTED: leucine-rich repeat receptor-like tyrosine-protein kinase At2g41820 [Prunus mume]
ocean	inland	13577800	13607164	Ach21g095421	PREDICTED: LOW QUALITY PROTEIN: Niemann-Pick C1 protein [Vitis vinifera]
ocean	inland	6756745	6775759	Ach11g262851	PREDICTED: mediator of RNA polymerase II transcription subunit 1 [Vitis vinifera]
ocean	inland	11167885	11171748	Ach20g368881	PREDICTED: methyl-CpG-binding domain-containing protein 2 [Vitis vinifera]
ocean	inland	14320289	14360407	Ach11g050161	PREDICTED: midasin [Vitis vinifera]
ocean	inland	6128366	6136216	Ach24g216201	PREDICTED: mitochondrial acidic protein MAM33 [Vitis vinifera]

ocean	inland	16236472	16289681	Ach05g207281	PREDICTED: MORC family CW-type zinc finger protein 3 [Vitis vinifera]
ocean	inland	8953516	8954207	Ach24g202311	PREDICTED: myosin-9-like [Vitis vinifera]
ocean	inland	3518517	3519799	Ach04g057581	PREDICTED: NAC domain-containing protein 7-like isoform X1 [Glycine max]
ocean	inland	63888	73210	Ach23g226161	PREDICTED: organic cation/carnitine transporter 7 [Vitis vinifera]
ocean	inland	7296898	7297608	Ach01g285191	PREDICTED: pentatricopeptide repeat-containing protein At5g25630 [Nelumbo nucifera]
ocean	inland	13588229	13589119	Ach07g391421	PREDICTED: pre-mRNA-splicing factor ISY1 homolog [Beta vulgaris subsp. vulgaris]
ocean	inland	12110354	12136329	Ach08g237601	PREDICTED: probable E3 ubiquitin-protein ligase ARI8 [Nelumbo nucifera]
ocean	inland	7034738	7048112	Ach01g285271	PREDICTED: probable lactoylglutathione lyase, chloroplast [Vitis vinifera]
ocean	inland	9358460	9362621	Ach05g107311	PREDICTED: probable pectinesterase/pectinesterase inhibitor 51 [Nelumbo nucifera]
ocean	inland	9369099	9372056	Ach05g107321	PREDICTED: probable pectinesterase/pectinesterase inhibitor 51 [Vitis vinifera]
ocean	inland	607008	612188	Ach04g204861	PREDICTED: probable serine/threonine-protein kinase At1g09600-like [Solanum tuberosum]
ocean	inland	7204635	7207273	Ach06g428561	PREDICTED: probable WRKY transcription factor 71 [Vitis vinifera]
ocean	inland	9109544	9114935	Ach22g415551	PREDICTED: protease Do-like 10, mitochondrial [Setaria italica]
ocean	inland	14327091	14360580	Ach03g306941	PREDICTED: protease Do-like 7 isoform X1 [Vitis vinifera]
ocean	inland	344436	346818	Ach04g204981	PREDICTED: protein FAR1-RELATED SEQUENCE 5-like [Prunus mume]
ocean	inland	32245	37303	Ach07g340231	PREDICTED: protein FAR1-RELATED SEQUENCE 9 isoform X2 [Vitis vinifera]
ocean	inland	396673	396954	Ach04g441001	PREDICTED: protein FIZZY-RELATED 3 [Cucumis sativus]
ocean	inland	3697589	3725001	Ach18g102051	PREDICTED: protein MOR1-like [Vitis vinifera]
ocean	inland	13573052	13576728	Ach21g094971	PREDICTED: protein NLP4-like [Vitis vinifera]
ocean	inland	2846780	2848480	Ach04g057661	PREDICTED: protein ROOT PRIMORDIUM DEFECTIVE 1-like [Vitis vinifera]
ocean	inland	3128751	3130277	Ach04g057721	PREDICTED: protein SENSITIVE TO PROTON RHIZOTOXICITY 1 [Vitis vinifera]
ocean	inland	196158	206296	Ach04g456801	PREDICTED: putative 1-phosphatidylinositol-3-phosphate 5-kinase FAB1D [Vitis vinifera]
ocean	inland	2566998	2568562	Ach04g448251	PREDICTED: putative CCA tRNA nucleotidyltransferase 2 [Nicotiana sylvestris]

ocean	inland	2611098	2612384	Ach04g242921	PREDICTED: putative CCA tRNA nucleotidyltransferase 2 [Vitis vinifera]
ocean	inland	545802	566110	Ach04g204871	PREDICTED: putative methyltransferase At1g22800 isoform X2 [Gossypium raimondii]
ocean	inland	8464054	8464818	Ach22g439181	PREDICTED: putative pentatricopeptide repeat-containing protein At5g37570 [Vitis vinifera]
ocean	inland	510345	516636	Ach04g204901	PREDICTED: ran-binding protein 10-like [Jatropha curcas]
ocean	inland	12765866	12781922	Ach07g000341	PREDICTED: S-adenosylmethionine carrier 1, chloroplastic/mitochondrial isoform X2 [Cucumis melo]
ocean	inland	9988946	9996441	Ach07g110441	PREDICTED: serine/threonine-protein kinase BRI1-like 2 [Vitis vinifera]
ocean	inland	9577943	9581951	Ach05g107201	PREDICTED: serine/threonine-protein kinase ULK3-like isoform X2 [Solanum tuberosum]
ocean	inland	9641649	9656750	Ach15g285511	PREDICTED: serine/threonine-protein phosphatase PP2A catalytic subunit-like [Citrus sinensis]
ocean	inland	9813585	9816234	Ach01g229991	PREDICTED: SUMO-conjugating enzyme SCE1 isoform X2 [Populus euphratica]
ocean	inland	11931491	11931979	Ach07g139531	PREDICTED: THO complex subunit 4C-like [Nelumbo nucifera]
ocean	inland	14313892	14314188	Ach11g050421	PREDICTED: threonine synthase, chloroplastic [Sesamum indicum]
ocean	inland	14314225	14315460	Ach11g050411	PREDICTED: threonine synthase, chloroplastic-like [Nelumbo nucifera]
ocean	inland	10558937	10566102	Ach10g270311	PREDICTED: transmembrane protein 56-like [Vitis vinifera]
ocean	inland	9506924	9508156	Ach05g107211	PREDICTED: U-box domain-containing protein 27 [Vitis vinifera]
ocean	inland	3664003	3665931	Ach21g372201	PREDICTED: UDP-glycosyltransferase 85A5-like [Prunus mume]
ocean	inland	438896	443723	Ach04g204941	PREDICTED: uncharacterized protein At1g04910 isoform X2 [Solanum lycopersicum]
ocean	inland	7040100	7056283	Ach05g440101	PREDICTED: uncharacterized protein At2g33490 isoform X2 [Prunus mume]
ocean	inland	6116151	6122210	Ach24g443921	PREDICTED: uncharacterized protein At5g39865-like [Fragaria vesca subsp. vesca]
ocean	inland	377763	390165	Ach04g204971	PREDICTED: uncharacterized protein LOC100233118 [Vitis vinifera]
ocean	inland	453141	461487	Ach04g441021	PREDICTED: uncharacterized protein LOC100242657 isoform X1 [Vitis vinifera]
ocean	inland	12792673	12805286	Ach07g000231	PREDICTED: uncharacterized protein LOC100247348 isoform X3 [Vitis vinifera]

ocean	inland	12763658	12782529	Ach12g167761	PREDICTED: uncharacterized protein LOC100249222 isoform X1 [Vitis vinifera]
ocean	inland	105654	116600	Ach23g445191	PREDICTED: uncharacterized protein LOC100258138 isoform X2 [Vitis vinifera]
ocean	inland	8935709	8936602	Ach01g167061	PREDICTED: uncharacterized protein LOC103339118 [Prunus mume]
ocean	inland	11843222	11851122	Ach08g238001	PREDICTED: uncharacterized protein LOC103432724 isoform X1 [Malus domestica]
ocean	inland	3691470	3694479	Ach18g102061	PREDICTED: uncharacterized protein LOC104235748 [Nicotiana sylvestris]
ocean	inland	7979387	7981378	Ach01g284931	PREDICTED: uncharacterized protein LOC104436568 [Eucalyptus grandis]
ocean	inland	163815	165066	Ach23g226101	PREDICTED: uncharacterized protein LOC104609040 [Nelumbo nucifera]
ocean	inland	165371	165622	Ach23g445201	PREDICTED: uncharacterized protein LOC104609046 [Nelumbo nucifera]
ocean	inland	125769	126717	Ach04g456771	PREDICTED: uncharacterized protein LOC104742444 isoform X2 [Camelina sativa]
ocean	inland	6123777	6124184	Ach24g443901	PREDICTED: uncharacterized protein LOC104878497 [Vitis vinifera]
ocean	inland	3183923	3184624	Ach04g420301	PREDICTED: uncharacterized protein LOC104880586 isoform X1 [Vitis vinifera]
ocean	inland	6123253	6123741	Ach24g443911	PREDICTED: uncharacterized protein LOC104880786 isoform X1 [Vitis vinifera]
ocean	inland	298540	299689	Ach04g282881	PREDICTED: uncharacterized protein LOC105127877 [Populus euphratica]
ocean	inland	244273	246879	Ach04g282871	PREDICTED: uncharacterized protein LOC105158252 [Sesamum indicum]
ocean	inland	5999833	6018693	Ach27g469261	PREDICTED: uncharacterized protein LOC105177598 [Sesamum indicum]
ocean	inland	71705	72229	Ach07g410351	PREDICTED: uncharacterized protein LOC105635576 [Jatropha curcas]
ocean	inland	2889414	2914452	Ach04g057681	PREDICTED: uncharacterized protein LOC105637143 [Jatropha curcas]
ocean	inland	100791	105250	Ach23g226181	PREDICTED: uncharacterized protein LOC105646135 isoform X2 [Jatropha curcas]
ocean	inland	16454413	16459511	Ach05g402081	PREDICTED: uncharacterized protein LOC105965337 [Erythranthe guttatus]
ocean	inland	8933702	8934694	Ach01g167021	PREDICTED: uncharacterized protein LOC105972900 [Erythranthe guttatus]
ocean	inland	220675	221625	Ach04g456811	PREDICTED: uncharacterized protein LOC105977068 [Erythranthe guttatus]
ocean	inland	9451774	9457406	Ach05g426681	PREDICTED: uncharacterized protein LOC105977276 [Erythranthe guttatus]

ocean	inland	11859092	11875951	Ach08g402711	PREDICTED: zinc finger BED domain-containing protein RICESLEEPER 1-like [Populus euphratica]
ocean	inland	8922787	8927534	Ach01g167011	PREDICTED: zinc finger CCCH domain-containing protein 16 isoform X2 [Vitis vinifera]
ocean	inland	7427292	7434783	Ach01g405511	Protein mrp [Gossypium arboreum]
ocean	inland	495493	502999	Ach04g204921	SPla/RYanodine receptor domain-containing protein isoform 2 [Theobroma cacao]
ocean	inland	2945652	2953937	Ach04g057701	sucrose transporter 3 [Camellia sinensis]
ocean	inland	3243124	3247851	Ach04g057771	sucrose transporter 6 [Camellia sinensis]
ocean	inland	8884342	8886995	Ach01g167071	transcription factor APETALA2 [Vitis vinifera]
ocean	inland	12058931	12062123	Ach18g449791	trehalose-6-phosphate synthase [Camellia sinensis]
ocean	island	5491547	5495725	Ach20g091981	PREDICTED: probable transcription factor KAN2 isoform X2 [Vitis vinifera]
ocean	island	639529	640782	Ach04g204841	3-phenylpropionate/cinnamic acid dioxygenase ferredoxin subunit [Gossypium arboreum]
ocean	island	13808773	13811174	Ach24g319621	60S ribosomal protein L15 [Populus trichocarpa]
ocean	island	18824309	18846012	Ach15g142891	Androgen induced inhibitor of proliferation / pds5 isoform 2 [Theobroma cacao]
ocean	island	3901206	3904635	Ach11g451831	BnaC02g00390D [Brassica napus]
ocean	island	10675490	10723329	Ach01g088261	hypothetical protein B456_003G091100 [Gossypium raimondii]
ocean	island	5733583	5738769	Ach19g209761	hypothetical protein EUGRSUZ_H00613 [Eucalyptus grandis]
ocean	island	1472827	1474889	Ach13g308671	hypothetical protein L484_025043 [Morus notabilis]
ocean	island	1198930	1200855	Ach13g458761	hypothetical protein M569_02206 [Genlisea aurea]
ocean	island	9077872	9092819	Ach11g263361	hypothetical protein MIMGU_mgv11b021346mg, partial [Erythranthe guttata]
ocean	island	13812993	13824483	Ach24g407031	hypothetical protein POPTR_0001s00830g [Populus trichocarpa]
ocean	island	9162196	9162720	Ach11g263351	hypothetical protein POPTR_0535s00220g, partial [Populus trichocarpa]
ocean	island	5119185	5122972	Ach12g063201	hypothetical protein VITISV_028177 [Vitis vinifera]
ocean	island	18859714	18861895	Ach15g142911	hypothetical protein VITISV_030147 [Vitis vinifera]
ocean	island	11946829	11947047	Ach25g445761	hypothetical protein VITISV_036853 [Vitis vinifera]
ocean	island	11950282	11950972	Ach25g228881	leucine-rich repeat receptor-like protein kinase [Arabidopsis thaliana]
ocean	island	4250832	4258324	Ach20g367881	PREDICTED: 28S ribosomal protein S29, mitochondrial isoform X2 [Nelumbo nucifera]
ocean	island	4020672	4022642	Ach01g023501	PREDICTED: 3-ketoacyl-CoA synthase 15-like [Populus euphratica]
ocean	island	12629973	12643887	Ach18g071801	PREDICTED: ATP-dependent helicase BRM [Vitis vinifera]
ocean	island	6609209	6628982	Ach25g374251	PREDICTED: cysteine--tRNA ligase, cytoplasmic-like [Erythranthe guttatus]

ocean	island	8400091	8408238	Ach03g081151	PREDICTED: flap endonuclease GEN-like 2 isoform X4 [Populus euphratica]
ocean	island	1438387	1445646	Ach23g115701	PREDICTED: MAR-binding filament-like protein 1-1 isoform X1 [Vitis vinifera]
ocean	island	9043322	9074382	Ach11g263841	PREDICTED: methionine aminopeptidase 2B-like isoform X3 [Elaeis guineensis]
ocean	island	2549164	2574696	Ach06g053631	PREDICTED: methyl-CpG-binding domain-containing protein 9 isoform X3 [Jatropha curcas]
ocean	island	5725879	5730324	Ach19g210451	PREDICTED: NAC domain-containing protein 86 isoform X2 [Vitis vinifera]
ocean	island	1238453	1250491	Ach13g458771	PREDICTED: N-alpha-acetyltransferase 20-like [Tarenaya hassleriana]
ocean	island	1203971	1205569	Ach13g308881	PREDICTED: NF-kappa-B-activating protein [Jatropha curcas]
ocean	island	4023661	4030689	Ach01g395021	PREDICTED: organic cation/carnitine transporter 7-like isoform X1 [Citrus sinensis]
ocean	island	3908851	3916626	Ach11g265751	PREDICTED: phospho-2-dehydro-3-deoxyheptonate aldolase 1, chloroplastic [Sesamum indicum]
ocean	island	18855262	18857133	Ach15g432191	PREDICTED: probable ADP-ribosylation factor GTPase-activating protein AGD13 [Vitis vinifera]
ocean	island	9094778	9112277	Ach11g263861	PREDICTED: probable methionine--tRNA ligase [Sesamum indicum]
ocean	island	11954164	11955915	Ach25g228631	PREDICTED: proline-rich protein 12-like [Prunus mume]
ocean	island	11957802	11978002	Ach25g228861	PREDICTED: protein ENHANCED DISEASE RESISTANCE 2 isoform X2 [Prunus mume]
ocean	island	6031187	6045667	Ach19g320351	PREDICTED: protein transport protein Sec24-like At4g32640 [Vitis vinifera]
ocean	island	1222327	1228708	Ach13g308761	PREDICTED: pyruvate dehydrogenase E1 component subunit alpha, mitochondrial-like isoform X1 [Gossypium raimondii]
ocean	island	1464276	1469381	Ach13g309021	PREDICTED: transcription factor bHLH69-like isoform X2 [Vitis vinifera]
ocean	island	1434729	1435907	Ach23g114711	PREDICTED: trihelix transcription factor ASIL1-like [Vitis vinifera]
ocean	island	12623254	12628351	Ach18g071911	PREDICTED: ubiquitin-conjugating enzyme E2-23 kDa [Vitis vinifera]
ocean	island	2514777	2527041	Ach06g053641	PREDICTED: ubiquitin-like-specific protease 1D isoform X3 [Jatropha curcas]
ocean	island	13803974	13804615	Ach24g319191	PREDICTED: uncharacterized protein At1g01500-like isoform X2 [Nelumbo nucifera]
ocean	island	4239267	4247140	Ach20g467071	PREDICTED: uncharacterized protein LOC100249371 isoform X1 [Vitis vinifera]

ocean	island	13792990	13801616	Ach24g319611	PREDICTED: uncharacterized protein LOC100260717 [Vitis vinifera]
ocean	island	10641878	10667998	Ach01g088281	PREDICTED: uncharacterized protein LOC103410371 [Malus domestica]
ocean	island	9027865	9034360	Ach11g263371	PREDICTED: uncharacterized protein LOC103709368 [Phoenix dactylifera]
ocean	island	8409299	8412425	Ach03g081141	PREDICTED: uncharacterized protein LOC105160492 [Sesamum indicum]
ocean	island	13805611	13805889	Ach24g319181	PREDICTED: uncharacterized protein LOC105160622 [Sesamum indicum]
ocean	island	5098185	5098718	Ach12g063211	PREDICTED: uncharacterized protein LOC105352373 [Fragaria vesca subsp. vesca]
ocean	island	1218924	1219826	Ach13g308891	PREDICTED: uncharacterized protein LOC105649981 [Jatropha curcas]
ocean	island	9022346	9025210	Ach11g263381	Protein Z, putative [Ricinus communis]
ocean	island	9195048	9206182	Ach11g263331	RNA recognition motif-containing protein [Vitis pseudoreticulata]
ocean	island	1416379	1422743	Ach12g434421	Ubiquitin-like family protein [Populus trichocarpa]
lake	JSC	15183097	15183756	Ach15g219791	hypothetical protein VITISV_013933 [Vitis vinifera]
lake	JSC	5615576	5627561	Ach13g146571	3-hydroxy-3-methylglutaryl-coenzyme A reductase, HMGR {active site} [tomatoes, cultivar VFNT cherry LA1221, Peptide Partial, 249 aa]
lake	JSC	3439628	3443973	Ach22g390981	6-phosphogluconolactonase 1 isoform 1 [Theobroma cacao]
lake	JSC	8836674	8838439	Ach03g354661	Aldo-keto reductase family 4 member C9 [Glycine soja]
lake	JSC	4260520	4261926	Ach03g154601	anthocyanin 5-aromatic acyltransferase [Vaccinium dunalianum]
lake	JSC	1570878	1573038	Ach02g250001	arginine/serine-rich splicing factor, putative [Ricinus communis]
lake	JSC	11906110	11940844	Ach05g396181	beta-galactosidase 3 [Camellia sinensis]
lake	JSC	8408075	8414503	Ach12g173651	bZIP transcription factor bZIP7 [Camellia sinensis]
lake	JSC	822915	825049	Ach26g296901	C2H2-like zinc finger protein [Theobroma cacao]
lake	JSC	5640144	5646739	Ach13g146651	Calcium-dependent lipid-binding family protein isoform 2 [Theobroma cacao]
lake	JSC	8715964	8718151	Ach23g163791	Disease resistance protein [Morus notabilis]
lake	JSC	4245044	4246748	Ach03g154591	DIV3B protein [Heptacodium miconioides]
lake	JSC	2765283	2782954	Ach14g331681	DNA2-like helicase [Aegilops tauschii]
lake	JSC	7535756	7537942	Ach26g271241	DNAJ heat shock N-terminal domain-containing protein, putative [Theobroma cacao]
lake	JSC	8843614	8857037	Ach06g354701	Endoplasmic reticulum oxidoreductins 1 isoform 1 [Theobroma cacao]
lake	JSC	12262577	12266338	Ach03g153511	Floral homeotic protein APETALA 2 [Morus notabilis]
lake	JSC	9339271	9348068	Ach29g386611	galactokinase 1 [Camellia sinensis]

lake	JSC	389379	403461	Ach26g297011	Gb:AAD20392.1 [Theobroma cacao]
lake	JSC	5779325	5781303	Ach15g001281	GTP-binding family protein [Populus trichocarpa]
lake	JSC	1919911	1920963	Ach24g117761	Heat shock protein DnaJ [Theobroma cacao]
lake	JSC	15915407	15922189	Ach26g242161	hexokinase [Actinidia chinensis]
lake	JSC	16621065	16628431	Ach23g191261	High-level expression of sugar-inducible gene 2, putative isoform 1 [Theobroma cacao]
lake	JSC	13555622	13558740	Ach25g438481	hypothetical protein CARUB_v10003614mg [Capsella rubella]
lake	JSC	8065118	8070054	Ach11g429441	hypothetical protein CICLE_v10007867mg [Citrus clementina]
lake	JSC	11689882	11707289	Ach23g258391	hypothetical protein CICLE_v10028404mg [Citrus clementina]
lake	JSC	742691	746699	Ach27g451391	hypothetical protein Csa_4G614180 [Cucumis sativus]
lake	JSC	11265531	11270582	Ach03g138941	hypothetical protein JCGZ_01259 [Jatropha curcas]
lake	JSC	7328941	7331210	Ach25g251601	hypothetical protein MIMGU_mgv1a011015mg [Erythranthe guttata]
lake	JSC	3081729	3089186	Ach26g404951	hypothetical protein OsI_31998 [Oryza sativa Indica Group]
lake	JSC	931844	939801	Ach27g448241	hypothetical protein PHAVU_007G012200g [Phaseolus vulgaris]
lake	JSC	2182347	2202329	Ach21g271631	hypothetical protein PRUPE_ppa000809mg [Prunus persica]
lake	JSC	9649220	9658530	Ach23g005111	hypothetical protein PRUPE_ppa002180mg [Prunus persica]
lake	JSC	157777	158241	Ach01g331231	hypothetical protein PRUPE_ppa012468mg [Prunus persica]
lake	JSC	6996053	6997836	Ach03g045901	hypothetical protein PRUPE_ppa019586mg [Prunus persica]
lake	JSC	16649929	16663194	Ach23g191291	hypothetical protein TRIUR3_23696 [Triticum urartu]
lake	JSC	457165	457761	Ach25g147411	hypothetical protein VITISV_005323 [Vitis vinifera]
lake	JSC	11274019	11279538	Ach03g431271	hypothetical protein VITISV_006934 [Vitis vinifera]
lake	JSC	16375158	16381529	Ach15g219101	hypothetical protein VITISV_010916 [Vitis vinifera]
lake	JSC	8057522	8057902	Ach11g124701	hypothetical protein VITISV_022341 [Vitis vinifera]
lake	JSC	4350234	4352777	Ach11g277461	hypothetical protein VITISV_029650 [Vitis vinifera]
lake	JSC	1879355	1884010	Ach10g370611	hypothetical protein VITISV_031201 [Vitis vinifera]
lake	JSC	7246174	7248933	Ach17g053021	hypothetical protein VITISV_041982 [Vitis vinifera]
lake	JSC	1968261	1970525	Ach24g427201	Multidrug resistance-associated protein 4 isoform 2 [Theobroma cacao]
lake	JSC	7776673	7789720	Ach08g300611	myosin XI, putative [Ricinus communis]
lake	JSC	4432086	4433434	Ach06g182861	NAP-like transcription factor [Vitis vinifera]
lake	JSC	6330921	6349858	Ach09g171691	phosphofructokinase [Hevea brasiliensis]
lake	JSC	5019872	5022946	Ach11g284221	pollen-specific protein [Vitis pseudoreticulata]
lake	JSC	1593187	1597434	Ach07g006701	polyol transporter [Camellia sinensis]

lake	JSC	11677900	11683535	Ach23g258411	PREDICTED: 4-coumarate--CoA ligase-like 7 [Nicotiana tomentosiformis]
lake	JSC	13461837	13466949	Ach08g320651	PREDICTED: 50S ribosomal protein L18, chloroplastic [Fragaria vesca subsp. vesca]
lake	JSC	3822230	3872228	Ach26g382131	PREDICTED: activating signal cointegrator 1 complex subunit 3 isoform X1 [Vitis vinifera]
lake	JSC	6990693	6995105	Ach03g045891	PREDICTED: acyl-CoA-binding domain-containing protein 1-like [Solanum lycopersicum]
lake	JSC	18727128	18737459	Ach23g359911	PREDICTED: adenylate kinase isoenzyme 6 homolog [Malus domestica]
lake	JSC	1869254	1886848	Ach29g368831	PREDICTED: ADP-ribosylation factor GTPase-activating protein AGD3 [Vitis vinifera]
lake	JSC	11826911	11829824	Ach11g322521	PREDICTED: ammonium transporter 3 member 1-like [Vitis vinifera]
lake	JSC	9642461	9643681	Ach23g005121	PREDICTED: arogenate dehydratase/prephenate dehydratase 6, chloroplastic-like [Nicotiana tomentosiformis]
lake	JSC	1899975	1904430	Ach29g368771	PREDICTED: aspartokinase 2, chloroplastic isoform X2 [Elaeis guineensis]
lake	JSC	1584032	1590635	Ach07g006001	PREDICTED: ATP-dependent Clp protease proteolytic subunit 6, chloroplastic isoform X1 [Jatropha curcas]
lake	JSC	15195859	15207264	Ach15g219651	PREDICTED: autophagy-related protein 18h-like isoform X1 [Nicotiana tomentosiformis]
lake	JSC	14379592	14385631	Ach07g000671	PREDICTED: BTB/POZ domain-containing protein At2g13690 [Vitis vinifera]
lake	JSC	4276474	4277883	Ach03g154401	PREDICTED: BTB/POZ domain-containing protein At3g09030 [Nicotiana tomentosiformis]
lake	JSC	1582365	1583177	Ach07g005991	PREDICTED: BURP domain-containing protein 5-like [Malus domestica]
lake	JSC	14387759	14408919	Ach07g391651	PREDICTED: callose synthase 5-like [Elaeis guineensis]
lake	JSC	5434971	5440755	Ach21g029321	PREDICTED: cyclin-A1-4 [Nelumbo nucifera]
lake	JSC	4423401	4428969	Ach06g400861	PREDICTED: cytochrome P450 704B1 [Malus domestica]
lake	JSC	3356164	3367822	Ach06g183531	PREDICTED: DEAD-box ATP-dependent RNA helicase ISE2, chloroplastic [Vitis vinifera]
lake	JSC	16950577	16961965	Ach26g241571	PREDICTED: DNA (cytosine-5)-methyltransferase CMT3-like isoform X3 [Nicotiana sylvestris]
lake	JSC	3983208	3985803	Ach23g121821	PREDICTED: DNA damage-binding protein 1a isoform X3 [Populus euphratica]
lake	JSC	3778450	3797770	Ach10g261771	PREDICTED: DNA repair protein REV1 isoform X2 [Vitis vinifera]
lake	JSC	10075794	10080827	Ach24g203971	PREDICTED: double-stranded RNA-binding protein 2-like [Solanum lycopersicum]

lake	JSC	2853669	2869279	Ach19g306231	PREDICTED: E3 ubiquitin-protein ligase UPL1 isoform X1 [Vitis vinifera]
lake	JSC	8160997	8162412	Ach19g334731	PREDICTED: elongation factor Tu, chloroplastic-like [Glycine max]
lake	JSC	10640531	10644563	Ach24g203721	PREDICTED: ethylene-responsive transcription factor RAP2-7-like isoform X2 [Nelumbo nucifera]
lake	JSC	2784138	2791583	Ach14g331821	PREDICTED: eukaryotic translation initiation factor 3 subunit B [Vitis vinifera]
lake	JSC	2797580	2804697	Ach14g331811	PREDICTED: eukaryotic translation initiation factor 3 subunit B-like [Sesamum indicum]
lake	JSC	9371719	9388498	Ach29g386711	PREDICTED: flowering time control protein FCA isoform X2 [Vitis vinifera]
lake	JSC	3456316	3457216	Ach22g470531	PREDICTED: folic acid synthesis protein fol1-like [Fragaria vesca subsp. vesca]
lake	JSC	12637302	12653330	Ach15g261211	PREDICTED: glutamine--fructose-6-phosphate aminotransferase [isomerizing] 2 [Vitis vinifera]
lake	JSC	572756	574679	Ach29g164611	PREDICTED: glutaredoxin-C4 [Solanum lycopersicum]
lake	JSC	15188587	15190583	Ach15g219661	PREDICTED: histidine-containing phosphotransfer protein 1 [Vitis vinifera]
lake	JSC	19227928	19267179	Ach03g239881	PREDICTED: histone-lysine N-methyltransferase, H3 lysine-9 specific SUVH4 [Vitis vinifera]
lake	JSC	10047557	10059400	Ach24g203981	PREDICTED: homeobox-leucine zipper protein REVOLUTA-like [Nelumbo nucifera]
lake	JSC	7326580	7328319	Ach25g251861	PREDICTED: homologous-pairing protein 2 homolog [Jatropha curcas]
lake	JSC	1894210	1896049	Ach29g368841	PREDICTED: inactive leucine-rich repeat receptor-like protein kinase CORYNE [Vitis vinifera]
lake	JSC	1033747	1055252	Ach10g370261	PREDICTED: ion channel DMI1-like isoform X1 [Populus euphratica]
lake	JSC	1928532	1942123	Ach24g117551	PREDICTED: kinesin-13A [Vitis vinifera]
lake	JSC	7273016	7273525	Ach28g457941	PREDICTED: laccase-3-like [Solanum lycopersicum]
lake	JSC	19217762	19224489	Ach03g239891	PREDICTED: light-regulated protein [Vitis vinifera]
lake	JSC	7517679	7519688	Ach26g271251	PREDICTED: magnesium protoporphyrin IX methyltransferase, chloroplastic [Vitis vinifera]
lake	JSC	1562095	1563681	Ach02g250011	PREDICTED: MATE efflux family protein LAL5-like isoform X1 [Pyrus x bretschneideri]
lake	JSC	7769190	7774606	Ach08g457241	PREDICTED: mitochondrial carrier protein MTM1-like [Brassica rapa]
lake	JSC	12544333	12554178	Ach08g096131	PREDICTED: mitochondrial import inner membrane translocase subunit tim16 [Cucumis melo]
lake	JSC	6989920	6990156	Ach03g045571	PREDICTED: mitogen-activated protein kinase kinase 5-like [Nelumbo nucifera]

lake	JSC	10064940	10074655	Ach24g202581	PREDICTED: mRNA-decapping enzyme subunit 2 [Nicotiana tomentosiformis]
lake	JSC	10224668	10261409	Ach13g295601	PREDICTED: NAD-dependent malic enzyme 59 kDa isoform, mitochondrial [Sesamum indicum]
lake	JSC	8169903	8177982	Ach19g334771	PREDICTED: pachytene checkpoint protein 2 homolog [Solanum tuberosum]
lake	JSC	3370392	3382536	Ach06g183401	PREDICTED: paired amphipathic helix protein Sin3-like 4 isoform X3 [Musa acuminata subsp. malaccensis]
lake	JSC	8167094	8168992	Ach19g334741	PREDICTED: pentatricopeptide repeat-containing protein At3g48250, chloroplastic [Vitis vinifera]
lake	JSC	11818650	11821286	Ach11g322551	PREDICTED: pentatricopeptide repeat-containing protein At3g54980, mitochondrial-like isoform X1 [Vitis vinifera]
lake	JSC	148135	150036	Ach26g296991	PREDICTED: pentatricopeptide repeat-containing protein At4g18750, chloroplastic-like [Citrus sinensis]
lake	JSC	966045	968624	Ach26g296881	PREDICTED: pentatricopeptide repeat-containing protein At5g16860 [Prunus mume]
lake	JSC	2843680	2848341	Ach19g406381	PREDICTED: pentatricopeptide repeat-containing protein At5g55840 [Vitis vinifera]
lake	JSC	11835734	11844894	Ach11g322531	PREDICTED: peptidyl-prolyl cis-trans isomerase CYP95-like isoform X1 [Cicer arietinum]
lake	JSC	3458950	3461898	Ach22g390571	PREDICTED: peptidyl-prolyl cis-trans isomerase E [Nicotiana tomentosiformis]
lake	JSC	5030209	5032250	Ach11g454991	PREDICTED: phosphoglycerate mutase [Sesamum indicum]
lake	JSC	1887216	1894559	Ach10g411921	PREDICTED: pre-rRNA-processing protein TSR2 homolog [Prunus mume]
lake	JSC	17990290	17996398	Ach06g347911	PREDICTED: probable 26S proteasome non-ATPase regulatory subunit 3 [Nelumbo nucifera]
lake	JSC	764246	764569	Ach26g406581	PREDICTED: probable ADP-ribosylation factor GTPase-activating protein AGD11 [Vitis vinifera]
lake	JSC	11892482	11904115	Ach05g047911	PREDICTED: probable glucan 1,3-alpha-glucosidase [Prunus mume]
lake	JSC	11827215	11834227	Ach20g404351	PREDICTED: probable glycosyltransferase At5g03795 [Nelumbo nucifera]
lake	JSC	11837153	11844893	Ach20g404331	PREDICTED: probable glycosyltransferase At5g03795 [Vitis vinifera]
lake	JSC	124261	126324	Ach26g296981	PREDICTED: probable leucine-rich repeat receptor-like protein kinase At1g68400 [Vitis vinifera]
lake	JSC	18748380	18750809	Ach23g359541	PREDICTED: probable WRKY transcription factor 21 [Eucalyptus grandis]
lake	JSC	9396059	9397263	Ach29g386581	PREDICTED: probable WRKY transcription factor 48 [Vitis vinifera]

lake	JSC	8362592	8367944	Ach13g307091	PREDICTED: purple acid phosphatase 15 [Vitis vinifera]
lake	JSC	5034096	5047878	Ach11g284251	PREDICTED: random slug protein 5-like [Gossypium raimondii]
lake	JSC	367810	373461	Ach18g166381	PREDICTED: ras-related protein RABF1 [Vitis vinifera]
lake	JSC	563934	566482	Ach29g164661	PREDICTED: receptor-like protein 12 [Vitis vinifera]
lake	JSC	5422131	5431108	Ach21g029201	PREDICTED: replication factor C subunit 2 [Eucalyptus grandis]
lake	JSC	16630139	16630657	Ach23g439121	PREDICTED: RING-H2 finger protein ATL72 isoform X2 [Eucalyptus grandis]
lake	JSC	1911903	1916288	Ach29g368761	PREDICTED: serine carboxypeptidase-like 45 [Vitis vinifera]
lake	JSC	1899515	1918962	Ach10g370621	PREDICTED: serine/threonine-protein kinase EDR1 [Vitis vinifera]
lake	JSC	7346841	7353536	Ach25g251591	PREDICTED: serine/threonine-protein kinase prpf4B-like [Vitis vinifera]
lake	JSC	12244745	12247939	Ach08g238131	PREDICTED: serine/threonine-protein kinase-like protein ACR4-like [Solanum tuberosum]
lake	JSC	7522141	7534012	Ach26g271081	PREDICTED: signal recognition particle subunit SRP68 [Sesamum indicum]
lake	JSC	376644	377117	Ach18g435771	PREDICTED: SKP1-like protein 11 [Nicotiana sylvestris]
lake	JSC	259895	261716	Ach01g094151	PREDICTED: staphylococcal nuclease domain-containing protein 1-like isoform X2 [Gossypium raimondii]
lake	JSC	11846651	11860054	Ach20g256251	PREDICTED: syntaxin-132 [Sesamum indicum]
lake	JSC	530336	555248	Ach29g435481	PREDICTED: TBC1 domain family member 15-like isoform X3 [Gossypium raimondii]
lake	JSC	11771020	11795548	Ach13g383891	PREDICTED: TBC1 domain family member 17-like isoform X2 [Gossypium raimondii]
lake	JSC	17997653	18000406	Ach06g347901	PREDICTED: trafficking protein particle complex subunit 2-like protein [Vitis vinifera]
lake	JSC	472857	473621	Ach27g258161	PREDICTED: transcription factor TCP7 [Vitis vinifera]
lake	JSC	403744	406383	Ach27g451371	PREDICTED: transcription initiation factor TFIID subunit 15 isoform X2 [Cicer arietinum]
lake	JSC	552610	555921	Ach27g258171	PREDICTED: transcription termination factor 2 isoform X4 [Jatropha curcas]
lake	JSC	3877022	3882460	Ach26g382141	PREDICTED: transmembrane protein adipocyte-associated 1 homolog [Gossypium raimondii]
lake	JSC	14437123	14443986	Ach17g051591	PREDICTED: ubiquitin-60S ribosomal protein L40-like [Cucumis melo]
lake	JSC	14446465	14459027	Ach17g051601	PREDICTED: ubiquitin-NEDD8-like protein RUB2 [Camelina sativa]

lake	JSC	12656612	12657205	Ach26g068271	PREDICTED: uncharacterized protein At1g08160-like [Citrus sinensis]
lake	JSC	155111	174302	Ach26g296971	PREDICTED: uncharacterized protein LOC100244234 isoform X2 [Vitis vinifera]
lake	JSC	14464657	14480291	Ach17g051611	PREDICTED: uncharacterized protein LOC100246156 isoform X3 [Vitis vinifera]
lake	JSC	13499102	13513562	Ach23g347381	PREDICTED: uncharacterized protein LOC100248282 [Vitis vinifera]
lake	JSC	2236838	2239768	Ach03g419211	PREDICTED: uncharacterized protein LOC100256408 [Vitis vinifera]
lake	JSC	15932386	15963585	Ach26g242151	PREDICTED: uncharacterized protein LOC100256902 isoform X2 [Vitis vinifera]
lake	JSC	8826190	8835691	Ach03g353891	PREDICTED: uncharacterized protein LOC100257148 isoform X2 [Vitis vinifera]
lake	JSC	10088559	10090556	Ach04g418271	PREDICTED: uncharacterized protein LOC100257501 [Vitis vinifera]
lake	JSC	16930206	16943832	Ach26g241701	PREDICTED: uncharacterized protein LOC100263956 [Vitis vinifera]
lake	JSC	13517012	13523656	Ach23g347371	PREDICTED: uncharacterized protein LOC100265339 [Vitis vinifera]
lake	JSC	8384707	8396722	Ach13g307101	PREDICTED: uncharacterized protein LOC100267035 isoform X2 [Vitis vinifera]
lake	JSC	12635947	12642459	Ach26g068281	PREDICTED: uncharacterized protein LOC102623678 isoform X1 [Citrus sinensis]
lake	JSC	8718793	8725976	Ach23g163391	PREDICTED: uncharacterized protein LOC102629203 [Citrus sinensis]
lake	JSC	8179691	8182620	Ach19g334761	PREDICTED: uncharacterized protein LOC103337936 isoform X1 [Prunus mume]
lake	JSC	13538348	13550943	Ach25g438471	PREDICTED: uncharacterized protein LOC103423381 [Malus domestica]
lake	JSC	3131670	3156522	Ach26g253591	PREDICTED: uncharacterized protein LOC103455509 [Malus domestica]
lake	JSC	12664836	12674548	Ach26g068261	PREDICTED: uncharacterized protein LOC103503043 isoform X1 [Cucumis melo]
lake	JSC	5763652	5770842	Ach15g002921	PREDICTED: uncharacterized protein LOC104103818 [Nicotiana tomentosiformis]
lake	JSC	8736108	8737770	Ach09g215851	PREDICTED: uncharacterized protein LOC104449216 isoform X3 [Eucalyptus grandis]
lake	JSC	15304756	15320289	Ach15g219611	PREDICTED: uncharacterized protein LOC104596382 isoform X2 [Nelumbo nucifera]
lake	JSC	14361325	14361825	Ach07g391641	PREDICTED: uncharacterized protein LOC104606757 [Nelumbo nucifera]
lake	JSC	7796016	7797128	Ach08g457231	PREDICTED: uncharacterized protein LOC105134795 [Populus euphratica]

lake	JSC	4263406	4269825	Ach03g154411	PREDICTED: uncharacterized protein LOC105174543 [Sesamum indicum]
lake	JSC	7322519	7326248	Ach25g251611	PREDICTED: uncharacterized protein LOC105642006 isoform X3 [Jatropha curcas]
lake	JSC	8370885	8371844	Ach13g307031	PREDICTED: uncharacterized protein LOC105955972 [Erythranthe guttatus]
lake	JSC	9358833	9359735	Ach29g386701	PREDICTED: uncharacterized protein LOC105962627 [Erythranthe guttatus]
lake	JSC	4198590	4205598	Ach11g265681	PREDICTED: uncharacterized WD repeat-containing protein C2A9.03 [Vitis vinifera]
lake	JSC	341078	348506	Ach18g166121	PREDICTED: vacuolar amino acid transporter 1 isoform X2 [Sesamum indicum]
lake	JSC	12597635	12606467	Ach08g424661	PREDICTED: vesicle transport protein GOT1B isoform X2 [Eucalyptus grandis]
lake	JSC	9349289	9352030	Ach29g386681	PREDICTED: zinc finger MYM-type protein 1-like [Citrus sinensis]
lake	JSC	2421531	2422343	Ach22g286491	PREDICTED: zinc-finger homeodomain protein 9-like [Nicotiana sylvestris]
lake	JSC	8723889	8734364	Ach09g215841	Protein FAM135A-like protein [Glycine soja]
lake	JSC	12619498	12628620	Ach15g261201	Serine carboxypeptidase-like 40 [Theobroma cacao]
lake	JSC	2928644	2930633	Ach26g253521	Transcription factor BEE 3 [Glycine soja]
lake	JSC	223428	226105	Ach20g013921	WRKY transcription factor 22 -like protein [Gossypium arboreum]
lake	JSC	2751513	2763838	Ach14g331671	Zinc ion binding isoform 1 [Theobroma cacao]
lake	YMC	1972430	1981503	Ach10g083561	PREDICTED: protein BONZAI 3-like isoform X2 [Vitis vinifera]
lake	YMC	7906809	7920770	Ach27g338651	PREDICTED: HEAT repeat-containing protein 5B [Vitis vinifera]
lake	YMC	5425198	5447565	Ach15g392661	PREDICTED: regulator of nonsense transcripts 1 homolog isoform X2 [Vitis vinifera]
lake	YMC	13175677	13176042	Ach07g000191	hypothetical protein VITISV_020733 [Vitis vinifera]
lake	YMC	5352252	5364311	Ach01g185131	1-deoxy-D-xylulose-5-phosphate synthase [Actinidia chinensis]
lake	YMC	16897425	16905251	Ach06g232491	1-hydroxy-2-methyl-butenyl 4-diphosphate reductase [Actinidia deliciosa]
lake	YMC	6517865	6519658	Ach06g232061	AWPM-19-like family protein [Theobroma cacao]
lake	YMC	3691481	3702044	Ach04g329231	basic blue copper family protein [Populus trichocarpa]
lake	YMC	15683630	15686125	Ach05g145261	class I chitinase [Dimocarpus longan]
lake	YMC	13616841	13618703	Ach23g346881	EIN3-like protein EIL1 [Actinidia chinensis]
lake	YMC	13607365	13609233	Ach23g347321	EIN3-like protein EIL2 [Actinidia chinensis]
lake	YMC	7233262	7234707	Ach25g251811	Eukaryotic aspartyl protease family protein [Theobroma cacao]
lake	YMC	1989906	1996631	Ach10g083521	Eukaryotic translation initiation factor 3 subunit I [Gossypium arboreum]

lake	YMC	8432804	8434084	Ach09g401811	fasciclin-like arabinogalactan protein [Vitis hybrid cultivar]
lake	YMC	7298789	7311335	Ach03g045991	Glutamate synthase 1 [NADH], chloroplastic -like protein [Gossypium arboreum]
lake	YMC	6417001	6420504	Ach07g175371	GTP-binding protein SAR1A-like [Solanum tuberosum]
lake	YMC	8971239	8974171	Ach03g353951	Haloacid dehalogenase-like hydrolase superfamily protein isoform 2 [Theobroma cacao]
lake	YMC	7692098	7696449	Ach19g138671	hypothetical protein B456_005G136200 [Gossypium raimondii]
lake	YMC	9248413	9252737	Ach11g404141	hypothetical protein B456_005G140200 [Gossypium raimondii]
lake	YMC	2787705	2797170	Ach29g196971	hypothetical protein B456_005G195600 [Gossypium raimondii]
lake	YMC	15484537	15497285	Ach06g146961	hypothetical protein B456_008G043200 [Gossypium raimondii]
lake	YMC	9256432	9262448	Ach24g442501	hypothetical protein B456_009G310800 [Gossypium raimondii]
lake	YMC	12825877	12842065	Ach03g408121	hypothetical protein CICLE_v10011846mg [Citrus clementina]
lake	YMC	3718515	3721157	Ach01g022941	hypothetical protein CICLE_v10012648mg [Citrus clementina]
lake	YMC	2282359	2305684	Ach18g004451	hypothetical protein CICLE_v10014454mg [Citrus clementina]
lake	YMC	709545	710903	Ach05g205151	hypothetical protein CISIN_1g008539mg [Citrus sinensis]
lake	YMC	15664028	15668929	Ach05g145291	hypothetical protein CISIN_1g020187mg [Citrus sinensis]
lake	YMC	9262996	9264400	Ach24g202411	hypothetical protein CISIN_1g031655mg [Citrus sinensis]
lake	YMC	11301218	11301562	Ach25g432051	hypothetical protein CISIN_1g040576mg [Citrus sinensis]
lake	YMC	13147205	13149962	Ach07g000411	hypothetical protein EUTSA_v100035741mg, partial [Eutrema salsugineum]
lake	YMC	1174272	1175009	Ach06g425071	hypothetical protein JCGZ_17923 [Jatropha curcas]
lake	YMC	397968	398372	Ach02g047131	hypothetical protein L484_001582 [Morus notabilis]
lake	YMC	14825145	14825531	Ach01g288951	hypothetical protein L484_008283 [Morus notabilis]
lake	YMC	6446823	6447332	Ach01g406771	hypothetical protein L484_011949 [Morus notabilis]
lake	YMC	25698	27146	Ach11g389251	hypothetical protein L484_016045 [Morus notabilis]
lake	YMC	9265216	9265869	Ach24g202421	hypothetical protein L484_017051 [Morus notabilis]
lake	YMC	16363316	16363558	Ach13g398181	hypothetical protein MTR_1g016950 [Medicago truncatula]
lake	YMC	19130	23803	Ach11g389241	hypothetical protein OsI_37796 [Oryza sativa Indica Group]

lake	YMC	9945434	9949975	Ach11g134381	hypothetical protein POPTR_0001s14680g [Populus trichocarpa]
lake	YMC	14827655	14828041	Ach01g288961	hypothetical protein POPTR_0003s17360g [Populus trichocarpa]
lake	YMC	6766323	6766676	Ach10g086051	hypothetical protein POPTR_0005s20850g [Populus trichocarpa]
lake	YMC	16377854	16381172	Ach13g145041	hypothetical protein POPTR_0008s12630g [Populus trichocarpa]
lake	YMC	10294886	10300179	Ach07g110491	hypothetical protein POPTR_0010s10990g [Populus trichocarpa]
lake	YMC	1855806	1856030	Ach18g003361	hypothetical protein POPTR_0010s24510g [Populus trichocarpa]
lake	YMC	16567457	16567705	Ach11g275441	hypothetical protein POPTR_0014s09080g [Populus trichocarpa]
lake	YMC	379517	387049	Ach02g047111	hypothetical protein POPTR_0015s00350g [Populus trichocarpa]
lake	YMC	5006613	5012125	Ach16g411361	hypothetical protein POPTR_0016s06240g [Populus trichocarpa]
lake	YMC	10199741	10206270	Ach11g441351	hypothetical protein POPTR_0019s11180g [Populus trichocarpa]
lake	YMC	6477214	6491734	Ach01g307961	hypothetical protein POPTR_0025s00390g [Populus trichocarpa]
lake	YMC	7923262	7923858	Ach27g339641	hypothetical protein PRUPE_ppa001251mg [Prunus persica]
lake	YMC	10162235	10167921	Ach11g441341	hypothetical protein PRUPE_ppa012354mg [Prunus persica]
lake	YMC	9137218	9150630	Ach19g097871	hypothetical protein SCLCIDRAFT_30966 [Scleroderma citrinum Foug A]
lake	YMC	2309450	2329582	Ach18g004461	hypothetical protein SORBIDRAFT_09g030580 [Sorghum bicolor]
lake	YMC	10301208	10304997	Ach11g441371	hypothetical protein VITISV_010464 [Vitis vinifera]
lake	YMC	9278919	9281015	Ach11g263911	hypothetical protein VITISV_023482 [Vitis vinifera]
lake	YMC	4118371	4118775	Ach21g372111	hypothetical protein VITISV_035517 [Vitis vinifera]
lake	YMC	715752	733598	Ach05g205141	hypothetical protein VITISV_036910 [Vitis vinifera]
lake	YMC	16221201	16221836	Ach11g275721	Late embryogenesis abundant hydroxyproline-rich glycofamily protein, putative [Theobroma cacao]
lake	YMC	15477825	15481014	Ach06g147281	malate dehydrogenase, putative [Ricinus communis]
lake	YMC	6685482	6687440	Ach28g238771	Maternal effect embryo arrest 59 [Theobroma cacao]
lake	YMC	3297192	3300938	Ach14g027041	Mitochondrial transcription termination factor family protein, putative [Theobroma cacao]
lake	YMC	15896293	15896820	Ach01g464831	neurofilament heavy protein, putative [Medicago truncatula]
lake	YMC	2336131	2344729	Ach18g003171	phabulosa [Sarracenia purpurea]
lake	YMC	9831793	9845454	Ach13g352941	Phospholipase D beta 1 isoform 1 [Theobroma cacao]

lake	YMC	5119660	5121142	Ach25g080501	polygalacturonase A [Actinidia chinensis]
lake	YMC	7901005	7901262	Ach27g338661	PREDICTED: 30S ribosomal protein 3, chloroplastic-like [Vitis vinifera]
lake	YMC	9697471	9710694	Ach12g147501	PREDICTED: 3beta-hydroxysteroid-dehydrogenase/decarboxylase isoform 2 [Vitis vinifera]
lake	YMC	5125797	5128424	Ach25g422541	PREDICTED: 40S ribosomal protein S2-3-like [Cucumis melo]
lake	YMC	7834731	7840808	Ach13g034621	PREDICTED: acyl-coenzyme A thioesterase 9, mitochondrial-like [Solanum tuberosum]
lake	YMC	6596163	6599403	Ach03g274741	PREDICTED: ADP-ribosylation factor 1-like 2 isoform X1 [Phoenix dactylifera]
lake	YMC	19469089	19470801	Ach08g149451	PREDICTED: alpha carbonic anhydrase 1, chloroplastic isoform X1 [Cucumis sativus]
lake	YMC	1087213	1105365	Ach19g182041	PREDICTED: alpha,alpha-trehalose-phosphate synthase [UDP-forming] 1-like [Vitis vinifera]
lake	YMC	14641934	14644864	Ach24g446281	PREDICTED: arginine/serine-rich-splicing factor RSP40-like isoform X1 [Citrus sinensis]
lake	YMC	9308171	9335560	Ach24g204121	PREDICTED: armadillo repeat-containing kinesin-like protein 1 isoform X2 [Vitis vinifera]
lake	YMC	360465	364068	Ach02g418561	PREDICTED: auxin-induced protein X15-like [Nelumbo nucifera]
lake	YMC	9057761	9064846	Ach13g352541	PREDICTED: auxin-responsive protein IAA11 isoform X2 [Jatropha curcas]
lake	YMC	17110236	17114920	Ach15g111161	PREDICTED: bifunctional riboflavin kinase/FMN phosphatase [Nicotiana sylvestris]
lake	YMC	16182715	16190186	Ach26g242061	PREDICTED: calcium-dependent protein kinase 8 [Cucumis sativus]
lake	YMC	13252964	13274549	Ach13g349921	PREDICTED: CCR4-NOT transcription complex subunit 3 isoform X1 [Vitis vinifera]
lake	YMC	13583593	13586600	Ach03g198291	PREDICTED: charged multivesicular body protein 5-like [Tarenaya hassleriana]
lake	YMC	7031397	7048593	Ach20g119331	PREDICTED: chloride channel protein CLC-d isoform X1 [Prunus mume]
lake	YMC	16571855	16578290	Ach11g404791	PREDICTED: CMP-sialic acid transporter 1-like isoform X1 [Nelumbo nucifera]
lake	YMC	12899746	12915885	Ach11g283701	PREDICTED: coatomer subunit beta'-2-like [Glycine max]
lake	YMC	17116040	17138485	Ach15g112001	PREDICTED: conserved oligomeric Golgi complex subunit 3 [Vitis vinifera]
lake	YMC	9904332	9917433	Ach13g352081	PREDICTED: cytochrome b561 and DOMON domain-containing protein At5g47530-like [Gossypium raimondii]
lake	YMC	7882127	7885981	Ach27g338671	PREDICTED: cytokinin hydroxylase [Sesamum indicum]

lake	YMC	2690797	2692407	Ach19g306271	PREDICTED: DELLA protein RGL1-like [Fragaria vesca subsp. vesca]
lake	YMC	4363982	4372589	Ach29g122811	PREDICTED: deoxynucleoside triphosphate triphosphohydrolase SAMHD1 homolog [Solanum tuberosum]
lake	YMC	1176189	1182192	Ach06g099571	PREDICTED: DNA polymerase alpha subunit B [Nicotiana sylvestris]
lake	YMC	8291385	8292470	Ach18g072781	PREDICTED: early nodulin-like protein 1 [Nicotiana tomentosiformis]
lake	YMC	3709143	3715356	Ach01g023391	PREDICTED: ethanolamine-phosphate cytidylyltransferase [Vitis vinifera]
lake	YMC	10186610	10187155	Ach11g205451	PREDICTED: ethylene-responsive transcription factor ERF021-like [Populus euphratica]
lake	YMC	9255113	9264669	Ach11g263321	PREDICTED: eukaryotic translation initiation factor 3 subunit I [Cucumis sativus]
lake	YMC	9277167	9306575	Ach24g202431	PREDICTED: exocyst complex component SEC8 [Fragaria vesca subsp. vesca]
lake	YMC	11302784	11303974	Ach25g141651	PREDICTED: expansin-like B1 [Vitis vinifera]
lake	YMC	10311553	10339229	Ach11g205411	PREDICTED: Fanconi anemia group M protein isoform X2 [Vitis vinifera]
lake	YMC	10150165	10157184	Ach11g205461	PREDICTED: far upstream element-binding protein 1 isoform X1 [Vitis vinifera]
lake	YMC	3705712	3706563	Ach01g022961	PREDICTED: F-box protein At4g19940-like [Sesamum indicum]
lake	YMC	7841931	7847389	Ach13g034901	PREDICTED: formin-like protein 1-like [Citrus sinensis]
lake	YMC	12699507	12705391	Ach01g209171	PREDICTED: formin-like protein 2 [Jatropha curcas]
lake	YMC	5383657	5386875	Ach17g399451	PREDICTED: GDSL esterase/lipase At4g26790 [Vitis vinifera]
lake	YMC	1875003	1875386	Ach18g003341	PREDICTED: germin-like protein subfamily 3 member 2 [Fragaria vesca subsp. vesca]
lake	YMC	13244238	13247284	Ach13g349931	PREDICTED: glucan endo-1,3-beta-glucosidase 8 [Sesamum indicum]
lake	YMC	2680361	2686347	Ach19g406421	PREDICTED: glyceraldehyde-3-phosphate dehydrogenase GAPCP1, chloroplastic-like [Pyrus x bretschneideri]
lake	YMC	8521189	8522095	Ach18g072301	PREDICTED: G-type lectin S-receptor-like serine/threonine-protein kinase At4g27290 [Vitis vinifera]
lake	YMC	17802724	17811370	Ach15g111571	PREDICTED: heat shock 70 kDa protein 16 [Nicotiana tomentosiformis]
lake	YMC	19477024	19481870	Ach08g398751	PREDICTED: heterogeneous nuclear ribonucleoprotein R-like [Sesamum indicum]

lake	YMC	4993349	5004348	Ach16g365651	PREDICTED: histone-lysine N-methyltransferase ATXR4-like [Malus domestica]
lake	YMC	1839780	1849365	Ach18g003371	PREDICTED: iron-sulfur assembly protein IscA-like 1, mitochondrial [Populus euphratica]
lake	YMC	11484671	11495555	Ach26g078181	PREDICTED: J domain-containing protein spf31 [Eucalyptus grandis]
lake	YMC	1930450	1939428	Ach13g017811	PREDICTED: KH domain-containing protein At4g18375-like [Sesamum indicum]
lake	YMC	8133367	8139925	Ach03g081261	PREDICTED: kinesin-like protein KIF22 isoform X2 [Nicotiana sylvestris]
lake	YMC	3244086	3251103	Ach07g262081	PREDICTED: KRR1 small subunit processome component homolog [Sesamum indicum]
lake	YMC	11336393	11354664	Ach25g141641	PREDICTED: lon protease homolog 2, peroxisomal-like isoform X2 [Pyrus x bretschneideri]
lake	YMC	1982509	1987139	Ach10g083531	PREDICTED: LOW QUALITY PROTEIN: cyclin-L1-1 [Sesamum indicum]
lake	YMC	2117424	2129855	Ach17g259531	PREDICTED: LOW QUALITY PROTEIN: sister-chromatid cohesion protein 3-like [Populus euphratica]
lake	YMC	15870161	15899009	Ach15g219381	PREDICTED: luminal-binding protein 4-like [Nelumbo nucifera]
lake	YMC	7855286	7861437	Ach13g417201	PREDICTED: mediator of RNA polymerase II transcription subunit 19a-like isoform X2 [Eucalyptus grandis]
lake	YMC	16192674	16219137	Ach01g190901	PREDICTED: methyltransferase-like protein 13 [Vitis vinifera]
lake	YMC	1149795	1166166	Ach06g099581	PREDICTED: microtubule-associated protein futsch [Vitis vinifera]
lake	YMC	6801161	6825129	Ach11g262751	PREDICTED: mitogen-activated protein kinase kinase kinase 1-like [Gossypium raimondii]
lake	YMC	2104644	2115181	Ach17g259521	PREDICTED: NPL4-like protein 2 [Sesamum indicum]
lake	YMC	2694918	2728978	Ach19g306261	PREDICTED: nuclear cap-binding protein subunit 1 [Vitis vinifera]
lake	YMC	9676287	9682747	Ach12g123991	PREDICTED: nuclear transcription factor Y subunit A-1-like isoform X2 [Nelumbo nucifera]
lake	YMC	1997054	1999974	Ach10g083571	PREDICTED: pentatricopeptide repeat-containing protein At1g03560, mitochondrial-like [Nelumbo nucifera]
lake	YMC	9219123	9221390	Ach11g404131	PREDICTED: pentatricopeptide repeat-containing protein At1g71420 [Prunus mume]
lake	YMC	10192722	10194785	Ach11g205441	PREDICTED: pentatricopeptide repeat-containing protein At1g71460, chloroplastic [Vitis vinifera]
lake	YMC	15886813	15887802	Ach01g431011	PREDICTED: pentatricopeptide repeat-containing protein At3g46790, chloroplastic-like [Cucumis sativus]

lake	YMC	9858560	9860694	Ach13g352111	PREDICTED: peroxisome biogenesis protein 16-like isoform X2 [Pyrus x bretschneideri]
lake	YMC	9376888	9385652	Ach22g015871	PREDICTED: phospholipase A(1) LCAT3 isoform X2 [Prunus mume]
lake	YMC	16912812	16939246	Ach06g233271	PREDICTED: phospholipase A-2-activating protein-like [Malus domestica]
lake	YMC	51545	55621	Ach06g169781	PREDICTED: potassium transporter 5 [Vitis vinifera]
lake	YMC	9075539	9090549	Ach13g464621	PREDICTED: pre-mRNA-processing factor 39-like isoform X1 [Populus euphratica]
lake	YMC	35828	41228	Ach06g169791	PREDICTED: probable 3-beta-hydroxysteroid-Delta(8),Delta(7)-isomerase [Erythranthe guttatus]
lake	YMC	15691148	15695310	Ach05g145421	PREDICTED: probable acyl-activating enzyme 2 isoform X1 [Jatropha curcas]
lake	YMC	49361	53959	Ach11g389221	PREDICTED: probable cytokinin riboside 5'-monophosphate phosphoribohydrolase LOGL2-like [Citrus sinensis]
lake	YMC	3408828	3431390	Ach05g147711	PREDICTED: probable cytosolic oligopeptidase A [Sesamum indicum]
lake	YMC	2318980	2321424	Ach07g240911	PREDICTED: probable indole-3-acetic acid-amido synthetase GH3.1 [Sesamum indicum]
lake	YMC	14852749	14855127	Ach01g455811	PREDICTED: probable leucine-rich repeat receptor-like protein kinase At2g33170 [Vitis vinifera]
lake	YMC	4532826	4547169	Ach07g459551	PREDICTED: probable protein S-acyltransferase 14 [Nelumbo nucifera]
lake	YMC	10666849	10673745	Ach27g434621	PREDICTED: probable RNA-binding protein 46 isoform X3 [Vitis vinifera]
lake	YMC	7048035	7052688	Ach03g045531	PREDICTED: protein ABIL2-like [Sesamum indicum]
lake	YMC	4104840	4116190	Ach21g371941	PREDICTED: protein disulfide isomerase-like 5-2 [Sesamum indicum]
lake	YMC	8292976	8310992	Ach18g072401	PREDICTED: protein disulfide isomerase-like 5-4 [Vitis vinifera]
lake	YMC	6395057	6398433	Ach07g175361	PREDICTED: protein ROOT PRIMORDIUM DEFECTIVE 1 [Prunus mume]
lake	YMC	15678069	15682641	Ach05g145271	PREDICTED: protein trichome birefringence-like 34 [Vitis vinifera]
lake	YMC	9151667	9156932	Ach19g097511	PREDICTED: PTI1-like tyrosine-protein kinase At3g15890 [Jatropha curcas]
lake	YMC	17101790	17104932	Ach15g111151	PREDICTED: putative F-box protein At1g49610 [Nelumbo nucifera]
lake	YMC	8532623	8532952	Ach18g421271	PREDICTED: putative hydrolase C777.06c [Nicotiana tomentosiformis]
lake	YMC	889765	890360	Ach09g063501	PREDICTED: ras-related protein RABA4d-like [Glycine max]

lake	YMC	16225720	16228286	Ach11g275731	PREDICTED: reticulon-like protein B5 [Nicotiana sylvestris]
lake	YMC	9178856	9181430	Ach13g352481	PREDICTED: rho GDP-dissociation inhibitor 1-like [Jatropha curcas]
lake	YMC	3717027	3722661	Ach04g329261	PREDICTED: ribosome maturation protein SBDS [Jatropha curcas]
lake	YMC	4374865	4381567	Ach29g122831	PREDICTED: RNA-binding protein 25 isoform X2 [Vitis vinifera]
lake	YMC	9972673	9998647	Ach11g205501	PREDICTED: SCY1-like protein 2 [Vitis vinifera]
lake	YMC	15715442	15730873	Ach05g145431	PREDICTED: serine acetyltransferase 2-like [Nicotiana sylvestris]
lake	YMC	4417124	4423655	Ach29g122451	PREDICTED: serine/arginine-rich splicing factor RSZ21A isoform X2 [Phoenix dactylifera]
lake	YMC	8442078	8444654	Ach09g215471	PREDICTED: serine/threonine-protein kinase Aurora-3 isoform X1 [Sesamum indicum]
lake	YMC	10758687	10764819	Ach24g202871	PREDICTED: serine/threonine-protein kinase CDL1-like [Sesamum indicum]
lake	YMC	19998601	20013480	Ach08g398971	PREDICTED: serine/threonine-protein kinase CTR1 [Vitis vinifera]
lake	YMC	11257178	11269666	Ach09g020211	PREDICTED: serine/threonine-protein kinase CTR1 isoform X1 [Jatropha curcas]
lake	YMC	8449074	8467714	Ach09g215461	PREDICTED: spastin-like isoform X1 [Citrus sinensis]
lake	YMC	1117015	1125183	Ach19g182661	PREDICTED: subtilisin-like protease SBT1.7 [Erythranthe guttatus]
lake	YMC	9184947	9197900	Ach13g352691	PREDICTED: succinyl-CoA ligase [ADP-forming] subunit beta, mitochondrial [Elaeis guineensis]
lake	YMC	8277734	8284884	Ach18g421241	PREDICTED: TBC1 domain family member 15-like isoform X2 [Nicotiana sylvestris]
lake	YMC	10766833	10781131	Ach24g203681	PREDICTED: TBC1 domain family member 8B [Prunus mume]
lake	YMC	7681099	7682548	Ach19g137541	PREDICTED: T-complex protein 1 subunit gamma-like [Phoenix dactylifera]
lake	YMC	3723504	3724473	Ach04g329271	PREDICTED: thioredoxin [Vitis vinifera]
lake	YMC	9851500	9852474	Ach13g352121	PREDICTED: transcription factor TCP9 [Vitis vinifera]
lake	YMC	8115710	8122015	Ach03g081271	PREDICTED: U-box domain-containing protein 32 isoform X1 [Vitis vinifera]
lake	YMC	392552	395355	Ach02g047121	PREDICTED: UNC93-like protein 1 [Malus domestica]
lake	YMC	17814281	17821985	Ach15g111711	PREDICTED: uncharacterized GPI-anchored protein At1g61900 [Vitis vinifera]
lake	YMC	8128299	8132365	Ach03g081411	PREDICTED: uncharacterized protein At3g49055 [Vitis vinifera]
lake	YMC	7365385	7370960	Ach25g251571	PREDICTED: uncharacterized protein LOC100242253 isoform X2 [Vitis vinifera]

lake	YMC	6582286	6594875	Ach03g274751	PREDICTED: uncharacterized protein LOC100247726 isoform X1 [Vitis vinifera]
lake	YMC	7896912	7900036	Ach27g339631	PREDICTED: uncharacterized protein LOC100249716 [Vitis vinifera]
lake	YMC	6501544	6511599	Ach06g232051	PREDICTED: uncharacterized protein LOC100251997 [Vitis vinifera]
lake	YMC	1857842	1867327	Ach18g003351	PREDICTED: uncharacterized protein LOC100252823 isoform X1 [Vitis vinifera]
lake	YMC	8667702	8688790	Ach07g224231	PREDICTED: uncharacterized protein LOC100254417 isoform X1 [Vitis vinifera]
lake	YMC	5134039	5158908	Ach25g080511	PREDICTED: uncharacterized protein LOC100255472 isoform X1 [Vitis vinifera]
lake	YMC	16156359	16184475	Ach01g190881	PREDICTED: uncharacterized protein LOC100258878 [Vitis vinifera]
lake	YMC	12977318	12994816	Ach21g396671	PREDICTED: uncharacterized protein LOC100259195 [Vitis vinifera]
lake	YMC	15680594	15693903	Ach01g135951	PREDICTED: uncharacterized protein LOC100260062 isoform X2 [Vitis vinifera]
lake	YMC	7361651	7363772	Ach25g251581	PREDICTED: uncharacterized protein LOC100266408 isoform X1 [Vitis vinifera]
lake	YMC	16050568	16059242	Ach28g148791	PREDICTED: uncharacterized protein LOC100267227 [Vitis vinifera]
lake	YMC	2337455	2357746	Ach07g240871	PREDICTED: uncharacterized protein LOC100267918 [Vitis vinifera]
lake	YMC	1416797	1429128	Ach05g423591	PREDICTED: uncharacterized protein LOC100853492 [Vitis vinifera]
lake	YMC	9069273	9072347	Ach13g352531	PREDICTED: uncharacterized protein LOC102586584 [Solanum tuberosum]
lake	YMC	16044969	16045250	Ach28g148801	PREDICTED: uncharacterized protein LOC102593296 [Solanum tuberosum]
lake	YMC	2758326	2760565	Ach29g196131	PREDICTED: uncharacterized protein LOC102606262 [Solanum tuberosum]
lake	YMC	6465005	6475363	Ach01g307971	PREDICTED: uncharacterized protein LOC102622022 [Citrus sinensis]
lake	YMC	5013597	5043664	Ach16g365641	PREDICTED: uncharacterized protein LOC102624787 isoform X1 [Citrus sinensis]
lake	YMC	9130501	9134082	Ach19g424781	PREDICTED: uncharacterized protein LOC103328095 isoform X2 [Prunus mume]
lake	YMC	10159915	10161339	Ach11g441331	PREDICTED: uncharacterized protein LOC103411943 [Malus domestica]
lake	YMC	18677782	18681187	Ach08g066801	PREDICTED: uncharacterized protein LOC103490615 [Cucumis melo]
lake	YMC	1852357	1853001	Ach18g394191	PREDICTED: uncharacterized protein LOC104105517 [Nicotiana tomentosiformis]

lake	YMC	16178479	16180468	Ach26g241201	PREDICTED: uncharacterized protein LOC104112615 [Nicotiana tomentosiformis]
lake	YMC	6767347	6789811	Ach10g423531	PREDICTED: uncharacterized protein LOC104237992 isoform X1 [Nicotiana sylvestris]
lake	YMC	503705	524966	Ach05g449641	PREDICTED: uncharacterized protein LOC104432514 isoform X1 [Eucalyptus grandis]
lake	YMC	32590	35058	Ach11g389231	PREDICTED: uncharacterized protein LOC104605392 [Nelumbo nucifera]
lake	YMC	16212350	16219971	Ach26g241211	PREDICTED: uncharacterized protein LOC104612993 [Nelumbo nucifera]
lake	YMC	12737	13863	Ach06g169801	PREDICTED: uncharacterized protein LOC104722491 isoform X2 [Camelina sativa]
lake	YMC	909633	921873	Ach09g063481	PREDICTED: uncharacterized protein LOC104880112 isoform X1 [Vitis vinifera]
lake	YMC	4970	10905	Ach06g436341	PREDICTED: uncharacterized protein LOC105110836 [Populus euphratica]
lake	YMC	14828908	14829303	Ach01g288971	PREDICTED: uncharacterized protein LOC105123170 [Populus euphratica]
lake	YMC	19472469	19473278	Ach08g149101	PREDICTED: uncharacterized protein LOC105174057 [Sesamum indicum]
lake	YMC	5397602	5399656	Ach17g399441	PREDICTED: uncharacterized protein LOC105178634 [Sesamum indicum]
lake	YMC	13623786	13637058	Ach23g347311	PREDICTED: uncharacterized protein LOC105629320 [Jatropha curcas]
lake	YMC	1170807	1171571	Ach06g099011	PREDICTED: uncharacterized protein LOC105644999 [Jatropha curcas]
lake	YMC	5335882	5338934	Ach11g021121	PREDICTED: uncharacterized protein LOC105974441 [Erythranthe guttatus]
lake	YMC	7216228	7222410	Ach08g292071	PREDICTED: uridine 5'-monophosphate synthase [Erythranthe guttatus]
lake	YMC	9105262	9129179	Ach19g097521	PREDICTED: zinc finger BED domain-containing protein DAYSLEEPER-like [Vitis vinifera]
lake	YMC	1432447	1443880	Ach05g091151	PREDICTED: zinc finger CCCH domain-containing protein 62-like [Vitis vinifera]
lake	YMC	11420114	11424216	Ach09g019451	protein phosphatase 2c, putative [Ricinus communis]
lake	YMC	15696317	15697685	Ach01g135931	Pti1-like kinase [Medicago truncatula]
lake	YMC	6399362	6411535	Ach07g436991	putative pyruvate dehydrogenase E3 subunit [Capsicum annuum]
lake	YMC	7684185	7685351	Ach19g137531	ribulose-1,5-bisphosphate carboxylase/oxygenase large subunit, partial (chloroplast) [Hamamelis x intermedia]
lake	YMC	7317425	7320912	Ach03g046001	RING/U-box superfamily protein, putative [Theobroma cacao]
lake	YMC	7059477	7061205	Ach03g045931	RWP-RK domain-containing protein, putative [Theobroma cacao]

lake	YMC	11464871	11483621	Ach26g078081	Serine/threonine-protein phosphatase BSL3 -like protein [Gossypium arboreum]
lake	YMC	12842329	12859700	Ach03g408111	SnRK1.3 [Camellia sinensis]
lake	YMC	16229744	16243030	Ach11g404671	Structural maintenance of chromosome 1 protein, putative [Ricinus communis]
lake	YMC	2374778	2375101	Ach13g472501	Thylakoid lumenal P17.1 protein [Theobroma cacao]
lake	YMC	3728691	3741537	Ach04g329221	Transcriptional corepressor SEUSS, putative [Ricinus communis]
lake	YMC	9892666	9896086	Ach13g352091	UDP-galactose/UDP-glucose transporter 4 [Morus notabilis]
lake	YMC	7105661	7106533	Ach04g396371	VQ motif-containing protein, putative [Theobroma cacao]

Table S9. Genes in the GWAS analysis for the trichome trait.

Gegne__ID	Annotation
Ach29g356271	14-3-3-like protein GF14 iota [Morus notabilis]
Ach00g311611	1-deoxy-D-xylulose-5-phosphate synthase [Actinidia chinensis]
Ach00g222771	20S proteasome alpha subunit C1 isoform 1 [Theobroma cacao]
Ach10g189751	20S proteasome alpha subunit C1 isoform 1 [Theobroma cacao]
Ach00g264361	20S proteasome alpha subunit G1 [Theobroma cacao]
Ach15g184361	26S proteasome non-ATPase regulatory subunit RPN12A [Morus notabilis]
Ach29g364301	30S ribosomal protein S10 [Populus trichocarpa]
Ach05g264541	3-hydroxy-3-methylglutaryl coenzyme A reductase [Camellia sinensis]
Ach03g045511	3-ketoacyl-acyl carrier protein synthase I [Theobroma cacao]
Ach22g272681	3-phosphoinositide-dependent protein kinase-1 [Solanum lycopersicum]
Ach24g041091	40S ribosomal protein S15D [Hevea brasiliensis]
Ach00g382041	6-phosphofructokinase 5 [Morus notabilis]
Ach05g389821	ABC transporter B family member 26, chloroplastic [Aegilops tauschii]
Ach06g232011	ABC transporter family protein [Populus trichocarpa]
Ach00g240381	Actin-11 [Theobroma cacao]
Ach15g109181	actin-depolymerizing factor family protein [Populus trichocarpa]
Ach00g461241	actinidin Act3a [Actinidia eriantha]
Ach22g415581	acyl carrier protein, putative [Ricinus communis]
Ach00g324381	ADP-ribosylation factor [Gossypium arboreum]
Ach29g122721	ADP-ribosylation factor [Gossypium arboreum]
Ach14g022221	Amidase family protein isoform 1 [Theobroma cacao]
Ach19g034331	amsh, putative [Ricinus communis]
Ach00g009601	anthranilate synthase alpha 1 [Camptotheca acuminata]
Ach00g033271	AP2/ERF domain-containing transcription factor, putative [Theobroma cacao]
Ach29g414041	arginine N-methyltransferase protein [Platanus x acerifolia]
Ach13g394481	ASYNAPTIC 1 family protein, partial [Populus trichocarpa]
Ach24g082471	Ataxin-7-like protein 1 [Gossypium arboreum]
Ach16g212461	ATP binding protein, putative [Ricinus communis]
Ach00g130951	Auxin-induced protein 6B, putative [Ricinus communis]
Ach00g260251	Auxin-induced protein 6B, putative [Ricinus communis]
Ach06g232061	AWPM-19-like family protein [Theobroma cacao]
Ach00g310341	Basic helix-loop-helix DNA-binding family protein [Theobroma cacao]
Ach14g027171	Basic helix-loop-helix DNA-binding superfamily protein isoform 2 [Theobroma cacao]
Ach00g361761	Basic helix-loop-helix DNA-binding superfamily protein, putative [Theobroma cacao]
Ach09g171161	beta-1,3-glucanase [Camellia sinensis]

Ach00g289721	beta-galactosidase 2 precursor [Petunia x hybrida]
Ach00g311571	Bifunctional aspartokinase/homoserine dehydrogenase [Morus notabilis]
Ach18g156681	BnaA03g58360D, partial [Brassica napus]
Ach00g124721	BnaA08g12440D [Brassica napus]
Ach24g082551	BnaA10g16340D [Brassica napus]
Ach18g394041	BnaAnng11880D [Brassica napus]
Ach03g185641	BnaC02g08260D [Brassica napus]
Ach13g469781	BnaC07g48190D [Brassica napus]
Ach02g252381	C2 and GRAM domain-containing protein [Morus notabilis]
Ach14g452381	calcineurin B-like protein 01 [Vitis vinifera]
Ach23g347661	Callose synthase [Medicago truncatula]
Ach00g106331	CBS domain-containing protein CBSX6 [Morus notabilis]
Ach00g106311	cdc2Pnc [Pinus contorta]
Ach18g156851	cellulose synthase [Gossypium hirsutum]
Ach02g118311	cellulose synthase 2 [Betula luminifera]
Ach00g328741	chitin elicitor receptor kinase 1 [Chrysanthemum boreale]
Ach08g152591	Chlorophyll a-b binding protein CP29.2, chloroplastic [Glycine soja]
Ach27g297271	chloroplast carbonic anhydrase [Pachysandra terminalis]
Ach07g410341	cinnamoyl-CoA reductase, putative [Ricinus communis]
Ach00g281341	cinnamyl alcohol dehydrogenase [Gossypium hirsutum]
Ach00g222951	CLAVATA1 [Rhododendron ovatum]
Ach25g441641	clavata3/esr-related 12 family protein [Populus trichocarpa]
Ach23g465151	COL1-1 [Populus tomentosa]
Ach16g036001	conserved hypothetical protein [Ricinus communis]
Ach00g460841	conserved hypothetical protein [Ricinus communis]
Ach04g459731	conserved hypothetical protein [Ricinus communis]
Ach18g416731	conserved hypothetical protein [Ricinus communis]
Ach26g301101	conserved hypothetical protein [Ricinus communis]
Ach16g365191	Conserved oligomeric Golgi complex component, putative [Ricinus communis]
Ach00g328771	contains similarity to mouse and human SL15 proteins (GB:AF038961 and U41996) [Arabidopsis thaliana]
Ach22g337101	Core-2/I-branching beta-1,6-N-acetylglucosaminyltransferase family protein [Theobroma cacao]
Ach04g087501	cryptochrome 1 family protein [Populus trichocarpa]
Ach00g260181	cyclin [Camellia sinensis]
Ach15g002441	DEA(D/H)-box RNA helicase family protein isoform 1 [Theobroma cacao]
Ach23g176621	DEAD-box ATP-dependent RNA helicase 53 -like protein [Gossypium arboreum]
Ach01g285481	dehydration-responsive element-binding protein DREB3 [Solanum tuberosum]
Ach22g448581	delta-6-desaturase [Camellia sinensis]

Ach18g326151	Dihydrolipoyllysine-residue succinyltransferase component of 2-oxoglutarate dehydrogenase complex [Theobroma cacao]
Ach03g478681	Disease resistance family protein / LRR family protein, putative [Theobroma cacao]
Ach17g192101	Disease resistance protein [Morus notabilis]
Ach00g065871	D-isomer specific 2-hydroxyacid dehydrogenase family protein isoform 2 [Theobroma cacao]
Ach19g260401	disulfide isomerase-like protein [Viola biflora]
Ach00g011091	DNA binding protein, putative [Ricinus communis]
Ach01g235091	DNA binding protein, putative [Ricinus communis]
Ach21g095101	DNA binding protein, putative [Ricinus communis]
Ach25g316741	DNA binding protein, putative [Ricinus communis]
Ach29g195961	DNA binding protein, putative [Ricinus communis]
Ach13g349581	DNA-binding HORMA family protein [Theobroma cacao]
Ach00g260191	Dolichol-phosphate mannosyltransferase [Gossypium arboreum]
Ach24g082921	EF-hand calcium-binding domain-containing protein 4A [Theobroma cacao]
Ach00g132301	EG isoform 2 [Theobroma cacao]
Ach23g346881	EIN3-like protein EIL1 [Actinidia chinensis]
Ach23g347321	EIN3-like protein EIL2 [Actinidia chinensis]
Ach08g420691	elongation factor 1 alpha [Actinidia deliciosa]
Ach27g463331	Endo-1,3(4)-beta-glucanase 1 [Morus notabilis]
Ach03g267461	Endoplasmic reticulum retention defective 2B [Theobroma cacao]
Ach03g218031	ENTH/VHS family protein isoform 2 [Theobroma cacao]
Ach25g316771	ENTH/VHS/GAT family protein, putative [Theobroma cacao]
Ach27g423161	Esterase, putative [Theobroma cacao]
Ach23g359661	ethylene response factor 10 [Actinidia deliciosa]
Ach11g404811	F1N21.14 [Arabidopsis thaliana]
Ach08g283071	F22L4.1 protein, putative isoform 1 [Theobroma cacao]
Ach00g340081	F23A5.27 isoform 7 [Theobroma cacao]
Ach20g236041	fatty acid beta-oxidation multifunctional protein [Camellia oleifera]
Ach29g386861	fatty acid beta-oxidation multifunctional protein [Camellia oleifera]
Ach00g033341	F-box protein [Morus notabilis]
Ach00g126921	F-box/WD repeat-containing protein pof10 [Glycine soja]
Ach00g478351	ferric reductase oxidase [Manihot esculenta]
Ach01g373171	Formin [Theobroma cacao]
Ach15g377061	FRIGIDA-like protein isoform 1 [Theobroma cacao]
Ach08g040371	fructose-1,6-bisphosphatase [Camellia sinensis]
Ach15g044851	fructose-bisphosphate aldolase 2 [Camellia oleifera]
Ach22g190711	FT-like protein [Betula luminifera]
Ach11g125761	GA signaling F-Box [Actinidia deliciosa]
Ach00g164821	Galactosyltransferase family protein isoform 1 [Theobroma cacao]
Ach25g374471	GDSL-motif lipase/hydrolase 6 [Theobroma cacao]
Ach18g102241	General transcription factor IIH subunit 4 [Morus notabilis]

Ach05g254601	geranylgeranyl diphosphate synthase [Malus domestica]
Ach11g049971	GH1 protein [Medicago truncatula]
Ach00g047311	Global transcription factor group E4, putative isoform 1 [Theobroma cacao]
Ach00g452231	Glucose-1-phosphate adenylyltransferase [Theobroma cacao]
Ach24g203661	glutathione transferase, partial [Dimocarpus longan]
Ach04g343701	Glyceraldehyde-3-phosphate dehydrogenase B [Morus notabilis]
Ach13g352901	glycerate dehydrogenase, putative [Ricinus communis]
Ach20g092021	glycerol-3-phosphate acyltransferase [Camellia sinensis]
Ach18g326431	glycine decarboxylase complex T, partial [Populus fremontii]
Ach24g486361	Glycosyl hydrolase superfamily protein, putative [Theobroma cacao]
Ach03g188201	glycosyltransferase [Actinidia deliciosa]
Ach03g188221	glycosyltransferase [Actinidia deliciosa]
Ach00g444901	Haloacid dehalogenase-like hydrolase domain-containing protein 3 [Morus notabilis]
Ach27g088781	HAUS augmin-like complex subunit 6 [Theobroma cacao]
Ach15g184401	Heat shock protein 70 (Hsp 70) family protein [Theobroma cacao]
Ach12g071381	heat shock protein, putative [Ricinus communis]
Ach20g247541	Heat shock transcription factor A2 isoform 1 [Theobroma cacao]
Ach28g248151	hexokinase [Actinidia chinensis]
Ach24g041061	histone-like protein [Fritillaria liliacea]
Ach21g014781	Homeodomain-like superfamily protein, putative [Theobroma cacao]
Ach13g349391	HXXXD-type acyl-transferase family protein, putative [Theobroma cacao]
Ach13g349401	HXXXD-type acyl-transferase family protein, putative [Theobroma cacao]
Ach06g169691	Hydroxyproline-rich glycoprotein family protein, putative [Theobroma cacao]
Ach26g329101	hypothetical protein [Arabidopsis thaliana]
Ach26g107801	hypothetical protein [Camellia sinensis]
Ach04g120941	hypothetical protein [Trypanosoma brucei brucei TREU927]
Ach00g384801	hypothetical protein AALP_AA1G310700 [Arabis alpina]
Ach00g165161	hypothetical protein AALP_AA6G029600 [Arabis alpina]
Ach16g035801	hypothetical protein AMTR_s00049p00143510 [Amborella trichopoda]
Ach13g279051	hypothetical protein AMTR_s00059p00118280 [Amborella trichopoda]
Ach18g004031	hypothetical protein ARALYDRAFT_334284 [Arabidopsis lyrata subsp. lyrata]
Ach05g440311	hypothetical protein ARALYDRAFT_905093 [Arabidopsis lyrata subsp. lyrata]
Ach02g020711	hypothetical protein B456_001G268500 [Gossypium raimondii]
Ach07g309091	hypothetical protein B456_001G270800 [Gossypium raimondii]
Ach23g384961	hypothetical protein B456_002G104300 [Gossypium raimondii]

Ach19g133531	hypothetical protein B456_004G029400 [Gossypium raimondii]
Ach25g177631	hypothetical protein B456_004G122300 [Gossypium raimondii]
Ach26g241851	hypothetical protein B456_006G1295001, partial [Gossypium raimondii]
Ach24g320821	hypothetical protein B456_006G237100 [Gossypium raimondii]
Ach00g485391	hypothetical protein B456_007G028900 [Gossypium raimondii]
Ach13g229511	hypothetical protein B456_007G118500 [Gossypium raimondii]
Ach13g352351	hypothetical protein B456_007G145600 [Gossypium raimondii]
Ach25g181581	hypothetical protein B456_008G219400 [Gossypium raimondii]
Ach00g444911	hypothetical protein B456_009G257800 [Gossypium raimondii]
Ach13g454161	hypothetical protein B456_010G149700 [Gossypium raimondii]
Ach28g123911	hypothetical protein B456_011G289700 [Gossypium raimondii]
Ach25g452061	hypothetical protein B456_012G136700 [Gossypium raimondii]
Ach17g191891	hypothetical protein B456_013G104700 [Gossypium raimondii]
Ach02g472301	hypothetical protein BVRB_2g035470 [Beta vulgaris subsp. vulgaris]
Ach14g401531	hypothetical protein BVRB_3g052700 [Beta vulgaris subsp. vulgaris]
Ach25g438481	hypothetical protein CARUB_v10003614mg [Capsella rubella]
Ach19g382841	hypothetical protein CARUB_v10008779mg [Capsella rubella]
Ach00g008401	hypothetical protein CARUB_v10018904mg [Capsella rubella]
Ach11g262871	hypothetical protein CICLE_v10001133mg [Citrus clementina]
Ach00g480071	hypothetical protein CICLE_v10002081mg [Citrus clementina]
Ach00g447651	hypothetical protein CICLE_v10009013mg [Citrus clementina]
Ach00g317111	hypothetical protein CICLE_v10010241mg [Citrus clementina]
Ach00g471051	hypothetical protein CICLE_v10013049mg [Citrus clementina]
Ach03g431301	hypothetical protein CICLE_v10013449mg [Citrus clementina]
Ach10g037931	hypothetical protein CICLE_v10014116mg [Citrus clementina]
Ach29g163081	hypothetical protein CICLE_v10014399mg [Citrus clementina]
Ach15g345911	hypothetical protein CICLE_v10014675mg [Citrus clementina]
Ach00g162631	hypothetical protein CICLE_v10015182mg [Citrus clementina]
Ach13g307581	hypothetical protein CICLE_v10015544mg [Citrus clementina]
Ach23g347351	hypothetical protein CICLE_v10015833mg [Citrus clementina]
Ach12g353561	hypothetical protein CICLE_v10017336mg [Citrus clementina]
Ach02g118331	hypothetical protein CICLE_v10017383mg [Citrus clementina]
Ach15g002471	hypothetical protein CICLE_v10022388mg [Citrus clementina]
Ach26g033631	hypothetical protein CICLE_v10023895mg, partial [Citrus clementina]
Ach09g019741	hypothetical protein CICLE_v10028098mg [Citrus clementina]
Ach26g301081	hypothetical protein CICLE_v10028618mg [Citrus clementina]
Ach23g347251	hypothetical protein CICLE_v10028962mg [Citrus clementina]
Ach26g456951	hypothetical protein CICLE_v10033613mg [Citrus clementina]
Ach09g401331	hypothetical protein CISIN_1g0001713mg, partial [Citrus sinensis]
Ach04g197371	hypothetical protein CISIN_1g000269mg [Citrus sinensis]
Ach23g068881	hypothetical protein CISIN_1g0013342mg, partial [Citrus sinensis]
Ach04g316121	hypothetical protein CISIN_1g0017372mg, partial [Citrus sinensis]

Ach16g017061	hypothetical protein CISIN_1g001797mg [Citrus sinensis]
Ach27g088731	hypothetical protein CISIN_1g008853mg [Citrus sinensis]
Ach25g400161	hypothetical protein CISIN_1g012629mg [Citrus sinensis]
Ach23g417701	hypothetical protein CISIN_1g013528mg [Citrus sinensis]
Ach12g353641	hypothetical protein CISIN_1g015407mg [Citrus sinensis]
Ach09g012991	hypothetical protein CISIN_1g020899mg [Citrus sinensis]
Ach00g454871	hypothetical protein CISIN_1g022144mg [Citrus sinensis]
Ach15g392421	hypothetical protein CISIN_1g022953mg [Citrus sinensis]
Ach00g388681	hypothetical protein CISIN_1g023131mg [Citrus sinensis]
Ach24g082671	hypothetical protein CISIN_1g024406mg [Citrus sinensis]
Ach15g314071	hypothetical protein CISIN_1g025979mg [Citrus sinensis]
Ach19g080121	hypothetical protein CISIN_1g027488mg [Citrus sinensis]
Ach29g446851	hypothetical protein CISIN_1g0286151mg, partial [Citrus sinensis]
Ach14g308561	hypothetical protein CISIN_1g036137mg [Citrus sinensis]
Ach14g308571	hypothetical protein CISIN_1g036137mg [Citrus sinensis]
Ach00g067721	hypothetical protein CISIN_1g036584mg [Citrus sinensis]
Ach00g460861	hypothetical protein CISIN_1g040778mg [Citrus sinensis]
Ach03g218301	hypothetical protein Csa_2G405030 [Cucumis sativus]
Ach04g087511	hypothetical protein Csa_3G889690 [Cucumis sativus]
Ach00g335311	hypothetical protein Csa_7G039270 [Cucumis sativus]
Ach04g343381	hypothetical protein EUGRSUZ_F01826 [Eucalyptus grandis]
Ach00g333741	hypothetical protein EUGRSUZ_G02568 [Eucalyptus grandis]
Ach16g113211	hypothetical protein EUGRSUZ_J02959 [Eucalyptus grandis]
Ach00g477401	hypothetical protein EUGRSUZ_L00753 [Eucalyptus grandis]
Ach05g247671	hypothetical protein EUTSA_v100130050mg, partial [Eutrema salsugineum]
Ach15g314181	hypothetical protein F383_17776 [Gossypium arboreum]
Ach05g291841	hypothetical protein F383_29159 [Gossypium arboreum]
Ach23g359991	hypothetical protein F383_32015 [Gossypium arboreum]
Ach16g113061	hypothetical protein JCGZ_06216 [Jatropha curcas]
Ach05g374871	hypothetical protein JCGZ_09432 [Jatropha curcas]
Ach16g021771	hypothetical protein JCGZ_11473 [Jatropha curcas]
Ach13g035161	hypothetical protein JCGZ_15146 [Jatropha curcas]
Ach08g152361	hypothetical protein JCGZ_25039 [Jatropha curcas]
Ach03g218051	hypothetical protein JCGZ_26820 [Jatropha curcas]
Ach15g412031	hypothetical protein L484_001429 [Morus notabilis]
Ach00g354741	hypothetical protein L484_004031 [Morus notabilis]
Ach00g310861	hypothetical protein L484_005483 [Morus notabilis]
Ach00g452871	hypothetical protein L484_006581 [Morus notabilis]
Ach04g204931	hypothetical protein L484_006960 [Morus notabilis]
Ach03g358811	hypothetical protein L484_011867 [Morus notabilis]
Ach28g395481	hypothetical protein L484_012533 [Morus notabilis]
Ach04g087201	hypothetical protein L484_012746 [Morus notabilis]
Ach08g152401	hypothetical protein L484_015571 [Morus notabilis]

Ach01g094291	hypothetical protein L484_019588 [Morus notabilis]
Ach00g449411	hypothetical protein L484_020110 [Morus notabilis]
Ach25g268711	hypothetical protein L484_023064 [Morus notabilis]
Ach06g247081	hypothetical protein L484_024148 [Morus notabilis]
Ach25g374411	hypothetical protein L484_025845 [Morus notabilis]
Ach27g170111	hypothetical protein L484_026521 [Morus notabilis]
Ach13g458761	hypothetical protein M569_02206 [Genlisea aurea]
Ach00g312161	hypothetical protein M569_03369, partial [Genlisea aurea]
Ach05g198131	hypothetical protein M569_13482 [Genlisea aurea]
Ach05g198121	hypothetical protein MIMGU_mgv11b009377mg [Erythranthe guttata]
Ach05g456401	hypothetical protein MIMGU_mgv1a000477mg [Erythranthe guttata]
Ach00g061531	hypothetical protein MIMGU_mgv1a000743mg [Erythranthe guttata]
Ach10g306521	hypothetical protein MIMGU_mgv1a0017942mg, partial [Erythranthe guttata]
Ach00g461881	hypothetical protein MIMGU_mgv1a002450mg [Erythranthe guttata]
Ach10g270231	hypothetical protein MIMGU_mgv1a004873mg [Erythranthe guttata]
Ach00g360931	hypothetical protein MIMGU_mgv1a007287mg [Erythranthe guttata]
Ach00g462911	hypothetical protein MIMGU_mgv1a010403mg [Erythranthe guttata]
Ach04g396381	hypothetical protein MIMGU_mgv1a014700mg [Erythranthe guttata]
Ach00g018661	hypothetical protein MIMGU_mgv1a020792mg, partial [Erythranthe guttata]
Ach08g341801	hypothetical protein MIMGU_mgv1a020792mg, partial [Erythranthe guttata]
Ach22g016161	hypothetical protein MIMGU_mgv1a020792mg, partial [Erythranthe guttata]
Ach13g301841	hypothetical protein MIMGU_mgv1a0210682mg, partial [Erythranthe guttata]
Ach23g258341	hypothetical protein MIMGU_mgv1a025010mg [Erythranthe guttata]
Ach28g431941	hypothetical protein PHAVU_001G037800g [Phaseolus vulgaris]
Ach21g290371	hypothetical protein PHAVU_004G096500g [Phaseolus vulgaris]
Ach00g072141	hypothetical protein PHAVU_008G259000g [Phaseolus vulgaris]
Ach16g016951	hypothetical protein PHAVU_009G162500g [Phaseolus vulgaris]
Ach02g417101	hypothetical protein PHAVU_010G008000g [Phaseolus vulgaris]
Ach26g298081	hypothetical protein PHAVU_010G036200g [Phaseolus vulgaris]
Ach23g417691	hypothetical protein PHAVU_010G0831000g, partial [Phaseolus vulgaris]
Ach00g209591	hypothetical protein POPTR_0001s11300g [Populus trichocarpa]
Ach16g216301	hypothetical protein POPTR_0001s19250g, partial [Populus trichocarpa]
Ach27g089191	hypothetical protein POPTR_0001s35940g [Populus trichocarpa]
Ach00g298471	hypothetical protein POPTR_0001s40220g [Populus trichocarpa]
Ach23g163361	hypothetical protein POPTR_0001s41870g [Populus trichocarpa]
Ach24g202501	hypothetical protein POPTR_0001s44980g [Populus trichocarpa]

Ach17g439701	hypothetical protein POPTR_0002s04710g [Populus trichocarpa]
Ach24g407551	hypothetical protein POPTR_0003s08310g [Populus trichocarpa]
Ach15g005761	hypothetical protein POPTR_0003s13050g [Populus trichocarpa]
Ach13g349571	hypothetical protein POPTR_0003s17220g [Populus trichocarpa]
Ach00g449401	hypothetical protein POPTR_0005s13270g [Populus trichocarpa]
Ach10g038111	hypothetical protein POPTR_0005s13270g [Populus trichocarpa]
Ach13g352441	hypothetical protein POPTR_0005s24730g [Populus trichocarpa]
Ach17g132881	hypothetical protein POPTR_0006s02200g [Populus trichocarpa]
Ach00g252181	hypothetical protein POPTR_0006s130902g, partial [Populus trichocarpa]
Ach00g467001	hypothetical protein POPTR_0006s24590g [Populus trichocarpa]
Ach00g340221	hypothetical protein POPTR_0007s03340g [Populus trichocarpa]
Ach00g462901	hypothetical protein POPTR_0007s03340g [Populus trichocarpa]
Ach24g442521	hypothetical protein POPTR_0008s02980g [Populus trichocarpa]
Ach00g039781	hypothetical protein POPTR_0008s13160g [Populus trichocarpa]
Ach24g041101	hypothetical protein POPTR_0010s08780g [Populus trichocarpa]
Ach18g269221	hypothetical protein POPTR_0010s23810g [Populus trichocarpa]
Ach00g053341	hypothetical protein POPTR_0011s05250g [Populus trichocarpa]
Ach00g266311	hypothetical protein POPTR_0012s04950g, partial [Populus trichocarpa]
Ach16g365801	hypothetical protein POPTR_0012s07310g [Populus trichocarpa]
Ach12g395221	hypothetical protein POPTR_0013s06390g [Populus trichocarpa]
Ach04g456791	hypothetical protein POPTR_0013s12060g [Populus trichocarpa]
Ach05g396291	hypothetical protein POPTR_0015s06990g [Populus trichocarpa]
Ach00g148961	hypothetical protein POPTR_0016s11490g, partial [Populus trichocarpa]
Ach01g270721	hypothetical protein POPTR_0017s00430g [Populus trichocarpa]
Ach10g270371	hypothetical protein POPTR_0017s07570g [Populus trichocarpa]
Ach13g454171	hypothetical protein POPTR_0019s13970g [Populus trichocarpa]
Ach01g307961	hypothetical protein POPTR_0025s00390g [Populus trichocarpa]
Ach00g485741	hypothetical protein PRUPE_ppa000072mg [Prunus persica]
Ach17g133011	hypothetical protein PRUPE_ppa000077mg [Prunus persica]
Ach11g263821	hypothetical protein PRUPE_ppa000165mg [Prunus persica]
Ach21g290811	hypothetical protein PRUPE_ppa000257mg [Prunus persica]
Ach13g455561	hypothetical protein PRUPE_ppa000306mg [Prunus persica]
Ach00g480731	hypothetical protein PRUPE_ppa000317mg [Prunus persica]
Ach20g092571	hypothetical protein PRUPE_ppa000744mg [Prunus persica]
Ach13g279781	hypothetical protein PRUPE_ppa000832mg [Prunus persica]
Ach27g170631	hypothetical protein PRUPE_ppa001080mg [Prunus persica]
Ach24g041051	hypothetical protein PRUPE_ppa001134mg [Prunus persica]
Ach17g132911	hypothetical protein PRUPE_ppa001147mg [Prunus persica]
Ach08g237801	hypothetical protein PRUPE_ppa001251mg [Prunus persica]
Ach00g105011	hypothetical protein PRUPE_ppa001398mg [Prunus persica]
Ach15g380641	hypothetical protein PRUPE_ppa001447mg [Prunus persica]

Ach08g471891	hypothetical protein PRUPE_ppa001870mg [Prunus persica]
Ach19g138191	hypothetical protein PRUPE_ppa002021mg [Prunus persica]
Ach24g202241	hypothetical protein PRUPE_ppa002109mg [Prunus persica]
Ach15g134771	hypothetical protein PRUPE_ppa002193mg [Prunus persica]
Ach19g320331	hypothetical protein PRUPE_ppa002343mg [Prunus persica]
Ach22g273101	hypothetical protein PRUPE_ppa003182mg [Prunus persica]
Ach05g278101	hypothetical protein PRUPE_ppa005639mg [Prunus persica]
Ach03g354501	hypothetical protein PRUPE_ppa005739mg [Prunus persica]
Ach01g366701	hypothetical protein PRUPE_ppa005939mg [Prunus persica]
Ach06g182781	hypothetical protein PRUPE_ppa006204mg [Prunus persica]
Ach08g300531	hypothetical protein PRUPE_ppa006562mg [Prunus persica]
Ach29g130211	hypothetical protein PRUPE_ppa008552mg [Prunus persica]
Ach16g113031	hypothetical protein PRUPE_ppa008845mg [Prunus persica]
Ach23g320621	hypothetical protein PRUPE_ppa009851mg [Prunus persica]
Ach29g163091	hypothetical protein PRUPE_ppa010607mg [Prunus persica]
Ach07g240771	hypothetical protein PRUPE_ppa011181mg [Prunus persica]
Ach00g035621	hypothetical protein PRUPE_ppa011222mg [Prunus persica]
Ach00g382051	hypothetical protein PRUPE_ppa011455mg [Prunus persica]
Ach27g423171	hypothetical protein PRUPE_ppa011793mg [Prunus persica]
Ach00g289871	hypothetical protein PRUPE_ppa012939mg [Prunus persica]
Ach01g365491	hypothetical protein PRUPE_ppa013360mg [Prunus persica]
Ach00g076651	hypothetical protein PRUPE_ppa014774mg [Prunus persica]
Ach13g324681	hypothetical protein PRUPE_ppa015365mg, partial [Prunus persica]
Ach11g126061	hypothetical protein PRUPE_ppa015636mg [Prunus persica]
Ach18g479801	hypothetical protein PRUPE_ppa015873mg [Prunus persica]
Ach23g226171	hypothetical protein PRUPE_ppa016079mg [Prunus persica]
Ach20g091661	hypothetical protein PRUPE_ppa017791mg [Prunus persica]
Ach11g125361	hypothetical protein PRUPE_ppa018927mg, partial [Prunus persica]
Ach17g133051	hypothetical protein PRUPE_ppa020219mg [Prunus persica]
Ach00g375031	hypothetical protein PRUPE_ppa023217mg [Prunus persica]
Ach00g384831	hypothetical protein PRUPE_ppa023755mg [Prunus persica]
Ach17g009451	hypothetical protein PRUPE_ppa024063mg, partial [Prunus persica]
Ach14g278371	hypothetical protein PRUPE_ppa024550mg [Prunus persica]
Ach16g443551	hypothetical protein PRUPE_ppa024929mg [Prunus persica]
Ach15g002451	hypothetical protein PRUPE_ppa027142mg, partial [Prunus persica]
Ach03g354371	hypothetical protein PRUPE_ppa1027166mg [Prunus persica]
Ach28g085301	hypothetical protein RCOM_1590900 [Ricinus communis]
Ach19g097871	hypothetical protein SCLCIDRAFT_30966 [Scleroderma citrinum Foug A]
Ach22g016051	hypothetical protein SORBIDRAFT_01g050160 [Sorghum bicolor]
Ach22g016111	hypothetical protein SOVF_025630 isoform B [Spinacia oleracea]
Ach13g349981	hypothetical protein SOVF_077850 [Spinacia oleracea]
Ach05g452481	hypothetical protein SOVF_172130 [Spinacia oleracea]
Ach04g086911	hypothetical protein VITISV_000475 [Vitis vinifera]

Ach23g226261	hypothetical protein VITISV_001500 [Vitis vinifera]
Ach00g466991	hypothetical protein VITISV_001579 [Vitis vinifera]
Ach28g200121	hypothetical protein VITISV_002764 [Vitis vinifera]
Ach06g175791	hypothetical protein VITISV_002923 [Vitis vinifera]
Ach00g232101	hypothetical protein VITISV_002957 [Vitis vinifera]
Ach23g176611	hypothetical protein VITISV_002996 [Vitis vinifera]
Ach24g082591	hypothetical protein VITISV_003244 [Vitis vinifera]
Ach00g462251	hypothetical protein VITISV_004501 [Vitis vinifera]
Ach26g242111	hypothetical protein VITISV_006042 [Vitis vinifera]
Ach16g212491	hypothetical protein VITISV_007010 [Vitis vinifera]
Ach15g451021	hypothetical protein VITISV_007114 [Vitis vinifera]
Ach27g089391	hypothetical protein VITISV_008739 [Vitis vinifera]
Ach00g480161	hypothetical protein VITISV_008825 [Vitis vinifera]
Ach00g101221	hypothetical protein VITISV_009742 [Vitis vinifera]
Ach13g329511	hypothetical protein VITISV_010335 [Vitis vinifera]
Ach00g447881	hypothetical protein VITISV_011170 [Vitis vinifera]
Ach15g005581	hypothetical protein VITISV_013417 [Vitis vinifera]
Ach12g473391	hypothetical protein VITISV_013736 [Vitis vinifera]
Ach02g036821	hypothetical protein VITISV_014341 [Vitis vinifera]
Ach27g118121	hypothetical protein VITISV_015004 [Vitis vinifera]
Ach00g425651	hypothetical protein VITISV_015775 [Vitis vinifera]
Ach17g430571	hypothetical protein VITISV_016601 [Vitis vinifera]
Ach26g084591	hypothetical protein VITISV_016795 [Vitis vinifera]
Ach00g458681	hypothetical protein VITISV_017622 [Vitis vinifera]
Ach23g320431	hypothetical protein VITISV_018909 [Vitis vinifera]
Ach21g414701	hypothetical protein VITISV_019243 [Vitis vinifera]
Ach17g051431	hypothetical protein VITISV_020704 [Vitis vinifera]
Ach00g390291	hypothetical protein VITISV_020749 [Vitis vinifera]
Ach00g289081	hypothetical protein VITISV_022650 [Vitis vinifera]
Ach08g297701	hypothetical protein VITISV_025424 [Vitis vinifera]
Ach13g461441	hypothetical protein VITISV_026156 [Vitis vinifera]
Ach13g286871	hypothetical protein VITISV_026987 [Vitis vinifera]
Ach02g174991	hypothetical protein VITISV_027111 [Vitis vinifera]
Ach08g406161	hypothetical protein VITISV_032076 [Vitis vinifera]
Ach14g027111	hypothetical protein VITISV_032323 [Vitis vinifera]
Ach00g316021	hypothetical protein VITISV_033249 [Vitis vinifera]
Ach03g354511	hypothetical protein VITISV_034344 [Vitis vinifera]
Ach00g139321	hypothetical protein VITISV_034693 [Vitis vinifera]
Ach28g321461	hypothetical protein VITISV_036396 [Vitis vinifera]
Ach11g134501	hypothetical protein VITISV_036455 [Vitis vinifera]
Ach00g302941	hypothetical protein VITISV_040666 [Vitis vinifera]
Ach22g272791	hypothetical protein VITISV_042049 [Vitis vinifera]
Ach15g001331	hypothetical protein ZEAMMB73_061678 [Zea mays]
Ach26g426481	hypothetical protein ZEAMMB73_313798 [Zea mays]

Ach00g032911	hypothetical protein, partial [Aeromonas hydrophila]
Ach04g305291	hypothetical protein, partial [Paenibacillus sp. IHB B 3415]
Ach00g312171	Inactive TPR repeat-containing thioredoxin TTL3 [Morus notabilis]
Ach24g319351	iron superoxide dismutase 1a [Camellia sinensis]
Ach22g190741	JHL23J11.4 protein isoform 1 [Theobroma cacao]
Ach15g153811	Kinase superfamily protein [Theobroma cacao]
Ach26g107521	kiwellin [Actinidia arguta]
Ach00g455941	leucine-rich repeat protein [Citrus x microcarpa]
Ach00g131651	Leucine-rich repeat protein kinase family protein isoform 1 [Theobroma cacao]
Ach00g361621	leucoanthocyanidin dioxygenase [Actinidia chinensis]
Ach25g268931	lipoic acid synthetase, putative [Ricinus communis]
Ach09g171511	L-myo-inositol-1-phosphate synthase [Actinidia deliciosa]
Ach17g192111	LRR and NB-ARC domains-containing disease resistance protein, putative [Theobroma cacao]
Ach12g301571	Lysosomal Pro-X carboxypeptidase [Morus notabilis]
Ach00g324471	MADS-box domain protein [Camellia sinensis]
Ach13g279041	Major facilitator superfamily protein isoform 2 [Theobroma cacao]
Ach03g325801	Matrixin family protein, putative [Theobroma cacao]
Ach00g225611	Metalloendoproteinase 1 precursor, putative [Ricinus communis]
Ach01g270491	mildew resistance locus o 15 [Vitis vinifera]
Ach19g399041	Mitochondrial import receptor subunit TOM5 [Theobroma cacao]
Ach00g459961	Mitochondrial substrate carrier family protein W [Morus notabilis]
Ach25g080601	Mitogen-activated protein kinase 12 [Morus notabilis]
Ach28g304071	MLO-like protein 1 [Morus notabilis]
Ach05g193531	monovalent cation:proton antiporter, putative [Ricinus communis]
Ach03g244851	multidrug/pheromone exporter protein [Hevea brasiliensis]
Ach29g197141	Myb domain protein 17 isoform 1 [Theobroma cacao]
Ach24g313331	Myb domain protein 39, putative [Theobroma cacao]
Ach23g366791	MYB transcription factor [Paeonia suffruticosa]
Ach21g267001	Myosin heavy chain-related protein [Theobroma cacao]
Ach04g087291	Myosin heavy chain-related protein [Theobroma cacao]
Ach00g139311	NA
Ach00g143481	NA
Ach00g217371	NA
Ach00g222791	NA
Ach00g245971	NA
Ach00g246021	NA
Ach00g264711	NA
Ach00g328931	NA
Ach00g353291	NA
Ach00g420451	NA
Ach00g444361	NA
Ach00g470201	NA

Ach00g472431	NA
Ach00g477461	NA
Ach00g477741	NA
Ach00g480171	NA
Ach01g178521	NA
Ach01g178531	NA
Ach03g433481	NA
Ach04g418241	NA
Ach05g374821	NA
Ach05g449601	NA
Ach05g467761	NA
Ach06g362061	NA
Ach08g457061	NA
Ach13g457451	NA
Ach15g376631	NA
Ach15g412081	NA
Ach16g443151	NA
Ach22g016171	NA
Ach23g410161	NA
Ach24g204221	NA
Ach25g137261	NA
Ach25g425401	NA
Ach28g321591	NA
Ach28g409571	NA
Ach00g031831	NA
Ach00g146331	NA
Ach00g164811	NA
Ach00g165121	NA
Ach00g266381	NA
Ach00g310331	NA
Ach00g335301	NA
Ach00g375471	NA
Ach00g429991	NA
Ach00g430401	NA
Ach00g430421	NA
Ach00g434991	NA
Ach00g444291	NA
Ach00g445371	NA
Ach00g447871	NA
Ach00g447891	NA
Ach00g455931	NA
Ach00g457001	NA
Ach00g457131	NA
Ach00g459291	NA

Ach00g460851	NA
Ach00g462891	NA
Ach00g466351	NA
Ach00g466371	NA
Ach00g472681	NA
Ach00g474681	NA
Ach00g478011	NA
Ach01g406781	NA
Ach03g154371	NA
Ach03g244561	NA
Ach04g249431	NA
Ach05g048801	NA
Ach05g278231	NA
Ach05g307431	NA
Ach05g458481	NA
Ach06g436181	NA
Ach07g410301	NA
Ach07g410311	NA
Ach08g473681	NA
Ach09g400371	NA
Ach09g415661	NA
Ach10g270101	NA
Ach10g449811	NA
Ach11g275941	NA
Ach12g157151	NA
Ach12g167631	NA
Ach12g421441	NA
Ach13g018391	NA
Ach13g455551	NA
Ach14g027261	NA
Ach14g469121	NA
Ach14g469131	NA
Ach14g469141	NA
Ach14g469151	NA
Ach15g184461	NA
Ach15g184491	NA
Ach15g377301	NA
Ach15g430771	NA
Ach16g397041	NA
Ach17g009441	NA
Ach17g051621	NA
Ach17g133161	NA
Ach17g155441	NA
Ach17g272421	NA

Ach18g004511	NA
Ach18g032071	NA
Ach18g433991	NA
Ach19g137971	NA
Ach20g073341	NA
Ach20g270811	NA
Ach21g396541	NA
Ach22g015841	NA
Ach22g404601	NA
Ach22g415611	NA
Ach22g431891	NA
Ach23g114811	NA
Ach23g410961	NA
Ach24g442351	NA
Ach25g080671	NA
Ach25g080681	NA
Ach25g147431	NA
Ach25g409731	NA
Ach26g271231	NA
Ach26g402881	NA
Ach26g406601	NA
Ach26g426441	NA
Ach26g426451	NA
Ach29g054631	NA
Ach29g237211	NA
Ach29g386511	NA
Ach28g141961	NAC domain protein, IPR003441 [Theobroma cacao]
Ach00g464141	NAC domain-containing protein 8 [Glycine soja]
Ach00g456311	NAC transcription factor [Camellia sinensis]
Ach00g162311	NAD(P)-linked oxidoreductase superfamily protein [Theobroma cacao]
Ach00g311561	NADPH-cytochrome P450 reductase [Camptotheca acuminata]
Ach00g246001	Nascent polypeptide-associated complex subunit alpha-like protein [Morus notabilis]
Ach24g486371	NDR1/HIN1-like 8, putative isoform 1 [Theobroma cacao]
Ach00g345061	nerolidol synthase [Actinidia chinensis]
Ach26g107781	Nfrkb, putative isoform 5 [Theobroma cacao]
Ach03g010841	Nodulation receptor kinase precursor, putative [Ricinus communis]
Ach00g049641	NtN2 family protein [Populus trichocarpa]
Ach00g248441	Nucleic acid binding protein, putative [Theobroma cacao]
Ach04g428901	Nucleotide binding,nucleic acid binding [Theobroma cacao]
Ach00g485841	O-fucosyltransferase family protein [Theobroma cacao]
Ach13g320011	O-Glycosyl hydrolases family 17 protein isoform 2 [Theobroma cacao]

Ach00g008361	OleIV [Camellia oleifera]
Ach08g152531	omega-3 fatty acid desaturase [Camellia sinensis]
Ach19g260341	Os03g0831900 [Oryza sativa Japonica Group]
Ach00g031821	OSJNBb0003B01.26 [Oryza sativa Japonica Group]
Ach15g111491	Pectinesterase/pectinesterase inhibitor, putative [Theobroma cacao]
Ach08g105501	Pentatricopeptide repeat-containing protein [Morus notabilis]
Ach00g222911	peptidase M50 family protein [Populus trichocarpa]
Ach00g162391	phabulosa [Sarracenia purpurea]
Ach24g320921	Phosphatase 2C 70 [Theobroma cacao]
Ach25g374421	Phosphatidyl serine synthase family protein isoform 1 [Theobroma cacao]
Ach27g170781	Phosphoenolpyruvate carboxylase 4 [Glycine soja]
Ach19g305841	phosphoglycerate kinase [Gossypium hirsutum]
Ach00g104841	Phototropic-responsive NPH3 family protein isoform 1 [Theobroma cacao]
Ach06g332781	Plant basic secretory protein (BSP) family protein [Theobroma cacao]
Ach00g209471	P-loop containing nucleoside triphosphate hydrolases superfamily protein [Theobroma cacao]
Ach17g065121	Poly [ADP-ribose] polymerase-1 [Acetobacter malorum]
Ach04g459741	Polyadenylate-binding RBP47C -like protein [Gossypium arboreum]
Ach00g311491	PREDICTED: (DL)-glycerol-3-phosphatase 2 [Sesamum indicum]
Ach21g095451	PREDICTED: 1,4-alpha-glucan-branching enzyme 1, chloroplastic/amyloplastic isoform X1 [Vitis vinifera]
Ach26g108541	PREDICTED: 125 kDa kinesin-related protein [Vitis vinifera]
Ach00g382031	PREDICTED: 14-3-3-like protein GF14 kappa [Fragaria vesca subsp. vesca]
Ach02g036831	PREDICTED: 17.1 kDa class II heat shock protein-like [Pyrus x bretschneideri]
Ach00g164891	PREDICTED: 187-kDa microtubule-associated protein AIR9 isoform X4 [Vitis vinifera]
Ach22g015991	PREDICTED: 22.0 kDa class IV heat shock protein [Jatropha curcas]
Ach15g314081	PREDICTED: 26S protease regulatory subunit 6B homolog [Nicotiana tomentosiformis]
Ach25g291631	PREDICTED: 26S protease regulatory subunit 8 homolog A [Nelumbo nucifera]
Ach29g355751	PREDICTED: 26S proteasome non-ATPase regulatory subunit 10 [Vitis vinifera]
Ach00g375181	PREDICTED: 26S proteasome non-ATPase regulatory subunit 13 homolog A [Vitis vinifera]
Ach00g131931	PREDICTED: 3-ketoacyl-CoA synthase 4 [Nicotiana sylvestris]
Ach19g138181	PREDICTED: 40S ribosomal protein S19-3-like [Eucalyptus grandis]
Ach16g366291	PREDICTED: 40S ribosomal protein S5-2-like isoform X1 [Citrus sinensis]

Ach03g081471	PREDICTED: 4-coumarate--CoA ligase-like 10 [Nicotiana tomentosiformis]
Ach22g341461	PREDICTED: 4-hydroxy-tetrahydrodipicolinate reductase 1, chloroplastic [Vitis vinifera]
Ach25g291621	PREDICTED: 4-hydroxy-tetrahydrodipicolinate reductase 1, chloroplastic-like isoform X1 [Nelumbo nucifera]
Ach15g261221	PREDICTED: 50S ribosomal protein L1, chloroplastic-like [Populus euphratica]
Ach28g303081	PREDICTED: 50S ribosomal protein L17, chloroplastic [Nicotiana tomentosiformis]
Ach22g325311	PREDICTED: 50S ribosomal protein L3-2, chloroplastic [Vitis vinifera]
Ach23g171931	PREDICTED: 50S ribosomal protein L4, chloroplastic-like [Nelumbo nucifera]
Ach15g373511	PREDICTED: 5'-3' exoribonuclease 4 isoform X3 [Elaeis guineensis]
Ach13g472521	PREDICTED: 60S ribosomal protein L30-like [Nicotiana tomentosiformis]
Ach00g131921	PREDICTED: 60S ribosomal protein L6, mitochondrial [Cucumis sativus]
Ach27g353791	PREDICTED: 60S ribosomal protein L7a [Nelumbo nucifera]
Ach05g193521	PREDICTED: 60S ribosomal protein L7a [Nelumbo nucifera]
Ach07g309071	PREDICTED: 65-kDa microtubule-associated protein 8 [Vitis vinifera]
Ach14g189251	PREDICTED: 6-phosphogluconate dehydrogenase, decarboxylating 1, chloroplastic [Vitis vinifera]
Ach23g226121	PREDICTED: 7-dehydrocholesterol reductase-like [Malus domestica]
Ach15g154071	PREDICTED: ABC transporter B family member 1 [Vitis vinifera]
Ach06g232021	PREDICTED: ABC transporter B family member 9-like isoform X1 [Vitis vinifera]
Ach06g446741	PREDICTED: ABC transporter B family member 9-like isoform X1 [Vitis vinifera]
Ach15g184541	PREDICTED: ABC transporter C family member 10-like [Populus euphratica]
Ach26g260571	PREDICTED: ABC transporter G family member 14 [Vitis vinifera]
Ach03g187991	PREDICTED: ABC transporter G family member 8-like [Sesamum indicum]
Ach16g157661	PREDICTED: ABC transporter I family member 1 [Nicotiana tomentosiformis]
Ach20g098281	PREDICTED: abscisic acid 8'-hydroxylase 1-like [Sesamum indicum]
Ach13g307571	PREDICTED: aconitate hydratase, cytoplasmic [Nelumbo nucifera]
Ach00g307471	PREDICTED: actin-related protein 8 isoform X3 [Prunus mume]
Ach00g458701	PREDICTED: actin-related protein 8-like isoform X1 [Solanum tuberosum]

Ach03g185661	PREDICTED: acyl-CoA-binding domain-containing protein 4 [Prunus mume]
Ach20g159861	PREDICTED: acyl-protein thioesterase 2 isoform X1 [Vitis vinifera]
Ach16g309471	PREDICTED: adenine phosphoribosyltransferase 2-like isoform X2 [Malus domestica]
Ach15g002751	PREDICTED: adenine phosphoribosyltransferase 3 [Vitis vinifera]
Ach26g271101	PREDICTED: ADP-ribosylation factor GTPase-activating protein AGD3 isoform X2 [Erythranthe guttatus]
Ach03g081131	PREDICTED: ADP-ribosylation factor-related protein 1-like [Jatropha curcas]
Ach00g449441	PREDICTED: alanine--glyoxylate aminotransferase 2 homolog 1, mitochondrial [Nelumbo nucifera]
Ach25g409751	PREDICTED: aldo-keto reductase family 4 member C10-like [Malus domestica]
Ach24g319141	PREDICTED: aldo-keto reductase family 4 member C9 [Prunus mume]
Ach22g272821	PREDICTED: alkaline/neutral invertase CINV1-like [Vitis vinifera]
Ach29g356261	PREDICTED: alpha-1,4 glucan phosphorylase L-2 isozyme, chloroplastic/amyloplastic-like [Vitis vinifera]
Ach24g148061	PREDICTED: alpha-soluble NSF attachment protein-like [Erythranthe guttatus]
Ach14g237421	PREDICTED: aluminum-activated malate transporter 12-like [Sesamum indicum]
Ach14g188801	PREDICTED: aluminum-activated malate transporter 9-like [Jatropha curcas]
Ach24g082621	PREDICTED: anaphase-promoting complex subunit 7 [Vitis vinifera]
Ach23g116911	PREDICTED: anaphase-promoting complex subunit 8 [Vitis vinifera]
Ach23g116901	PREDICTED: anaphase-promoting complex subunit 8-like [Nelumbo nucifera]
Ach18g166231	PREDICTED: ankyrin repeat domain-containing protein 2 [Jatropha curcas]
Ach22g351171	PREDICTED: ankyrin repeat-containing protein At3g12360 [Vitis vinifera]
Ach23g115551	PREDICTED: ankyrin repeat-containing protein At3g12360-like [Vitis vinifera]
Ach00g266321	PREDICTED: ankyrin repeat-containing protein At5g02620-like [Elaeis guineensis]
Ach00g405161	PREDICTED: ankyrin-2-like isoform X2 [Vitis vinifera]
Ach16g021631	PREDICTED: AP-1 complex subunit mu-2 [Elaeis guineensis]
Ach26g068381	PREDICTED: AP-2 complex subunit alpha-1 [Vitis vinifera]
Ach00g354791	PREDICTED: AP2-like ethylene-responsive transcription factor ANT isoform X1 [Vitis vinifera]
Ach00g453871	PREDICTED: apoptosis-inducing factor 2 isoform X1 [Vitis vinifera]

Ach09g028721	PREDICTED: apoptotic chromatin condensation inducer in the nucleus-like isoform X1 [Populus euphratica]
Ach00g307511	PREDICTED: aquaporin TIP1-1 [Vitis vinifera]
Ach15g438091	PREDICTED: arabinogalactan peptide 20-like [Nelumbo nucifera]
Ach06g182791	PREDICTED: ARF guanine-nucleotide exchange factor GNOM [Jatropha curcas]
Ach03g403461	PREDICTED: ARF guanine-nucleotide exchange factor GNOM-like [Nelumbo nucifera]
Ach00g222321	PREDICTED: arginine N-methyltransferase 2 [Nicotiana tomentosiformis]
Ach12g192421	PREDICTED: aspartate aminotransferase, mitochondrial-like [Jatropha curcas]
Ach17g155041	PREDICTED: aspartate carbamoyltransferase, chloroplastic [Vitis vinifera]
Ach00g340001	PREDICTED: aspartate-semialdehyde dehydrogenase [Vitis vinifera]
Ach07g241021	PREDICTED: aspartate--tRNA ligase, mitochondrial [Nelumbo nucifera]
Ach10g270051	PREDICTED: aspartate--tRNA ligase, mitochondrial-like isoform X2 [Camelina sativa]
Ach10g270151	PREDICTED: aspartic proteinase nepenthesin-1 [Vitis vinifera]
Ach00g248511	PREDICTED: aspartic proteinase-like protein 2 [Prunus mume]
Ach01g379221	PREDICTED: ATP-dependent DNA helicase 2 subunit KU70 isoform X1 [Nicotiana sylvestris]
Ach22g415521	PREDICTED: ATP-dependent DNA helicase RRM3-like [Beta vulgaris subsp. vulgaris]
Ach00g353461	PREDICTED: ATP-dependent zinc metalloprotease FTSH 2, chloroplastic [Vitis vinifera]
Ach00g065311	PREDICTED: ATP-dependent zinc metalloprotease FTSH 3, mitochondrial-like [Brachypodium distachyon]
Ach21g095231	PREDICTED: AT-rich interactive domain-containing protein 5-like [Sesamum indicum]
Ach06g231151	PREDICTED: auxin response factor 1 [Prunus mume]
Ach20g073331	PREDICTED: auxin response factor 19-like [Vitis vinifera]
Ach05g053221	PREDICTED: auxin-induced protein 15A [Vitis vinifera]
Ach17g051581	PREDICTED: auxin-induced protein X15 [Vitis vinifera]
Ach28g070831	PREDICTED: auxin-responsive protein IAA11 isoform X1 [Vitis vinifera]
Ach15g227901	PREDICTED: B3 domain-containing protein At2g36080-like [Nelumbo nucifera]
Ach00g273601	PREDICTED: B3 domain-containing protein At3g19184-like isoform X1 [Sesamum indicum]
Ach21g014771	PREDICTED: B3 domain-containing protein REM16-like [Sesamum indicum]

Ach00g281921	PREDICTED: B3 domain-containing transcription factor NGA1-like [Sesamum indicum]
Ach14g278251	PREDICTED: B3 domain-containing transcription factor NGA1-like isoform X1 [Glycine max]
Ach03g081641	PREDICTED: BAG family molecular chaperone regulator 1 [Nicotiana tomentosiformis]
Ach15g002511	PREDICTED: BAG family molecular chaperone regulator 3-like [Nicotiana tomentosiformis]
Ach00g385731	PREDICTED: basic leucine zipper 61-like [Nelumbo nucifera]
Ach03g188121	PREDICTED: beta-carotene hydroxylase 2, chloroplastic [Vitis vinifera]
Ach29g446871	PREDICTED: bifunctional 3-dehydroquinate dehydratase/shikimate dehydrogenase, chloroplastic isoform X1 [Vitis vinifera]
Ach20g236251	PREDICTED: bifunctional 3-dehydroquinate dehydratase/shikimate dehydrogenase, chloroplastic isoform X2 [Vitis vinifera]
Ach15g002461	PREDICTED: bifunctional UDP-glucose 4-epimerase and UDP-xylose 4-epimerase 1 [Jatropha curcas]
Ach19g137931	PREDICTED: biotin carboxyl carrier protein of acetyl-CoA carboxylase 2, chloroplastic [Erythranthe guttatus]
Ach29g164601	PREDICTED: blue copper protein-like [Eucalyptus grandis]
Ach03g287911	PREDICTED: BRCA1-A complex subunit Abraxas [Jatropha curcas]
Ach22g059741	PREDICTED: bromodomain and WD repeat-containing protein 3 isoform X1 [Vitis vinifera]
Ach29g237111	PREDICTED: BSD domain-containing protein 1-like [Solanum tuberosum]
Ach26g068361	PREDICTED: BTB/POZ domain-containing protein At3g44820 [Vitis vinifera]
Ach14g278681	PREDICTED: BTB/POZ domain-containing protein POB1 isoform X1 [Nelumbo nucifera]
Ach14g188821	PREDICTED: BTB/POZ domain-containing protein POB1-like [Vitis vinifera]
Ach29g237101	PREDICTED: calcium-binding protein 39-like isoform X2 [Nelumbo nucifera]
Ach08g030821	PREDICTED: calcium-binding protein PBP1 [Vitis vinifera]
Ach15g184561	PREDICTED: calcium-dependent protein kinase 3-like [Nelumbo nucifera]
Ach07g341081	PREDICTED: calcium-dependent protein kinase 4 isoform X2 [Cucumis melo]
Ach23g172341	PREDICTED: calcium-transporting ATPase 4, endoplasmic reticulum-type-like [Brassica rapa]
Ach28g038291	PREDICTED: calcium-transporting ATPase 4, endoplasmic reticulum-type-like [Sesamum indicum]
Ach13g286881	PREDICTED: callose synthase 3-like [Sesamum indicum]
Ach17g133021	PREDICTED: callose synthase 7-like [Nicotiana tomentosiformis]

Ach17g133001	PREDICTED: callose synthase 7-like [Solanum tuberosum]
Ach13g279361	PREDICTED: calpain-type cysteine protease DEK1 [Vitis vinifera]
Ach22g015901	PREDICTED: calreticulin-3-like [Populus euphratica]
Ach20g452531	PREDICTED: carbonyl reductase [NADPH] 2-like [Eucalyptus grandis]
Ach26g457261	PREDICTED: casparian strip membrane protein 3-like [Solanum tuberosum]
Ach22g246411	PREDICTED: CASP-like protein 5B3 isoform X1 [Vitis vinifera]
Ach03g415281	PREDICTED: CASP-like protein RCOM_0464280-like [Citrus sinensis]
Ach00g433271	PREDICTED: CASP-like protein RCOM_1174750 [Prunus mume]
Ach15g314311	PREDICTED: cation/H(+) antiporter 14-like [Vitis vinifera]
Ach15g314321	PREDICTED: cation/H(+) antiporter 15-like [Pyrus x bretschneideri]
Ach05g193541	PREDICTED: cation/H(+) antiporter 15-like isoform X1 [Nicotiana tomentosiformis]
Ach15g184551	PREDICTED: cation/H(+) antiporter 19-like [Populus euphratica]
Ach28g303771	PREDICTED: cation/H(+) antiporter 24-like [Nicotiana sylvestris]
Ach29g386911	PREDICTED: cation/H(+) antiporter 27-like [Populus euphratica]
Ach18g003231	PREDICTED: cationic amino acid transporter 5-like [Prunus mume]
Ach22g029621	PREDICTED: cationic amino acid transporter 6, chloroplastic [Vitis vinifera]
Ach29g164731	PREDICTED: CBL-interacting serine/threonine-protein kinase 24 [Nelumbo nucifera]
Ach15g313991	PREDICTED: CBS domain-containing protein CBSX3, mitochondrial [Nicotiana tomentosiformis]
Ach09g028091	PREDICTED: CCR4-NOT transcription complex subunit 11 isoform X1 [Nelumbo nucifera]
Ach24g319131	PREDICTED: CCR4-NOT transcription complex subunit 1-like [Vitis vinifera]
Ach13g349921	PREDICTED: CCR4-NOT transcription complex subunit 3 isoform X1 [Vitis vinifera]
Ach03g045801	PREDICTED: CDPK-related kinase 4-like isoform X1 [Nicotiana sylvestris]
Ach00g361531	PREDICTED: CDT1-like protein a, chloroplastic [Nicotiana sylvestris]
Ach13g352781	PREDICTED: CDT1-like protein a, chloroplastic [Vitis vinifera]
Ach18g156861	PREDICTED: cellulose synthase A catalytic subunit 8 [UDP-forming]-like [Sesamum indicum]
Ach26g296941	PREDICTED: CENP-B homolog protein 2-like [Camelina sativa]
Ach11g451911	PREDICTED: centlein-like [Nicotiana tomentosiformis]
Ach26g241111	PREDICTED: centromere protein O isoform X3 [Nelumbo nucifera]
Ach22g443781	PREDICTED: chaperonin CPN60-2, mitochondrial isoform X1 [Gossypium raimondii]

Ach00g278781	PREDICTED: charged multivesicular body protein 5-like [Sesamum indicum]
Ach09g028731	PREDICTED: CID domain-containing protein 1 [Vitis vinifera]
Ach29g033211	PREDICTED: clavaminate synthase-like protein At3g21360 [Populus euphratica]
Ach13g388991	PREDICTED: CLIP-associated protein isoform X1 [Vitis vinifera]
Ach18g256921	PREDICTED: clustered mitochondria protein homolog [Vitis vinifera]
Ach19g199361	PREDICTED: coatomer subunit zeta-1-like [Nicotiana sylvestris]
Ach25g206731	PREDICTED: coiled-coil domain-containing protein 130-like [Populus euphratica]
Ach17g140101	PREDICTED: coiled-coil domain-containing protein 94 homolog isoform X2 [Beta vulgaris subsp. vulgaris]
Ach00g052591	PREDICTED: coilin isoform X3 [Vitis vinifera]
Ach16g113051	PREDICTED: coleoptile phototropism protein 1-like [Citrus sinensis]
Ach00g431781	PREDICTED: conserved oligomeric Golgi complex subunit 1 [Nicotiana tomentosiformis]
Ach25g466571	PREDICTED: conserved oligomeric Golgi complex subunit 1 [Nicotiana tomentosiformis]
Ach17g133041	PREDICTED: conserved oligomeric Golgi complex subunit 5 [Nelumbo nucifera]
Ach03g186241	PREDICTED: CONSTANS interacting protein 2b isoform X1 [Solanum lycopersicum]
Ach27g088721	PREDICTED: COP9 signalosome complex subunit 3 [Vitis vinifera]
Ach00g105101	PREDICTED: COP9 signalosome complex subunit 5b-like [Vitis vinifera]
Ach00g444271	PREDICTED: copper transporter 1-like [Fragaria vesca subsp. vesca]
Ach15g111501	PREDICTED: CRIB domain-containing protein RIC10 [Sesamum indicum]
Ach03g055081	PREDICTED: CTD nuclear envelope phosphatase 1 homolog [Erythranthe guttatus]
Ach08g360481	PREDICTED: C-terminal binding protein AN [Vitis vinifera]
Ach00g384691	PREDICTED: cyclic phosphodiesterase-like [Jatropha curcas]
Ach09g171321	PREDICTED: cyclin-D2-1 [Prunus mume]
Ach00g425711	PREDICTED: cyclin-D4-1-like isoform X1 [Jatropha curcas]
Ach05g048451	PREDICTED: cyclin-dependent kinase C-1-like [Vitis vinifera]
Ach29g431511	PREDICTED: cyclin-T1-3 [Vitis vinifera]
Ach11g397171	PREDICTED: cycloartenol-C-24-methyltransferase [Vitis vinifera]
Ach17g272411	PREDICTED: cytochrome P450 724B1 isoform X1 [Vitis vinifera]
Ach27g118111	PREDICTED: cytochrome P450 81D11 [Prunus mume]
Ach20g092461	PREDICTED: cytochrome P450 94C1-like [Nicotiana tomentosiformis]
Ach00g270961	PREDICTED: cytokinin dehydrogenase 7 [Jatropha curcas]
Ach00g270951	PREDICTED: cytokinin dehydrogenase 7 [Nelumbo nucifera]

Ach00g130981	PREDICTED: cytokinin riboside 5'-monophosphate phosphoribohydrolase LOG1-like [Sesamum indicum]
Ach00g281371	PREDICTED: D-3-phosphoglycerate dehydrogenase 3, chloroplastic [Nelumbo nucifera]
Ach19g305821	PREDICTED: DEAD-box ATP-dependent RNA helicase 10 [Cucumis sativus]
Ach10g189961	PREDICTED: DEAD-box ATP-dependent RNA helicase 3, chloroplastic [Vitis vinifera]
Ach00g248941	PREDICTED: DEAD-box ATP-dependent RNA helicase 38 [Solanum lycopersicum]
Ach29g197131	PREDICTED: DEAD-box ATP-dependent RNA helicase 8 [Nelumbo nucifera]
Ach06g183531	PREDICTED: DEAD-box ATP-dependent RNA helicase ISE2, chloroplastic [Vitis vinifera]
Ach19g306271	PREDICTED: DELLA protein RGL1-like [Fragaria vesca subsp. vesca]
Ach00g065981	PREDICTED: dentin sialophosphoprotein-like [Citrus sinensis]
Ach10g240651	PREDICTED: diacylglycerol kinase 7-like isoform X1 [Citrus sinensis]
Ach22g272691	PREDICTED: dihydrolipoyllysine-residue acetyltransferase component 1 of pyruvate dehydrogenase complex, mitochondrial [Vitis vinifera]
Ach22g325501	PREDICTED: DNA (cytosine-5)-methyltransferase DRM2 isoform X2 [Vitis vinifera]
Ach01g327771	PREDICTED: DNA mismatch repair protein MSH1, mitochondrial isoform X1 [Vitis vinifera]
Ach00g444851	PREDICTED: DNA replication licensing factor MCM6 [Vitis vinifera]
Ach24g141021	PREDICTED: DNA/RNA-binding protein KIN17 [Nicotiana sylvestris]
Ach24g431751	PREDICTED: DNA/RNA-binding protein KIN17 [Nicotiana sylvestris]
Ach00g151721	PREDICTED: DNA-directed RNA polymerase II subunit RPB2 [Beta vulgaris subsp. vulgaris]
Ach23g172431	PREDICTED: DNA-directed RNA polymerase III subunit rpc1 [Vitis vinifera]
Ach07g175321	PREDICTED: DNA-directed RNA polymerase V subunit 1 [Sesamum indicum]
Ach05g254591	PREDICTED: dolichyl-diphosphooligosaccharide--protein glycosyltransferase 48 kDa subunit isoform X2 [Vitis vinifera]
Ach26g077941	PREDICTED: dolichyl-diphosphooligosaccharide--protein glycosyltransferase subunit 2-like [Solanum tuberosum]
Ach00g008421	PREDICTED: dolichyl-diphosphooligosaccharide--protein glycosyltransferase subunit STT3B isoform X2 [Elaeis guineensis]

Ach01g088081	PREDICTED: double-stranded RNA-binding protein 4 isoform X3 [Vitis vinifera]
Ach27g339251	PREDICTED: double-stranded RNA-binding protein 4 isoform X3 [Vitis vinifera]
Ach07g241041	PREDICTED: DUF21 domain-containing protein At2g14520 [Vitis vinifera]
Ach03g186411	PREDICTED: DUF21 domain-containing protein At4g14240 [Vitis vinifera]
Ach14g308591	PREDICTED: dynamin-related protein 1E-like [Nelumbo nucifera]
Ach00g264351	PREDICTED: E3 SUMO-protein ligase SIZ1-like isoform X1 [Sesamum indicum]
Ach15g001801	PREDICTED: E3 ubiquitin-protein ligase At1g12760-like [Cucumis sativus]
Ach01g327921	PREDICTED: E3 ubiquitin-protein ligase At3g02290-like [Prunus mume]
Ach02g036131	PREDICTED: E3 ubiquitin-protein ligase Hakai [Vitis vinifera]
Ach26g271051	PREDICTED: E3 ubiquitin-protein ligase ORTHRUS 2-like [Nelumbo nucifera]
Ach15g005731	PREDICTED: E3 ubiquitin-protein ligase RHA1B [Nicotiana tomentosiformis]
Ach24g313381	PREDICTED: E3 ubiquitin-protein ligase RNF14 [Vitis vinifera]
Ach15g228061	PREDICTED: E3 ubiquitin-protein ligase RNF4-like [Nicotiana sylvestris]
Ach03g062581	PREDICTED: E3 ubiquitin-protein ligase SHPRH isoform X1 [Vitis vinifera]
Ach03g451731	PREDICTED: E3 ubiquitin-protein ligase synoviolin-like [Nelumbo nucifera]
Ach04g086981	PREDICTED: E3 ubiquitin-protein ligase UPL2-like [Vitis vinifera]
Ach00g403581	PREDICTED: E3 ubiquitin-protein ligase UPL7 isoform X1 [Vitis vinifera]
Ach19g134021	PREDICTED: early nodulin-like protein 2 [Nelumbo nucifera]
Ach29g164551	PREDICTED: EIN3-binding F-box protein 1-like [Vitis vinifera]
Ach00g328691	PREDICTED: endoglucanase 11-like [Jatropha curcas]
Ach23g024651	PREDICTED: endoglucanase 17 [Vitis vinifera]
Ach00g414611	PREDICTED: endoplasmic reticulum oxidoreductin-1-like [Cicer arietinum]
Ach00g384681	PREDICTED: endoplasmic reticulum oxidoreductin-1-like [Vitis vinifera]
Ach29g237091	PREDICTED: enhancer of mRNA-decapping protein 4-like [Nelumbo nucifera]
Ach15g377071	PREDICTED: epoxide hydrolase 3 [Jatropha curcas]
Ach22g451621	PREDICTED: ethylene-insensitive protein 2 [Vitis vinifera]
Ach26g329031	PREDICTED: ethylene-responsive transcription factor ERF003-like [Nelumbo nucifera]

Ach15g044861	PREDICTED: ethylene-responsive transcription factor ERF024-like [Pyrus x bretschneideri]
Ach15g002521	PREDICTED: ethylene-responsive transcription factor ERF026-like [Populus euphratica]
Ach28g142001	PREDICTED: ethylene-responsive transcription factor ERF098-like [Gossypium raimondii]
Ach16g366301	PREDICTED: ethylene-responsive transcription factor RAP2-11 [Vitis vinifera]
Ach24g140981	PREDICTED: eukaryotic translation initiation factor 3 subunit F [Cucumis melo]
Ach05g456381	PREDICTED: eukaryotic translation initiation factor 3 subunit M-like [Nelumbo nucifera]
Ach22g448501	PREDICTED: eukaryotic translation initiation factor isoform 4E-2 [Vitis vinifera]
Ach13g295641	PREDICTED: eukaryotic translation initiation factor isoform 4G-1-like [Sesamum indicum]
Ach03g218021	PREDICTED: exocyst complex component 7-like [Solanum tuberosum]
Ach00g106321	PREDICTED: exocyst complex component EXO70A1 [Vitis vinifera]
Ach03g444021	PREDICTED: exocyst complex component EXO70B1 [Solanum lycopersicum]
Ach02g292991	PREDICTED: exocyst complex component EXO70B1-like [Nicotiana sylvestris]
Ach03g218011	PREDICTED: exocyst complex component EXO70B1-like [Sesamum indicum]
Ach22g351151	PREDICTED: exocyst complex component EXO70B1-like [Vitis vinifera]
Ach03g267611	PREDICTED: exportin-1-like [Sesamum indicum]
Ach20g270821	PREDICTED: exportin-7 isoform X1 [Nelumbo nucifera]
Ach00g335221	PREDICTED: fasciclin-like arabinogalactan protein 9 [Nicotiana tomentosiformis]
Ach06g146921	PREDICTED: fatty acid amide hydrolase [Jatropha curcas]
Ach09g416501	PREDICTED: fatty acid amide hydrolase-like [Gossypium raimondii]
Ach03g471131	PREDICTED: F-box protein At5g07610-like [Citrus sinensis]
Ach00g446991	PREDICTED: F-box protein At5g49610-like isoform X1 [Nicotiana sylvestris]
Ach28g148861	PREDICTED: F-box/kelch-repeat protein At1g55270-like isoform X1 [Nicotiana tomentosiformis]
Ach15g425731	PREDICTED: F-box/kelch-repeat protein SKIP25-like [Sesamum indicum]
Ach00g031811	PREDICTED: F-box/kelch-repeat protein SKIP6 [Nicotiana sylvestris]
Ach15g376921	PREDICTED: F-box/LRR-repeat protein 12 [Sesamum indicum]
Ach02g482341	PREDICTED: F-box/LRR-repeat protein 3 [Solanum lycopersicum]

Ach28g142351	PREDICTED: F-box/LRR-repeat protein At4g14103-like isoform X2 [Vitis vinifera]
Ach09g012681	PREDICTED: F-box-like/WD repeat-containing protein TBL1XR1 isoform X1 [Vitis vinifera]
Ach15g184571	PREDICTED: fidgetin-like protein 1-like [Solanum tuberosum]
Ach09g416511	PREDICTED: filament-like plant protein [Vitis vinifera]
Ach15g376821	PREDICTED: fimbrin-2-like [Vitis vinifera]
Ach24g358551	PREDICTED: flap endonuclease GEN-like 2 [Vitis vinifera]
Ach28g085041	PREDICTED: flavonoid 3',5'-hydroxylase 2-like [Eucalyptus grandis]
Ach08g425941	PREDICTED: flavonol synthase/flavanone 3-hydroxylase-like [Citrus sinensis]
Ach13g279031	PREDICTED: flavonol synthase/flavanone 3-hydroxylase-like [Nicotiana sylvestris]
Ach15g364801	PREDICTED: floral homeotic protein APETALA 2-like [Nicotiana tomentosiformis]
Ach00g324411	PREDICTED: formate dehydrogenase, mitochondrial [Jatropha curcas]
Ach13g279111	PREDICTED: formimidoyltransferase-cyclodeaminase-like isoform X1 [Vitis vinifera]
Ach15g377041	PREDICTED: formin-like protein 1 [Solanum lycopersicum]
Ach28g409541	PREDICTED: FRIGIDA-like protein 3 [Vitis vinifera]
Ach20g424221	PREDICTED: fructose-bisphosphate aldolase cytoplasmic isozyme-like [Nelumbo nucifera]
Ach20g091731	PREDICTED: fructose-bisphosphate aldolase cytoplasmic isozyme-like [Sesamum indicum]
Ach23g121491	PREDICTED: galactoside 2-alpha-L-fucosyltransferase-like isoform X1 [Gossypium raimondii]
Ach23g429791	PREDICTED: galactoside 2-alpha-L-fucosyltransferase-like isoform X2 [Nelumbo nucifera]
Ach26g241731	PREDICTED: GATA transcription factor 18-like [Sesamum indicum]
Ach00g067301	PREDICTED: GDSL esterase/lipase At5g03980-like isoform X2 [Nicotiana tomentosiformis]
Ach02g436261	PREDICTED: GDT1-like protein 2, chloroplastic [Vitis vinifera]
Ach22g214561	PREDICTED: GDT1-like protein 2, chloroplastic [Vitis vinifera]
Ach15g001831	PREDICTED: general transcription factor IIE subunit 2-like [Nelumbo nucifera]
Ach15g002901	PREDICTED: gibberellin 20 oxidase 1 [Vitis vinifera]
Ach22g325591	PREDICTED: gibberellin 2-beta-dioxygenase 2 [Jatropha curcas]
Ach04g343721	PREDICTED: glucan endo-1,3-beta-glucosidase 12 [Jatropha curcas]
Ach13g349931	PREDICTED: glucan endo-1,3-beta-glucosidase 8 [Sesamum indicum]
Ach26g298011	PREDICTED: glucuronoxylan 4-O-methyltransferase 1 [Vitis vinifera]

Ach04g343251	PREDICTED: glutamate receptor 3.3 isoform X1 [Nicotiana sylvestris]
Ach00g305011	PREDICTED: glutamic acid-rich protein [Vitis vinifera]
Ach24g082421	PREDICTED: glutamine synthetase leaf isozyme, chloroplastic [Cucumis melo]
Ach10g257071	PREDICTED: glutamine-dependent NAD(+) synthetase [Populus euphratica]
Ach03g218041	PREDICTED: glutaredoxin-C5-like [Prunus mume]
Ach22g214751	PREDICTED: glutathione reductase, cytosolic [Vitis vinifera]
Ach00g127861	PREDICTED: glutathione S-transferase omega-like 2 [Jatropha curcas]
Ach13g195271	PREDICTED: glutelin type-B 5-like [Sesamum indicum]
Ach27g088881	PREDICTED: glycerol-3-phosphate dehydrogenase [NAD(+)] [Vitis vinifera]
Ach00g400961	PREDICTED: glycine cleavage system H protein 2, mitochondrial [Vitis vinifera]
Ach25g181591	PREDICTED: glycine-rich protein A3-like isoform X1 [Nelumbo nucifera]
Ach15g134981	PREDICTED: glycosyltransferase-like At2g41451 [Sesamum indicum]
Ach23g427641	PREDICTED: glyoxylate/succinic semialdehyde reductase 2, chloroplastic [Prunus mume]
Ach00g239741	PREDICTED: growth-regulating factor 3 [Vitis vinifera]
Ach20g213561	PREDICTED: grpE protein homolog, mitochondrial [Prunus mume]
Ach03g185671	PREDICTED: GTP-binding protein OBGC, chloroplastic isoform X3 [Vitis vinifera]
Ach13g017911	PREDICTED: G-type lectin S-receptor-like serine/threonine-protein kinase SD2-5 [Vitis vinifera]
Ach00g464151	PREDICTED: guanylate-binding protein 3-like [Camelina sativa]
Ach08g264971	PREDICTED: H/ACA ribonucleoprotein complex subunit 4 [Jatropha curcas]
Ach14g308491	PREDICTED: haloacid dehalogenase-like hydrolase domain-containing protein Sgpp [Malus domestica]
Ach14g308501	PREDICTED: haloacid dehalogenase-like hydrolase domain-containing protein Sgpp [Nicotiana tomentosiformis]
Ach19g199021	PREDICTED: heat shock 70 kDa protein 14-like [Sesamum indicum]
Ach03g188131	PREDICTED: heat shock protein 83 [Cucumis melo]
Ach03g478721	PREDICTED: heat stress transcription factor B-2a-like [Sesamum indicum]
Ach00g282551	PREDICTED: heavy metal-associated isoprenylated plant protein 26 [Vitis vinifera]
Ach26g107541	PREDICTED: helicase protein MOM1 isoform X1 [Vitis vinifera]
Ach00g222961	PREDICTED: hemK methyltransferase family member 2 [Nelumbo nucifera]

Ach01g365591	PREDICTED: heterogeneous nuclear ribonucleoprotein 1 [Jatropha curcas]
Ach23g427651	PREDICTED: heterogeneous nuclear ribonucleoprotein 1 [Prunus mume]
Ach08g425931	PREDICTED: heterogeneous nuclear ribonucleoprotein 1 [Vitis vinifera]
Ach11g275741	PREDICTED: hevamine-A-like [Gossypium raimondii]
Ach00g471021	PREDICTED: high affinity nitrate transporter 2.7-like isoform X1 [Solanum tuberosum]
Ach08g152391	PREDICTED: high mobility group B protein 1-like [Nelumbo nucifera]
Ach00g237481	PREDICTED: histidine kinase 4 isoform X2 [Vitis vinifera]
Ach25g098661	PREDICTED: histidinol-phosphate aminotransferase, chloroplastic-like [Pyrus x bretschneideri]
Ach23g226151	PREDICTED: histone acetyltransferase HAC1-like [Vitis vinifera]
Ach00g344101	PREDICTED: histone H1 [Vitis vinifera]
Ach11g126071	PREDICTED: homeobox-leucine zipper protein HAT22 isoform X3 [Vitis vinifera]
Ach15g002881	PREDICTED: homeobox-leucine zipper protein HAT4-like [Jatropha curcas]
Ach15g184681	PREDICTED: hydroquinone glucosyltransferase-like [Nelumbo nucifera]
Ach16g113331	PREDICTED: hydroxyphenylpyruvate reductase-like [Nicotiana sylvestris]
Ach13g352341	PREDICTED: hyoscyamine 6-dioxygenase [Vitis vinifera]
Ach13g464661	PREDICTED: hyoscyamine 6-dioxygenase [Vitis vinifera]
Ach25g409761	PREDICTED: IAA-alanine resistance protein 1 [Prunus mume]
Ach07g458861	PREDICTED: importin subunit alpha-1 [Solanum lycopersicum]
Ach05g254471	PREDICTED: indole-3-pyruvate monooxygenase YUCCA6-like [Vitis vinifera]
Ach00g285661	PREDICTED: inner membrane protein PPF-1, chloroplastic [Nicotiana sylvestris]
Ach22g190651	PREDICTED: INO80 complex subunit D-like [Sesamum indicum]
Ach05g440361	PREDICTED: inositol hexakisphosphate and diphosphoinositol-pentakisphosphate kinase 2-like isoform X1 [Citrus sinensis]
Ach16g216311	PREDICTED: inositol oxygenase 1-like isoform X2 [Erythranthe guttatus]
Ach04g197741	PREDICTED: inositol-pentakisphosphate 2-kinase-like [Vitis vinifera]
Ach15g134761	PREDICTED: integrator complex subunit 9 homolog [Prunus mume]
Ach00g344711	PREDICTED: interferon-induced guanylate-binding protein 2 [Vitis vinifera]
Ach15g220221	PREDICTED: interferon-induced guanylate-binding protein 2 [Vitis vinifera]

Ach15g314141	PREDICTED: intracellular protein transport protein USO1 [Nicotiana sylvestris]
Ach09g012981	PREDICTED: isoflavone 2'-hydroxylase-like isoform X1 [Prunus mume]
Ach27g118141	PREDICTED: isoflavone 2'-hydroxylase-like isoform X1 [Prunus mume]
Ach27g339861	PREDICTED: kanadaptin [Vitis vinifera]
Ach00g367481	PREDICTED: ketol-acid reductoisomerase, chloroplastic [Prunus mume]
Ach23g397351	PREDICTED: kinesin KP1 isoform X1 [Vitis vinifera]
Ach00g461831	PREDICTED: kinesin-like protein KCA2 [Vitis vinifera]
Ach24g407861	PREDICTED: kinesin-like protein KIF22 isoform X2 [Nicotiana sylvestris]
Ach05g270021	PREDICTED: kinesin-like protein NACK1 [Vitis vinifera]
Ach06g247091	PREDICTED: kinetochore protein ndc80-like [Vitis vinifera]
Ach25g364671	PREDICTED: kirola-like [Erythranthe guttatus]
Ach29g130131	PREDICTED: lactoylglutathione lyase [Solanum lycopersicum]
Ach15g044441	PREDICTED: L-aminoadipate-semialdehyde dehydrogenase-phosphopantetheinyl transferase isoform X2 [Vitis vinifera]
Ach04g087921	PREDICTED: lariat debranching enzyme isoform X1 [Vitis vinifera]
Ach15g228041	PREDICTED: L-ascorbate oxidase-like [Vitis vinifera]
Ach00g324081	PREDICTED: lecithin-cholesterol acyltransferase-like 4 [Vitis vinifera]
Ach24g034101	PREDICTED: LETM1 and EF-hand domain-containing protein 1, mitochondrial isoform X1 [Populus euphratica]
Ach22g439201	PREDICTED: LETM1 and EF-hand domain-containing protein 1, mitochondrial-like isoform X1 [Nelumbo nucifera]
Ach17g173081	PREDICTED: lipoyl synthase 2, mitochondrial-like [Nicotiana sylvestris]
Ach24g442361	PREDICTED: LOB domain-containing protein 1-like [Populus euphratica]
Ach00g104851	PREDICTED: LOB domain-containing protein 37-like [Nicotiana tomentosiformis]
Ach22g351181	PREDICTED: LOW QUALITY PROTEIN: ankyrin repeat-containing protein At3g12360-like [Sesamum indicum]
Ach05g047671	PREDICTED: LOW QUALITY PROTEIN: dnaJ homolog subfamily C member 21 [Cucumis melo]
Ach27g338631	PREDICTED: LOW QUALITY PROTEIN: protein GAMETE EXPRESSED 3 [Vitis vinifera]
Ach00g405151	PREDICTED: LOW QUALITY PROTEIN: subtilisin-like protease SBT3.5 [Vitis vinifera]
Ach28g409531	PREDICTED: LOW QUALITY PROTEIN: uncharacterized protein LOC100242929 [Vitis vinifera]
Ach07g110581	PREDICTED: LOW QUALITY PROTEIN: uncharacterized protein LOC100265010 [Vitis vinifera]

Ach16g426851	PREDICTED: LOW QUALITY PROTEIN: uncharacterized protein LOC103328018 [Prunus mume]
Ach21g446031	PREDICTED: lysine-specific histone demethylase 1 homolog 3 [Vitis vinifera]
Ach15g002501	PREDICTED: lysophospholipid acyltransferase 1-like [Pyrus x bretschneideri]
Ach07g458871	PREDICTED: mannosyl-oligosaccharide glucosidase GCS1 [Vitis vinifera]
Ach23g115561	PREDICTED: mavicyanin [Gossypium raimondii]
Ach00g289621	PREDICTED: mediator of RNA polymerase II transcription subunit 15a isoform X2 [Nelumbo nucifera]
Ach28g409561	PREDICTED: mediator of RNA polymerase II transcription subunit 19a-like isoform X1 [Eucalyptus grandis]
Ach28g304051	PREDICTED: mediator of RNA polymerase II transcription subunit 34-like isoform X1 [Nicotiana tomentosiformis]
Ach16g212471	PREDICTED: mediator of RNA polymerase II transcription subunit 8 isoform X2 [Jatropha curcas]
Ach15g445551	PREDICTED: membrane protein of ER body 1-like [Populus euphratica]
Ach00g076771	PREDICTED: metal tolerance protein 11 isoform X1 [Vitis vinifera]
Ach03g325781	PREDICTED: metalloendoproteinase 1-like [Vitis vinifera]
Ach03g325791	PREDICTED: metalloendoproteinase 1-like [Vitis vinifera]
Ach03g081561	PREDICTED: methyl-CpG-binding domain-containing protein 13 [Malus domestica]
Ach08g297751	PREDICTED: methylsterol monooxygenase 1-1-like [Sesamum indicum]
Ach22g272771	PREDICTED: methyltransferase-like protein 13 [Vitis vinifera]
Ach11g125751	PREDICTED: MFP1 attachment factor 1-like [Vitis vinifera]
Ach08g283241	PREDICTED: mitochondrial adenine nucleotide transporter ADNT1-like [Nelumbo nucifera]
Ach00g468671	PREDICTED: mitochondrial amidoxime-reducing component 1 isoform X4 [Nelumbo nucifera]
Ach26g300991	PREDICTED: mitochondrial fission protein ELM1 [Sesamum indicum]
Ach11g126211	PREDICTED: mitochondrial GTPase 1-like [Eucalyptus grandis]
Ach05g235521	PREDICTED: mitochondrial import receptor subunit TOM20 [Vitis vinifera]
Ach28g303061	PREDICTED: mitochondrial import receptor subunit TOM40-1-like [Nicotiana sylvestris]
Ach27g088871	PREDICTED: mitochondrial inner membrane protease ATP23 [Nelumbo nucifera]
Ach13g472511	PREDICTED: mitochondrial outer membrane import complex protein METAXIN [Sesamum indicum]

Ach15g002101	PREDICTED: mitochondrial substrate carrier family protein B-like [Sesamum indicum]
Ach00g074341	PREDICTED: mitogen-activated protein kinase homolog MMK2 [Vitis vinifera]
Ach14g472811	PREDICTED: mitogen-activated protein kinase kinase kinase 1-like [Solanum tuberosum]
Ach00g457091	PREDICTED: mitogen-activated protein kinase kinase kinase YODA isoform X2 [Prunus mume]
Ach03g185771	PREDICTED: monothiol glutaredoxin-S10 [Vitis vinifera]
Ach02g118511	PREDICTED: MORC family CW-type zinc finger protein 3-like isoform X2 [Vitis vinifera]
Ach22g272701	PREDICTED: mRNA cap guanine-N7 methyltransferase 2 [Sesamum indicum]
Ach06g247161	PREDICTED: mRNA turnover protein 4 homolog [Vitis vinifera]
Ach27g088831	PREDICTED: muscle M-line assembly protein unc-89 [Vitis vinifera]
Ach07g240751	PREDICTED: myb-like protein X [Nicotiana tomentosiformis]
Ach05g307261	PREDICTED: myb-related protein 315 [Vitis vinifera]
Ach24g203651	PREDICTED: myosin heavy chain, non-muscle [Vitis vinifera]
Ach23g163821	PREDICTED: myosin-2-like [Erythranthe guttatus]
Ach24g202311	PREDICTED: myosin-9-like [Vitis vinifera]
Ach26g395721	PREDICTED: NAC domain-containing protein 21/22-like [Solanum lycopersicum]
Ach17g009471	PREDICTED: NAC domain-containing protein 67-like [Prunus mume]
Ach14g237291	PREDICTED: NAC domain-containing protein 78-like [Citrus sinensis]
Ach00g457111	PREDICTED: NAC domain-containing protein 78-like isoform X2 [Solanum tuberosum]
Ach15g219211	PREDICTED: NAC domain-containing protein 8 isoform X2 [Vitis vinifera]
Ach17g132951	PREDICTED: N-acetyl-D-glucosamine kinase [Vitis vinifera]
Ach03g185951	PREDICTED: NADH dehydrogenase [ubiquinone] 1 alpha subcomplex subunit 8-B [Eucalyptus grandis]
Ach05g445711	PREDICTED: NADH dehydrogenase [ubiquinone] complex I, assembly factor 7 isoform X1 [Solanum lycopersicum]
Ach09g028691	PREDICTED: NADPH-dependent 1-acyldihydroxyacetone phosphate reductase [Vitis vinifera]
Ach03g186251	PREDICTED: NEDD8-activating enzyme E1 catalytic subunit-like [Citrus sinensis]
Ach13g308881	PREDICTED: NF-kappa-B-activating protein [Jatropha curcas]
Ach27g322391	PREDICTED: nicotinate-nucleotide pyrophosphorylase [carboxylating], chloroplastic isoform X2 [Nelumbo nucifera]
Ach24g202881	PREDICTED: nitrilase-like protein 2 [Vitis vinifera]
Ach03g239861	PREDICTED: nodulation receptor kinase [Nicotiana tomentosiformis]

Ach19g306261	PREDICTED: nuclear cap-binding protein subunit 1 [Vitis vinifera]
Ach16g113451	PREDICTED: nuclear pore complex protein NUP50A [Vitis vinifera]
Ach18g003161	PREDICTED: nuclear transcription factor Y subunit A-7 [Vitis vinifera]
Ach13g350801	PREDICTED: nuclear transcription factor Y subunit gamma [Eucalyptus grandis]
Ach00g345681	PREDICTED: nucleobase-ascorbate transporter 2 [Erythranthe guttatus]
Ach26g241721	PREDICTED: nucleolar complex protein 2 homolog isoform X3 [Vitis vinifera]
Ach03g354521	PREDICTED: nucleolin-like isoform X2 [Nelumbo nucifera]
Ach00g315981	PREDICTED: nucleoside diphosphate kinase 3-like [Nicotiana sylvestris]
Ach27g170351	PREDICTED: nudix hydrolase 1 [Nicotiana tomentosiformis]
Ach26g213341	PREDICTED: nudix hydrolase 3 [Vitis vinifera]
Ach22g059341	PREDICTED: ocs element-binding factor 1-like [Sesamum indicum]
Ach28g304061	PREDICTED: oligoribonuclease-like [Nicotiana sylvestris]
Ach17g253221	PREDICTED: omega-hydroxypalmitate O-feruloyl transferase [Vitis vinifera]
Ach19g306251	PREDICTED: organic cation/carnitine transporter 3-like [Nelumbo nucifera]
Ach23g226161	PREDICTED: organic cation/carnitine transporter 7 [Vitis vinifera]
Ach07g006711	PREDICTED: organ-specific protein P4 [Eucalyptus grandis]
Ach26g301001	PREDICTED: OTU domain-containing protein 5 [Vitis vinifera]
Ach00g311871	PREDICTED: OTU domain-containing protein DDB_G0284757-like isoform X2 [Nicotiana tomentosiformis]
Ach22g059321	PREDICTED: outer envelope pore protein 16-2, chloroplastic [Vitis vinifera]
Ach09g401421	PREDICTED: outer envelope pore protein 24A, chloroplastic-like [Nelumbo nucifera]
Ach00g126971	PREDICTED: paired amphipathic helix protein Sin3-like 3 isoform X3 [Vitis vinifera]
Ach06g183401	PREDICTED: paired amphipathic helix protein Sin3-like 4 isoform X3 [Musa acuminata subsp. malaccensis]
Ach06g247071	PREDICTED: partner of Y14 and mago-like [Solanum tuberosum]
Ach00g052561	PREDICTED: patatin-like protein 6 [Vitis vinifera]
Ach03g287021	PREDICTED: patellin-6 [Vitis vinifera]
Ach07g240741	PREDICTED: pathogenesis-related leaf protein 6 [Vitis vinifera]
Ach18g003501	PREDICTED: pentatricopeptide repeat-containing protein At1g08610 [Vitis vinifera]
Ach10g306501	PREDICTED: pentatricopeptide repeat-containing protein At1g30610, chloroplastic [Nelumbo nucifera]
Ach20g098241	PREDICTED: pentatricopeptide repeat-containing protein At1g31790 [Vitis vinifera]

Ach00g082311	PREDICTED: pentatricopeptide repeat-containing protein At1g61870, mitochondrial [Nelumbo nucifera]
Ach24g486381	PREDICTED: pentatricopeptide repeat-containing protein At1g63330 [Vitis vinifera]
Ach01g088321	PREDICTED: pentatricopeptide repeat-containing protein At1g77360, mitochondrial-like [Sesamum indicum]
Ach18g418961	PREDICTED: pentatricopeptide repeat-containing protein At2g13600-like [Vitis vinifera]
Ach15g154101	PREDICTED: pentatricopeptide repeat-containing protein At2g32630-like [Nicotiana tomentosiformis]
Ach20g447381	PREDICTED: pentatricopeptide repeat-containing protein At2g36980, mitochondrial [Vitis vinifera]
Ach22g190761	PREDICTED: pentatricopeptide repeat-containing protein At3g02650, mitochondrial [Nelumbo nucifera]
Ach15g377271	PREDICTED: pentatricopeptide repeat-containing protein At3g07290, mitochondrial isoform X1 [Vitis vinifera]
Ach19g483751	PREDICTED: pentatricopeptide repeat-containing protein At3g16010 [Vitis vinifera]
Ach15g109211	PREDICTED: pentatricopeptide repeat-containing protein At3g53700, chloroplastic [Nicotiana tomentosiformis]
Ach15g109191	PREDICTED: pentatricopeptide repeat-containing protein At3g53700, chloroplastic [Sesamum indicum]
Ach00g223061	PREDICTED: pentatricopeptide repeat-containing protein At3g57430, chloroplastic [Vitis vinifera]
Ach22g273071	PREDICTED: pentatricopeptide repeat-containing protein At4g02750-like [Vitis vinifera]
Ach13g308951	PREDICTED: pentatricopeptide repeat-containing protein At4g02820, mitochondrial isoform X1 [Vitis vinifera]
Ach14g269741	PREDICTED: pentatricopeptide repeat-containing protein At4g17616 [Vitis vinifera]
Ach05g254461	PREDICTED: pentatricopeptide repeat-containing protein At4g36680, mitochondrial-like [Nicotiana tomentosiformis]
Ach15g364781	PREDICTED: pentatricopeptide repeat-containing protein At5g02830, chloroplastic [Nicotiana sylvestris]
Ach22g029631	PREDICTED: pentatricopeptide repeat-containing protein At5g04780 [Vitis vinifera]
Ach05g193401	PREDICTED: pentatricopeptide repeat-containing protein At5g14080 [Vitis vinifera]
Ach27g353721	PREDICTED: pentatricopeptide repeat-containing protein At5g14080 isoform X1 [Prunus mume]
Ach05g440151	PREDICTED: pentatricopeptide repeat-containing protein At5g14080 isoform X2 [Prunus mume]
Ach26g296881	PREDICTED: pentatricopeptide repeat-containing protein At5g16860 [Prunus mume]

Ach22g351071	PREDICTED: pentatricopeptide repeat-containing protein At5g42450, mitochondrial [Vitis vinifera]
Ach16g017051	PREDICTED: pentatricopeptide repeat-containing protein At5g61990, mitochondrial [Jatropha curcas]
Ach23g320421	PREDICTED: peptidyl-prolyl cis-trans isomerase [Eucalyptus grandis]
Ach08g381581	PREDICTED: peptidyl-prolyl cis-trans isomerase CYP22 isoform X2 [Cucumis sativus]
Ach28g200131	PREDICTED: peptidyl-prolyl cis-trans isomerase CYP71 isoform X1 [Jatropha curcas]
Ach20g159851	PREDICTED: peptidyl-prolyl cis-trans isomerase CYP95-like isoform X1 [Cicer arietinum]
Ach26g297051	PREDICTED: peroxidase 31-like [Eucalyptus grandis]
Ach28g304091	PREDICTED: peroxisomal membrane protein 13 [Glycine max]
Ach15g377011	PREDICTED: peroxisomal membrane protein 13 [Glycine max]
Ach22g246571	PREDICTED: peroxygenase [Jatropha curcas]
Ach23g397311	PREDICTED: persulfide dioxygenase ETHE1 homolog, mitochondrial-like [Jatropha curcas]
Ach00g008371	PREDICTED: pescadillo homolog [Nelumbo nucifera]
Ach02g118571	PREDICTED: pescadillo homolog [Sesamum indicum]
Ach00g464961	PREDICTED: PHD finger protein MALE STERILITY 1 [Vitis vinifera]
Ach25g316731	PREDICTED: PHD finger protein MALE STERILITY 1 [Vitis vinifera]
Ach10g255191	PREDICTED: pheophytinase, chloroplastic [Nelumbo nucifera]
Ach00g384861	PREDICTED: phosphatase IMPL1, chloroplastic isoform X2 [Prunus mume]
Ach00g249031	PREDICTED: phosphatidylinositol 4-phosphate 5-kinase 9 [Vitis vinifera]
Ach06g176071	PREDICTED: phosphatidylserine decarboxylase proenzyme 2-like [Vitis vinifera]
Ach27g297261	PREDICTED: phosphoglucan phosphatase LSF1, chloroplastic isoform X1 [Vitis vinifera]
Ach19g305831	PREDICTED: phosphoglycerate kinase, chloroplastic [Sesamum indicum]
Ach02g036511	PREDICTED: phosphoinositide phosphatase SAC8 [Nicotiana sylvestris]
Ach03g055431	PREDICTED: phosphoinositide phospholipase C 2-like isoform X2 [Nicotiana sylvestris]
Ach08g150651	PREDICTED: phosphoribosylglycinamide formyltransferase, chloroplastic [Vitis vinifera]
Ach15g219631	PREDICTED: piezo-type mechanosensitive ion channel homolog isoform X1 [Vitis vinifera]
Ach00g411601	PREDICTED: plasma membrane-associated cation-binding protein 1 [Eucalyptus grandis]

Ach00g222881	PREDICTED: plasma membrane-associated cation-binding protein 1 [Sesamum indicum]
Ach00g385741	PREDICTED: PLASMODESMATA CALLOSE-BINDING PROTEIN 5-like isoform X4 [Nicotiana tomentosiformis]
Ach16g113041	PREDICTED: plastidic ATP/ADP-transporter-like [Nicotiana sylvestris]
Ach03g138981	PREDICTED: pleckstrin homology domain-containing protein 1 [Sesamum indicum]
Ach26g420931	PREDICTED: pleiotropic drug resistance protein 2-like [Erythranthe guttatus]
Ach08g149561	PREDICTED: pleiotropic drug resistance protein 2-like [Eucalyptus grandis]
Ach17g139601	PREDICTED: polyadenylate-binding protein-interacting protein 8-like isoform X4 [Solanum lycopersicum]
Ach02g118501	PREDICTED: polyribonucleotide nucleotidyltransferase 2, mitochondrial [Pyrus x bretschneideri]
Ach03g417901	PREDICTED: PP2A regulatory subunit TAP46 [Sesamum indicum]
Ach00g456271	PREDICTED: PRA1 family protein G2 [Sesamum indicum]
Ach22g246581	PREDICTED: pre-mRNA-processing factor 19 [Vitis vinifera]
Ach26g078401	PREDICTED: pre-mRNA-processing factor 39 [Vitis vinifera]
Ach00g467011	PREDICTED: pre-mRNA-processing factor 39-like isoform X3 [Glycine max]
Ach13g308971	PREDICTED: preprotein translocase subunit SCY2, chloroplastic isoform X1 [Gossypium raimondii]
Ach07g006381	PREDICTED: PRKR-interacting protein 1 [Nicotiana sylvestris]
Ach21g230191	PREDICTED: probable 1-acylglycerol-3-phosphate O-acyltransferase [Nelumbo nucifera]
Ach09g020311	PREDICTED: probable 26S proteasome non-ATPase regulatory subunit 3 [Nelumbo nucifera]
Ach19g406411	PREDICTED: probable 28S rRNA (cytosine(4447)-C(5))-methyltransferase [Jatropha curcas]
Ach16g113071	PREDICTED: probable 2-oxoglutarate-dependent dioxygenase AOP1 [Nicotiana sylvestris]
Ach05g235511	PREDICTED: probable adenylate kinase 7, mitochondrial [Vitis vinifera]
Ach20g213551	PREDICTED: probable ADP-ribosylation factor GTPase-activating protein AGD14 [Vitis vinifera]
Ach01g467601	PREDICTED: probable amino-acid acetyltransferase NAGS1, chloroplastic [Vitis vinifera]
Ach00g006941	PREDICTED: probable aquaporin PIP2-5 [Pyrus x bretschneideri]
Ach04g282861	PREDICTED: probable arabinosyltransferase ARAD1 [Nelumbo nucifera]
Ach13g349561	PREDICTED: probable arabinosyltransferase ARAD1 isoform X2 [Eucalyptus grandis]

Ach20g270921	PREDICTED: probable auxin efflux carrier component 1b [Vitis vinifera]
Ach00g282591	PREDICTED: probable beta-1,3-galactosyltransferase 6 [Vitis vinifera]
Ach28g284001	PREDICTED: probable beta-D-xylosidase 2 isoform X1 [Vitis vinifera]
Ach00g289891	PREDICTED: probable BOI-related E3 ubiquitin-protein ligase 3 [Nicotiana tomentosiformis]
Ach00g281361	PREDICTED: probable carbohydrate esterase At4g34215 [Sesamum indicum]
Ach15g306761	PREDICTED: probable CCR4-associated factor 1 homolog 9 [Nelumbo nucifera]
Ach00g353311	PREDICTED: probable DNA primase large subunit [Vitis vinifera]
Ach24g082931	PREDICTED: probable E3 ubiquitin-protein ligase LUL4-like [Citrus sinensis]
Ach07g476681	PREDICTED: probable F-box protein At3g61730 isoform X2 [Vitis vinifera]
Ach25g374401	PREDICTED: probable fructose-bisphosphate aldolase 3, chloroplastic [Sesamum indicum]
Ach17g191901	PREDICTED: probable galacturonosyltransferase 6-like isoform X1 [Solanum tuberosum]
Ach05g197881	PREDICTED: probable galacturonosyltransferase-like 10 [Vitis vinifera]
Ach04g329281	PREDICTED: probable glucuronoxylan glucuronosyltransferase F8H [Nelumbo nucifera]
Ach15g314681	PREDICTED: probable glutathione S-transferase parA [Prunus mume]
Ach18g433971	PREDICTED: probable LRR receptor-like serine/threonine-protein kinase At1g07650 [Prunus mume]
Ach17g418401	PREDICTED: probable LRR receptor-like serine/threonine-protein kinase At1g56140 [Nicotiana tomentosiformis]
Ach24g320961	PREDICTED: probable LRR receptor-like serine/threonine-protein kinase At1g67720 [Vitis vinifera]
Ach18g433981	PREDICTED: probable LRR receptor-like serine/threonine-protein kinase RFK1 isoform X2 [Nicotiana sylvestris]
Ach08g056741	PREDICTED: probable LRR receptor-like serine/threonine-protein kinase RKF3 [Fragaria vesca subsp. vesca]
Ach08g419141	PREDICTED: probable LRR receptor-like serine/threonine-protein kinase RKF3 [Nelumbo nucifera]
Ach08g056731	PREDICTED: probable LRR receptor-like serine/threonine-protein kinase RKF3 [Sesamum indicum]
Ach08g056751	PREDICTED: probable LRR receptor-like serine/threonine-protein kinase RKF3 [Sesamum indicum]

Ach08g419131	PREDICTED: probable LRR receptor-like serine/threonine-protein kinase RKF3 [Vitis vinifera]
Ach08g056771	PREDICTED: probable LRR receptor-like serine/threonine-protein kinase RKF3 isoform X1 [Gossypium raimondii]
Ach22g408201	PREDICTED: probable membrane-associated kinase regulator 2 [Jatropha curcas]
Ach03g008071	PREDICTED: probable methionine--tRNA ligase, mitochondrial [Vitis vinifera]
Ach04g120781	PREDICTED: probable methyltransferase PMT24 [Vitis vinifera]
Ach05g048051	PREDICTED: probable mitochondrial adenine nucleotide transporter BTL3 [Nelumbo nucifera]
Ach04g087491	PREDICTED: probable mitochondrial adenine nucleotide transporter BTL3 [Prunus mume]
Ach21g266681	PREDICTED: probable mitochondrial adenine nucleotide transporter BTL3 isoform X1 [Beta vulgaris subsp. vulgaris]
Ach04g197751	PREDICTED: probable mitochondrial chaperone BCS1-B [Sesamum indicum]
Ach22g351951	PREDICTED: probable pectate lyase 4 [Populus euphratica]
Ach14g189321	PREDICTED: probable potassium transporter 2 isoform X2 [Phoenix dactylifera]
Ach08g381571	PREDICTED: probable protein arginine N-methyltransferase 3 [Prunus mume]
Ach22g059511	PREDICTED: probable protein phosphatase 2C 42 [Vitis vinifera]
Ach00g104901	PREDICTED: probable protein phosphatase 2C 63 [Jatropha curcas]
Ach27g118061	PREDICTED: probable protein phosphatase 2C 63 [Vitis vinifera]
Ach27g271451	PREDICTED: probable protein S-acyltransferase 22 [Vitis vinifera]
Ach27g029511	PREDICTED: probable protein S-acyltransferase 7 [Sesamum indicum]
Ach12g192251	PREDICTED: probable pyruvate kinase, cytosolic isozyme [Nelumbo nucifera]
Ach00g237491	PREDICTED: probable receptor protein kinase TMK1 [Nicotiana sylvestris]
Ach00g311241	PREDICTED: probable receptor-like protein kinase At1g67000 [Nelumbo nucifera]
Ach07g224191	PREDICTED: probable rhamnogalacturonate lyase B [Sesamum indicum]
Ach07g224171	PREDICTED: probable rhamnogalacturonate lyase B isoform X1 [Jatropha curcas]
Ach18g004521	PREDICTED: probable ribose-5-phosphate isomerase 4, chloroplastic [Vitis vinifera]
Ach05g229271	PREDICTED: probable RNA 3'-terminal phosphate cyclase-like protein isoform X2 [Jatropha curcas]
Ach00g377441	PREDICTED: probable serine/threonine-protein kinase At1g54610 isoform X2 [Nelumbo nucifera]

Ach08g040351	PREDICTED: probable serine/threonine-protein kinase At5g41260 [Vitis vinifera]
Ach03g185761	PREDICTED: probable serine/threonine-protein phosphatase 2A regulatory subunit B'' subunit TON2 [Phoenix dactylifera]
Ach24g202321	PREDICTED: probable small nuclear ribonucleoprotein F [Fragaria vesca subsp. vesca]
Ach15g411961	PREDICTED: probable sodium/metabolite cotransporter BASS3, chloroplastic [Sesamum indicum]
Ach24g233991	PREDICTED: probable transcriptional regulator SLK2 isoform X3 [Pyrus x bretschneideri]
Ach00g056241	PREDICTED: probable U3 small nucleolar RNA-associated protein 7 isoform X1 [Nicotiana tomentosiformis]
Ach17g418341	PREDICTED: probable ubiquitin-conjugating enzyme E2 26 [Vitis vinifera]
Ach00g165131	PREDICTED: probable WRKY transcription factor 14 [Vitis vinifera]
Ach12g071331	PREDICTED: probable WRKY transcription factor 20 isoform X1 [Vitis vinifera]
Ach15g184451	PREDICTED: probable WRKY transcription factor 41 [Populus euphratica]
Ach01g229941	PREDICTED: probable WRKY transcription factor 7 [Vitis vinifera]
Ach00g318171	PREDICTED: programmed cell death protein 2 isoform X2 [Nelumbo nucifera]
Ach00g462231	PREDICTED: prohibitin-1, mitochondrial [Nicotiana sylvestris]
Ach28g409501	PREDICTED: proline-rich protein PRCC [Nicotiana sylvestris]
Ach11g252791	PREDICTED: prolyl endopeptidase isoform X1 [Vitis vinifera]
Ach25g268721	PREDICTED: prolyl endopeptidase-like isoform X1 [Nelumbo nucifera]
Ach00g324531	PREDICTED: protease Do-like 1, chloroplastic isoform X1 [Nelumbo nucifera]
Ach22g451551	PREDICTED: protease Do-like 10, mitochondrial [Nelumbo nucifera]
Ach18g003221	PREDICTED: proteasome subunit alpha type-4 [Cucumis sativus]
Ach08g297741	PREDICTED: proteasome subunit beta type-1-like [Sesamum indicum]
Ach25g400151	PREDICTED: protein ABIL2-like [Populus euphratica]
Ach05g053261	PREDICTED: protein BASIC PENTACYSTEINE6-like [Sesamum indicum]
Ach05g048531	PREDICTED: protein CHROMATIN REMODELING 24 [Jatropha curcas]
Ach26g077931	PREDICTED: protein CHROMATIN REMODELING 8 [Jatropha curcas]
Ach05g445701	PREDICTED: protein CLP1 homolog {ECO:0000255|HAMAP-Rule:MF_03035} [Nicotiana tomentosiformis]
Ach29g033161	PREDICTED: protein decapping 5 [Vitis vinifera]

Ach00g052541	PREDICTED: protein disulfide isomerase-like 1-4 [Populus euphratica]
Ach16g113361	PREDICTED: protein disulfide isomerase-like 1-6 [Vitis vinifera]
Ach05g048691	PREDICTED: protein disulfide-isomerase [Nelumbo nucifera]
Ach21g117261	PREDICTED: protein disulfide-isomerase [Vitis vinifera]
Ach00g364931	PREDICTED: protein disulfide-isomerase like 2-2-like [Tarenaya hassleriana]
Ach23g166681	PREDICTED: protein Dr1 homolog [Vitis vinifera]
Ach17g192081	PREDICTED: protein ELC [Vitis vinifera]
Ach21g266791	PREDICTED: protein EXORDIUM-like [Nicotiana tomentosiformis]
Ach11g216741	PREDICTED: protein FAM32A-like isoform X1 [Vitis vinifera]
Ach16g212481	PREDICTED: protein FAM32A-like isoform X1 [Vitis vinifera]
Ach15g001711	PREDICTED: protein FAM63A-like [Nelumbo nucifera]
Ach28g409491	PREDICTED: protein FAR1-RELATED SEQUENCE 5 [Jatropha curcas]
Ach28g409481	PREDICTED: protein FAR1-RELATED SEQUENCE 5 [Vitis vinifera]
Ach15g001701	PREDICTED: protein FIZZY-RELATED 2 [Vitis vinifera]
Ach27g338511	PREDICTED: protein furry homolog-like [Nicotiana tomentosiformis]
Ach03g081581	PREDICTED: protein GLC8 isoform X1 [Nicotiana tomentosiformis]
Ach24g319851	PREDICTED: protein HOTHEAD-like isoform X2 [Nicotiana sylvestris]
Ach24g407181	PREDICTED: protein HOTHEAD-like isoform X2 [Nicotiana sylvestris]
Ach20g247331	PREDICTED: protein IQ-DOMAIN 1 [Jatropha curcas]
Ach28g431951	PREDICTED: protein IQ-DOMAIN 1-like [Gossypium raimondii]
Ach08g066791	PREDICTED: protein IQ-DOMAIN 1-like [Vitis vinifera]
Ach00g070151	PREDICTED: protein IQ-DOMAIN 32 isoform X1 [Vitis vinifera]
Ach20g270901	PREDICTED: protein kinase and PP2C-like domain-containing protein [Vitis vinifera]
Ach11g252671	PREDICTED: protein LURP-one-related 10-like [Gossypium raimondii]
Ach23g482431	PREDICTED: protein MIZU-KUSSEI 1 [Vitis vinifera]
Ach00g311081	PREDICTED: protein MKS1 [Vitis vinifera]
Ach04g120911	PREDICTED: protein MKS1-like [Nicotiana tomentosiformis]
Ach00g417321	PREDICTED: protein NAP1 isoform X1 [Sesamum indicum]
Ach15g184391	PREDICTED: protein NLP7 isoform X1 [Sesamum indicum]
Ach23g042791	PREDICTED: protein N-lysine methyltransferase METTL21A-like [Solanum tuberosum]
Ach00g307221	PREDICTED: protein NRT1/ PTR FAMILY 5.6 [Nelumbo nucifera]
Ach28g123671	PREDICTED: protein NRT1/ PTR FAMILY 7.1-like [Populus euphratica]
Ach15g378491	PREDICTED: protein N-terminal asparagine amidohydrolase-like [Prunus mume]

Ach18g003901	PREDICTED: protein PELOTA 1 isoform X2 [Sesamum indicum]
Ach22g431881	PREDICTED: protein PHR1-LIKE 1 isoform X2 [Nelumbo nucifera]
Ach00g106391	PREDICTED: protein PIR [Sesamum indicum]
Ach00g480721	PREDICTED: protein PIR-like [Glycine max]
Ach00g361441	PREDICTED: protein prune homolog isoform X1 [Vitis vinifera]
Ach00g131251	PREDICTED: protein RADIALIS-like 3 [Sesamum indicum]
Ach00g461231	PREDICTED: protein RER1A-like [Nicotiana sylvestris]
Ach16g216411	PREDICTED: protein SAWADEE HOMEODOMAIN HOMOLOG 1 isoform X2 [Vitis vinifera]
Ach23g437031	PREDICTED: protein SCAI isoform X1 [Gossypium raimondii]
Ach00g066871	PREDICTED: protein SENSITIVITY TO RED LIGHT REDUCED 1 [Vitis vinifera]
Ach00g249061	PREDICTED: protein SHOOT GRAVITROPISM 6 isoform X1 [Vitis vinifera]
Ach03g198321	PREDICTED: protein SMG7-like [Vitis vinifera]
Ach18g051971	PREDICTED: protein SPA1-RELATED 2 isoform X1 [Vitis vinifera]
Ach23g429881	PREDICTED: protein SPIRAL1-like 1 [Nicotiana tomentosiformis]
Ach00g194641	PREDICTED: protein SPIRAL1-like 1 [Tarenaya hassleriana]
Ach13g388941	PREDICTED: protein SULFUR DEFICIENCY-INDUCED 1 [Jatropha curcas]
Ach22g015961	PREDICTED: protein TIC 20-II, chloroplastic [Nelumbo nucifera]
Ach08g031241	PREDICTED: protein TRANSPORT INHIBITOR RESPONSE 1 [Vitis vinifera]
Ach14g416611	PREDICTED: protein transport protein Sec61 subunit beta [Cucumis sativus]
Ach12g061691	PREDICTED: protein transport protein SFT2-like isoform X2 [Jatropha curcas]
Ach00g454881	PREDICTED: protein unc-50 homolog isoform X2 [Nelumbo nucifera]
Ach07g006371	PREDICTED: protein unc-50 homolog isoform X2 [Nelumbo nucifera]
Ach00g067311	PREDICTED: protein UPSTREAM OF FLC [Nicotiana sylvestris]
Ach17g133061	PREDICTED: protein VACUOLELESS1 isoform X2 [Nelumbo nucifera]
Ach28g148631	PREDICTED: protein YLS3 [Nelumbo nucifera]
Ach24g296561	PREDICTED: protein YLS7-like [Citrus sinensis]
Ach25g177711	PREDICTED: protein ZGRF1-like isoform X2 [Nicotiana sylvestris]
Ach00g076701	PREDICTED: protein-tyrosine-phosphatase MKP1 [Vitis vinifera]
Ach29g386731	PREDICTED: psbB mRNA maturation factor Mbb1, chloroplastic [Vitis vinifera]
Ach20g452521	PREDICTED: psbP domain-containing protein 6, chloroplastic [Vitis vinifera]
Ach02g118611	PREDICTED: pullulanase 1, chloroplastic isoform X1 [Vitis vinifera]
Ach18g448261	PREDICTED: puromycin-sensitive aminopeptidase [Cicer arietinum]

Ach27g322401	PREDICTED: putative 1-phosphatidylinositol-3-phosphate 5-kinase FAB1C [Vitis vinifera]
Ach04g456801	PREDICTED: putative 1-phosphatidylinositol-3-phosphate 5-kinase FAB1D [Vitis vinifera]
Ach10g400551	PREDICTED: putative 3,4-dihydroxy-2-butanone kinase-like [Solanum tuberosum]
Ach03g185411	PREDICTED: putative E3 ubiquitin-protein ligase RF298 [Vitis vinifera]
Ach29g237201	PREDICTED: putative E3 ubiquitin-protein ligase RF298 [Vitis vinifera]
Ach08g264721	PREDICTED: putative E3 ubiquitin-protein ligase XBAT31 [Vitis vinifera]
Ach03g334571	PREDICTED: putative E3 ubiquitin-protein ligase XBAT34 isoform X2 [Nicotiana tomentosiformis]
Ach13g352421	PREDICTED: putative E3 ubiquitin-protein ligase XBAT35 isoform X2 [Nicotiana sylvestris]
Ach13g280071	PREDICTED: putative germin-like protein 2-1 [Vitis vinifera]
Ach15g001271	PREDICTED: putative glycerol-3-phosphate transporter 4 [Vitis vinifera]
Ach06g400881	PREDICTED: putative glycosyltransferase 3 [Cucumis melo]
Ach06g400871	PREDICTED: putative glycosyltransferase 5 [Jatropha curcas]
Ach26g395711	PREDICTED: putative lysine-specific demethylase JMJ16 isoform X4 [Vitis vinifera]
Ach02g393561	PREDICTED: putative nuclear matrix constituent protein 1-like protein isoform X2 [Vitis vinifera]
Ach15g228111	PREDICTED: putative nuclease HARBI1 [Vitis vinifera]
Ach22g135821	PREDICTED: putative pectinesterase/pectinesterase inhibitor 24 [Jatropha curcas]
Ach16g443161	PREDICTED: putative pentatricopeptide repeat-containing protein At1g16830 [Vitis vinifera]
Ach11g217011	PREDICTED: putative pentatricopeptide repeat-containing protein At1g16830-like isoform X1 [Solanum tuberosum]
Ach00g162321	PREDICTED: putative pentatricopeptide repeat-containing protein At3g15930 [Vitis vinifera]
Ach24g148211	PREDICTED: putative pentatricopeptide repeat-containing protein At3g25970 [Vitis vinifera]
Ach05g197781	PREDICTED: putative pentatricopeptide repeat-containing protein At3g28640 [Vitis vinifera]
Ach22g439181	PREDICTED: putative pentatricopeptide repeat-containing protein At5g37570 [Vitis vinifera]
Ach00g282461	PREDICTED: putative peptidyl-tRNA hydrolase PTRHD1 [Gossypium raimondii]
Ach00g274031	PREDICTED: putative phagocytic receptor 1b [Solanum lycopersicum]

Ach23g166651	PREDICTED: putative phospholipid-transporting ATPase 8 isoform X2 [Nicotiana tomentosiformis]
Ach02g086281	PREDICTED: putative phytosulfokines 6 [Vitis vinifera]
Ach23g122101	PREDICTED: putative pre-mRNA-splicing factor ATP-dependent RNA helicase DHX16 isoform X1 [Sesamum indicum]
Ach13g417271	PREDICTED: putative quinone-oxidoreductase homolog, chloroplastic [Erythranthe guttatus]
Ach15g377251	PREDICTED: putative quinone-oxidoreductase homolog, chloroplastic [Fragaria vesca subsp. vesca]
Ach15g377261	PREDICTED: putative quinone-oxidoreductase homolog, chloroplastic [Populus euphratica]
Ach29g197151	PREDICTED: putative ripening-related protein 1 [Nicotiana tomentosiformis]
Ach29g197171	PREDICTED: putative ripening-related protein 1 [Populus euphratica]
Ach03g419321	PREDICTED: putative serine/threonine-protein kinase-like protein CCR3 [Sesamum indicum]
Ach03g055101	PREDICTED: putative serine/threonine-protein kinase-like protein CCR3, partial [Pyrus x bretschneideri]
Ach24g148201	PREDICTED: putative sodium-coupled neutral amino acid transporter 7 [Jatropha curcas]
Ach01g088091	PREDICTED: putative transcription factor bHLH086 [Vitis vinifera]
Ach15g314281	PREDICTED: putative tRNA pseudouridine synthase [Gossypium raimondii]
Ach11g252591	PREDICTED: putative tRNA pseudouridine synthase Pus10 [Nelumbo nucifera]
Ach03g081571	PREDICTED: putative uncharacterized protein DDB_G0287975 isoform X1 [Solanum lycopersicum]
Ach13g388951	PREDICTED: putative vesicle-associated membrane protein 726 [Eucalyptus grandis]
Ach04g087631	PREDICTED: pyridoxine/pyridoxamine 5'-phosphate oxidase 1, chloroplastic [Vitis vinifera]
Ach01g307921	PREDICTED: pyrophosphate-energized vacuolar membrane proton pump [Nicotiana sylvestris]
Ach28g303631	PREDICTED: ran-binding protein 1 homolog b-like [Vitis vinifera]
Ach07g157321	PREDICTED: ras-related protein Rab11D [Vitis vinifera]
Ach18g156821	PREDICTED: ras-related protein RABA1d [Vitis vinifera]
Ach17g051281	PREDICTED: ras-related protein RABA1f [Elaeis guineensis]
Ach00g008391	PREDICTED: ras-related protein RABB1b [Vitis vinifera]
Ach29g237221	PREDICTED: receptor-like cytosolic serine/threonine-protein kinase RBK2-like [Solanum tuberosum]
Ach26g108521	PREDICTED: receptor-like protein kinase HERK 1-like [Glycine max]
Ach00g345481	PREDICTED: receptor-like protein kinase HSL1 [Nicotiana sylvestris]

Ach00g345491	PREDICTED: receptor-like protein kinase HSL1 [Sesamum indicum]
Ach14g308531	PREDICTED: regulator of nonsense transcripts 1 homolog isoform X1 [Vitis vinifera]
Ach00g388731	PREDICTED: regulatory-associated protein of TOR 1 [Prunus mume]
Ach00g162651	PREDICTED: reticuline oxidase-like protein [Vitis vinifera]
Ach00g281351	PREDICTED: reticulon-like protein B11 [Solanum lycopersicum]
Ach15g184581	PREDICTED: reticulon-like protein B2 [Vitis vinifera]
Ach28g409521	PREDICTED: rhodanese-like domain-containing protein 4A, chloroplastic [Vitis vinifera]
Ach00g151731	PREDICTED: riboflavin synthase [Vitis vinifera]
Ach10g143281	PREDICTED: ribonuclease H2 subunit B isoform X1 [Gossypium raimondii]
Ach23g121991	PREDICTED: ribulose-phosphate 3-epimerase, chloroplastic [Sesamum indicum]
Ach02g180101	PREDICTED: RING finger and transmembrane domain-containing protein 1 [Erythranthe guttatus]
Ach11g265791	PREDICTED: RING-H2 finger protein ATL11-like [Nicotiana sylvestris]
Ach11g126221	PREDICTED: RING-H2 finger protein ATL43 [Vitis vinifera]
Ach23g024631	PREDICTED: RING-H2 finger protein ATL5 [Vitis vinifera]
Ach00g072161	PREDICTED: RING-H2 finger protein ATL66 [Gossypium raimondii]
Ach19g306241	PREDICTED: RNA polymerase II degradation factor 1 isoform X3 [Vitis vinifera]
Ach07g470591	PREDICTED: RNA pseudouridine synthase 4, mitochondrial isoform X2 [Nicotiana sylvestris]
Ach24g442331	PREDICTED: RNA-binding protein 24 isoform X3 [Jatropha curcas]
Ach21g211461	PREDICTED: RNA-binding protein 38-like [Nelumbo nucifera]
Ach09g401341	PREDICTED: rop guanine nucleotide exchange factor 1 [Vitis vinifera]
Ach24g141181	PREDICTED: rop guanine nucleotide exchange factor 5 [Vitis vinifera]
Ach15g002071	PREDICTED: rop guanine nucleotide exchange factor 7 [Prunus mume]
Ach22g408211	PREDICTED: rRNA-processing protein fcf2 [Solanum lycopersicum]
Ach13g461841	PREDICTED: ruBisCO large subunit-binding protein subunit alpha [Populus euphratica]
Ach28g278001	PREDICTED: ruBisCO large subunit-binding protein subunit beta, chloroplastic [Fragaria vesca subsp. vesca]
Ach23g115571	PREDICTED: ruBisCO large subunit-binding protein subunit beta, chloroplastic [Vitis vinifera]
Ach26g301091	PREDICTED: secoisolariciresinol dehydrogenase-like [Vitis vinifera]
Ach22g059731	PREDICTED: seed maturation protein PM36 [Nicotiana tomentosiformis]

Ach24g203921	PREDICTED: serine carboxypeptidase 24 isoform X3 [Solanum lycopersicum]
Ach03g153661	PREDICTED: serine carboxypeptidase-like 27 [Vitis vinifera]
Ach23g121301	PREDICTED: serine/arginine repetitive matrix protein 2 [Prunus mume]
Ach03g185471	PREDICTED: serine/arginine-rich SC35-like splicing factor SCL28 isoform X2 [Populus euphratica]
Ach24g082831	PREDICTED: serine/arginine-rich SC35-like splicing factor SCL28 isoform X3 [Nicotiana sylvestris]
Ach23g355091	PREDICTED: serine/threonine-protein kinase 16 [Eucalyptus grandis]
Ach25g177621	PREDICTED: serine/threonine-protein kinase BLUS1-like isoform X2 [Vitis vinifera]
Ach00g282741	PREDICTED: serine/threonine-protein kinase BLUS1-like isoform X2 [Vitis vinifera]
Ach14g382621	PREDICTED: serine/threonine-protein kinase CDL1-like isoform X1 [Vitis vinifera]
Ach12g028831	PREDICTED: serine/threonine-protein kinase cst-1 isoform X2 [Vitis vinifera]
Ach02g086761	PREDICTED: serine/threonine-protein kinase CTR1 [Vitis vinifera]
Ach22g272831	PREDICTED: serine/threonine-protein kinase D6PKL2-like [Eucalyptus grandis]
Ach15g184631	PREDICTED: serine/threonine-protein kinase GIN4 [Nicotiana sylvestris]
Ach08g150641	PREDICTED: serine/threonine-protein kinase HT1 [Jatropha curcas]
Ach01g254891	PREDICTED: serine/threonine-protein kinase HT1 [Vitis vinifera]
Ach24g082461	PREDICTED: serine/threonine-protein kinase ULK4 [Vitis vinifera]
Ach00g223081	PREDICTED: serine/threonine-protein phosphatase 7 long form homolog [Sesamum indicum]
Ach00g367201	PREDICTED: serine/threonine-protein phosphatase 7 long form homolog [Sesamum indicum]
Ach04g087591	PREDICTED: serine--tRNA ligase, mitochondrial [Jatropha curcas]
Ach27g297321	PREDICTED: shaggy-related protein kinase epsilon-like isoform X2 [Phoenix dactylifera]
Ach21g094941	PREDICTED: small nuclear ribonucleoprotein Sm D3 [Gossypium raimondii]
Ach00g259981	PREDICTED: small nuclear ribonucleoprotein Sm D3-like [Musa acuminata subsp. malaccensis]
Ach01g191041	PREDICTED: small subunit processome component 20 homolog isoform X1 [Vitis vinifera]
Ach01g439021	PREDICTED: small subunit processome component 20 homolog isoform X1 [Vitis vinifera]
Ach23g121421	PREDICTED: SNAP25 homologous protein SNAP33 [Nicotiana sylvestris]
Ach00g242491	PREDICTED: sodium/hydrogen exchanger 8 [Nelumbo nucifera]

Ach21g405941	PREDICTED: sodium-coupled neutral amino acid transporter 5-like [Solanum lycopersicum]
Ach23g171921	PREDICTED: sodium-dependent phosphate transport protein 1, chloroplastic isoform X1 [Jatropha curcas]
Ach06g169911	PREDICTED: somatic embryogenesis receptor kinase 2-like isoform X2 [Gossypium raimondii]
Ach08g237811	PREDICTED: sphinganine C(4)-monooxygenase 1-like isoform X2 [Camelina sativa]
Ach00g361431	PREDICTED: sphingoid long-chain bases kinase 1 [Vitis vinifera]
Ach09g201781	PREDICTED: splicing factor 3B subunit 4 [Nelumbo nucifera]
Ach05g254451	PREDICTED: splicing factor U2af large subunit B isoform X2 [Sesamum indicum]
Ach22g190751	PREDICTED: splicing factor U2AF-associated protein 2 isoform X2 [Eucalyptus grandis]
Ach24g034131	PREDICTED: splicing factor U2AF-associated protein 2 isoform X2 [Nelumbo nucifera]
Ach24g203731	PREDICTED: sporulation protein RMD1 [Nelumbo nucifera]
Ach15g002541	PREDICTED: SPX domain-containing membrane protein At4g22990 [Vitis vinifera]
Ach10g270251	PREDICTED: squalene monooxygenase [Vitis vinifera]
Ach10g270271	PREDICTED: squalene monooxygenase-like [Eucalyptus grandis]
Ach04g343391	PREDICTED: squamosa promoter-binding-like protein 14 isoform X2 [Vitis vinifera]
Ach03g185971	PREDICTED: squamosa promoter-binding-like protein 7 [Vitis vinifera]
Ach00g443021	PREDICTED: STE20/SPS1-related proline-alanine-rich protein kinase isoform X1 [Nicotiana sylvestris]
Ach00g165171	PREDICTED: SUMO-activating enzyme subunit 2 [Prunus mume]
Ach06g231841	PREDICTED: superkiller viralicidic activity 2-like 2 isoform X2 [Sesamum indicum]
Ach24g422221	PREDICTED: superoxide dismutase [Cu-Zn] [Eucalyptus grandis]
Ach13g279091	PREDICTED: syntaxin-124-like [Nicotiana tomentosiformis]
Ach12g058911	PREDICTED: syntaxin-132 [Sesamum indicum]
Ach03g240051	PREDICTED: syntaxin-22-like isoform X2 [Jatropha curcas]
Ach13g301811	PREDICTED: TBC1 domain family member 13 [Vitis vinifera]
Ach14g027121	PREDICTED: T-complex protein 1 subunit alpha [Vitis vinifera]
Ach16g017381	PREDICTED: tetratricopeptide repeat protein 1-like [Fragaria vesca subsp. vesca]
Ach25g468641	PREDICTED: tetratricopeptide repeat protein 5-like isoform X2 [Gossypium raimondii]
Ach05g445731	PREDICTED: thioredoxin H2-like [Solanum tuberosum]
Ach28g248161	PREDICTED: THUMP domain-containing protein 1 isoform X1 [Vitis vinifera]

Ach08g152581	PREDICTED: thylakoid ADP,ATP carrier protein, chloroplastic [Nelumbo nucifera]
Ach00g035381	PREDICTED: thymidylate kinase isoform X2 [Nelumbo nucifera]
Ach25g409691	PREDICTED: TIMELESS-interacting protein [Nicotiana tomentosiformis]
Ach13g329591	PREDICTED: trafficking protein particle complex subunit 5 [Pyrus x bretschneideri]
Ach23g042811	PREDICTED: transcription activator GLK1-like isoform X1 [Fragaria vesca subsp. vesca]
Ach03g062891	PREDICTED: transcription elongation factor A protein 2-like [Vitis vinifera]
Ach20g368981	PREDICTED: transcription factor bHLH35-like [Pyrus x bretschneideri]
Ach26g300901	PREDICTED: transcription factor E2FB [Vitis vinifera]
Ach00g035361	PREDICTED: transcription factor GTE8 isoform X1 [Vitis vinifera]
Ach02g416961	PREDICTED: transcription factor HBP-1b(c38)-like [Jatropha curcas]
Ach22g324811	PREDICTED: transcription factor MYB108-like [Populus euphratica]
Ach23g171911	PREDICTED: transcription factor MYB29 [Nelumbo nucifera]
Ach07g006361	PREDICTED: transcription factor MYB44 [Vitis vinifera]
Ach00g281601	PREDICTED: transcription factor MYB44-like [Prunus mume]
Ach00g101161	PREDICTED: transcription factor RF2a-like [Solanum tuberosum]
Ach05g047841	PREDICTED: transcription factor TGA1-like, partial [Sesamum indicum]
Ach19g199461	PREDICTED: transcription initiation factor TFIID subunit 12b-like isoform X2 [Populus euphratica]
Ach05g476341	PREDICTED: transcription initiation factor TFIID subunit 14b-like isoform X6 [Populus euphratica]
Ach29g130141	PREDICTED: transcription repressor OFP11 [Vitis vinifera]
Ach15g001751	PREDICTED: transcriptional corepressor SEUSS isoform X1 [Malus domestica]
Ach05g159591	PREDICTED: transformation/transcription domain-associated protein-like isoform X1 [Solanum tuberosum]
Ach15g154081	PREDICTED: transient receptor potential protein-like isoform X1 [Tarenaya hassleriana]
Ach00g462241	PREDICTED: translation factor GUF1 homolog, organellar chromatophore [Nelumbo nucifera]
Ach00g106401	PREDICTED: translational activator GCN1 [Jatropha curcas]
Ach09g201011	PREDICTED: transmembrane and coiled-coil domain-containing protein 4 isoform X2 [Vitis vinifera]
Ach12g168041	PREDICTED: transmembrane protein 184 homolog DDB_G0279555 isoform X2 [Sesamum indicum]
Ach00g377451	PREDICTED: transmembrane protein 230 [Populus euphratica]
Ach27g338621	PREDICTED: transmembrane protein 33 homolog [Jatropha curcas]

Ach06g247061	PREDICTED: transmembrane protein 87B [Nelumbo nucifera]
Ach04g087131	PREDICTED: transport inhibitor response 1-like protein [Vitis vinifera]
Ach03g186001	PREDICTED: transposon TX1 uncharacterized 149 kDa protein [Vitis vinifera]
Ach12g192201	PREDICTED: triacylglycerol lipase SDP1-like [Vitis vinifera]
Ach19g199081	PREDICTED: trihelix transcription factor ASIL1-like [Vitis vinifera]
Ach02g020701	PREDICTED: trihelix transcription factor GT-1 [Nelumbo nucifera]
Ach20g092541	PREDICTED: trihelix transcription factor PTL [Vitis vinifera]
Ach07g434361	PREDICTED: tRNA (cytosine(34)-C(5))-methyltransferase [Vitis vinifera]
Ach16g021351	PREDICTED: tryptophan aminotransferase-related protein 2 [Vitis vinifera]
Ach03g081211	PREDICTED: tubulin beta-4A chain [Bison bison bison]
Ach25g226451	PREDICTED: two-component response regulator ARR1 isoform X1 [Vitis vinifera]
Ach15g314671	PREDICTED: two-component response regulator ARR17 isoform X2 [Malus domestica]
Ach22g246661	PREDICTED: two-component response regulator-like APRR7 [Jatropha curcas]
Ach14g458641	PREDICTED: type I inositol 1,4,5-trisphosphate 5-phosphatase 2-like isoform X3 [Nelumbo nucifera]
Ach23g360041	PREDICTED: type I inositol 1,4,5-trisphosphate 5-phosphatase CVP2 isoform X1 [Vitis vinifera]
Ach18g004041	PREDICTED: U11/U12 small nuclear ribonucleoprotein 48 kDa protein [Vitis vinifera]
Ach18g394061	PREDICTED: U11/U12 small nuclear ribonucleoprotein 48 kDa protein isoform X1 [Populus euphratica]
Ach21g029131	PREDICTED: ubinuclein-2-like isoform X1 [Vitis vinifera]
Ach00g278831	PREDICTED: ubiquitin carboxyl-terminal hydrolase 12 isoform X1 [Vitis vinifera]
Ach20g404361	PREDICTED: ubiquitin carboxyl-terminal hydrolase 12 isoform X2 [Jatropha curcas]
Ach15g134721	PREDICTED: ubiquitin carboxyl-terminal hydrolase 24 [Sesamum indicum]
Ach24g407831	PREDICTED: ubiquitin carboxyl-terminal hydrolase isozyme L3 [Jatropha curcas]
Ach28g248221	PREDICTED: ubiquitin-conjugating enzyme E2 20-like [Sesamum indicum]
Ach29g386261	PREDICTED: ubiquitin-like-specific protease ESD4 [Cucumis sativus]
Ach17g051601	PREDICTED: ubiquitin-NEDD8-like protein RUB2 [Camelina sativa]
Ach04g121091	PREDICTED: U-box domain-containing protein 28-like [Nicotiana sylvestris]

Ach13g352431	PREDICTED: U-box domain-containing protein 3 [Vitis vinifera]
Ach18g071781	PREDICTED: U-box domain-containing protein 33-like isoform X4 [Jatropha curcas]
Ach00g289531	PREDICTED: UBP1-associated protein 2A-like [Nelumbo nucifera]
Ach00g242451	PREDICTED: UBX domain-containing protein 2 isoform X1 [Prunus mume]
Ach18g166241	PREDICTED: UDP-galactose transporter 2-like [Nelumbo nucifera]
Ach00g459801	PREDICTED: UDP-glucose 4-epimerase GEPI48-like [Nicotiana sylvestris]
Ach02g293001	PREDICTED: UDP-glycosyltransferase 83A1 [Vitis vinifera]
Ach26g402891	PREDICTED: UDP-glycosyltransferase 92A1-like [Vitis vinifera]
Ach15g109201	PREDICTED: UNC93-like protein 3 [Vitis vinifera]
Ach15g109221	PREDICTED: UNC93-like protein 3 [Vitis vinifera]
Ach24g141291	PREDICTED: uncharacterized acetyltransferase At3g50280-like [Nicotiana tomentosiformis]
Ach16g035981	PREDICTED: uncharacterized GPI-anchored protein At4g28100 [Vitis vinifera]
Ach25g080821	PREDICTED: uncharacterized protein At1g04910 [Eucalyptus grandis]
Ach00g344531	PREDICTED: uncharacterized protein At1g04910 isoform X1 [Vitis vinifera]
Ach18g244121	PREDICTED: uncharacterized protein At1g76660 [Nicotiana tomentosiformis]
Ach18g003111	PREDICTED: uncharacterized protein At2g02148 isoform X2 [Nelumbo nucifera]
Ach24g322251	PREDICTED: uncharacterized protein At3g49055 [Vitis vinifera]
Ach03g045521	PREDICTED: uncharacterized protein At4g00950 [Prunus mume]
Ach15g306691	PREDICTED: uncharacterized protein At5g41620 [Vitis vinifera]
Ach00g297851	PREDICTED: uncharacterized protein LOC100240757 [Vitis vinifera]
Ach18g156811	PREDICTED: uncharacterized protein LOC100241189 [Vitis vinifera]
Ach00g078841	PREDICTED: uncharacterized protein LOC100241254 [Vitis vinifera]
Ach00g422141	PREDICTED: uncharacterized protein LOC100241254 [Vitis vinifera]
Ach23g172351	PREDICTED: uncharacterized protein LOC100241609 [Vitis vinifera]
Ach03g403451	PREDICTED: uncharacterized protein LOC100242230 [Vitis vinifera]
Ach27g088821	PREDICTED: uncharacterized protein LOC100242361 [Vitis vinifera]
Ach04g441021	PREDICTED: uncharacterized protein LOC100242657 isoform X1 [Vitis vinifera]

Ach17g064511	PREDICTED: uncharacterized protein LOC100243435 [Vitis vinifera]
Ach29g414031	PREDICTED: uncharacterized protein LOC100244229 isoform X1 [Vitis vinifera]
Ach24g041081	PREDICTED: uncharacterized protein LOC100244302 isoform X3 [Vitis vinifera]
Ach24g082721	PREDICTED: uncharacterized protein LOC100244334 [Vitis vinifera]
Ach00g218361	PREDICTED: uncharacterized protein LOC100244977 [Vitis vinifera]
Ach24g202211	PREDICTED: uncharacterized protein LOC100245448 [Vitis vinifera]
Ach00g377641	PREDICTED: uncharacterized protein LOC100245710 [Vitis vinifera]
Ach11g225671	PREDICTED: uncharacterized protein LOC100245761 isoform X1 [Vitis vinifera]
Ach18g244261	PREDICTED: uncharacterized protein LOC100245966 [Vitis vinifera]
Ach13g417281	PREDICTED: uncharacterized protein LOC100246086 isoform X2 [Vitis vinifera]
Ach17g051611	PREDICTED: uncharacterized protein LOC100246156 isoform X3 [Vitis vinifera]
Ach24g202791	PREDICTED: uncharacterized protein LOC100246256 [Vitis vinifera]
Ach00g456251	PREDICTED: uncharacterized protein LOC100246258 [Vitis vinifera]
Ach23g435331	PREDICTED: uncharacterized protein LOC100247040 isoform X2 [Vitis vinifera]
Ach22g325321	PREDICTED: uncharacterized protein LOC100247647 [Vitis vinifera]
Ach27g353711	PREDICTED: uncharacterized protein LOC100248040 [Vitis vinifera]
Ach05g193391	PREDICTED: uncharacterized protein LOC100248040 [Vitis vinifera]
Ach13g280281	PREDICTED: uncharacterized protein LOC100248242 [Vitis vinifera]
Ach15g005651	PREDICTED: uncharacterized protein LOC100248343 [Vitis vinifera]
Ach23g113671	PREDICTED: uncharacterized protein LOC100248456 [Vitis vinifera]
Ach23g428241	PREDICTED: uncharacterized protein LOC100248456 [Vitis vinifera]
Ach24g319861	PREDICTED: uncharacterized protein LOC100248586 [Vitis vinifera]

Ach16g364961	PREDICTED: uncharacterized protein LOC100249354 isoform X1 [Vitis vinifera]
Ach01g365471	PREDICTED: uncharacterized protein LOC100249441 isoform X1 [Vitis vinifera]
Ach24g202681	PREDICTED: uncharacterized protein LOC100249661 [Vitis vinifera]
Ach23g172441	PREDICTED: uncharacterized protein LOC100250176 [Vitis vinifera]
Ach15g002741	PREDICTED: uncharacterized protein LOC100250510 isoform X1 [Vitis vinifera]
Ach00g323561	PREDICTED: uncharacterized protein LOC100250825 isoform X2 [Vitis vinifera]
Ach22g443761	PREDICTED: uncharacterized protein LOC100251145 [Vitis vinifera]
Ach00g475681	PREDICTED: uncharacterized protein LOC100251314 isoform X2 [Vitis vinifera]
Ach23g068901	PREDICTED: uncharacterized protein LOC100251420 [Vitis vinifera]
Ach00g194711	PREDICTED: uncharacterized protein LOC100251831 isoform X2 [Vitis vinifera]
Ach06g232051	PREDICTED: uncharacterized protein LOC100251997 [Vitis vinifera]
Ach24g233721	PREDICTED: uncharacterized protein LOC100252183 [Vitis vinifera]
Ach08g433061	PREDICTED: uncharacterized protein LOC100252669 [Vitis vinifera]
Ach16g216501	PREDICTED: uncharacterized protein LOC100253221 [Vitis vinifera]
Ach00g340211	PREDICTED: uncharacterized protein LOC100253681 [Vitis vinifera]
Ach08g297711	PREDICTED: uncharacterized protein LOC100254100 [Vitis vinifera]
Ach21g117231	PREDICTED: uncharacterized protein LOC100254101 [Vitis vinifera]
Ach27g089201	PREDICTED: uncharacterized protein LOC100254358 [Vitis vinifera]
Ach08g398661	PREDICTED: uncharacterized protein LOC100254761 [Vitis vinifera]
Ach02g180381	PREDICTED: uncharacterized protein LOC100255300 [Vitis vinifera]
Ach28g419721	PREDICTED: uncharacterized protein LOC100255365 [Vitis vinifera]
Ach07g340301	PREDICTED: uncharacterized protein LOC100255496 [Vitis vinifera]

Ach20g120631	PREDICTED: uncharacterized protein LOC100255893 isoform X1 [Vitis vinifera]
Ach22g272861	PREDICTED: uncharacterized protein LOC100256822 [Vitis vinifera]
Ach00g240431	PREDICTED: uncharacterized protein LOC100257848 [Vitis vinifera]
Ach23g445191	PREDICTED: uncharacterized protein LOC100258138 isoform X2 [Vitis vinifera]
Ach25g284671	PREDICTED: uncharacterized protein LOC100259190 [Vitis vinifera]
Ach29g033171	PREDICTED: uncharacterized protein LOC100259365 [Vitis vinifera]
Ach00g105221	PREDICTED: uncharacterized protein LOC100259962 [Vitis vinifera]
Ach10g143021	PREDICTED: uncharacterized protein LOC100260404 [Vitis vinifera]
Ach24g407481	PREDICTED: uncharacterized protein LOC100260794 [Vitis vinifera]
Ach24g204261	PREDICTED: uncharacterized protein LOC100260933 [Vitis vinifera]
Ach05g048681	PREDICTED: uncharacterized protein LOC100261237 isoform X1 [Vitis vinifera]
Ach24g465681	PREDICTED: uncharacterized protein LOC100261746 [Vitis vinifera]
Ach16g335441	PREDICTED: uncharacterized protein LOC100262175 isoform X1 [Vitis vinifera]
Ach04g439981	PREDICTED: uncharacterized protein LOC100262487 isoform X1 [Vitis vinifera]
Ach21g396611	PREDICTED: uncharacterized protein LOC100262535 [Vitis vinifera]
Ach03g154361	PREDICTED: uncharacterized protein LOC100262578 [Vitis vinifera]
Ach05g307251	PREDICTED: uncharacterized protein LOC100262596 isoform X2 [Vitis vinifera]
Ach21g266671	PREDICTED: uncharacterized protein LOC100262645 [Vitis vinifera]
Ach24g407851	PREDICTED: uncharacterized protein LOC100262848 isoform X1 [Vitis vinifera]
Ach19g198661	PREDICTED: uncharacterized protein LOC100263481 isoform X6 [Vitis vinifera]
Ach00g194741	PREDICTED: uncharacterized protein LOC100263840 isoform X3 [Vitis vinifera]
Ach21g094931	PREDICTED: uncharacterized protein LOC100264304 [Vitis vinifera]

Ach00g317011	PREDICTED: uncharacterized protein LOC100264575 [Vitis vinifera]
Ach11g225651	PREDICTED: uncharacterized protein LOC100264575 [Vitis vinifera]
Ach15g002111	PREDICTED: uncharacterized protein LOC100264910 [Vitis vinifera]
Ach23g347371	PREDICTED: uncharacterized protein LOC100265339 [Vitis vinifera]
Ach04g316131	PREDICTED: uncharacterized protein LOC100266128 isoform X1 [Vitis vinifera]
Ach16g365171	PREDICTED: uncharacterized protein LOC100266414 isoform X2 [Vitis vinifera]
Ach24g082731	PREDICTED: uncharacterized protein LOC100266667 [Vitis vinifera]
Ach00g324391	PREDICTED: uncharacterized protein LOC100267992 [Vitis vinifera]
Ach28g409581	PREDICTED: uncharacterized protein LOC100852824 [Vitis vinifera]
Ach24g203741	PREDICTED: uncharacterized protein LOC100852955 isoform X1 [Vitis vinifera]
Ach00g082271	PREDICTED: uncharacterized protein LOC100854177 [Vitis vinifera]
Ach05g396281	PREDICTED: uncharacterized protein LOC100854642 isoform X2 [Vitis vinifera]
Ach15g438111	PREDICTED: uncharacterized protein LOC101210365 isoform X2 [Cucumis sativus]
Ach15g376931	PREDICTED: uncharacterized protein LOC101247846 [Solanum lycopersicum]
Ach13g352321	PREDICTED: uncharacterized protein LOC101250730 [Solanum lycopersicum]
Ach23g121411	PREDICTED: uncharacterized protein LOC101258762 [Solanum lycopersicum]
Ach03g410581	PREDICTED: uncharacterized protein LOC101268739 [Solanum lycopersicum]
Ach15g418051	PREDICTED: uncharacterized protein LOC101493395 [Cicer arietinum]
Ach08g297731	PREDICTED: uncharacterized protein LOC102580329 [Solanum tuberosum]
Ach13g035151	PREDICTED: uncharacterized protein LOC102582294 [Solanum tuberosum]
Ach26g443531	PREDICTED: uncharacterized protein LOC102592857 [Solanum tuberosum]
Ach27g401981	PREDICTED: uncharacterized protein LOC102600784 [Solanum tuberosum]

Ach00g101231	PREDICTED: uncharacterized protein LOC102601837 [Solanum tuberosum]
Ach05g047611	PREDICTED: uncharacterized protein LOC102604432 [Solanum tuberosum]
Ach01g307971	PREDICTED: uncharacterized protein LOC102622022 [Citrus sinensis]
Ach15g392361	PREDICTED: uncharacterized protein LOC102626352 isoform X2 [Citrus sinensis]
Ach28g321581	PREDICTED: uncharacterized protein LOC102627405 [Citrus sinensis]
Ach26g033671	PREDICTED: uncharacterized protein LOC102627691 [Citrus sinensis]
Ach15g314701	PREDICTED: uncharacterized protein LOC102629445 [Citrus sinensis]
Ach04g249421	PREDICTED: uncharacterized protein LOC102659506 [Glycine max]
Ach00g146401	PREDICTED: uncharacterized protein LOC102666990 [Glycine max]
Ach00g344091	PREDICTED: uncharacterized protein LOC102670332 [Glycine max]
Ach00g218391	PREDICTED: uncharacterized protein LOC103321768 isoform X1 [Prunus mume]
Ach22g246631	PREDICTED: uncharacterized protein LOC103323855 [Prunus mume]
Ach16g459111	PREDICTED: uncharacterized protein LOC103328095 isoform X2 [Prunus mume]
Ach19g424781	PREDICTED: uncharacterized protein LOC103328095 isoform X2 [Prunus mume]
Ach17g133271	PREDICTED: uncharacterized protein LOC103329100 [Prunus mume]
Ach05g235461	PREDICTED: uncharacterized protein LOC103330348 [Prunus mume]
Ach05g446781	PREDICTED: uncharacterized protein LOC103330348 [Prunus mume]
Ach03g188031	PREDICTED: uncharacterized protein LOC103333790 [Prunus mume]
Ach00g457141	PREDICTED: uncharacterized protein LOC103337169 [Prunus mume]
Ach19g138621	PREDICTED: uncharacterized protein LOC103338302 [Prunus mume]
Ach09g201451	PREDICTED: uncharacterized protein LOC103340246, partial [Prunus mume]
Ach29g164591	PREDICTED: uncharacterized protein LOC103341094 [Prunus mume]

Ach02g180111	PREDICTED: uncharacterized protein LOC103342587 [Prunus mume]
Ach00g466361	PREDICTED: uncharacterized protein LOC103403603 [Malus domestica]
Ach25g438471	PREDICTED: uncharacterized protein LOC103423381 [Malus domestica]
Ach15g109511	PREDICTED: uncharacterized protein LOC103433379 [Malus domestica]
Ach24g407801	PREDICTED: uncharacterized protein LOC103437052 [Malus domestica]
Ach15g184501	PREDICTED: uncharacterized protein LOC103440146 [Malus domestica]
Ach16g017341	PREDICTED: uncharacterized protein LOC103445027 [Malus domestica]
Ach18g156521	PREDICTED: uncharacterized protein LOC103696520 [Phoenix dactylifera]
Ach27g170331	PREDICTED: uncharacterized protein LOC103697676 [Phoenix dactylifera]
Ach24g203401	PREDICTED: uncharacterized protein LOC103926661 [Pyrus x bretschneideri]
Ach24g203411	PREDICTED: uncharacterized protein LOC103926661 [Pyrus x bretschneideri]
Ach00g484281	PREDICTED: uncharacterized protein LOC103933717 [Pyrus x bretschneideri]
Ach00g282731	PREDICTED: uncharacterized protein LOC103949307 [Pyrus x bretschneideri]
Ach11g128051	PREDICTED: uncharacterized protein LOC104093294 isoform X2 [Nicotiana tomentosiformis]
Ach00g105211	PREDICTED: uncharacterized protein LOC104094238 [Nicotiana tomentosiformis]
Ach00g082321	PREDICTED: uncharacterized protein LOC104097749 [Nicotiana tomentosiformis]
Ach04g367921	PREDICTED: uncharacterized protein LOC104100576 [Nicotiana tomentosiformis]
Ach08g283081	PREDICTED: uncharacterized protein LOC104103776 [Nicotiana tomentosiformis]
Ach15g002921	PREDICTED: uncharacterized protein LOC104103818 [Nicotiana tomentosiformis]
Ach21g029371	PREDICTED: uncharacterized protein LOC104104703 [Nicotiana tomentosiformis]
Ach15g451031	PREDICTED: uncharacterized protein LOC104106126 isoform X1 [Nicotiana tomentosiformis]
Ach08g040501	PREDICTED: uncharacterized protein LOC104106742 [Nicotiana tomentosiformis]

Ach08g150691	PREDICTED: uncharacterized protein LOC104108893 [Nicotiana tomentosiformis]
Ach08g150681	PREDICTED: uncharacterized protein LOC104108895 [Nicotiana tomentosiformis]
Ach28g148611	PREDICTED: uncharacterized protein LOC104112297 [Nicotiana tomentosiformis]
Ach08g238201	PREDICTED: uncharacterized protein LOC104112987 [Nicotiana tomentosiformis]
Ach24g141191	PREDICTED: uncharacterized protein LOC104113405 [Nicotiana tomentosiformis]
Ach10g143101	PREDICTED: uncharacterized protein LOC104114070 [Nicotiana tomentosiformis]
Ach10g255181	PREDICTED: uncharacterized protein LOC104114922 [Nicotiana tomentosiformis]
Ach12g435901	PREDICTED: uncharacterized protein LOC104115430 [Nicotiana tomentosiformis]
Ach26g068371	PREDICTED: uncharacterized protein LOC104210260 [Nicotiana sylvestris]
Ach15g427111	PREDICTED: uncharacterized protein LOC104212095 [Nicotiana sylvestris]
Ach22g273021	PREDICTED: uncharacterized protein LOC104214663 [Nicotiana sylvestris]
Ach00g444831	PREDICTED: uncharacterized protein LOC104221408 [Nicotiana sylvestris]
Ach00g340041	PREDICTED: uncharacterized protein LOC104221718 [Nicotiana sylvestris]
Ach00g311311	PREDICTED: uncharacterized protein LOC104225578 [Nicotiana sylvestris]
Ach15g111701	PREDICTED: uncharacterized protein LOC104229610 [Nicotiana sylvestris]
Ach00g316431	PREDICTED: uncharacterized protein LOC104230825 [Nicotiana sylvestris]
Ach15g001411	PREDICTED: uncharacterized protein LOC104233751 [Nicotiana sylvestris]
Ach15g228101	PREDICTED: uncharacterized protein LOC104238943 [Nicotiana sylvestris]
Ach00g377551	PREDICTED: uncharacterized protein LOC104241132 [Nicotiana sylvestris]
Ach13g350791	PREDICTED: uncharacterized protein LOC104244399 [Nicotiana sylvestris]
Ach23g427631	PREDICTED: uncharacterized protein LOC104244504 [Nicotiana sylvestris]
Ach00g307491	PREDICTED: uncharacterized protein LOC104246594 isoform X2 [Nicotiana sylvestris]

Ach00g360911	PREDICTED: uncharacterized protein LOC104246952 [Nicotiana sylvestris]
Ach27g211041	PREDICTED: uncharacterized protein LOC104249122 [Nicotiana sylvestris]
Ach08g426201	PREDICTED: uncharacterized protein LOC104441193 isoform X2 [Eucalyptus grandis]
Ach00g194581	PREDICTED: uncharacterized protein LOC104445137 [Eucalyptus grandis]
Ach12g167621	PREDICTED: uncharacterized protein LOC104450974 [Eucalyptus grandis]
Ach28g248211	PREDICTED: uncharacterized protein LOC104589587 isoform X1 [Nelumbo nucifera]
Ach00g477871	PREDICTED: uncharacterized protein LOC104590110 [Nelumbo nucifera]
Ach18g003911	PREDICTED: uncharacterized protein LOC104590676 [Nelumbo nucifera]
Ach00g038211	PREDICTED: uncharacterized protein LOC104592497 isoform X4 [Nelumbo nucifera]
Ach27g089401	PREDICTED: uncharacterized protein LOC104592935 [Nelumbo nucifera]
Ach13g018381	PREDICTED: uncharacterized protein LOC104593479 [Nelumbo nucifera]
Ach01g449671	PREDICTED: uncharacterized protein LOC104594511 [Nelumbo nucifera]
Ach04g197531	PREDICTED: uncharacterized protein LOC104595729 [Nelumbo nucifera]
Ach28g038361	PREDICTED: uncharacterized protein LOC104599235 [Nelumbo nucifera]
Ach28g474551	PREDICTED: uncharacterized protein LOC104599235 [Nelumbo nucifera]
Ach23g172371	PREDICTED: uncharacterized protein LOC104599327 [Nelumbo nucifera]
Ach23g172741	PREDICTED: uncharacterized protein LOC104601103 isoform X2 [Nelumbo nucifera]
Ach28g142031	PREDICTED: uncharacterized protein LOC104601427 [Nelumbo nucifera]
Ach28g070421	PREDICTED: uncharacterized protein LOC104602750 [Nelumbo nucifera]
Ach21g396551	PREDICTED: uncharacterized protein LOC104603826 [Nelumbo nucifera]
Ach07g240761	PREDICTED: uncharacterized protein LOC104604213 [Nelumbo nucifera]
Ach00g307521	PREDICTED: uncharacterized protein LOC104604777 [Nelumbo nucifera]

Ach09g201021	PREDICTED: uncharacterized protein LOC104607530 [Nelumbo nucifera]
Ach24g358651	PREDICTED: uncharacterized protein LOC104608792 [Nelumbo nucifera]
Ach16g212391	PREDICTED: uncharacterized protein LOC104609046 [Nelumbo nucifera]
Ach23g385081	PREDICTED: uncharacterized protein LOC104610239 [Nelumbo nucifera]
Ach00g477391	PREDICTED: uncharacterized protein LOC104610772 [Nelumbo nucifera]
Ach00g411591	PREDICTED: uncharacterized protein LOC104649603 [Solanum lycopersicum]
Ach22g016211	PREDICTED: uncharacterized protein LOC104877596 [Vitis vinifera]
Ach08g426191	PREDICTED: uncharacterized protein LOC104878530 isoform X2 [Vitis vinifera]
Ach13g308961	PREDICTED: uncharacterized protein LOC104879777 [Vitis vinifera]
Ach12g119961	PREDICTED: uncharacterized protein LOC104880154 [Vitis vinifera]
Ach24g407811	PREDICTED: uncharacterized protein LOC104881044 [Vitis vinifera]
Ach22g273051	PREDICTED: uncharacterized protein LOC104881178 [Vitis vinifera]
Ach15g184521	PREDICTED: uncharacterized protein LOC104881234 [Vitis vinifera]
Ach26g271041	PREDICTED: uncharacterized protein LOC104882376 [Vitis vinifera]
Ach12g071361	PREDICTED: uncharacterized protein LOC104884024 [Beta vulgaris subsp. vulgaris]
Ach05g374831	PREDICTED: uncharacterized protein LOC105039750 [Elaeis guineensis]
Ach29g054531	PREDICTED: uncharacterized protein LOC105049253 isoform X4 [Elaeis guineensis]
Ach00g104891	PREDICTED: uncharacterized protein LOC105108889 [Populus euphratica]
Ach00g042361	PREDICTED: uncharacterized protein LOC105111326 isoform X1 [Populus euphratica]
Ach23g172451	PREDICTED: uncharacterized protein LOC105111383 [Populus euphratica]
Ach00g230491	PREDICTED: uncharacterized protein LOC105115019 [Populus euphratica]
Ach16g397001	PREDICTED: uncharacterized protein LOC105118819 [Populus euphratica]

Ach04g343731	PREDICTED: uncharacterized protein LOC105119907 [Populus euphratica]
Ach24g313061	PREDICTED: uncharacterized protein LOC105121539 [Populus euphratica]
Ach15g002151	PREDICTED: uncharacterized protein LOC105122005 isoform X2 [Populus euphratica]
Ach22g408301	PREDICTED: uncharacterized protein LOC105156818 [Sesamum indicum]
Ach20g236591	PREDICTED: uncharacterized protein LOC105156886 [Sesamum indicum]
Ach16g426861	PREDICTED: uncharacterized protein LOC105157559 [Sesamum indicum]
Ach16g113351	PREDICTED: uncharacterized protein LOC105157642 [Sesamum indicum]
Ach16g017111	PREDICTED: uncharacterized protein LOC105158223 isoform X2 [Sesamum indicum]
Ach13g394341	PREDICTED: uncharacterized protein LOC105159627 [Sesamum indicum]
Ach06g028591	PREDICTED: uncharacterized protein LOC105160026 [Sesamum indicum]
Ach24g465691	PREDICTED: uncharacterized protein LOC105160492 [Sesamum indicum]
Ach00g278971	PREDICTED: uncharacterized protein LOC105161016 [Sesamum indicum]
Ach17g173141	PREDICTED: uncharacterized protein LOC105161922 isoform X2 [Sesamum indicum]
Ach20g424891	PREDICTED: uncharacterized protein LOC105165005 [Sesamum indicum]
Ach27g464921	PREDICTED: uncharacterized protein LOC105165891 [Sesamum indicum]
Ach13g454151	PREDICTED: uncharacterized protein LOC105167377 [Sesamum indicum]
Ach27g456831	PREDICTED: uncharacterized protein LOC105167711 isoform X2 [Sesamum indicum]
Ach05g198111	PREDICTED: uncharacterized protein LOC105168755 isoform X3 [Sesamum indicum]
Ach07g340291	PREDICTED: uncharacterized protein LOC105169840 [Sesamum indicum]
Ach15g376701	PREDICTED: uncharacterized protein LOC105170116 [Sesamum indicum]
Ach29g130081	PREDICTED: uncharacterized protein LOC105171329 [Sesamum indicum]
Ach22g214621	PREDICTED: uncharacterized protein LOC105172030 [Sesamum indicum]

Ach14g027271	PREDICTED: uncharacterized protein LOC105173291 [Sesamum indicum]
Ach00g289631	PREDICTED: uncharacterized protein LOC105435263 isoform X1 [Cucumis sativus]
Ach21g299231	PREDICTED: uncharacterized protein LOC105630802 isoform X2 [Jatropha curcas]
Ach00g471031	PREDICTED: uncharacterized protein LOC105633686 [Jatropha curcas]
Ach00g130941	PREDICTED: uncharacterized protein LOC105635369 [Jatropha curcas]
Ach21g462721	PREDICTED: uncharacterized protein LOC105637143 [Jatropha curcas]
Ach00g065451	PREDICTED: uncharacterized protein LOC105637599 isoform X2 [Jatropha curcas]
Ach00g411581	PREDICTED: uncharacterized protein LOC105639541 [Jatropha curcas]
Ach22g462931	PREDICTED: uncharacterized protein LOC105641877 [Jatropha curcas]
Ach23g226181	PREDICTED: uncharacterized protein LOC105646135 isoform X2 [Jatropha curcas]
Ach19g260331	PREDICTED: uncharacterized protein LOC105647443 [Jatropha curcas]
Ach28g123851	PREDICTED: uncharacterized protein LOC105648257 [Jatropha curcas]
Ach03g420001	PREDICTED: uncharacterized protein LOC105649233 [Jatropha curcas]
Ach00g082291	PREDICTED: uncharacterized protein LOC105649405 [Jatropha curcas]
Ach00g484271	PREDICTED: uncharacterized protein LOC105771969 [Gossypium raimondii]
Ach11g397161	PREDICTED: uncharacterized protein LOC105772121, partial [Gossypium raimondii]
Ach24g319381	PREDICTED: uncharacterized protein LOC105785549 [Gossypium raimondii]
Ach16g365031	PREDICTED: uncharacterized protein LOC105788673 isoform X1 [Gossypium raimondii]
Ach13g229501	PREDICTED: uncharacterized protein LOC105802911 [Gossypium raimondii]
Ach00g164771	PREDICTED: uncharacterized protein LOC105951618 isoform X2 [Erythranthe guttatus]
Ach19g034321	PREDICTED: uncharacterized protein LOC105955367 [Erythranthe guttatus]
Ach19g474091	PREDICTED: uncharacterized protein LOC105955367 [Erythranthe guttatus]

Ach00g315991	PREDICTED: uncharacterized protein LOC105959686 [Erythranthe guttatus]
Ach27g353651	PREDICTED: uncharacterized protein LOC105961060 [Erythranthe guttatus]
Ach22g016101	PREDICTED: uncharacterized protein LOC105961341 [Erythranthe guttatus]
Ach13g146761	PREDICTED: uncharacterized protein LOC105962165 [Erythranthe guttatus]
Ach00g358511	PREDICTED: uncharacterized protein LOC105969838 [Erythranthe guttatus]
Ach16g212511	PREDICTED: uncharacterized protein LOC105975664 [Erythranthe guttatus]
Ach00g307551	PREDICTED: uncharacterized WD repeat-containing protein C2A9.03-like [Nelumbo nucifera]
Ach25g400171	PREDICTED: UPF0160 protein C694.04c [Nelumbo nucifera]
Ach00g209481	PREDICTED: UPF0160 protein C694.04c [Nelumbo nucifera]
Ach08g031261	PREDICTED: vacuolar protein 8 [Vitis vinifera]
Ach18g003131	PREDICTED: vacuolar protein sorting-associated protein 2 homolog 2 [Cucumis sativus]
Ach18g394251	PREDICTED: vacuolar protein sorting-associated protein 2 homolog 2 [Vitis vinifera]
Ach00g361471	PREDICTED: vacuolar protein sorting-associated protein 54, chloroplastic-like [Malus domestica]
Ach03g244571	PREDICTED: vesicle transport v-SNARE 13-like [Sesamum indicum]
Ach15g392601	PREDICTED: vesicle-associated protein 2-1-like isoform X3 [Nelumbo nucifera]
Ach17g051441	PREDICTED: vesicle-fusing ATPase [Jatropha curcas]
Ach17g051421	PREDICTED: vesicle-fusing ATPase [Sesamum indicum]
Ach05g476351	PREDICTED: villin-2 [Vitis vinifera]
Ach05g235411	PREDICTED: villin-4-like [Malus domestica]
Ach20g092031	PREDICTED: VIN3-like protein 2 [Vitis vinifera]
Ach12g464891	PREDICTED: vinorine synthase-like [Populus euphratica]
Ach05g374881	PREDICTED: violaxanthin de-epoxidase, chloroplastic-like [Populus euphratica]
Ach21g446021	PREDICTED: V-type proton ATPase 16 kDa proteolipid subunit [Cucumis melo]
Ach11g217001	PREDICTED: V-type proton ATPase catalytic subunit A [Nelumbo nucifera]
Ach16g212381	PREDICTED: V-type proton ATPase catalytic subunit A [Nelumbo nucifera]
Ach00g052551	PREDICTED: V-type proton ATPase subunit G 1-like [Sesamum indicum]
Ach00g305081	PREDICTED: WAT1-related protein At1g21890 [Vitis vinifera]

Ach06g176131	PREDICTED: WAT1-related protein At1g68170, partial [Nelumbo nucifera]
Ach00g305071	PREDICTED: WAT1-related protein At4g08290 [Vitis vinifera]
Ach06g230941	PREDICTED: WD repeat-containing protein 74 [Fragaria vesca subsp. vesca]
Ach21g095491	PREDICTED: WD repeat-containing protein DWA2 isoform X2 [Vitis vinifera]
Ach06g247151	PREDICTED: WD-40 repeat-containing protein MSI1 [Sesamum indicum]
Ach24g141001	PREDICTED: WUSCHEL-related homeobox 5 [Sesamum indicum]
Ach28g070411	PREDICTED: xyloglucan galactosyltransferase KATAMARI1-like [Glycine max]
Ach20g136311	PREDICTED: zeatin O-glucosyltransferase-like [Nicotiana tomentosiformis]
Ach19g097521	PREDICTED: zinc finger BED domain-containing protein DAYSLEEPER-like [Vitis vinifera]
Ach15g364791	PREDICTED: zinc finger CCCH domain-containing protein 11 [Nelumbo nucifera]
Ach26g241461	PREDICTED: zinc finger CCCH domain-containing protein 14-like [Jatropha curcas]
Ach00g015191	PREDICTED: zinc finger CCCH domain-containing protein 17 [Vitis vinifera]
Ach13g146491	PREDICTED: zinc finger CCCH domain-containing protein 37 isoform X1 [Nelumbo nucifera]
Ach23g429871	PREDICTED: zinc finger CCCH domain-containing protein 38 [Eucalyptus grandis]
Ach00g460871	PREDICTED: zinc finger CCCH domain-containing protein 41 [Vitis vinifera]
Ach23g347651	PREDICTED: zinc finger CCCH domain-containing protein 44-like [Sesamum indicum]
Ach28g123681	PREDICTED: zinc finger CCCH domain-containing protein 44-like [Sesamum indicum]
Ach24g082601	PREDICTED: zinc finger CCCH domain-containing protein 58-like [Solanum tuberosum]
Ach08g457071	PREDICTED: zinc finger CCCH domain-containing protein 69 isoform X2 [Vitis vinifera]
Ach00g480691	PREDICTED: zinc finger CCCH domain-containing protein ZFN-like [Populus euphratica]
Ach00g480701	PREDICTED: zinc finger CCCH domain-containing protein ZFN-like [Vitis vinifera]
Ach27g353861	PREDICTED: zinc finger protein 511-like [Citrus sinensis]
Ach08g030831	PREDICTED: zinc finger protein CONSTANS-LIKE 1-like isoform X1 [Sesamum indicum]
Ach00g353121	PREDICTED: zinc finger protein NUTCRACKER [Vitis vinifera]

Ach00g104831	PREDICTED: zinc finger protein ZAT10 [Vitis vinifera]
Ach00g452861	PREDICTED: zinc finger protein ZAT3 [Vitis vinifera]
Ach23g258281	PREDICTED: zinc finger protein ZAT5 [Vitis vinifera]
Ach28g123861	PREDICTED: zinc transporter ZTP29 isoform X1 [Sesamum indicum]
Ach21g128581	PREDICTED: zinc-finger homeodomain protein 4 [Nelumbo nucifera]
Ach27g338611	Proline-rich receptor protein kinase PERK10 isoform 1 [Theobroma cacao]
Ach24g204211	Protein neuralized [Morus notabilis]
Ach18g269251	Protein neuralized [Morus notabilis]
Ach03g354381	protein phosphatase 2c, putative [Ricinus communis]
Ach07g309101	protein with unknown function [Ricinus communis]
Ach27g423221	Protoporphyrinogen oxidase, chloroplastic/mitochondrial [Gossypium arboreum]
Ach20g098391	putative auxin-induced protein [Camellia sinensis]
Ach23g347011	Putative axial regulator YABBY 2 [Glycine soja]
Ach05g256731	putative chorismate mutase [Fagus sylvatica]
Ach15g002121	putative dynein light chain [Morus notabilis]
Ach13g394331	Putative H/ACA ribonucleoprotein complex subunit 1-like protein 1 [Morus notabilis]
Ach21g430101	putative hydroxycinnamoyl-CoA:shikimate/quinate hydroxycinnamoyltransferase [Camellia sinensis]
Ach29g129981	putative N-acetyltransferase p20 [Morus notabilis]
Ach03g410591	putative phosphatase 2C 75 -like protein [Gossypium arboreum]
Ach00g478021	putative protein [Arabidopsis thaliana]
Ach00g477381	putative reverse transcriptase [Arabidopsis thaliana]
Ach21g095501	putative serine/threonine-protein kinase [Morus notabilis]
Ach00g324541	putative universal stress protein [Catharanthus roseus]
Ach01g307911	pyrophosphate-energized vacuolar membrane proteon pump 1 [Ipomoea batatas]
Ach07g005981	RAB GTPase G3F isoform 1 [Theobroma cacao]
Ach28g303621	RAN BINDING protein 1 [Populus trichocarpa]
Ach26g395791	Ras-GTPase-activating protein-binding protein, putative [Ricinus communis]
Ach25g374161	RBCS1 [Actinidia chinensis]
Ach15g345761	RecName: Full=Plastid-lipid-associated protein, chloroplastic; AltName: Full=CitPAP; Flags: Precursor [Citrus unshiu]
Ach02g036521	Response to aba and salt 1, putative [Theobroma cacao]
Ach22g443771	rhomboid protein Ilepu_RBL14 [Ilex purpurea]
Ach00g385261	ring finger protein, putative [Ricinus communis]
Ach18g434011	RING/FYVE/PHD zinc finger superfamily protein, putative [Theobroma cacao]
Ach20g400061	RING/U-box superfamily protein with ARM repeat domain [Theobroma cacao]

Ach24g082571	RING/U-box superfamily protein, putative isoform 1 [Theobroma cacao]
Ach29g311811	RING/U-box superfamily protein, putative isoform 1 [Theobroma cacao]
Ach19g138121	RNA binding-like protein [Theobroma cacao]
Ach22g272781	RNA-binding (RRM/RBD/RNP motifs) family protein isoform 1 [Theobroma cacao]
Ach24g203641	RNA-binding family protein with retrovirus zinc finger-like domain isoform 3 [Theobroma cacao]
Ach00g165141	RNA-binding protein [Prochloron didemni]
Ach24g407821	Rossmann-fold NAD(P)-binding domain-containing protein isoform 1 [Theobroma cacao]
Ach00g481621	rrm/rnp domain, putative [Ricinus communis]
Ach05g048511	S-adenosyl-L-methionine-dependent methyltransferases superfamily protein [Theobroma cacao]
Ach05g048521	S-adenosyl-L-methionine-dependent methyltransferases superfamily protein [Theobroma cacao]
Ach17g133311	SCL domain class transcription factor [Theobroma cacao]
Ach08g031371	S-domain receptor-like kinase [Nicotiana tabacum]
Ach27g353871	SecE/sec61-gamma protein transport protein [Theobroma cacao]
Ach23g360031	selenocysteine methyltransferase [Camellia sinensis]
Ach04g367931	Serine/threonine-protein kinase [Theobroma cacao]
Ach03g185781	Serine/threonine-protein kinase PBS1 [Morus notabilis]
Ach15g314561	short chain alcohol dehydrogenase, putative [Ricinus communis]
Ach16g309481	Signal recognition particle, SRP54 subunit protein [Theobroma cacao]
Ach23g411051	small heat shock protein 35.9 [Boea hygrometrica]
Ach06g332641	S-methyl-5-thioribose kinase isoform 2 [Theobroma cacao]
Ach14g308611	S-norcoclaurine synthase 1 [Gossypium arboreum]
Ach08g149581	starch branching enzyme [Ipomoea batatas]
Ach08g149571	starch branching enzyme II [Ipomoea batatas]
Ach03g186231	Stem-specific TSJT1 [Gossypium arboreum]
Ach20g098581	Subtilisin-like protease [Morus notabilis]
Ach17g272541	Subtilisin-like protease, partial [Glycine soja]
Ach00g471961	sucrose responsive element binding protein [Vitis vinifera]
Ach23g024141	sucrose synthase [Actinidia deliciosa]
Ach02g036531	Syntaxin-binding protein 5 [Glycine soja]
Ach04g120851	T14N5.8 protein isoform 1 [Theobroma cacao]
Ach01g285291	TATA-binding protein-associated factor 2N [Morus notabilis]
Ach25g206551	TCP transcription factor 22 [Solanum lycopersicum]
Ach11g252581	TCP-1/cpn60 chaperonin family protein isoform 1 [Theobroma cacao]
Ach26g271091	Tetratricopeptide repeat (TPR)-like superfamily protein [Theobroma cacao]
Ach00g456981	tobamovirus multiplication protein 3 [Populus trichocarpa]

Ach29g221861	Transcription factor bHLH30 -like protein [Gossypium arboreum]
Ach00g298401	Transducin/WD40 repeat-like superfamily protein isoform 3, partial [Theobroma cacao]
Ach15g154091	Transitional endoplasmic reticulum ATPase, putative [Ricinus communis]
Ach00g281591	Translocon at inner membrane of chloroplasts 21 isoform 1 [Theobroma cacao]
Ach07g204611	transporter, putative [Ricinus communis]
Ach27g230631	trehalose-phosphate synthase 6 [Camellia sinensis]
Ach15g001421	Tryptophan RNA-binding attenuator protein-like [Theobroma cacao]
Ach15g184611	ubiquitin-conjugating enzyme E2 [Camellia sinensis]
Ach00g353541	ubiquitin-protein ligase, putative [Ricinus communis]
Ach24g203381	UDP-glucuronate decarboxylase [Camellia oleifera]
Ach00g397501	UGTPg18 [Panax ginseng]
Ach13g195261	Uncharacterized protein isoform 1 [Theobroma cacao]
Ach16g459101	Uncharacterized protein isoform 1 [Theobroma cacao]
Ach20g120721	Uncharacterized protein isoform 1 [Theobroma cacao]
Ach24g320851	Uncharacterized protein isoform 1 [Theobroma cacao]
Ach05g445721	Uncharacterized protein isoform 2 [Theobroma cacao]
Ach21g128531	Uncharacterized protein isoform 2 [Theobroma cacao]
Ach17g253251	Uncharacterized protein TCM_002409 [Theobroma cacao]
Ach27g170341	Uncharacterized protein TCM_003129 [Theobroma cacao]
Ach00g448041	Uncharacterized protein TCM_007254 [Theobroma cacao]
Ach13g307761	Uncharacterized protein TCM_011341 [Theobroma cacao]
Ach26g298071	Uncharacterized protein TCM_014645 [Theobroma cacao]
Ach06g231161	Uncharacterized protein TCM_027112 [Theobroma cacao]
Ach00g245961	Uncharacterized protein TCM_027312 [Theobroma cacao]
Ach00g289861	unknown [Lotus japonicus]
Ach00g340091	unknown [Lotus japonicus]
Ach20g447451	unknown [Lotus japonicus]
Ach29g414011	unknown [Lotus japonicus]
Ach00g458671	unknown [Medicago truncatula]
Ach13g195401	unknown [Medicago truncatula]
Ach10g449801	unknown [Populus trichocarpa x Populus deltoides]
Ach00g131261	unnamed protein product [Coffea canephora]
Ach00g131941	unnamed protein product [Coffea canephora]
Ach00g151581	unnamed protein product [Coffea canephora]
Ach00g222301	unnamed protein product [Coffea canephora]
Ach00g309231	unnamed protein product [Coffea canephora]
Ach00g332001	unnamed protein product [Coffea canephora]
Ach01g379201	unnamed protein product [Coffea canephora]
Ach03g018861	unnamed protein product [Coffea canephora]
Ach03g153671	unnamed protein product [Coffea canephora]
Ach05g449611	unnamed protein product [Coffea canephora]

Ach08g149681	unnamed protein product [Coffea canephora]
Ach08g398991	unnamed protein product [Coffea canephora]
Ach12g428501	unnamed protein product [Coffea canephora]
Ach13g308861	unnamed protein product [Coffea canephora]
Ach13g464641	unnamed protein product [Coffea canephora]
Ach15g002371	unnamed protein product [Coffea canephora]
Ach16g036011	unnamed protein product [Coffea canephora]
Ach16g212401	unnamed protein product [Coffea canephora]
Ach17g316951	unnamed protein product [Coffea canephora]
Ach18g003611	unnamed protein product [Coffea canephora]
Ach22g390741	unnamed protein product [Coffea canephora]
Ach23g068891	unnamed protein product [Coffea canephora]
Ach24g141011	unnamed protein product [Coffea canephora]
Ach24g422261	unnamed protein product [Coffea canephora]
Ach25g268731	unnamed protein product [Coffea canephora]
Ach25g268921	unnamed protein product [Coffea canephora]
Ach27g271381	unnamed protein product [Coffea canephora]
Ach00g008331	unnamed protein product [Coffea canephora]
Ach00g035611	unnamed protein product [Coffea canephora]
Ach00g038161	unnamed protein product [Coffea canephora]
Ach00g040741	unnamed protein product [Coffea canephora]
Ach00g082281	unnamed protein product [Coffea canephora]
Ach00g097131	unnamed protein product [Coffea canephora]
Ach00g105161	unnamed protein product [Coffea canephora]
Ach00g106271	unnamed protein product [Coffea canephora]
Ach00g146051	unnamed protein product [Coffea canephora]
Ach00g164831	unnamed protein product [Coffea canephora]
Ach00g240391	unnamed protein product [Coffea canephora]
Ach00g282051	unnamed protein product [Coffea canephora]
Ach00g289561	unnamed protein product [Coffea canephora]
Ach00g316321	unnamed protein product [Coffea canephora]
Ach00g318111	unnamed protein product [Coffea canephora]
Ach00g335331	unnamed protein product [Coffea canephora]
Ach00g361421	unnamed protein product [Coffea canephora]
Ach00g377541	unnamed protein product [Coffea canephora]
Ach00g457121	unnamed protein product [Coffea canephora]
Ach00g460831	unnamed protein product [Coffea canephora]
Ach00g478221	unnamed protein product [Coffea canephora]
Ach02g086291	unnamed protein product [Coffea canephora]
Ach03g045971	unnamed protein product [Coffea canephora]
Ach05g197811	unnamed protein product [Coffea canephora]
Ach05g440321	unnamed protein product [Coffea canephora]
Ach05g448861	unnamed protein product [Coffea canephora]
Ach06g182771	unnamed protein product [Coffea canephora]

Ach07g240731	unnamed protein product [Coffea canephora]
Ach07g241031	unnamed protein product [Coffea canephora]
Ach08g031251	unnamed protein product [Coffea canephora]
Ach08g031421	unnamed protein product [Coffea canephora]
Ach08g105491	unnamed protein product [Coffea canephora]
Ach08g426151	unnamed protein product [Coffea canephora]
Ach08g473691	unnamed protein product [Coffea canephora]
Ach11g404821	unnamed protein product [Coffea canephora]
Ach12g192181	unnamed protein product [Coffea canephora]
Ach12g192361	unnamed protein product [Coffea canephora]
Ach12g316621	unnamed protein product [Coffea canephora]
Ach13g279101	unnamed protein product [Coffea canephora]
Ach13g286861	unnamed protein product [Coffea canephora]
Ach14g027161	unnamed protein product [Coffea canephora]
Ach15g109501	unnamed protein product [Coffea canephora]
Ach15g184711	unnamed protein product [Coffea canephora]
Ach16g113221	unnamed protein product [Coffea canephora]
Ach17g173091	unnamed protein product [Coffea canephora]
Ach18g004501	unnamed protein product [Coffea canephora]
Ach18g166271	unnamed protein product [Coffea canephora]
Ach19g138111	unnamed protein product [Coffea canephora]
Ach19g305851	unnamed protein product [Coffea canephora]
Ach20g098111	unnamed protein product [Coffea canephora]
Ach20g160751	unnamed protein product [Coffea canephora]
Ach22g214671	unnamed protein product [Coffea canephora]
Ach22g246591	unnamed protein product [Coffea canephora]
Ach22g325331	unnamed protein product [Coffea canephora]
Ach23g042801	unnamed protein product [Coffea canephora]
Ach23g172071	unnamed protein product [Coffea canephora]
Ach23g172321	unnamed protein product [Coffea canephora]
Ach24g082611	unnamed protein product [Coffea canephora]
Ach24g141201	unnamed protein product [Coffea canephora]
Ach24g320911	unnamed protein product [Coffea canephora]
Ach26g078281	unnamed protein product [Coffea canephora]
Ach27g089221	unnamed protein product [Coffea canephora]
Ach27g456851	unnamed protein product [Coffea canephora]
Ach28g395531	unnamed protein product [Coffea canephora]
Ach29g311651	unnamed protein product [Coffea canephora]
Ach29g364311	unnamed protein product [Coffea canephora]
Ach29g431401	unnamed protein product [Coffea canephora]
Ach00g455951	unnamed protein product [Vitis vinifera]
Ach00g461721	unnamed protein product [Vitis vinifera]
Ach02g427291	unnamed protein product [Vitis vinifera]
Ach06g361981	unnamed protein product [Vitis vinifera]

Ach08g298601	unnamed protein product [Vitis vinifera]
Ach13g229491	unnamed protein product [Vitis vinifera]
Ach13g331881	unnamed protein product [Vitis vinifera]
Ach14g317601	unnamed protein product [Vitis vinifera]
Ach16g397031	unnamed protein product [Vitis vinifera]
Ach26g068121	unnamed protein product [Vitis vinifera]
Ach00g008351	unnamed protein product [Vitis vinifera]
Ach00g039741	unnamed protein product [Vitis vinifera]
Ach00g047281	unnamed protein product [Vitis vinifera]
Ach00g194721	unnamed protein product [Vitis vinifera]
Ach00g375041	unnamed protein product [Vitis vinifera]
Ach00g456261	unnamed protein product [Vitis vinifera]
Ach01g191051	unnamed protein product [Vitis vinifera]
Ach03g186291	unnamed protein product [Vitis vinifera]
Ach03g240031	unnamed protein product [Vitis vinifera]
Ach03g410511	unnamed protein product [Vitis vinifera]
Ach05g256461	unnamed protein product [Vitis vinifera]
Ach05g389831	unnamed protein product [Vitis vinifera]
Ach06g230921	unnamed protein product [Vitis vinifera]
Ach07g463841	unnamed protein product [Vitis vinifera]
Ach09g019801	unnamed protein product [Vitis vinifera]
Ach13g279081	unnamed protein product [Vitis vinifera]
Ach13g352331	unnamed protein product [Vitis vinifera]
Ach14g382281	unnamed protein product [Vitis vinifera]
Ach14g472821	unnamed protein product [Vitis vinifera]
Ach15g001321	unnamed protein product [Vitis vinifera]
Ach15g219811	unnamed protein product [Vitis vinifera]
Ach15g314291	unnamed protein product [Vitis vinifera]
Ach16g021781	unnamed protein product [Vitis vinifera]
Ach16g364951	unnamed protein product [Vitis vinifera]
Ach16g426871	unnamed protein product [Vitis vinifera]
Ach18g166261	unnamed protein product [Vitis vinifera]
Ach20g265091	unnamed protein product [Vitis vinifera]
Ach21g414691	unnamed protein product [Vitis vinifera]
Ach22g341371	unnamed protein product [Vitis vinifera]
Ach22g409601	unnamed protein product [Vitis vinifera]
Ach23g113631	unnamed protein product [Vitis vinifera]
Ach23g121311	unnamed protein product [Vitis vinifera]
Ach23g121811	unnamed protein product [Vitis vinifera]
Ach23g429821	unnamed protein product [Vitis vinifera]
Ach24g233981	unnamed protein product [Vitis vinifera]
Ach27g473421	unnamed protein product [Vitis vinifera]
Ach28g303391	unnamed protein product [Vitis vinifera]
Ach14g189241	vacuolar ATP synthase subunit [Camellia sinensis]

Ach08g152571	Vacuolar cation/proton exchanger 1a, putative [Ricinus communis]
Ach02g036621	Vacuolar cation/proton exchanger 3 [Glycine soja]
Ach17g133071	Vacuoleless1 (VCL1) isoform 2 [Theobroma cacao]
Ach15g392611	Vesicle-associated protein 2-1 [Morus notabilis]
Ach17g418351	Vesicle-fusing ATPase [Larimichthys crocea]
Ach17g155031	VTC2-like protein [Actinidia chinensis]
Ach25g268941	WD-repeat protein, putative [Ricinus communis]
Ach28g060481	WD-repeat protein, putative [Ricinus communis]
Ach00g377531	WIN1-like protein, putative [Theobroma cacao]
Ach15g314301	WRKY DNA-binding protein 33 isoform 1 [Theobroma cacao]
Ach00g285781	WUSCHEL related homeobox 2, putative [Theobroma cacao]
Ach15g376911	xyloglucan endotransglucosylase/hydrolase 7 [Actinidia deliciosa]
Ach28g070401	Xyloglucan galactosyltransferase KATAMARI1 [Morus notabilis]
Ach00g281701	YELLOW STRIPE like 1 isoform 1 [Theobroma cacao]
Ach25g316681	ZF-HD homeobox protein [Medicago truncatula]
Ach00g105181	Zinc finger protein-like 1-like protein [Morus notabilis]

Table S10. Common genes between the trichome GWAS analysis and the oceanic isolation comparison.

Population	Gene_ID	Annotation
Ocean island	Ach13g308881	PREDICTED: NF-kappa-B-activating protein [Jatropha curcas]
Ocean island	Ach13g458761	hypothetical protein M569_02206 [Genlisea aurea]
inland	Ach04g441021	PREDICTED: uncharacterized protein LOC100242657 isoform X1 [Vitis vinifera]
inland	Ach04g456801	PREDICTED: putative 1-phosphatidylinositol-3-phosphate 5-kinase FAB1D [Vitis vinifera]
inland	Ach10g270151	PREDICTED: aspartic proteinase nepenthesin-1 [Vitis vinifera]
inland	Ach10g306521	hypothetical protein MIMGU_mgv1a0017942mg, partial [Erythranthe guttata]
inland	Ach11g262871	hypothetical protein CICLE_v10001133mg [Citrus clementina]
inland	Ach22g016051	hypothetical protein SORBIDRAFT_01g050160 [Sorghum bicolor]
inland	Ach22g059741	PREDICTED: bromodomain and WD repeat-containing protein 3 isoform X1 [Vitis vinifera]
inland	Ach22g439181	PREDICTED: putative pentatricopeptide repeat-containing protein At5g37570 [Vitis vinifera]
inland	Ach23g226121	PREDICTED: 7-dehydrocholesterol reductase-like [Malus domestica]
inland	Ach23g226151	PREDICTED: histone acetyltransferase HAC1-like [Vitis vinifera]
inland	Ach23g226161	PREDICTED: organic cation/carnitine transporter 7 [Vitis vinifera]
inland	Ach23g226171	hypothetical protein PRUPE_ppa016079mg [Prunus persica]
inland	Ach23g226181	PREDICTED: uncharacterized protein LOC105646135 isoform X2 [Jatropha curcas]
inland	Ach23g445191	PREDICTED: uncharacterized protein LOC100258138 isoform X2 [Vitis vinifera]
inland	Ach24g202311	PREDICTED: myosin-9-like [Vitis vinifera]
inland	Ach24g204221	NA
inland	Ach24g442521	hypothetical protein POPTR_0008s02980g [Populus trichocarpa]
inland	Ach29g054631	NA

Table S11. Chromosomes 19 and 24 containing the highest associated single nucleotide polymorphisms (SNPs) with the trichome trait

chr	start	end	length_Signal	num_snp	peakPOS	ref	alt	maf	peak_value	peak_Effect	transcript_ID	Annotation	gene_length	distance	sig_snp_num
Chr19	2726677	2759422	32746	15	2740095	C	T	0.051471	33.33637	intronic(Ach19g305841)	Ach19g305831.1	PREDICTED: phosphoglycerate kinase, chloroplastic [Sesamum indicum]	4441	3268	15
Chr19	2726677	2759422	32746	15	2740095	C	T	0.051471	33.33637	intronic(Ach19g305841)	Ach19g305841.2	phosphoglycerate kinase [Gossypium hirsutum]	5231	0	15
Chr19	2726677	2759422	32746	15	2740095	C	T	0.051471	33.33637	intronic(Ach19g305841)	Ach19g306241.2	PREDICTED: RNA polymerase II degradation factor 1 isoform X3 [Vitis vinifera]	12041	-9319	14
Chr19	2726677	2759422	32746	15	2740095	C	T	0.051471	33.33637	intronic(Ach19g305841)	Ach19g306251.2	PREDICTED: organic cation/carnitine transporter 3-like [Nelumbo nucifera]	1653	-4671	15
Chr24	12030156	12058586	28431	9	12040393	G	A	0.356618	30.26447	downstream(Ach24g141021)	Ach24g140981.2	PREDICTED: eukaryotic translation initiation factor 3 subunit F [Cucumis melo]	12143	-17440	2
Chr24	12030156	12058586	28431	9	12040393	G	A	0.356618	30.26447	downstream(Ach24g141021)	Ach24g141001.2	PREDICTED: WUSCHEL-related homeobox 5 [Sesamum indicum]	756	-8088	9
Chr24	12030156	12058586	28431	9	12040393	G	A	0.356618	30.26447	downstream(Ach24g141021)	Ach24g141011.2	unnamed protein product [Coffea canephora]	378	-5292	9

Chr24	12030156	12058586	28431	9	12040393	G	A	0.356618	30.26447	downstream(Ach24g141021)	Ach24g141021.2	PREDICTED: DNA/RNA-binding protein KIN17 [Nicotiana sylvestris]	780	-500	9
Chr24	12030156	12058586	28431	9	12040393	G	A	0.356618	30.26447	downstream(Ach24g141021)	Ach24g141291.1	PREDICTED: uncharacterized acetyltransferase At3g50280-like [Nicotiana tomentosiformis]	1389	8319	5
Chr24	12030156	12058586	28431	9	12040393	G	A	0.356618	30.26447	downstream(Ach24g141021)	Ach24g431751.2	PREDICTED: DNA/RNA-binding protein KIN17 [Nicotiana sylvestris]	417	-1596	9
